# Synthesis of Diverse Glycosyl Bicyclo[1.1.1]pentanes Enabled by Electrochemical Functionalization of [1.1.1]Propellane

**DOI:** 10.1021/jacs.5c10732

**Published:** 2025-09-15

**Authors:** Jiandong Liu, Rajeshwaran Purushothaman, Fabian Hinrichs, Max Surke, Svenja Warratz, Lutz Ackermann

**Affiliations:** Wöhler Research Institute for Sustainable Chemistry (WISCh), 9375 Georg-August-Universität Göttingen, Tammannstraße 2, 37077 Göttingen, Germany

## Abstract

Over the past decade, bicyclo[1.1.1]­pentanes (BCPs) have emerged as valuable bioisosteres of aromatic rings, offering unique three-dimensional architectures for medicinal chemistry. Meanwhile, glycosyl derivatives play a pivotal role in chemical biology and drug discovery due to their widespread presence in biologically active molecules; however, the potential of bicyclo[1.1.1]­pentanes (BCPs) as versatile scaffolds in glycoscience remains largely unexplored. Herein, we report an electrochemistry strategy for the synthesis of BCP–glycosides via the functionalization of [1.1.1]­propellane. By leveraging an electrochemical halogen-atom transfer (*e*-XAT) process, we achieved a one-step, three-component reaction of glycosyl bromides, [1.1.1]­propellane, and radical acceptors under mild conditions, enabling the construction of glycosyl BCP–iodides, glycosyl BCP–H, and glycosyl BCP–pinacolboronic esters (Bpins) with exceptional functional group tolerance and scalability. Mechanistic studies suggested that the electrochemical process facilitated the generation of radical intermediates, which underwent selective addition to [1.1.1]­propellane, followed by trapping with radical acceptors. This study establishes a versatile platform for late-stage functionalization and streamlined access to privileged scaffolds in drug discovery and chemical biology.

## Graphical abstract

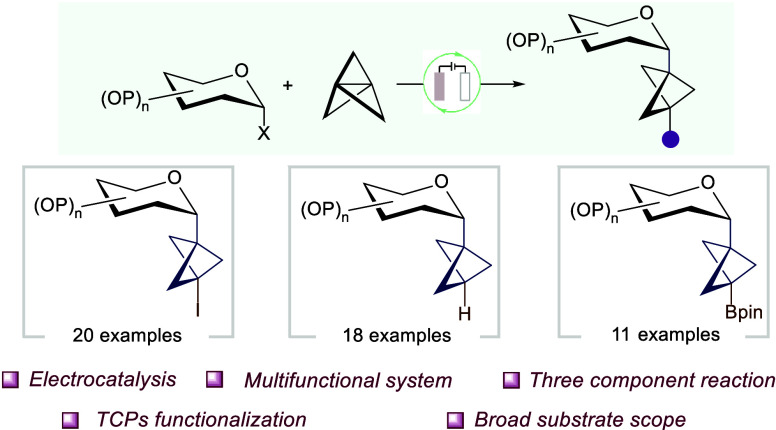

## Introduction

Benzenes, as one of the most prevalent structural motifs in commercially available small-molecule therapeutics, play a crucial role in drug design. However, they are often associated with suboptimal drug-like properties, such as metabolic instability and poor aqueous solubility.^1^ To overcome these challenges, recent advancements in medicinal chemistry have demonstrated that C­(sp^3^)-enriched bioisosteric frameworks possess superior physicochemical properties, offering greater metabolic stability and enhanced solubility compared to their aromatic counterparts (Figure 1A).^2^ Subsequently, bicyclo[1.1.1]­pentane (BCP) has garnered significant attention for its ability to serve as a para-substituted benzene surrogate, offering enhanced metabolic stability, improved solubility, and optimized pharmacokinetic properties in drug candidates.^3^ Following the successful synthesis of [1.1.1]­propellane by Wiberg and Walker,^4^ extensive efforts have been dedicated to its functionalization via radical and anionic pathways.^5–7^ In particular, recent years have witnessed significant momentum driven by the research groups of Knochel,^8^ Anderson,^9^ Aggarwal,^10^ Leonori,^11^ and MacMillan,^12^ among others,^13–17^ reflecting the outstanding potential of this unique scaffold.

**Figure 1:**
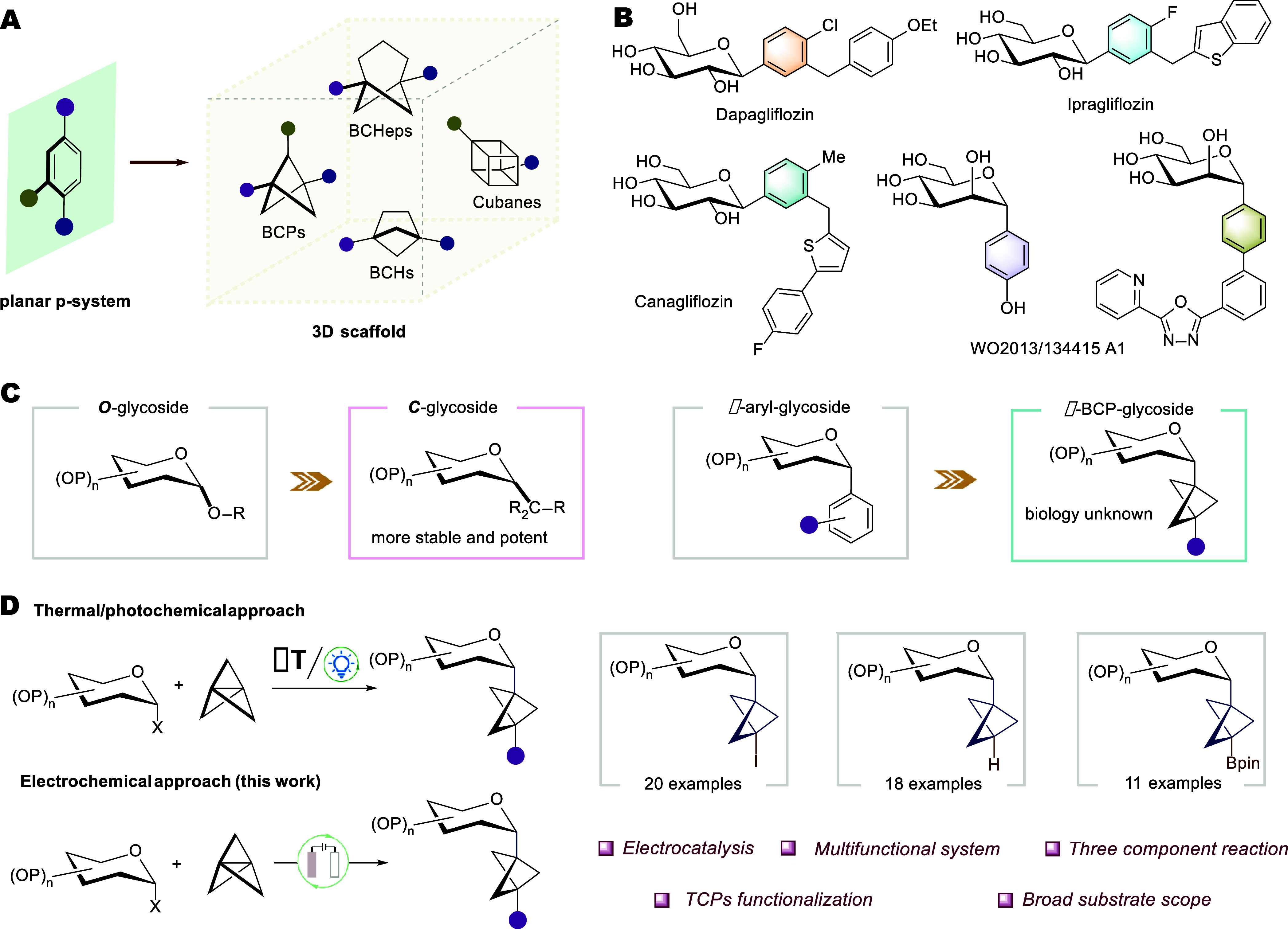
Electrochemical one-step multicomponent access to diverse α-glycosyl BCP derivatives. (A) Bioisosteres: transforming 2D benzenes into 3D molecular frameworks. (B) Drugs featuring aryl-*C*-glycoside. (C) Evolution from *O*-glycosides to *C*-glycosides to BCP–glycosides. (D) Electrochemical synthesis of diverse BCP glycosides.

*O*-Glycosides—a sugar moiety linked at its anomeric position to an aglycone through an oxygen atom—are prevalent in natural products and pharmaceuticals.^18^ However, *O*-glycosides are often susceptible to enzymatic hydrolysis, leading to limited metabolic stability and reduced therapeutic efficacy. To address these challenges, *C*-glycosides, in which the sugar is linked to the aglycone via a C–C bond, have emerged as stable alternatives.^19^ *C*-Glycosides exhibit enhanced resistance to enzymatic degradation and improved pharmacokinetic properties, making them attractive candidates for drug development (Figure 1B and 1C). Examples of *C*-glycoside-based drugs include the sodium-dependent glucose cotransporter 2 (SGLT2) inhibitor for the treatment of type II diabetes canagliflozin and dapagliflozin and the anticancer drug *C*-glycoside analogue of KRN7000, both of which highlight the therapeutic potential of this class of compounds.^20–22^ Representing a significant subclass of *C*-glycosides are α-aryl-*C*-glycosides, which are characterized by a direct C–C bond between the α-configured anomeric center (*C*1) of the pyranose/furanose ring and an aryl group. Their synthesis has attracted considerable interest due to their structural complexity and biological significance.^23–33^ Despite notable progress in recent years, most approaches rely on transition metal catalysis with inherent challenges, including the requirement for often toxic metals, sophisticated ligand structures, and limited substrate scope.

The incorporation of BCPs into *C*-glycosides, particularly α-BCP–glycosides, represents a promising strategy to expand the chemical space of glycoscience (Figure 1C, right). The distinctive three-dimensional structure of BCPs, combined with their bioisosteric properties, offers a unique opportunity to design glycosyl derivatives with enhanced stability, bioavailability, and biological activity. Despite these potential advantages, the synthesis of BCP–glycosides remains largely unexplored, with only sporadic examples, notably all by photochemical approaches, while electrochemistry strategies^34–43^ largely remain elusive (Figure 1D, left).^9,14^ Herein, we report on an electrochemistry strategy for the assembly of α-BCP–glycosides through the functionalization of [1.1.1]­propellane. By leveraging an electrochemical halogen-atom transfer (*e*-XAT) process, we achieved the direct coupling of glycosyl bromides with [1.1.1]­propellane and various radical acceptors—including γ-terpinene, *n*Bu_4_NI (tetrabutylammonium iodide), and B_2_pin_2_ (bis­(pinacolato)­diboron)—enabling the one-step construction of α-glycosyl BCP–H, −I, and −Bpin (Figure 1D). Our findings constitute an efficient electrochemical approach for the modular synthesis of BCP–glycosides, enabling access to functionalized BCPs with potential applications in drug discovery and chemical biology.

## Results and Discussion

### Electrochemical Synthesis of Glycosyl BCP–H

Our investigation initially focused on the electrochemical synthesis of glycosyl BCP–H by optimizing the reaction conditions using galactosyl bromide **1a** and [1.1.1]­propellane **2** as model substrates, commercially available and cost-efficient γ-terpinene as the H source, *n*Bu_4_NBF_4_ as the electrolyte, and diisopropylethylamine (DIPEA) as the *e*-XAT mediator and sacrificial anodic reagent. Based on preliminary experimentation, we conducted a series of control experiments and parameter optimizations to improve the efficiency of the electrochemistry strategy (Table 1). Changes to the applied current (entry 2) demonstrated that a constant current of 3.0 mA provided the optimal yield of 81%. The electrode material was found to significantly influence the reaction outcome. When platinum or glassy carbon (GC) was used as the cathode, only trace amounts of product were detected, whereas high yields were obtained with GF or RVC as the cathode materials (entries 5–7). These results suggest that both the cathode material and its specific surface area—which influences current density—play a key role in the efficiency of the electron transfer process. Similarly, replacing the anode with a sacrificial zinc electrode or platinum electrode led to poor performance, further emphasizing the importance of electrode material selection in guaranteeing effective electrochemical conditions (entries 3–4). In contrast, graphite felt (GF) proved ideal as the anode material. GF electrodes offer significant practical advantages, including low cost, exceptional durability, and nonsacrificial behavior. These attributes make GF electrodes particularly suitable for industrial-scale applications and facilitate large-scale implementation of the electrochemical process. Solvent effects were next examined (entry 8), revealing that acetonitrile (CH_3_CN) was the optimal reaction solvent. Removal of the electrical current (entry 9) completely suppressed product formation, unequivocally demonstrating the essential role of electrochemical activation in the reaction. Furthermore, when the reaction was conducted in the absence of DIPEA, a significantly diminished yield of merely 25% was obtained (entry 10). This highlights the critical role of DIPEA in the *e*-XAT process. Variation in the amount of γ-terpinene indicated that it influences the efficiency of the hydrogen atom transfer (HAT) process, as reflected in the reaction yield (entry 11). The choice of electrolyte was also examined, with alternative salts providing variable efficiency but ultimately underperforming compared to *n*Bu_4_NBF_4_ (entry 12). Temperature plays a crucial role for the reaction outcome (entry 13). While the reaction performed at −20 °C exhibited comparable efficacy, increasing the reaction temperature to room temperature led to slightly diminished yields (70% yield).

**Table 1:**
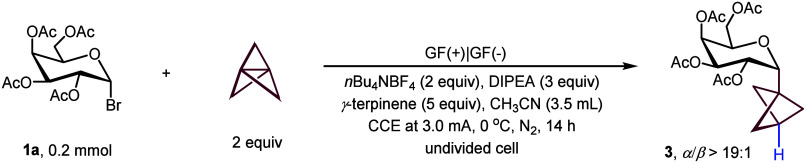
Optimization of the Reaction Parameters^a^

Entry	Deviation from standard conditions	Yield of **3** (%)^b^ ^,^ ^c^
1	none	86 (81)^d^
2	2.0 mA/4.0 mA/6.0 mA	80/83/75
3	Pt(+)/GF(−)	65
4	Zn(+)/GF(−)	41
5	GF(+)/Pt(−)	trace
6	GF(+)/GC(−)	trace
7	GF(+)/RVC(−)	84
8	DMF/THF	75/trace
9	*w/o* current	N.R.
10	*w/o* DIPEA	25
11	without/3 equiv/4 equiv γ-terpinene	56/78/82
12	*n*Bu_4_NClO_4_/*n*Bu_4_NPF_6_/LiClO_4_	76/80/55
13	–20 °C/r.t.	85/70

a Reaction conditions: **1** (0.2 mmol), [1.1.1]­propellane (0.4 mmol, Et_2_O/CH_2_(OEt)_2_ solution, 0.5–0.7 M), γ-terpinene (1.0 mmol), DIPEA (0.6 mmol), *n*Bu_4_NBF_4_ (0.4 mmol), CH_3_CN (3.5 mL) at 0 °C, 14 h under N_2_, GF as anode and cathode, constant current electrolysis (CCE) at 3.0 mA. Abbreviations: N.R., no reaction; DIPEA, *N*,*N*-diisopropylethylamine; THF, tetrahydrofuran; DMF, *N*,*N*-dimethylformamide.

b The ratio of α/β was determined by ^1^H NMR of the crude mixture.

c Determined by ^1^H NMR using 1,3,5-trimethoxybenzene as an internal standard.

d Isolated yield.

With the optimized electrochemistry conditions in hand (Table 1, entry 1), we evaluated the generality of the electrochemical glycosyl BCP–H synthesis (Figure 3). A diverse array of OAc-glycosyl bromides, including d-galactose, d-glucose, d-mannose, l-fucose, l-rhamnose, underwent efficient functionalization with [1.1.1]­propellane, selectively affording the desired glycosyl BCP–H products in moderate to excellent yields (**3**, **4**, **7**–**9**, 49%–82% yield). Glycosyl bromides with different *O*-groups, such as pivaloyl and benzoyl groups, were also amenable with 92% (**5**) and 34% yield (**6**), respectively. Notably, the reaction displayed exceptional diastereoselectivity, exhibiting exclusive or predominant formation of the α-isomer across all tested substrates, except for the five-membered d-ribofuranose **16**, which yielded the β-isomer (Figure 2A). Oligosaccharides are critical components of glycoconjugate vaccines, where bacterial capsular polysaccharides are conjugated to protein carriers (e.g., CRM197) to elicit T-cell dependent immune responses. Thus, the integration of BCP into oligosaccharide structures holds significant potential for enhancing the antigenic stability, conformational rigidity, and synthetic efficiency of glyco-conjugates. The successful assembly with a BCP unit into an oligosaccharide framework was achieved, including derivatives of maltotriose (**10**), maltose (**11**), lactose (**12**), cellobiose (**13**), melibiose (**14**) and isomaltose (**15**), highlighting the utility of this strategy for late-stage diversification. To demonstrate the broader applicability of our method, we extended it to a d-ribofuranosyl substrate. The reaction proceeded efficiently to afford compound **16** in 52% yield, highlighting the compatibility of our strategy with furanosides. Additionally, galactosyl bromides modified with natural products and drug derivatives were evaluated for direct late-stage modification (Figure 3B). Probenecid (**17**), diclofenac (**18**–**19**), and (*S*)-naproxen (**20**) were successfully incorporated into the glycosyl BCP framework, demonstrating the broad applicability of this approach for drug discovery and medicinal chemistry. Notably, the reaction displayed excellent α-stereoselectivity at the anomeric (C1) position, with >19:1 α/β ratio across all products. We further explored the compatibility with different protecting groups. For example, compound **16** was obtained in 52% yield from a ketal-protected d-ribofuranosyl bromide, demonstrating that such protecting groups are tolerated. In addition, glucosyl chloride with OBn groups showed no reactivity, likely due to the poor leaving ability of the C–Cl bond. Likewise, a mannosyl bromide bearing a ketal group gave low conversion (<10%). These findings highlight that protecting groups play a crucial role in modulating substrate reactivity and thus the overall reaction outcome.

**Figure 2:**
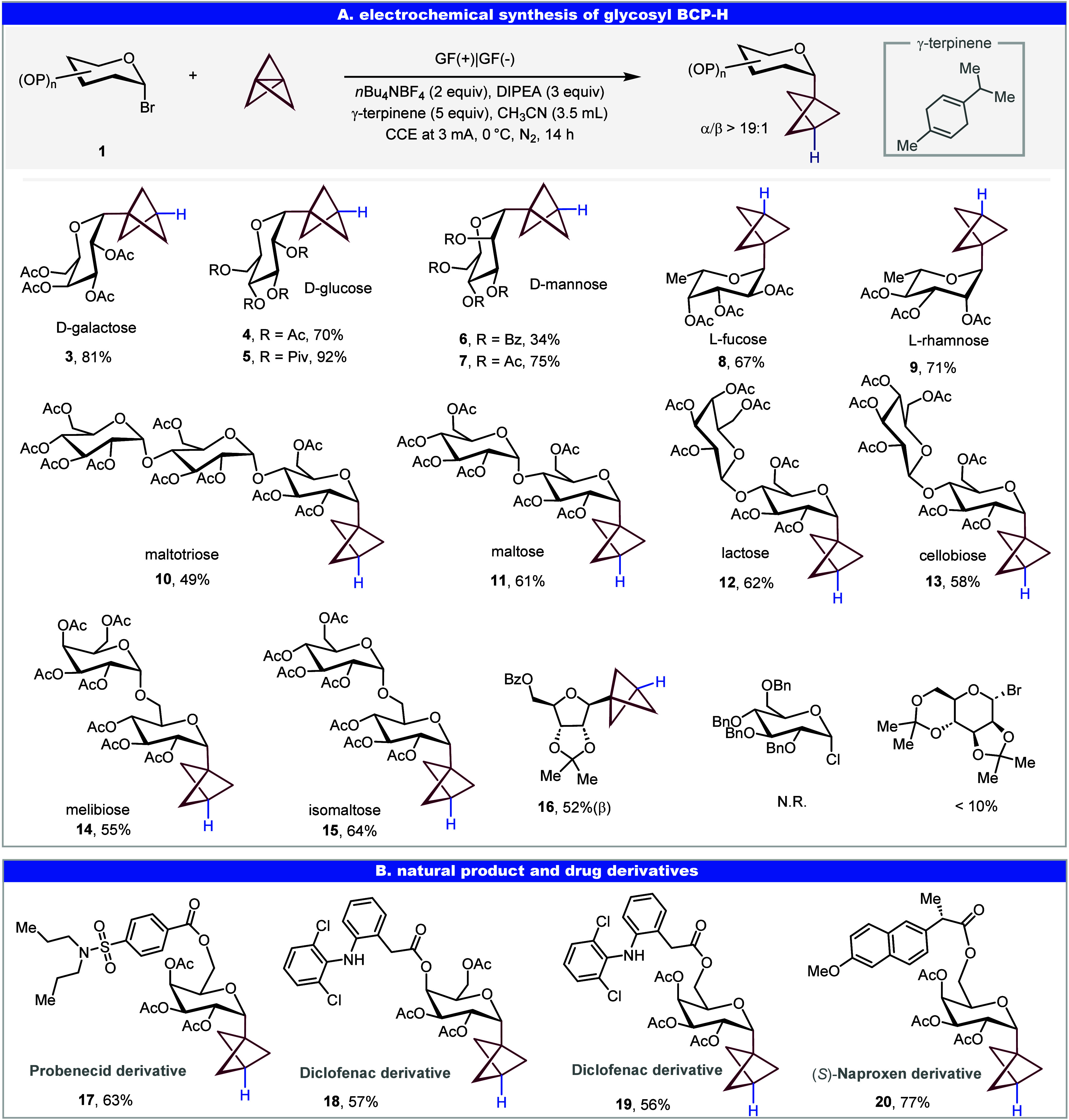
Electrochemistry strategy for *C*-glycoside-BCPs. Reaction conditions: **1** (0.2 mmol), [1.1.1]­propellane (0.4 mmol, Et_2_O/CH_2_(OEt)_2_ solution, 0.5–0.7 M), γ-terpinene (1.0 mmol), DIPEA (0.6 mmol), *n*Bu_4_NBF_4_ (0.4 mmol), CH_3_CN (3.5 mL) at 0 °C, 14 h under N_2_, GF as anode and cathode, CCE at 3.0 mA. Abbreviations: Ac, acetyl; Piv, pivaloyl; Bz, benzoyl; Me, methyl; DIPEA, *N*,*N*-diisopropylethylamine.

**Figure 3:**
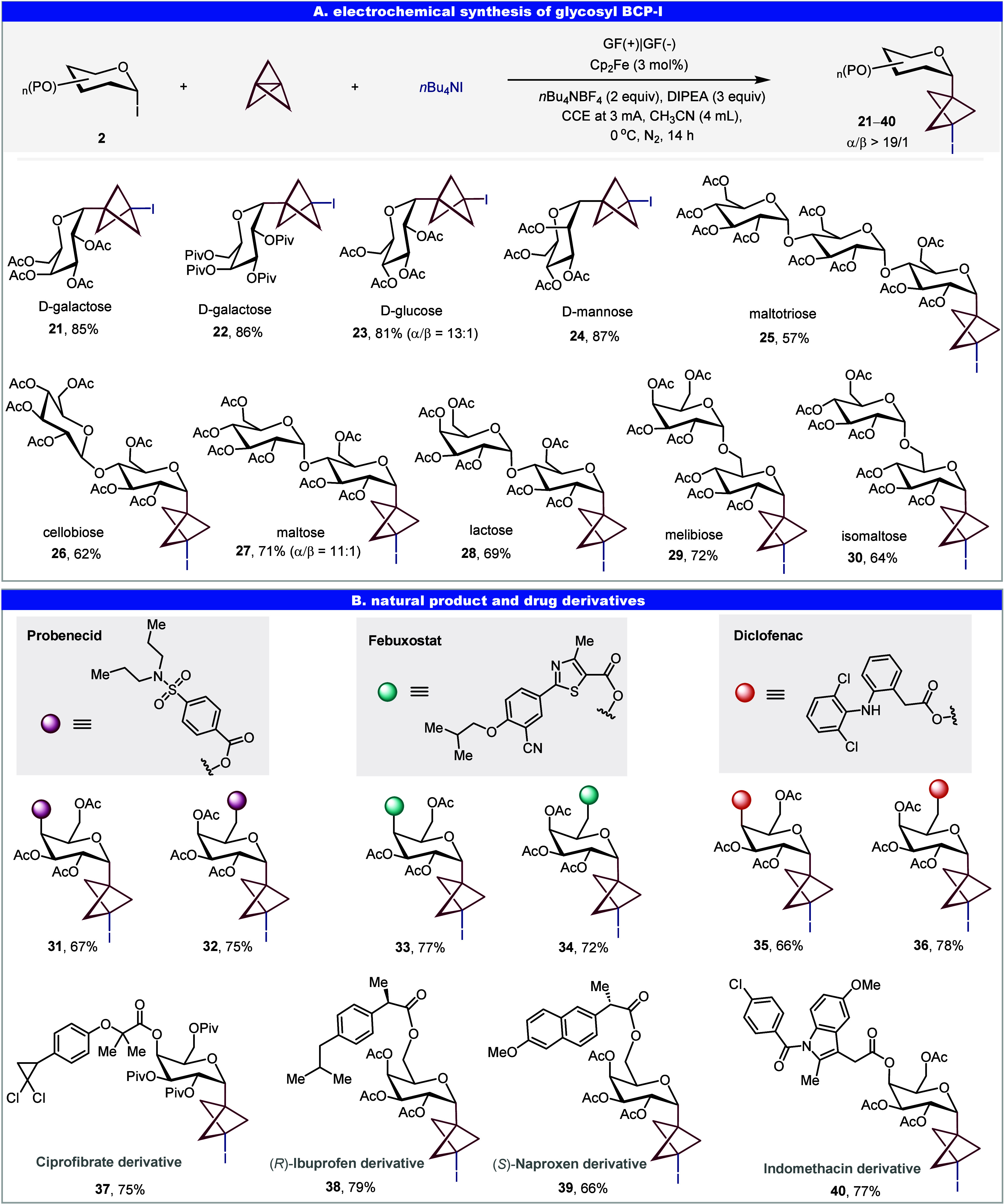
Electrochemistry strategy for *C*-glycoside-BCP–I. Reaction conditions: **2** (0.2 mmol), [1.1.1]­propellane (0.4 mmol, Et_2_O/CH_2_(OEt)_2_ solution, 0.5–0.7 M), *n*Bu_4_NI (0.4 mmol), DIPEA (0.6 mmol), *n*Bu_4_NBF_4_ (0.4 mmol), CH_3_CN (4.0 mL) at 0 °C, 14 h under N_2_, GF as anode and cathode, CCE at 3.0 mA. Abbreviations: Ac, acetyl; Piv, pivaloyl; Me, methyl; DIPEA, *N*,*N*-diisopropylethylamine.

With a successful synthetic route to glycosyl BCP–H in place, we next sought to develop a general strategy that facilitates the systematic construction of structurally diverse BCP-glycoside derivatives through the functionalization of a glycosyl BCP radical. However, the requirement for distinct reaction conditions along with extensive condition optimization for different reaction partners presents significant challenges. To address this limitation, we postulated that establishing straightforward synthetic routes to glycosyl BCP electrophiles and nucleophiles would enable rapid access to various BCP-glycoside derivatives through well-established coupling methodologies.

Consequently, we strategically targeted two highly versatile intermediates: glycosyl BCP–iodide as electrophilic coupling partners and glycosyl BCP–boronate as nucleophilic reagents. These intermediates were selected based on their exceptional reactivity profiles and broad compatibility with numerous cross-coupling conditions and functionalizations. By focusing on these valuable building blocks, we aimed to provide a platform that circumvents the need for bespoke reaction conditions for each substrate class, thereby significantly enhancing the versatility of BCP-glycoside synthesis for applications in medicinal chemistry and chemical biology.

### Electrochemical Synthesis of Glycosyl BCP–Iodide

We then turned our attention to glycosyl BCP electrophilic reagents. An example of glucosyl BCP–I synthesis under photochemical conditions has been reported.^9^ Herein, we developed a general and scalable electrochemical approach for the synthesis of glycosyl BCP–I derivatives. In the downstream iodination step, the glycosyl BCP radical reacts with tetrabutylammonium iodide (*n*Bu_4_NI), which serves as an inexpensive and readily available iodine source. Additionally, ferrocene was employed as a relay catalyst to facilitate the oxidative activation of iodide anions, enabling an efficient and scalable synthesis of glycosyl BCP–I derivatives. The electrochemistry strategy proved broadly applicable for a wide range of glycosyl donors and oligosaccharides, thus affording the corresponding glycosyl BCP–I products in moderate to good yields (**21**–**30**, 57–87% yield). Notably, protecting group variations on the carbohydrate backbone were well tolerated, demonstrating the robustness of this unifying electrochemistry strategy across diverse sugar scaffolds (**22**, 86% yield). Moreover, complex galactosyl-I bearing natural product or drug-derived functionalities at the C4 or C6 positions were successfully engaged in the reaction, underscoring the synthetic utility of this strategy for late-stage diversification and medicinal chemistry applications (**31**–**40**, 66–78% yield). The electrochemical reaction also exhibited excellent α-stereoselectivity at the anomeric (C1) position, with a ratio of >19:1 α/β observed across all products, except for **23** and **27**, which showed a slight decrease (13:1 and 11:1 α/β, respectively).

### Electrochemical Synthesis of Glycosyl BCP–Bpin

Boronic esters are widely utilized as transient functional groups in material sciences as well as pharmaceutical and agrochemical industries. BCP–boronates have emerged as a particularly significant subclass, owing to their exceptional capacity for rapid diversification into a broad range of BCP derivatives.^9,14,44,45^ Previous studies have demonstrated that, despite the intrinsic inertness of B_2_pin_2_ in radical alkyl borylation reactions,^46–49^ it can serve as an effective boron source in BCP radical borylation, particularly in the presence of coordinating additives.^14,17,50^ Notably, Molander and co-workers have reported the synthesis of a glucosyl BCP–Bpin compound using Bpin–SiMe_2_Ph as the boron source under photochemical conditions.^14^ Herein, leveraging our general electrocatalytic platform, we successfully adapted B_2_pin_2_ as a boron source to access a diverse array of glycosyl BCP–Bpins.

The substrate scope of this transformation was systematically investigated to assess its generality and functional group tolerance (Figure 4). A range of glycosyl BCP–Bpin, including d-glucose (**41**), d-mannose (**42**–**43**), d-galactose (**44**), l-rhamnose (**45**), underwent efficient electrochemical borylation, yielding the corresponding *C*-glycoside-BCP–Bpin in moderate to excellent yields. Notably, high α-stereoselectivity was observed, with the reaction displaying a strong preference for α-anomer formation. Moreover, the electrochemistry strategy proved highly effective for structurally complex oligosaccharides, underscoring its potential utility in glycochemistry and medicinal chemistry (**46**–**51**).

**Figure 4:**
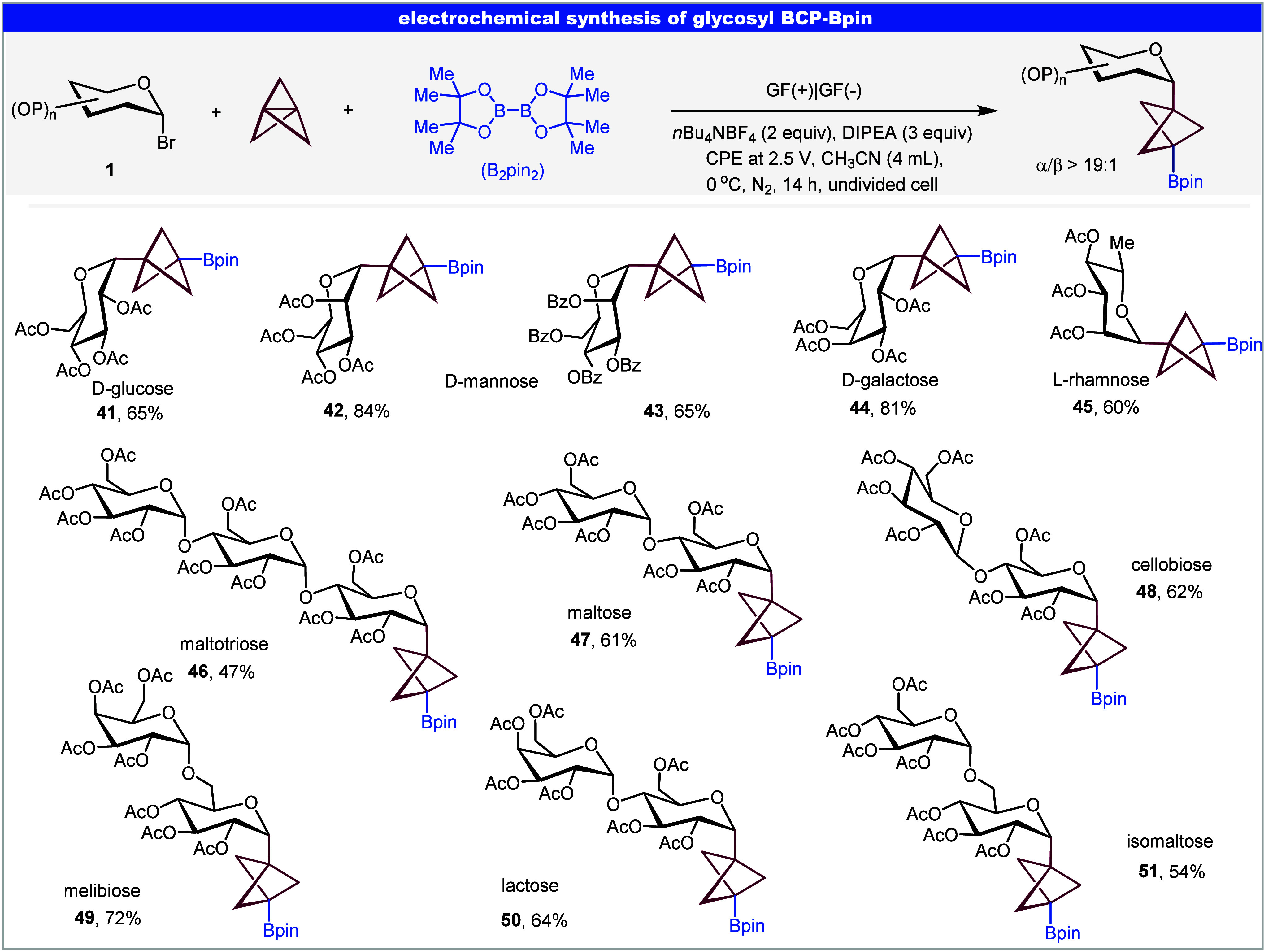
Electrochemistry strategy for *C*-glycoside-BCP–Bpin. Reaction conditions: **1** (0.2 mmol), [1.1.1]­propellane (0.4 mmol, Et_2_O/CH_2_(OEt)_2_ solution, 0.5–0.7 M), B_2_pin_2_ (0.6 mmol), DIPEA (0.6 mmol), *n*Bu_4_NBF_4_ (0.4 mmol), CH_3_CN (3.5 mL) at 0 °C, 14 h under N_2_, GF as anode and cathode, constant potential electrolysis (CPE) at 2.5 V. Abbreviations: Ac, acetyl; Bz, benzoyl; Me, methyl; DIPEA, *N*,*N*-Diisopropylethylamine.

To demonstrate the synthetic versatility of *C*-glycosyl BCP–I and BCP–Bpin, the gram-scale reaction and a series of derivatizations were carried out, yielding structurally diverse compounds (Figure 5A). On a 2 mmol scale, the electrochemical synthesis of glycosyl BCP–I afforded **21** in 95% yield (0.995 g). Likewise, glycosyl BCP–Bpin **41** was synthesized on a 3 mmol scale, delivering the product in 61% yield (0.963 g). These two bench-stable intermediates **21** and **41** served as valuable building blocks for late-stage functionalization. The oxidation of BCP–Bpin **41** led to the formation of the alcohol **52** (86% yield). BCP–Bpin **41** was smoothly transformed to the boronic acid **53** and the potassium trifluoroborate (BF_3_K) **54**, expanding the potential for downstream modifications. Arylation of glycosyl BCP–I **21** proved amenable, providing BCP–Ar **55** and **56**, as representative examples.^50,51^ Furthermore, amination of the glycosyl BCP–I provided BCP–NR_2_, with both an amide (**57**, 50% yield) and an indole (**58**, 51% yield) serving as effective nucleophiles.^52^ Overall, the ease of functionalization and broad synthetic utility of glycosyl BCP derivatives reinforce their potential as valuable building blocks in synthesis chemistry.

**Figure 5:**
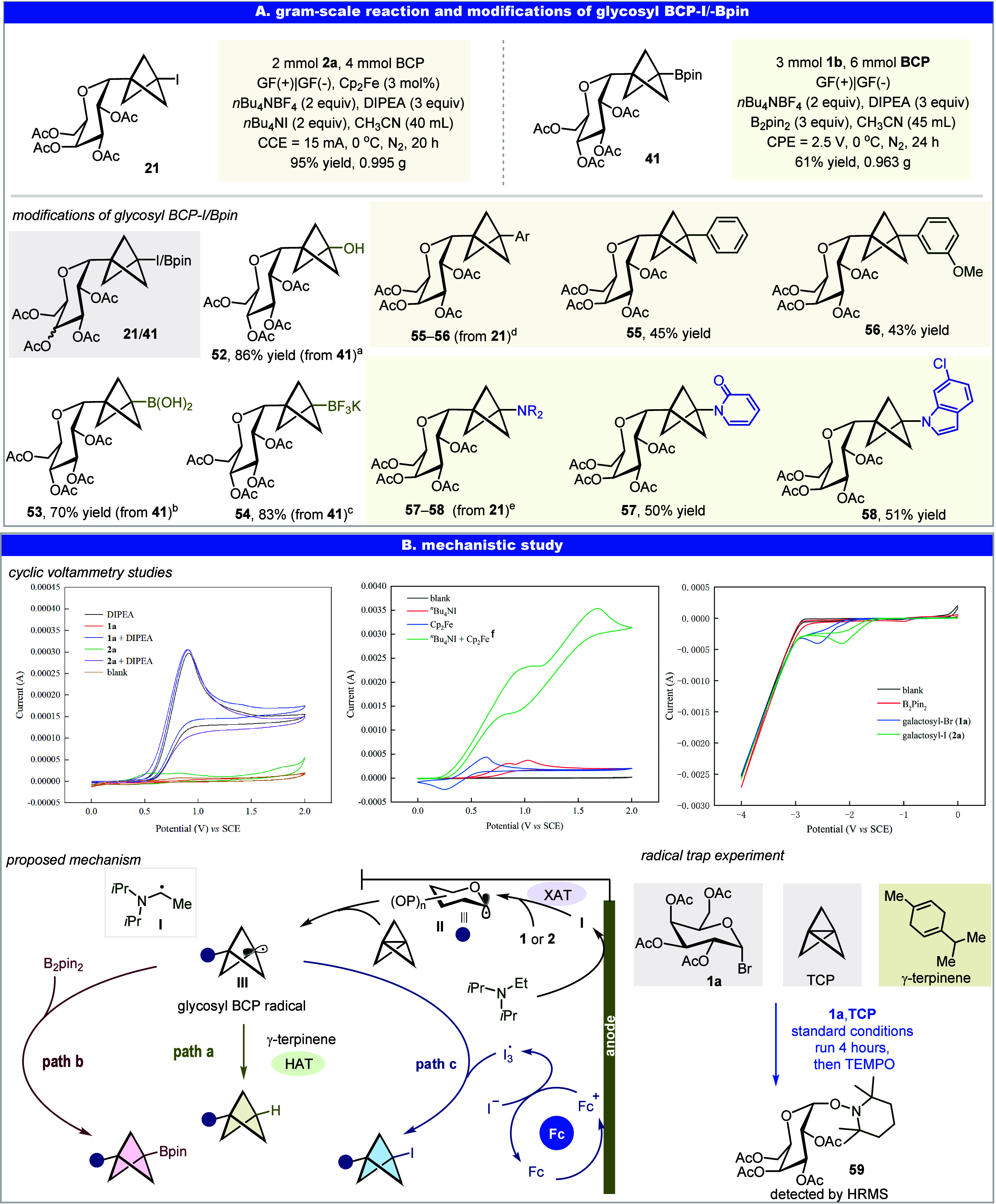
Application of glycosyl BCP–Bpin/I and mechanistic study. (A) Gram-scale reaction and applications of glycosyl BCP–I/Bpin. (B) Mechanistic studies. Cyclic voltammograms measured at 100 mV/s using CH_3_CN and *n*Bu_4_NBF_4_ (0.1 M) as the electrolyte, and all analytes were 20 mM. Legend: ^*a*^ NaBO_3_·4H_2_O, THF/H_2_O, rt, 2 h; ^*b*^ NaIO_4_, THF/H_2_O, rt, 17 h; ^*c*^ KHF_2_, MeOH, rt, 4 h; ^*d*^ Fe­(acac)_3_, *N*,*N*,*N*′,*N*′-tetramethylethylenediamine, THF, rt; ^*e*^ Cu­(TMHD)_2_, K_3_PO_4_, DMF, 100 °C, 16 h; ^*f*^ *n*Bu_4_NI (200 mM). Abbreviations: DMF, *N*,*N*-dimethylformamide; TMHD, 2,2,6,6-tetramethyl-3,5-heptanedionato; DIPEA, *N*,*N*-diisopropylethylamine; Cp_2_Fe and Fc, ferrocene; Ac, acetyl; TEMPO, 2,2,6,6-tetramethylpiperidin-1-yl)­oxy; Ar, aryl.

### Mechanistic Studies

We then turned our attention to elucidating the mechanism underlying this electrochemical transformation, aiming to gain insight into the nature of the radical intermediates formed under electrochemical conditions and the origin of the selectivity in this multicomponent reaction. To this end, we conducted a series of cyclic voltammetry (CV) experiments (Figure 5B). The CV studies revealed that the reduction peak of Fc^+^ completely disappears upon the addition of excess *n*Bu_4_NI, accompanied by a clear increase in catalytic current (Figure 5B, middle). This observation implies that the oxidized form of Fc^+^ is rapidly reduced back to its neutral state by iodide anions, indicating a fast and efficient electron transfer between Fc^+^ and I^–^. The observed catalytic current lends strong support to the rapid oxidation of iodide by Fc^+^, highlighting the critical role of ferrocene in promoting the formation of iodine radicals. In contrast, B_2_pin_2_ displays no observable reduction wave within the applied electrochemical window, indicating that it is not directly reduced at the cathode under these conditions (Figure 5B, right). We verified the involvement of radical species, through (2,2,6,6-tetramethylpiperidin-1-yl)­oxy (TEMPO) trapping experiments (Figure 5B, *radical trap experiment*). We successfully captured the radical precursors by halting the reaction at 4 h before adding TEMPO. This observation provides strong evidence for the involvement of glycosyl radicals. Based on our experimental findings and literature precedence, we propose a plausible mechanism for the electrochemical synthesis of glycosyl BCP dervatives.^30^ The reaction is initiated by anodic oxidation of the Hünig base (DIPEA), generating an α-amino alkyl radical (species **I**). The species **I** subsequently reacts with glycosyl halide and undergoes a XAT process to generate a glycosyl radical **II**, which exhibits intrinsic α-selectivity. In addition to the anodic pathway, cathodic reduction of the glycosyl halide may also contribute to the formation of the glycosyl radical (see Supporting Information, *Cathodic Process Study*, page S63). According to previous reports,^53^ the glucosyl and xylosyl radicals preferentially adopt B_2_,_5_- boatlike conformation, whereas the mannosyl and lyxosyl radicals favor ^4^C_1_-chairlike conformation. These pyranosyl radicals were maximally stabilized in these conformations because of the effective interaction between the radical orbital (SOMO) and the *p*-orbital of a lone pair of the ring oxygen in their periplanar arrangement. Following its formation, the α-glycosyl radical **II** readily reacts with [1.1.1]­propellane, leading to the formation of a α-glycosyl-BCP radical species **III**. By using different radical acceptors, we were able to furnish three different products: the neutral glycosyl BCP–H (path a), the nucleophilic glycosyl BCP–Bpin (path b), and the electrophilic glycosyl BCP–I (path c) (Figure 5B, *proposed mechanism*). The hydrogenation pathway, wherein the BCP radical undergoes a HAT process with γ-terpinene, has been studied under photochemical conditions.^17^ The borylation pathway has also been demonstrated by the Molander and Zhang groups under photochemical conditions.^14,17,50^ Notably, the high bond dissociation energy of the B–B bond in B_2_pin_2_ and its inertness toward alkyl radicals have rendered such transformations difficult under conventional conditions. Interestingly, despite these limitations, the glycosyl BCP radical successfully undergoes borylation with B_2_pin_2_ to afford glycosyl BCP–Bpin. Subsequent cyclic voltammetry and ^11^B NMR studies reveal that this reactivity likely stems from the *sp* ^2^-like electronic character of the BCP radical, which facilitates the reaction with B_2_pin_2_ (see Mechanistic studies part in Supporting Information).^14^ For the iodination pathway, we propose that iodide anions from *n*Bu_4_NI are oxidized at the anode by Fc^+^ to generate iodine radicals. The resulting iodine radical undergoes a radical–radical cross-coupling with the glycosyl BCP radical, yielding the glycosyl BCP–I.

## Conclusions

We have developed an electrochemistry strategy for the assembly of diverse glycosyl BCP derivatives through the functionalization of [1.1.1]­propellane. This approach, initiated by an electrochemically driven XAT process, provides access to glycosyl BCP–I, – H, and – Bpin under benign conditions, demonstrating exceptional functional group tolerance and broad substrate scope. Mechanistic investigations support the formation of α-glycosyl radical intermediates and provide a plausible reaction pathway. The electrochemitry platform enabled efficient late-stage modification of complex glycosyl substrates, including oligosaccharides, natural products and drug-derived derivatives. Given the increasing utility of BCPs as bioisosteres in medicinal chemistry, this strategy provides a concise and general approach for accessing functionalized glycosyl BCP derivatives, with broad potential in drug discovery and chemical biology.

## Supplementary Material



## Notes

Detailed experimental procedures and characterizations of new compounds. The Supporting Information is available free of charge at https://pubs.acs.org/doi/10.1021/jacs.5c10732.
