# Supplementary material for: Synthesis of Diverse Glycosyl Bicyclo[1.1.1]pentanes Enabled by Electrochemical Functionalization of [1.1.1]Propellane
Source: J Am Chem Soc. 2025 Sep 15;147(38):34813–22. doi: 10.1021/jacs.5c10732 (PMC12465002; doi:10.1021/jacs.5c10732)
Supplement: Supplementary file 1 [file ja5c10732_si_001.pdf]

Supporting Information for

Synthesis of diverse glycosyl bicyclo[1.1.1]pentanes enabled by electrochemical functionalization of [1.1.1]propellane

Jiandong Liu[1,2], Rajeshwaran Purushothaman[1,2], Fabian Hinrichs[1], Max Surke[1], Svenja Warratz[1], Lutz Ackermann[1,*]

[1]Wöhler Research Institute for Sustainable Chemistry, Georg-August-Universität Göttingen, Göttingen, Germany.

[2]These authors contributed equally

*Corresponding author. E-mail: Lutz.Ackermann@chemie.uni-goettingen.de

Table of Contents

Materials and methods..3

General procedure for the synthesis of glycosyl halides..............4

Optimization of reaction conditions 15

Table S1. Electrochemical synthesis of glycosyl BCP-H[a] 15

Table S2. Electrochemical synthesis of glycosyl BCP-I[a]..16

Table S3. Electrochemical synthesis of glycosyl BCP-Bpin[a] 16

General procedure for Electrochemical synthesis of diversely functionalized bicyclo[1.1.1]pentanyl glycosides........................18

I. General procedure for electrochemical synthesis of glycosyl BCP−H..............18

II. General procedure for electrochemical synthesis of glycosyl BCP−I..............18

III. General procedure for electrochemical synthesis of glycosyl BCP−Bpin 19

Full characterization of reaction products..................................21

Gram-scale reaction and modifications of glycosyl BCP-I/-Bpin 49

Gram-scale reaction of 21 49

Gram-scale reaction of 41 50

Modifications of glycosyl BCP−I (21) and glycosyl BCP−Bpin (41) 52

Mechanistic studies..58

Radical trap experiment..58

Cyclic Voltammetry studies **59**

Cathodic Process Study **63**

Copies of NMR spectra 66

References 163

Materials and methods

^1H and ^{13}C NMR spectra were recorded on a Bruker instrument (400 MHz, 500 MHz or 600 MHz) and internally referenced to tetramethylsilane signal or residual portion solvent signals. Data for ^1H NMR are recorded as follows: chemical shift (δ, ppm), multiplicity (s = singlet, d = doublet, t = triplet, m = multiplet or unresolved, br = broad singlet, coupling constant(s) in Hz, integration). Data for ^{13}C NMR are reported in terms of chemical shift (δ, ppm). All IR spectra were recorded on a Bruker FT-IR Alpha-P device. EI-MS spectra were recorded on Jeol AccuTOF at 70eV, ESI-MS spectra on Bruker MicrOTOF and maXis. Unless stated otherwise, all reactions were carried out in a Schlenk tube under an argon atmosphere. Platinum electrodes Pt 99,95%, 25x10x0.125 mm, electrical conductivity 9.43 MS/m were purchased from ESG Edelmetall-Handel GmbH & Co. KG, Rheinstetten, Germany. Graphite felt GFA6 were purchased from SGL Carbon GmbH, Meitlingen, Germany. All solvents are of HPLC grade and used directly without further purification. The substrates were either purchased directly from commercial suppliers or prepared according to previously reported procedures. BCP, $Et_2O/CH_2(OEt)_2$ solution, 0.5−0.7 M[23]. Chromatography was carried out on Merck silica gel 60 (40–63 μm)

General procedure for the synthesis of glycosyl halides

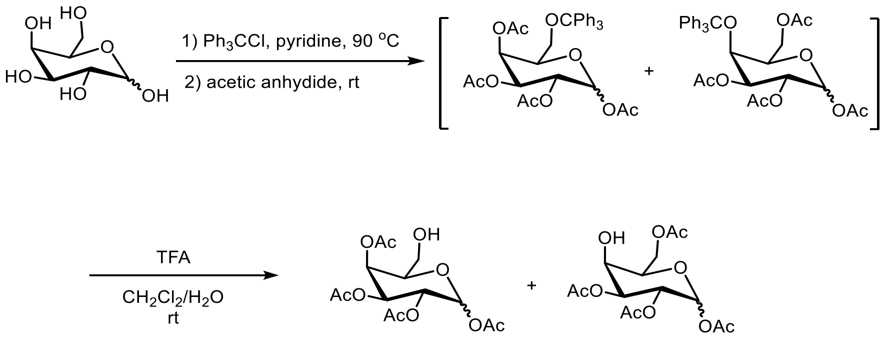

S1 **S2**

Trityl chloride (7.75 g, 27 mmol) was added into the solution of D-galactose (5.0 g, 27 mmol) in anhydrous pyridine (20 ml) under nitrogen flow. The mixture was stirred for 2 h at 90 °C. As the solution cooled down to room temperature, acetic anhydride (25 ml, 266 mmol) was added to the solution. The mixture was stirred for a further 20 h at room temperature. The solution was then poured into a mixture of ice-cold water (100 mL) and acetic acid (40 mL). The resulting mixture was stirred for 0.5 hours, followed by extraction with dichloromethane (50 mL × 3). The combined organic layers were successively washed with brine (100 mL × 3), then dried over $MgSO_4$. After filtration, the filtrate was concentrated under reduced pressure to afford the syrupy product, which was used directly in the next step without further purification.

The crude product from last step was dissolved in the mixture of dichloromethane (30 ml) and water (3 ml), followed by addition of trifluoroacetic acid (6.9 ml, 90 mmol). The mixture was stirred for 20 min at room temperature. The resulting mixture was diluted with dichloromethane (100 ml) then washed with water, saturated $NaHCO_3$ solution and brine. The mixture was dried with $MgSO_4$ then concentrated. The crude product was purified by flash column chromatography using a hexane/ethyl acetate (1:1, v/v) solvent system to afford the mixture of **S1** and **S2** as a syrupy. The crude products consisted of isomeric glycosides (deprotected at 4- and 6-positions) due to separation difficulties (observed as a single spot, R_f = 0.25 in n-hexane/EtOAc 1:1 by TLC analysis). The isomeric mixture was employed directly in subsequent transformations

without further purification.

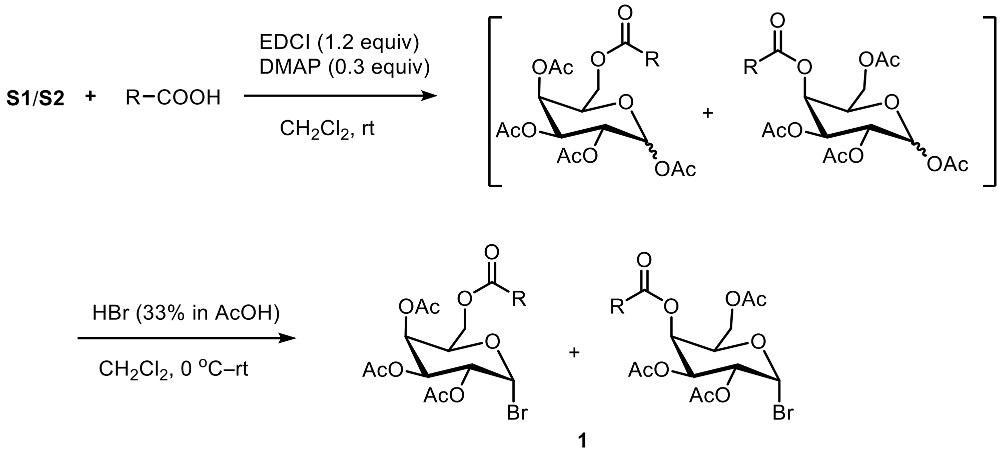

To a solution of **S1/S2** (3.48 g, 10 mmol, 1.00 equiv) in dry DCM (30 mL) were added carboxylic acid (11 mmol, 1.10 equiv), DMAP (366 mg, 3.0 mmol, 0.30 equiv), EDCI·HCl (4.60 g, 12 mmol, 1.20 equiv). After stirring at room temperature for 12 h, the reaction mixture was diluted with DCM and washed with saturated $NaHCO_3$ and brine successively. The organic phase was dried over Na_2SO_4, filtered, and concentrated *in vacuo*. The residue was purified by silica gel column chromatograph. The products still remained as a mixture due to the inability to separate these components, the mixture was carried forward directly to the subsequent bromination/iodination reaction without further purification.

The C-1 acetyl protected sugar from last step (2~5 mmol, 1.00 equiv) was dissolved in dry CH_2Cl_2 (0.500 M) and cooled to 0 °C. HBr (33% Wt in AcOH, 2.00 equiv) was added, and the reaction mixture was slowly warmed to room temp over 10 min. After stirring at room temperature for 3 h, the reaction mixture was poured onto an ice/water mixture. The organic phase was collected and the aqueous phase was extracted with CH_2Cl_2 twice. The combined organic layers were washed with satd. $NaHCO_3$, brine, dried over Mg_2SO_4, and filtered. The filtrate was concentrated under vacuum and the residue was purified by flash column chromatography on silica gel to afford the 4-position and 6-position natural product- or drug molecule-modified galactosyl

bromides, respectively. (Typically, on TLC plates using an ethyl acetate/petroleum ether eluent system, the 4-position modified galactosyl bromide runs above the 6-position modified one, the structure was determined by COSY and HMBC 2D NMR and can also be distinguished by the ^{1}H and ^{13}C NMR spectra of the OAc group at the 4- and 6-positions.)

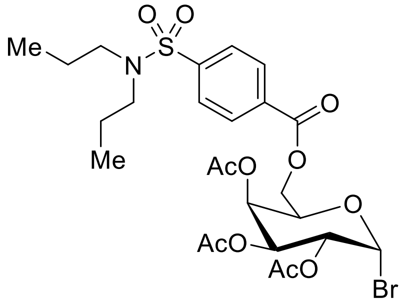

1n, foam, 0.357 g, 56% yield. ^{1}H NMR (400 MHz, $CDCl_3$) δ 8.13–8.10 (m, 2H), 7.90–7.87 (m, 2H), 6.73 (d, J = 4.0 Hz, 1H), 5.62 (dd, J = 3.3 Hz, J = 1.3 Hz, 1H), 5.45 (dd, J = 10.7 Hz, J = 3.3 Hz, 1H), 5.09 (dd, J = 10.7 Hz, J = 4.0 Hz, 1H), 4.66–4.62 (m, 1H), 4.48 (dd, J = 11.4 Hz, J = 6.7 Hz, 1H), 4.37 (dd, J = 11.4 Hz, J = 6.4 Hz, 1H), 3.12–3.08 (m, 4H), 2.18 (s, 3H), 2.12 (s, 3H), 2.02 (s, 3H), 1.60–1.51 (m, 4H), 0.88 (t, J = 7.4 Hz, 6H). ^{13}C NMR (100 MHz, $CDCl_3$) δ 170.1 (C_q), 170.0 (C_q), 169.9 (C_q), 164.6 (C_q), 144.7 (C_q), 132.5 (C_q), 130.5 (CH), 127.2 (CH), 88.2 (CH), 71.1 (CH), 68.0 (CH), 67.8 (CH), 67.1 (CH), 61.8 (CH_2), 50.1 (CH_2), 22.1 (CH_2), 20.8 (CH_3), 20.7 (CH_3), 11.2 (CH_3). HRMS (ESI) calcd for $C_{25}H_{34}BrNNaO_{11}S^+$ [M+Na]$^+$: 658.0928; Found: 658.0929.

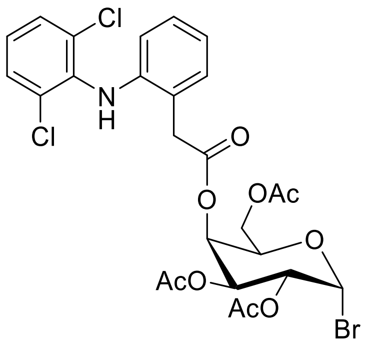

1o, foam, 0.275 g, 23% yield. ^{1}H NMR (300 MHz, $CDCl_3$) δ 7.34 (d, J = 8.1 Hz, 2H),

7.27 (dd, J = 7.5, 1.5 Hz, 1H), 7.12 (td, J = 7.7, 1.6 Hz, 1H), 6.98 (dd, J = 8.4, 7.8 Hz, 1H), 6.97 (td, J = 7.5, 1.4 Hz, 1H), 6.80 (s, 1H), 6.72 (d, J = 3.9 Hz, 1H), 6.55 (d, J = 8.0 Hz, 1H), 5.58 (dd, J = 3.4, 1.4 Hz, 1H), 5.39 (dd, J = 10.6, 3.4 Hz, 1H), 5.08 (dd, J = 10.6, 3.9 Hz, 1H), 4.54–4.46 (m, 1H), 4.18–4.10 (m, 2H), 4.02–3.79 (m, 2H), 2.10 (s, 3H), 1.99 (s, 3H), 1.70 (s, 3H). ^{13}C NMR (75 MHz, $CDCl_3$) δ 171.9 (C_q), 170.3 (C_q), 170.1 (C_q), 169.9 (C_q), 142.7 (C_q), 137.9 (C_q), 130.9 (CH), 129.6 (C_q), 129.0 (CH), 128.4 (CH), 124.3 (CH), 124.1 (C_q), 122.5 (CH), 118.7 (CH), 88.1 (CH), 71.0 (CH), 67.9 (CH), 67.8 (CH), 60.8 (CH_2), 38.0 (CH_2), 20.9 (CH_3), 20.7 (CH_3), 20.2 (CH_3). HRMS (ESI) calcd for $C_{26}H_{26}BrCl_2NaO_9^+$ [M+Na]$^+$: 668.0060; Found: 668.0061.

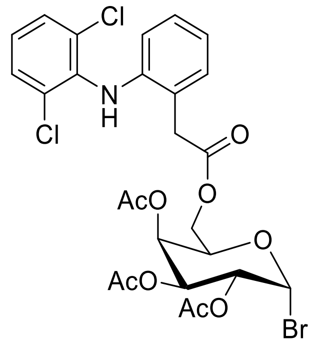

1p, foam, 0.443 g, 36% yield. ^{1}H NMR (300 MHz, $CDCl_3$) δ 7.34 (d, J = 8.1 Hz, 2H), 7.22 (dd, J = 7.5, 1.6 Hz, 1H), 7.12 (td, J = 7.7, 1.6 Hz, 1H), 6.98 (dd, J = 8.0, 7.7 Hz, 1H), 6.96 (td, J = 7.3, 1.2 Hz, 1H), 6.73 (s, 1H), 6.68 (d, J = 4.0 Hz, 1H), 6.54 (d, J = 8.0 Hz, 1H), 5.54 (dd, J = 3.3, 1.3 Hz, 1H), 5.41 (dd, J = 10.6, 3.3 Hz, 1H), 5.04 (dd, J = 10.6, 3.9 Hz, 1H), 4.53 (t, J = 6.8 Hz, 1H), 4.30–4.15 (m, 2H), 3.82 (s, 2H), 2.13 (s, 3H), 2.11 (s, 3H), 2.01 (s, 3H). ^{13}C NMR (75 MHz, $CDCl_3$) δ 171.9 (C_q), 170.2 (C_q), 170.0 (C_q), 169.8 (C_q), 142.8 (C_q), 137.9 (C_q), 131.1 (CH), 129.6 (C_q), 129.0 (CH), 128.3 (CH), 124.2 (CH), 124.0 (C_q), 122.3 (CH), 118.5 (CH), 88.1 (CH), 71.2 (CH), 68.0 (CH), 67.9 (CH), 67.1 (CH), 61.7 (CH_2), 38.2 (CH_2), 20.9 (CH_3), 20.7 (CH_3), 20.7 (CH_3). IR (ATR): ν_{max} (cm^{-1}) = 3332, 3069, 3029, 2964, 1745, 1452, 1370, 1212, 1076, 908, 728, 542, 470. HRMS (ESI) calcd for $C_{26}H_{26}BrCl_2NaO_9^+$ [M+Na]$^+$: 668.0060; Found: 668.0076.

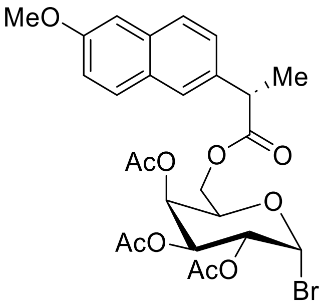

1q, foam, 0.431 g, 34% yield. ^{1}H NMR (300 MHz, $CDCl_3$) δ 7.76–7.58 (m, 3H), 7.37 (dd, J = 8.5, 1.9 Hz, 1H), 7.20–7.02 (m, 2H), 6.65 (d, J = 4.0 Hz, 1H), 5.50 (dd, J = 3.3, 1.3 Hz, 1H), 5.37 (dd, J = 10.7, 3.3 Hz, 1H), 5.02 (dd, J = 10.6, 3.9 Hz, 1H), 4.44 (t, J = 6.7 Hz, 1H), 4.26 (dd, J = 11.3, 6.5 Hz, 1H), 4.06 (dd, J = 11.3, 6.9 Hz, 1H), 3.91 (s, 3H), 3.85 (q, J = 7.3 Hz, 1H), 2.13 (s, 3H), 2.10 (s, 3H), 2.01 (s, 3H), 1.56 (d, J = 7.2 Hz, 3H). ^{13}C NMR (75 MHz, $CDCl_3$) δ 174.1 (C_q), 170.1 (C_q), 170.0 (C_q), 169.8 (C_q), 157.8 (C_q), 135.1 (C_q), 133.8 (C_q), 129.4 (CH), 129.0 (C_q), 127.4 (CH), 126.2 (CH), 126.1 (CH), 119.1 (CH), 105.7 (CH), 88.1 (CH), 71.2 (CH), 68.0 (CH), 67.9 (CH), 67.0 (CH), 60.9 (CH_2), 55.4 (CH_3), 45.2 (CH), 20.8 (CH_3), 20.7 (CH_3), 20.6 (CH_3), 18.5 (CH_3). IR (ATR): ν_{max} (cm^{-1}) = 2959, 2908, 1744, 1606, 1370, 1213, 1075, 1030, 912, 730, 543, 471. HRMS (ESI) calcd for $C_{26}H_{29}BrNaO_{10}^{+}$ $[M+Na]^{+}$: 603.0836; Found: 603.0846.

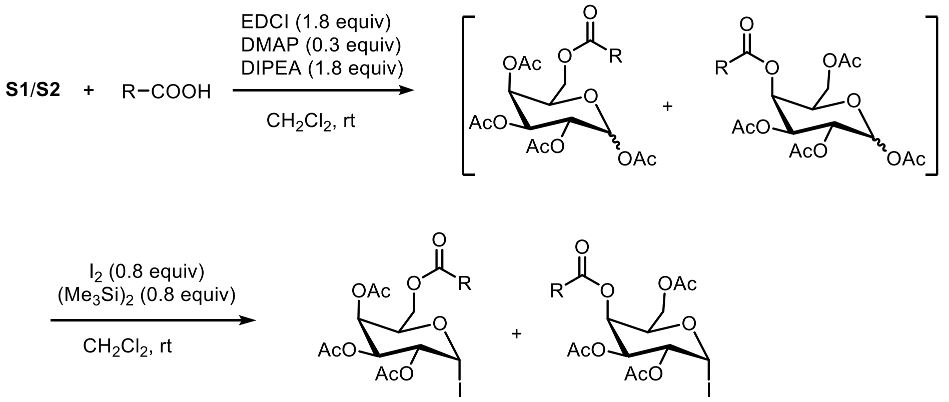

To a solution of **S1**/**S2** (3.48 g, 10 mmol, 1.00 equiv) in dry DCM (30 mL) were added carboxylic acid (11 mmol, 1.10 equiv), DMAP (366 mg, 3.0 mmol, 0.30 equiv),

EDCI·HCl (4.60 g, 12 mmol, 1.20 equiv). After stirring at room temperature for 12 h, the reaction mixture was diluted with DCM and washed with saturated $NaHCO_3$ and brine successively. The organic phase was dried over Na_2SO_4, filtered, and concentrated *in vacuo*. The residue was purified by silica gel column chromatograph. The products still remained as a mixture due to the inability to separate these components, the mixture was carried forward directly to the subsequent bromination/iodination reaction without further purification.

To a solution of mixture of the C1 acetyl protected sugar from last step (3 mmol, 1.00 equiv) in dry CH_2Cl_2 (0.400 M), I_2 (0.800 equiv) and $(Me_3Si)_2$ (0.800 equiv) were added and the reaction mixture was stirred at room temperature for 3-12 h. The resulting mixture was diluted with $CHCl_3$ and washed with 1:1 (v/v) saturated $NaHCO_3$:10% $Na_2S_2O_3$ and saturated $NaHCO_3$ twice. The combined aqueous layers were extracted with $CHCl_3$. The combined organic layers were washed with brine, dried over anhydrous Mg_2SO_4, and filtered. The filtrate was concentrated *in vacuo*. The residue was purified by flash column chromatography to afford the 4-position and 6-position natural product- or drug molecule-modified galactosyl iodides, respectively. (Typically, on TLC plates using an ethyl acetate/petroleum ether eluent system, the 4-position modified galactosyl bromide runs above the 6-position modified one, the structure was determined by COSY and HMBC 2D NMR)

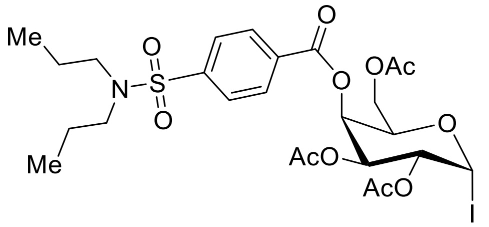

2k, foam, 0.80 g, 39% yield. ^{1}H NMR (300 MHz, $CDCl_3$) δ 8.13 (d, *J* = 8.0 Hz, 2H), 7.87 (d, *J* = 8.1 Hz, 2H), 7.13 (d, *J* = 4.2 Hz, 1H), 5.74 (s, 1H), 5.36 (dd, *J* = 10.6, 3.3 Hz, 1H), 4.45–4.30 (m, 2H), 4.21–4.15 (m, 2H), 3.06 (t, *J* = 7.7 Hz, 4H), 2.07 (s, 3H), 1.98 (s, 3H), 1.93 (s, 3H), 1.59–1.46 (m, 4H), 0.84 (t, *J* = 7.3 Hz, 6H). ^{13}C NMR (75

MHz, $CDCl_3$) δ 170.2 (C_q), 169.8, (C_q) 169.7 (C_q), 164.2 (C_q), 145.0 (CH), 131.9 (CH), 130.5 (C_q), 127.3 (C_q), 74.8 (CH), 73.8 (CH), 69.7 (CH), 67.9 (CH), 67.6 (CH), 60.9 (CH_2), 50.1 (CH_2), 22.1 (CH_2), 20.9 (CH_3), 20.6 (CH_3), 11.2 (CH_3). HRMS (ESI) calcd for $C_{25}H_{34}INNaO_{11}S^+$ $[M+Na]^+$: 706.0789; Found: 706.0798.

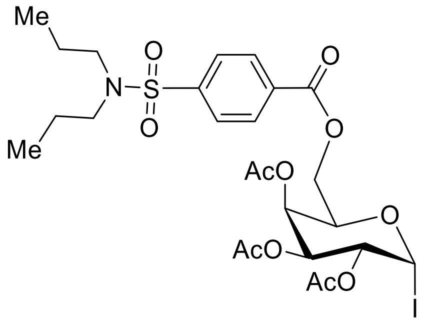

2l, foam, 0.60 g, 29% yield. ^{1}H NMR (300 MHz, $CDCl_3$) δ 8.08 (d, J = 8.2 Hz, 2H), 7.84 (d, J = 8.1 Hz, 2H), 7.08 (d, J = 4.2 Hz, 1H), 5.55 (s, 1H), 5.29 (dd, J = 10.6, 3.3 Hz, 1H), 4.50–4.30 (m, 4H), 3.05 (t, J = 7.7 Hz, 4H), 2.14 (s, 3H), 2.08 (s, 3H), 1.98 (s, 3H), 1.58–1.44 (m, 4H), 0.83 (t, J = 7.3 Hz, 6H). ^{13}C NMR (75 MHz, $CDCl_3$) δ 169.9 (C_q), 169.8 (C_q), 169.7 (C_q), 164.5 (C_q), 144.7 (CH), 132.5 (CH), 130.4 (C_q), 127.1 (C_q), 75.2 (CH), 73.6 (CH), 69.7 (CH), 67.5 (CH), 66.6 (CH), 61.7 (CH_2), 50.0 (CH_2), 22.0 (CH_2), 20.9 (CH_3), 20.6 (CH_3), 20.6 (CH_3), 11.2 (CH_3). HRMS (ESI) calcd for $C_{25}H_{34}INNaO_{11}S^+$ $[M+Na]^+$: 706.0789; Found: 706.0784.

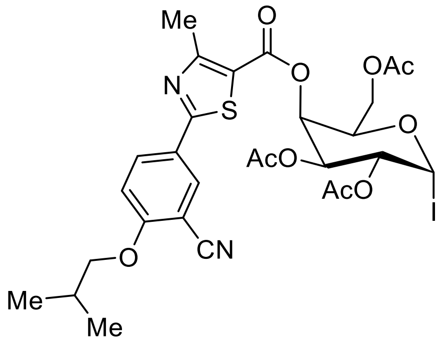

2m, foam, 0.33 g, 15% yield. ^{1}H NMR (300 MHz, $CDCl_3$) δ 8.16–8.09 (m, 1H), 8.07–7.98 (m, 1H), 7.13–7.07 (m, 1H), 6.97 (d, J = 9.0 Hz, 1H), 5.63 (s, 1H), 5.29 (dd, J = 10.5, 3.4 Hz, 1H), 4.38–4.26 (m, 2H), 4.23–4.10 (m, 2H), 3.84 (d, J = 6.5 Hz, 2H), 2.68 (s, 3H), 2.19–2.06 (m, 1H), 2.04 (s, 3H), 1.97 (s, 3H), 1.94 (s, 3H), 1.01 (d, J = 6.7 Hz,

6H). ^{13}C NMR (75 MHz, $CDCl_3$) δ 170.1, 169.6, 168.2, 162.6, 160.7, 132.7, 132.0, 125.5, 119.8, 115.1, 112.6, 102.9, 75.6, 74.9, 73.6, 69.7, 67.5, 67.5, 60.8, 28.0, 20.8, 20.5, 20.5, 18.9, 17.5. HRMS (ESI) calcd for $C_{28}H_{31}IN_2NaO_{10}S^+$ $[M+Na]^+$: 737.0636; Found: 737.0626.

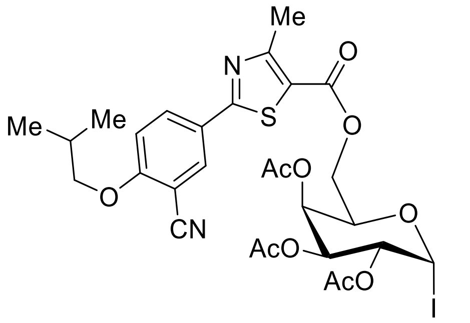

2n, foam, 0.31 g, 14% yield. ^{1}H NMR (300 MHz, $CDCl_3$) δ 8.15 (t, *J* = 2.9 Hz, 1H), 8.05 (d, *J* = 8.9 Hz, 1H), 7.09 (d, *J* = 4.2 Hz, 1H), 6.99 (d, *J* = 9.2 Hz, 1H), 5.53 (d, *J* = 3.3 Hz, 1H), 5.31 (dd, *J* = 10.6, 2.8 Hz, 1H), 4.48–4.15 (m, 4H), 3.88 (d, *J* = 6.4 Hz, 2H), 2.72 (s, 3H), 2.25–2.16 (m, 1H), 2.16 (s, 3H), 2.10 (s, 3H), 2.00 (s, 3H), 1.07 (d, *J* = 6.7 Hz, 6H). ^{13}C NMR (75 MHz, $CDCl_3$) δ 169.9, 169.8, 168.0, 162.6, 162.3, 161.2, 132.8, 132.2, 125.9, 120.7, 115.4, 112.7, 103.0, 75.7, 75.1, 73.7, 69.7, 67.6, 66.7, 61.5, 28.2, 21.0, 20.6, 19.1, 17.7. HRMS (ESI) calcd for $C_{28}H_{31}IN_2NaO_{10}S^+$ $[M+Na]^+$: 737.0636; Found: 737.0628.

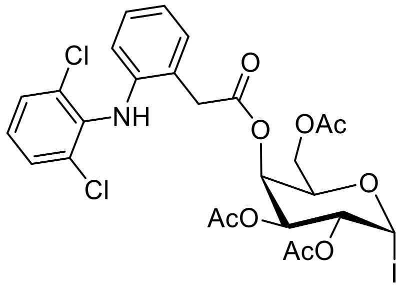

2o, foam, 0.30 g, 14% yield. ^{1}H NMR (300 MHz, $CDCl_3$) δ 7.34 (d, *J* = 8.0 Hz, 2H), 7.29–7.25 (m, 1H), 7.16–7.04 (m, 2H), 7.04–6.91 (m, 2H), 6.79 (s, 1H), 6.54 (d, *J* = 8.0 Hz, 1H), 5.55 (d, *J* = 3.1 Hz, 1H), 5.28 (dd, *J* = 10.5, 3.4 Hz, 1H), 4.39 (dd, *J* = 10.5, 4.2 Hz, 1H), 4.26 (t, *J* = 6.4 Hz, 1H), 4.19–4.08 (m, 2H), 3.99–3.81 (m, 2H), 2.10 (s, 3H), 1.99 (s, 3H), 1.70 (s, 3H). ^{13}C NMR (75 MHz, $CDCl_3$) δ 171.9 (C_q), 170.3 (C_q),

169.9 (C_q), 169.8 (C_q), 142.7 (C_q), 137.9 (C_q), 130.9 (C_q), 129.5 (CH), 129.0 (C_q), 128.4 (CH), 124.3 (CH), 124.1 (CH), 122.5 (C_q), 118.7 (CH), 75.1 (CH), 73.6 (CH), 69.6 (CH), 67.6 (CH), 67.3 (CH), 60.6 (CH), 38.0 (CH_2), 21.0 (CH_2), 20.7 (CH_3), 20.2 (CH_3). HRMS (ESI) calcd for $C_{26}H_{26}Cl_2INaNO_9^+$ $[M+Na]^+$: 715.9922; Found: 715.9913.

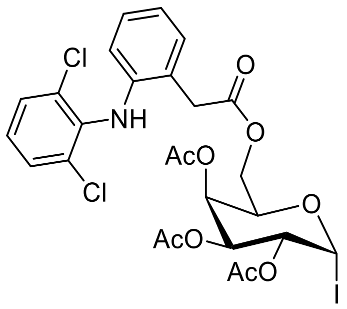

2p, foam, 0.40 g, 19% yield. ^{1}H NMR (300 MHz, $CDCl_3$) δ 7.33 (d, J = 8.0 Hz, 2H), 7.21 (d, J = 7.5 Hz, 1H), 7.12 (t, J = 7.5 Hz, 1H), 7.06 (d, J = 4.3 Hz, 1H), 7.03–6.90 (m, 2H), 6.72 (s, 1H), 6.54 (d, J = 8.0 Hz, 1H), 5.52 (d, J = 3.3 Hz, 1H), 5.29 (dd, J = 10.5, 3.3 Hz, 1H), 4.41–4.07 (m, 4H), 3.81 (s, 2H), 2.13 (s, 3H), 2.10 (s, 3H), 2.00 (s, 3H). ^{13}C NMR (75 MHz, $CDCl_3$) δ 171.8 (C_q), 169.90 (C_q), 169.88 (C_q), 169.7 (C_q), 142.7 (C_q), 137.9 (C_q), 131.0 (CH), 129.6 (C_q), 128.9 (CH), 128.2 (CH), 124.1 (CH), 123.9 (C_q), 122.3 (CH), 118.5 (CH), 75.0 (CH), 73.7 (CH), 69.7 (CH), 67.6 (CH), 66.7 (CH), 61.5 (CH_2), 38.1 (CH_2), 21.0 (CH_3), 20.6 (CH_3). HRMS (ESI) calcd for $C_{26}H_{26}Cl_2INaNO_9^+$ $[M+Na]^+$: 715.9922; Found: 715.9913.

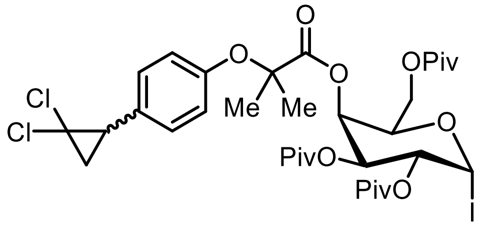

2q, foam, 1.51 g, 72% yield [from2R,3R,4S,5S,6R)-5-hydroxy-6-((pivaloyloxy)methyl)tetrahydro-2H-pyran-2,3,4-triyl tris(2,2-dimethylpropanoate)]. ^{1}H NMR (300 MHz, $CDCl_3$) δ 7.11 (d, J = 8.8 Hz, 2H), 7.01 (dd, J = 4.3, 2.7 Hz, 1H), 6.92–6.85 (m, 2H), 5.56 (d, J = 3.0 Hz, 1H), 5.40 (dd, J = 10.6, 3.1 Hz, 1H), 4.38–4.29 (m, 1H), 4.27–4.20 (m, 1H), 3.98–3.80 (m, 2H), 2.88–2.77 (m, 1H), 1.97–1.88 (m, 1H),

1.81–1.73 (m, 1H), 1.66 (s, 3H), 1.65 (s, 3H), 1.20 (s, 9H), 1.19 (s, 9H), 1.15 (d, *J* = 1.3 Hz, 9H). ^{13}C NMR (75 MHz, $CDCl_3$) δ 177.8 (C_q), 177.22 (C_q), 177.17 (C_q), 172.9 (C_q), 154.7 (C_q), *130.0 (CH), 129.9 (CH) (two signals due to diastereomers)*, *128.99 (C_q), 128.96 (C_q) (two signals due to diastereomers), 119.57 (CH), 119.47 (CH) (two signals due to diastereomers),* 79.47 *(C_q)*, 79.45 *(C_q) (two signals due to diastereomers)*, *75.38 (CH), 75.36 (CH) (two signals due to diastereomers, 74.18 (CH), 74.16 (CH) (two signals due to diastereomers,* 70.4 (CH), 68.1 (CH), 67.4 (CH), *60.92 (C_q), 60.90 (C_q) (two signals due to diastereomers),* 60.5 *(CH)*, 39.0 (C_q), 38.9 (C_q), 38.8 (C_q), 34.9 (CH), 27.30 (CH_3), 27.27 (CH_3), 27.25 (CH_3), *26.2 (CH_3), 26.0 (CH_3) (two signals due to diastereomers)*, *26.0 (CH_2)*, *25.9 (CH_2) (two signals due to diastereomers)*, *25.7 (CH_3)*, *25.5 (CH_3) (two signals due to diastereomers)*. HRMS (ESI) calcd for $C_{34}H_{47}ICl_2NaO_{10}^+$ $[M+Na]^+$: 835.1483; Found: 835.1479.

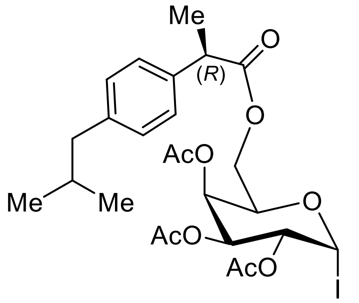

2r, foam, 0.40 g, 22% yield. ^{1}H NMR (300 MHz, $CDCl_3$) δ 7.16 (d, *J* = 8.1 Hz, 2H), 7.12–7.00 (m, 3H), 5.45 (d, *J* = 3.3 Hz, 1H), 5.25 (dd, *J* = 10.6, 3.3 Hz, 1H), 4.32 (dd, *J* = 10.5, 4.2 Hz, 1H), 4.29–4.15 (m, 2H), 4.05 (dd, *J* = 10.1, 5.9 Hz, 1H), 3.67 (q, *J* = 7.1 Hz, 1H), 2.43 (d, *J* = 7.2 Hz, 2H), 2.11 (s, 3H), 2.08 (s, 3H), 1.98 (s, 3H), 1.45 (d, *J* = 7.2 Hz, 3H), 0.89 (s, 3H), 0.87 (s, 3H). ^{13}C NMR (75 MHz, $CDCl_3$) δ 174.0 (C_q), 169.84 (C_q), 169.8 (C_q), 169.6 (C_q), 140.7 (C_q), 137.1 (C_q), 129.4 (CH), 127.2 (CH), 75.2 (CH), 73.7 (CH), 69.7 (CH), 67.5 (CH), 66.5 (CH), 60.6 (CH_2), 45.0 (CH_2), 44.8 (CH), 30.2 (CH), 22.44 (CH_3), 22.43 (CH_3), 20.9 (CH_3), 20.6 (CH_3), 20.5 (CH_3), 18.4 (CH_3). HRMS (ESI) calcd for $C_{25}H_{33}INaO_9^+$ $[M+Na]^+$: 627.1061; Found: 627.1056.

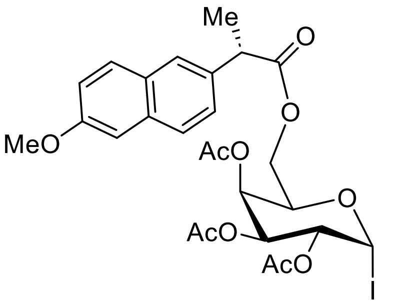

2s, foam, 0.38 g, 20% yield. ^{1}H NMR (300 MHz, $CDCl_3$) δ 7.73–7.64 (m, 3H), 7.38 (dd, *J* = 8.5, 1.9 Hz, 1H), 7.17–7.09 (m, 2H), 7.02 (d, *J* = 4.2 Hz, 1H), 5.47 (d, *J* = 3.4 Hz, 1H), 5.26 (dd, *J* = 10.6, 3.3 Hz, 1H), 4.33 (dd, *J* = 10.5, 4.2 Hz, 1H), 4.27 (dd, *J* = 10.5, 6.1 Hz, 1H), 4.23–4.16 (m, 1H), 4.07 (dd, *J* = 10.5, 6.3 Hz, 1H), 3.91 (s, 3H), 3.85 (q, *J* = 7.2 Hz, 1H), 2.13 (s, 3H), 2.10 (s, 3H), 2.01 (s, 3H), 1.56 (d, *J* = 7.2 Hz, 3H). ^{13}C NMR (75 MHz, $CDCl_3$) δ 174.1 (C_q), 170.01 (C_q), 169.98 (C_q), 169.8 (C_q), 157.8 (C_q), 135.1 (C_q), 133.9 (C_q), 129.5 (CH), 129.1 (C_q), 127.4 (CH), 126.3 (CH), 126.2 (CH), 119.1 (CH), 105.7 (CH), 75.1 (CH), 73.8 (CH), 69.8 (CH), 67.7 (CH), 66.6 (CH), 60.8 (CH_2), 55.4 (CH_3), 45.3 (CH), 21.1 (CH_3), 20.71 (CH_3), 20.68 (CH_3), 18.5 (CH_3). HRMS (ESI) calcd for $C_{26}H_{29}INaO_{10}^+$ $[M+Na]^+$: 651.0698; Found: 651.0691.

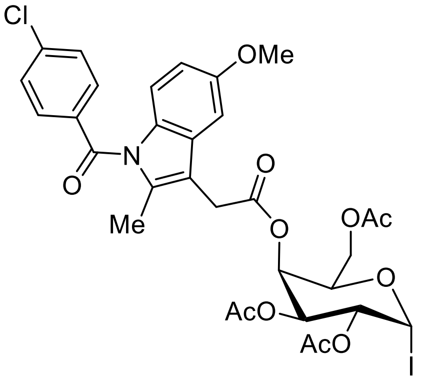

2t, foam, 0.32 g, 14% yield. ^{1}H NMR (300 MHz, $CDCl_3$) δ 7.73–7.64 (m, 3H), 7.38 (dd, *J* = 8.5, 1.9 Hz, 1H), 7.17–7.09 (m, 2H), 7.02 (d, *J* = 4.2 Hz, 1H), 5.47 (d, *J* = 3.4 Hz, 1H), 5.26 (dd, *J* = 10.6, 3.3 Hz, 1H), 4.33 (dd, *J* = 10.5, 4.2 Hz, 1H), 4.27 (dd, *J* = 10.5, 6.1 Hz, 1H), 4.23–4.16 (m, 1H), 4.07 (dd, *J* = 10.5, 6.3 Hz, 1H), 3.91 (s, 3H), 3.85 (q, *J* = 7.2 Hz, 1H), 2.13 (s, 3H), 2.10 (s, 3H), 2.01 (s, 3H), 1.56 (d, *J* = 7.2 Hz, 3H).

^{13}C NMR (75 MHz, $CDCl_3$) δ 170.1 (C_q), 169.8 (C_q), 169.7 (C_q), 169.6 (C_q), 168.3 (C_q), 156.1 (C_q), 139.3 (C_q), 136.2 (C_q), 133.9 (C_q), 131.3 (CH), 130.9 (C_q), 130.2 (C_q), 129.2 (CH), 115.2 (CH), 112.0 (CH), 111.8 (C_q), 101.1 (CH), 75.0 (CH), 73.5 (CH), 69.8 (CH), 67.5 (CH), 67.0 (CH), 60.2 (CH_2), 55.7 (CH), 30.1 (CH_2), 20.9 (CH_3), 20.6 (CH_3), 20.3 (CH_3), 13.3 (CH_3). HRMS (ESI) calcd for $C_{31}H_{31}ClINNaO_{11}^+$ [M+Na]$^+$: 778.0523; Found: 778.0514.

Optimization of reaction conditions

Table S1. Electrochemical synthesis of glycosyl BCP-H[a]

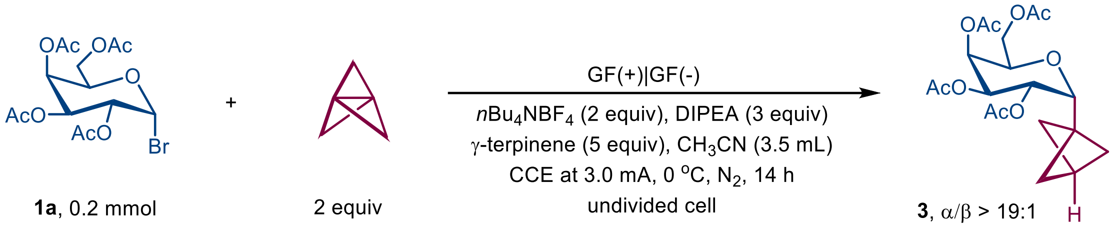

Entry	Variations	Yield of **3** (%)[b, c]
1	none	86
2	Et_3N instead of DIPEA	trace
3	2.0 mA/4.0 mA/6.0 mA	80/83/75
4	Pt(+)/GF(-)	65
5	GF(+)/Pt(-)	trace
6	Zn(+)/GF(-)	41
7	GF(+)/glassy carbon(-)	trace
8	GF(+)/RVC(-)	84
9	DMF/THF	75/trace
10	*w/o* current	N.R.[d]
11	*w/o* DIPEA	25
12	without/3 equiv/4 equiv γ-terpinene	56/78/82
13	nBu$_4$NClO$_4$/nBu$_4$NPF$_6$/LiClO$_4$	76/80/55
14	-20 °C/r.t.	85/70
15	air	73
16	CPE at 3 V	78
17	galactosyl I instead	35% **3**, 32% **21**

[a] Reaction conditions: **1a** (0.2 mmol), [1.1.1]propellane (0.4 mmol, $Et_2O/CH_2(OEt)_2$ solution, 0.5–0.7 M), γ-terpinene (1.0 mmol), DIPEA (0.6 mmol), nBu$_4$NBF$_4$ (0.4

mmol), CH_3CN (3.5 mL) at 0 °C, 14 h under N_2, GF as anode and cathode, CCE at 3.0 mA. [b] The ratio of α/β was determined by ^1H NMR of the crude mixture. [c] Determined by crude ^1H NMR using 1,3,5-trimethoxybenzene as an internal standard. [d] N.R.: no reaction.

Table S2. Electrochemical synthesis of glycosyl BCP-I[a]

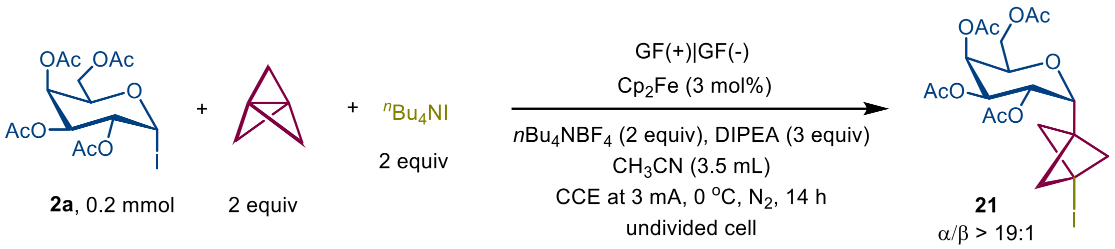

Entry	Variations	yield of **21** (%)[b, c]
1	none	88 (85)
2	*w/o* *n*Bu_4NBF_4	37
3	*w/o* *n*Bu_4NI	22
4	*w/o* Cp_2Fe	64
5	*w/o* DIPEA	38
6	galactosyl Br (1a)	N.D.[d]

[a] Reaction conditions: **2a** (0.2 mmol), [1.1.1]propellane (0.4 mmol, $Et_2O/CH_2(OEt)_2$ solution, 0.5–0.7 M), *n*Bu_4NI (0.4 mmol), DIPEA (0.6 mmol), *n*Bu_4NBF_4 (0.4 mmol), CH_3CN (4.0 mL) at 0 °C, 14 h under N_2, GF as anode and cathode, CCE at 3.0 mA. [b] The ratio of α/β was determined by ^1H NMR of the crude mixture. [c] Determined by crude ^1H NMR using 1,3,5-trimethoxybenzene as an internal standard. [d] N.D.: not detected.

Table S3. Electrochemical synthesis of glycosyl BCP-Bpin[a]

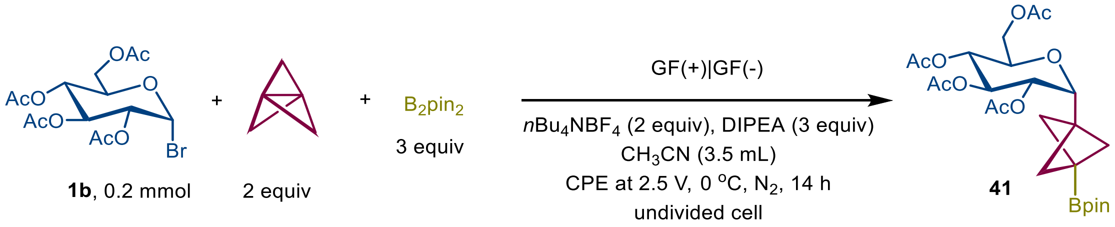

Entry	Variations	Yield of **41** (%)[b,c]

1	none	67
2	2.0 V/3.0 V/5.0 V	30 (28 h, 40% **1b** remain)/57/43
3	3.0 mA/2.0 mA	49/56
3	2 equiv B_2pin_2	56
4	Pt│GF	N.D.[d]
5	glucosyl I instead	64% **41**, 30% **23**
6	$Me_2Si(Ph)Bpin$	64
7	*w/o* DIPEA	N.D.

[a] Reaction conditions: **1b** (0.2 mmol), [1.1.1]propellane (0.4 mmol, $Et_2O/CH_2(OEt)_2$ solution, 0.5−0.7 M), B_2pin_2 (0.6 mmol), DIPEA (0.6 mmol), $n$$Bu_4NBF_4$ (0.4 mmol), CH_3CN (3.5 mL) at 0 °C, 14 h under N_2, GF as anode and cathode, CPE at 2.5 V. [b] The ratio of α/β was determined by ^{1}H NMR of the crude mixture. [c] Determined by crude ^{1}H NMR using 1,3,5-trimethoxybenzene as an internal standard. [d] N.D.: not detected.

As shown in Table S2 (Entry 3), the glycosyl BCP–I product **21** was still observed in the absence of TBAI, albeit in lower yield (22%). Similarly, in the borylation of the glycosyl BCP radical, the product was obtained in 30% yield using glucosyl I as substrate (Table S3, Entry 5). These results suggest that a chain radical mechanism may operate in the absence of an effective radical acceptor or when its reactivity is low.

General procedure for Electrochemical synthesis of diversely functionalized bicyclo[1.1.1]pentanyl glycosides

I. General procedure for electrochemical synthesis of glycosyl BCP−H

General procedure A (3−20)

The electrolysis was carried out in an undivided cell equipped with a graphite felt (GF) anode (10 mm × 15 mm × 2 mm) and a GF cathode (10 mm × 15 mm × 2 mm). In a Schlenk tube, tetrabutylammonium tetrafluoroborate (0.4 mmol, 131.6 mg) and the glycosyl bromide **1** (0.2 mmol) were added, followed by three cycles of evacuation and backfilling with nitrogen using a dual manifold Schlenk line. Under a nitrogen atmosphere, [1.1.1]propellane (0.4 mmol, $Et_2O/CH_2(OEt)_2$ solution, 0.5−0.7 M), γ-terpinene (1 mmol, 136.2 mg), DIPEA (0.6 mmol, 77.5 mg), and CH_3CN (3.5 mL) were added via syringe. Electrolysis was performed under a constant current of 3.0 mA for 14 hours at approximately 0 °C, using an ice bath placed in a thermally insulated Dewar flask covered with insulating cotton to minimize heat exchange. After completion, the reaction mixture was transferred to a flask, and the electrodes were rinsed with DCM (3 × 5.0 mL). The solvent was then removed under reduced pressure. The crude product was initially analyzed by NMR to determine the ratio of *α*- and *β*-products, followed by purification via column chromatography on silica gel (*n*-hexane/EtOAc) to afford the desired product.

II. General procedure for electrochemical synthesis of glycosyl BCP−I

General procedure B (21−40)

The electrolysis was carried out in an undivided cell equipped with a graphite felt (GF) anode (10 mm × 15 mm × 2 mm) and a GF cathode (10 mm × 15 mm × 2 mm). In a Schlenk tube, tetrabutylammonium tetrafluoroborate (0.4 mmol, 131.6 mg), ferrocene (0.006 mmol, 1.1 mg) and the glycosyl iodide **4** (0.2 mmol) were added, followed by three cycles of evacuation and backfilling with nitrogen using a dual manifold Schlenk

line. Under a nitrogen atmosphere, [1.1.1]propellane (0.4 mmol, $Et_2O/CH_2(OEt)_2$ solution, 0.5−0.7 M), DIPEA (0.6 mmol, 77.5 mg) and CH_3CN (3.5 mL) were added via syringe. Electrolysis was performed under a constant current of 3.0 mA for 10 minutes, after which tetrabutylammonium iodide (0.4 mmol, 147.7 mg) dissolved in 0.5 mL of CH_3CN was added to the reaction mixture via syringe. The reaction was then continued for 14 hours at approximately 0 °C, using an ice bath placed in a thermally insulated Dewar flask covered with insulating cotton to minimize heat exchange. After completion, the reaction mixture was transferred to a flask, and the electrodes were rinsed with DCM (3 × 5.0 mL). The solvent was then removed under reduced pressure. The crude product was initially analyzed by NMR to determine the ratio of *α*- and *β*-products, followed by purification via column chromatography on silica gel (*n*-hexane/EtOAc) to afford the desired product.

III. General procedure for electrochemical synthesis of glycosyl BCP−Bpin

General procedure C (41−51)

The electrolysis was carried out in an undivided cell equipped with a graphite felt (GF) anode (10 mm × 15 mm × 2 mm) and a GF cathode (10 mm × 15 mm × 2 mm). In a Schlenk tube, tetrabutylammonium tetrafluoroborate (0.4 mmol, 131.6 mg), B_2pin_2 (0.6 mmol, 152.4 mg), and the glycosyl bromide **1** (0.2 mmol) were added, followed by three cycles of evacuation and backfilling with nitrogen using a dual manifold Schlenk line. Under a nitrogen atmosphere, [1.1.1]propellane (0.4 mmol, $Et_2O/CH_2(OEt)_2$ solution, 0.5−0.7 M), DIPEA (0.6 mmol, 77.5 mg) and CH_3CN (3.5 mL) were added via syringe. Electrolysis was performed under a constant voltage of 2.5 V for 14 hours at approximately 0 °C, using an ice bath placed in a thermally insulated Dewar flask covered with insulating cotton to minimize heat exchange. After completion, the reaction mixture was transferred to a flask, and the electrodes were rinsed with DCM (3 × 5.0 mL). The solvent was then removed under reduced pressure. The crude product was initially analyzed by NMR to determine the ratio of *α*- and *β*-products, followed by

purification via column chromatography on silica gel (*n*-hexane/EtOAc) to afford the desired product.

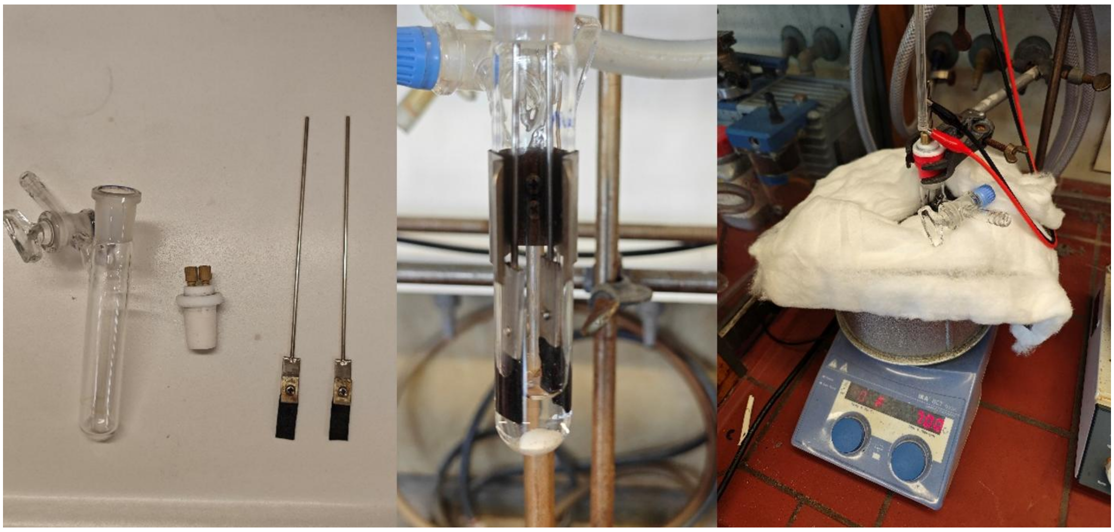

Full characterization of reaction products

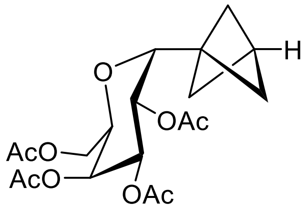

3, colorless syrup. 64.5 mg, 81% yield. >19:1 α/β (The α-configuration was confirmed by NOESY NMR analysis). ^{1}H NMR (600 MHz, $CDCl_3$) δ 5.41–5.38 (m, 1H), 5.32 (dd, J = 9.8, 3.4 Hz, 1H), 5.25 (dd, J = 9.9, 5.6 Hz, 1H), 4.28–4.25 (m, 1H), 4.17 (dd, J = 11.5, 7.5 Hz, 1H), 4.11 (d, J = 5.7 Hz, 1H), 4.03 (dd, J = 11.5, 5.3 Hz, 1H), 2.53 (s, 1H), 2.11 (s, 3H), 2.06 (s, 3H), 2.05 (s, 3H), 2.01 (s, 3H), 1.98–1.95 (m, 3H), 1.89–1.86 (m, 3H). ^{13}C NMR (126 MHz, $CDCl_3$) δ 170.7 (C_q), 170.3 (C_q), 170.1 (C_q), 170.0 (C_q), 70.9 (CH), 69.6 (CH), 68.8 (CH), 68.0 (CH), 67.8 (CH), 62.0 (CH_2), 52.0 (CH_2), 44.4 (CH_2), 29.3 (CH), 21.0 (CH_3), 20.9 (CH_3), 20.8 (CH_3), 20.8 (CH_3). HRMS (ESI) calcd for $C_{19}H_{26}NaO_9^+$ $[M+Na]^+$: 421.1469; Found: 421.1463.

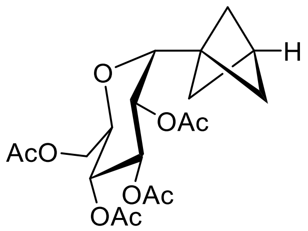

4, colorless syrup. 55.8 mg, 70% yield. >19:1 α/β (The α-configuration was confirmed by NOESY NMR analysis). ^{1}H NMR (500 MHz, $CDCl_3$) δ 5.44 (t, J = 9.4 Hz, 1H), 5.03 (dd, J = 9.9, 6.2 Hz, 1H), 4.96–4.88 (m, 1H), 4.18–4.13 (m, 1H), 4.11–4.03 (m, 3H), 2.55 (s, 1H), 2.07 (s, 3H), 2.03–1.99 (m, 12H), 1.92–1.89 (m, 3H). ^{13}C NMR (75 MHz, $CDCl_3$) δ 170.8 (C_q), 170.2 (C_q), 169.8 (C_q), 169.7 (C_q), 71.2 (CH), 70.5 (CH), 70.2 (CH), 69.0 (CH), 62.7 (CH_2), 52.2 (CH_2), 44.4 (C_q), 29.6 (CH), 20.8 (CH_3), 20.8 (CH_3). HRMS (ESI) calcd for $C_{19}H_{26}NaO_9^+$ $[M+Na]^+$: 421.1469; Found: 421.1462.

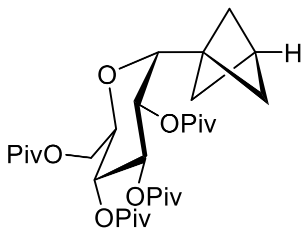

5, colorless syrup. 104.3 mg, 92% yield. >19:1 *α*/*β* (The *α*-configuration was confirmed by NOESY NMR analysis). ^{1}H NMR (600 MHz, $CDCl_3$) δ 5.58–5.54 (m, 1H), 5.07 (dd, *J* = 10.3, 6.3 Hz, 1H), 4.98 (t, *J* = 9.6 Hz, 1H), 4.11–4.10 (m, 1H), 4.10–4.08 (m, 1H), 4.06 (d, *J* = 6.3 Hz, 1H), 3.95 (dd, *J* = 12.5, 6.4 Hz, 1H), 2.55 (s, 1H), 2.04 (dd, *J* = 9.5, 1.9 Hz, 3H), 1.96 (dd, *J* = 9.5, 1.9 Hz, 3H), 1.20 (s, 9H), 1.16 (s, 9H), 1.15 (s, 9H), 1.11 (s, 9H). ^{13}C NMR (75 MHz, $CDCl_3$) δ 178.2 (C_q), 177.4 (C_q), 177.3 (C_q), 176.7 (C_q), 71.8 (CH), 71.6 (CH), 70.8 (CH), 70.0 (CH), 68.8 (CH), 63.0 (CH_2), 52.6 (CH_2), 44.6 (C_q), 38.9 (C_q), 38.83 (C_q), 38.81 (C_q), 29.6 (CH), 27.5 (CH_3), 27.31 (CH_3), 27.26 (CH_3), 27.2 (CH_3). HRMS (ESI) calcd for $C_{31}H_{50}NaO_9^+$ [M+Na]$^+$: 589.3347; Found: 589.3340.

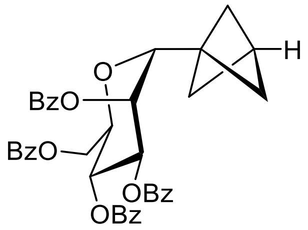

6, colorless syrup. 43.9 mg, 34% yield. >19:1 *α*/*β*. ^{1}H NMR (300 MHz, $CDCl_3$) δ 8.14–7.83 (m, 8H), 7.63–7.25 (m, 12H), 6.02 (t, *J* = 9.1 Hz, 1H), 5.87–5.76 (m, 2H), 4.69–4.51 (m, 3H), 4.19 (d, *J* = 2.4 Hz, 1H), 2.66 (s, 1H), 2.20–2.04 (m, 6H). ^{13}C NMR (75 MHz, $CDCl_3$) δ 166.4 (C_q), 165.9 (C_q), 165.7 (C_q), 165.6 (C_q), 133.6 (CH), 133.5 (CH), 133.4 (CH), 133.2 (CH), 130.0 (CH), 129.93 (CH), 129.91 (CH), 129.87 (CH), 129.85 (CH), 129.7 (C_q), 129.2 (C_q), 129.1 (C_q), 128.7 (CH), 128.63 (CH), 128.60 (CH), 128.55 (CH), 128.52 (CH), 75.5 (CH), 72.2 (CH), 70.5 (CH), 70.3 (CH), 67.5 (CH), 63.4 (CH_2), 51.5 (CH_2), 44.3 (C_q), 28.5 (CH). HRMS (ESI) calcd for $C_{39}H_{34}NaO_9^+$ [M+Na]$^+$: 669.2095; Found: 669.2087.

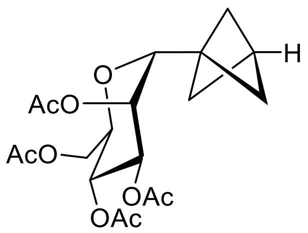

7, colorless syrup. 59.7 mg, 75% yield. >19:1 *α*/*β* (The *α*-configuration was confirmed by NOESY NMR analysis). ^{1}H NMR (300 MHz, $CDCl_3$) δ 5.28–5.12 (m, 3H), 4.24 (dd, *J* = 12.0, 6.2 Hz, 1H), 4.11–3.98 (m, 2H), 3.81 (d, *J* = 2.6 Hz, 1H), 2.54 (s, 1H), 2.09 (s, 3H), 2.07 (s, 3H), 2.03 (s, 3H), 1.99 (s, 3H), 1.95 (dd, *J* = 9.5, 1.8 Hz, 3H), 1.87 (dd, *J* = 9.6, 1.9 Hz, 3H). ^{13}C NMR (75 MHz, $CDCl_3$) δ 170.8 (C_q), 170.3 (C_q), 170.2 (C_q), 169.8 (C_q), 75.0 (CH), 71.9 (CH), 69.4 (CH), 69.0 (CH), 66.7 (CH), 62.9 (CH_2), 51.2 (CH_2), 44.1 (C_q), 28.3 (CH), 21.1 (CH_3), 20.9 (CH_3), 20.8 (CH_3). HRMS (ESI) calcd for $C_{19}H_{26}NaO_9^+$ [M+Na]$^+$: 421.1469; Found: 421.1462.

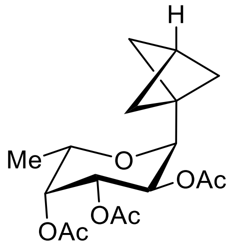

8, colorless syrup. 45.6 mg, 67% yield. >19:1 *α*/*β* (The *α*-configuration was confirmed by NOESY NMR analysis). ^{1}H NMR (400 MHz, $CDCl_3$) δ 5.35 (dd, *J* = 10.4, 3.4 Hz, 1H), 5.31 – 5.24 (m, 2H), 4.17 (qd, *J* = 6.4, 1.7 Hz, 1H), 4.07 (d, *J* = 6.0 Hz, 1H), 2.53 (s, 1H), 2.14 (s, 3H), 2.05 (s, 3H), 2.00 (s, 3H), 1.99 (dd, *J* = 9.5, 1.9 Hz, 3H), 1.88 (dd, *J* = 9.5, 1.9 Hz, 3H), 1.12 (d, *J* = 6.5 Hz, 3H). ^{13}C NMR (101 MHz, $CDCl_3$) δ 170.8 (C_q), 170.3 (C_q), 170.2 (C_q), 71.3 (CH), 71.1 (CH), 69.0 (CH), 68.4 (CH), 67.3 (CH), 52.4 (CH_2), 44.8 (C_q), 29.2 (CH), 21.0 (CH_3), 20.9 (CH_3), 20.8 (CH_3), 16.5 (CH_3). HRMS (ESI) calcd for $C_{17}H_{24}NaO_7^+$ [M+Na]$^+$: 363.1414; Found: 363.1414.

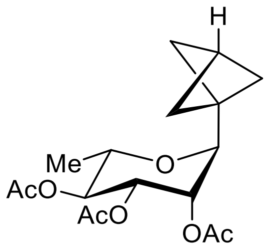

9, colorless syrup. 48.3 mg, 71% yield. >19:1 *α*/*β* (The *α*-configuration was confirmed by NOESY NMR analysis). ^{1}H NMR (400 MHz, $CDCl_3$) δ 5.29 (dd, *J* = 3.4, 2.3 Hz, 1H), 5.19 (dd, *J* = 9.6, 3.4 Hz, 1H), 5.02 (t, *J* = 9.3 Hz, 1H), 3.94 – 3.87 (m, 1H), 3.74 (d, *J* = 2.3 Hz, 1H), 2.55 (s, 1H), 2.11 (s, 3H), 2.04 (s, 3H), 1.99 (s, 3H), 1.97 (dd, *J* = 9.6, 2.0 Hz, 3H), 1.89 (dd, *J* = 9.6, 1.9 Hz, 3H), 1.20 (d, *J* = 6.2 Hz, 3H). ^{13}C NMR (101 MHz, $CDCl_3$) δ 170.5 (C_q), 170.4 (C_q), 170.0 (C_q), 75.4 (CH), 71.3 (CH), 69.7 (CH), 69.6 (CH), 69.5 (CH), 51.4 (CH_2), 44.4 (C_q), 28.1 (CH), 21.1 (CH_3), 21.0 (CH_3), 20.8 (CH_3), 18.0 (CH_3). HRMS (ESI) calcd for $C_{17}H_{24}NaO_7^+$ $[M+Na]^+$: 363.1415; Found: 363.1414.

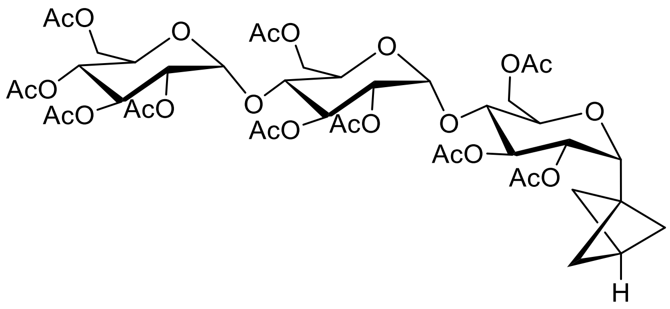

10, colorless syrup. 95.6 mg, 49% yield. >19:1 *α*/*β*. ^{1}H NMR (400 MHz, $CDCl_3$) δ 5.45 – 5.32 (m, 3H), 5.23 – 5.15 (m, 2H), 5.07 (t, *J* = 9.9 Hz, 1H), 4.97 (dd, *J* = 6.9, 5.1 Hz, 1H), 4.87 (dd, *J* = 10.5, 4.0 Hz, 1H), 4.78 (dd, *J* = 10.4, 3.9 Hz, 1H), 4.47 (dd, *J* = 12.4, 2.3 Hz, 1H), 4.31 – 3.89 (m, 10H), 3.64 (dd, *J* = 7.9, 5.1 Hz, 1H), 2.56 (s, 1H), 2.14 (s, 6H), 2.12 (s, 3H), 2.09 (s, 3H), 2.04 (s, 3H), 2.03 (s, 9H), 2.00 (s, 3H), 1.99 (s, 3H), 1.97 (dd, *J* = 9.5, 1.8 Hz, 3H), 1.89 (dd, *J* = 9.5, 1.8 Hz, 3H). ^{13}C NMR (101 MHz, $CDCl_3$) δ 170.8 (C_q), 170.7 (C_q), 170.62 (C_q), 170.57 (C_q), 170.3 (C_q), 170.0 (C_q), 169.91 (C_q), 169.86 (C_q), 169.6 (C_q), 96.5 (CH), 96.0 (CH), 75.1 (CH), 73.0 (CH), 72.1

(CH), 71.4 (CH), 70.9 (CH), 70.5 (CH), 70.2 (CH), 70.1 (CH), 69.5 (CH), 68.8 (CH), 68.7 (CH), 68.0 (CH), 63.4 (CH_2), 62.6 (CH_2), 61.5 (CH_2), 51.2 (CH_2), 44.2 (C_q), 29.4 (CH), 21.04 (CH_3), 21.02 (CH_3), 21.00 (CH_3), 20.9 (CH_3), 20.8 (CH_3), 20.7 (CH_3), 20.6 (CH_3). HRMS (ESI) calcd for $C_{43}H_{58}NaO_{25}^+$ $[M+Na]^+$: 997.3159; Found: 997.3159.

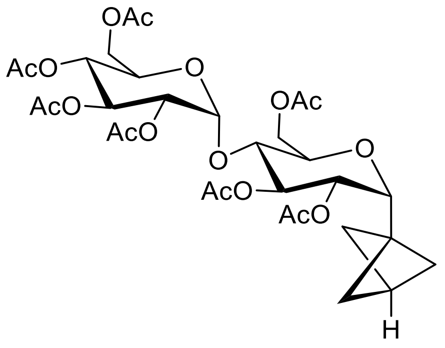

11, colorless syrup. 83.8 mg, 61% yield. >19:1 *α/β*. ^{1}H NMR (400 MHz, $CDCl_3$) δ 5.40 – 5.21 (m, 3H), 5.03 (t, *J* = 10.2 Hz, 1H), 4.96 – 4.84 (m, 2H), 4.31 – 4.18 (m, 3H), 4.08 (td, *J* = 12.5, 2.6 Hz, 3H), 3.93 (d, *J* = 5.1 Hz, 1H), 3.67 (t, *J* = 6.8 Hz, 1H), 2.54 (s, 1H), 2.10 (s, 3H), 2.08 (s, 6H), 2.04 (s, 3H), 2.02 (s, 3H), 2.01 (s, 3H), 1.98 (s, 3H), 1.97 – 1.92 (m, 3H), 1.89 – 1.83 (m, 3H). ^{13}C NMR (101 MHz, $CDCl_3$) δ 170.67 (C_q), 170.66 (C_q), 170.5 (C_q), 170.1 (C_q), 169.8 (C_q), 169.5 (C_q), 96.4 (CH), 74.1 (CH), 71.5 (CH), 71.2 (CH), 70.3(CH), 70.1 (CH), 69.7 (CH), 68.5(CH), 68.3 (CH), 63.1 (CH_2), 61.7 (CH_2), 51.3 (CH_2), 44.2 (C_q), 29.4 (CH), 21.0 (CH_3), 20.93 (CH_3), 20.91 (CH_3), 20.77 (CH_3), 20.73 (CH_3), 20.69 (CH_3), 20.64 (CH_3). HRMS (ESI) calcd for $C_{31}H_{42}NaO_{17}^+$ $[M+Na]^+$: 709.2314; Found: 709.2311.

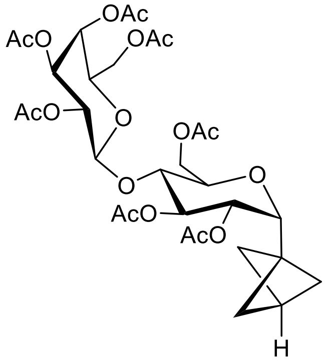

12, colorless syrup. 85.1 mg, 62% yield. >19:1 *α/β*. ^{1}H NMR (400 MHz, $CDCl_3$) δ 5.47

– 5.41 (m, 1H), 5.34 (d, J = 3.4 Hz, 1H), 5.11 (dd, J = 10.4, 7.9 Hz, 1H), 4.98 – 4.92 (m, 2H), 4.50 (d, J = 8.0 Hz, 1H), 4.33 (dd, J = 11.6, 2.5 Hz, 1H), 4.12 – 4.06 (m, 3H), 4.03 – 3.94 (m, 2H), 3.88 (t, J = 6.8 Hz, 1H), 3.63 – 3.56 (m, 1H), 2.53 (s, 1H), 2.13 (s, 3H), 2.10 (s, 3H), 2.05 (s, 3H), 2.04 (s, 3H), 2.04 (s, 6H), 1.97 (dd, J = 9.5, 1.9 Hz, 3H), 1.95 (s, 3H), 1.87 (dd, J = 9.5, 1.9 Hz, 3H). ^{13}C NMR (101 MHz, $CDCl_3$) δ 170.6 (C_q), 170.5 (C_q), 170.3 (C_q), 170.2 (C_q), 170.1 (C_q), 169.7 (C_q), 169.3 (C_q), 101.4 (CH), 77.0 (CH), 71.6 (CH), 71.2 (CH), 70.9 (CH), 70.83 (CH), 70.76 (CH), 69.7 (CH), 69.2 (CH), 66.9 (CH), 62.8 (CH_2), 61.0 (CH_2), 51.9 (CH_2), 44.3 (C_q), 29.4 (CH), 21.0 (CH_3), 20.94 (CH_3), 20.92 (CH_3), 20.78 (CH_3), 20.76 (CH_3), 20.6 (CH_3). HRMS (ESI) calcd for $C_{31}H_{42}NaO_{17}^+$ $[M+Na]^+$: 709.2314; Found: 709.2312.

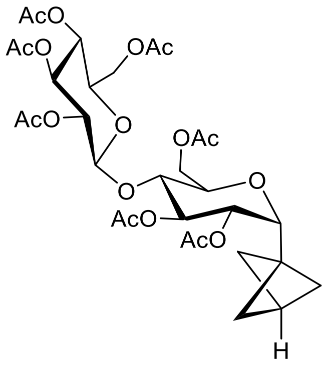

13, colorless syrup. 79.6 mg, 58% yield. >19:1 *α/β*. ^{1}H NMR (400 MHz, $CDCl_3$) δ 5.41 (t, J = 8.4 Hz, 1H), 5.12 (t, J = 9.3 Hz, 1H), 5.04 (t, J = 9.6 Hz, 1H), 4.98 – 4.87 (m, 2H), 4.52 (d, J = 8.1 Hz, 1H), 4.38 – 4.28 (m, 2H), 4.09 – 3.94 (m, 4H), 3.69 – 3.62 (m, 1H), 3.59 – 3.52 (m, 1H), 2.52 (s, 1H), 2.09 (s, 3H), 2.06 (s, 3H), 2.02 (s, 9H), 1.99 (s, 3H), 1.96 (s, 3H), 1.95 (dd, J = 9.4, 1.8 Hz, 3H), 1.86 (dd, J = 9.4, 1.8 Hz, 3H). ^{13}C NMR (101 MHz, $CDCl_3$) δ 170.64 (C_q), 170.55 (C_q), 170.3 (C_q), 170.1 (C_q), 169.8 (C_q), 169.4 (C_q), 169.3 (C_q), 101.0 (CH), 77.3 (CH), 73.1 (CH), 72.0 (CH), 71.7 (CH), 71.6 (CH), 70.9 (CH), 70.8 (CH), 69.4 (CH), 67.9 (CH), 62.7 (CH_2), 61.7 (CH_2), 51.9 (CH_2), 44.3 (C_q), 29.4 (CH), 20.92 (CH_3), 20.91 (CH_3), 20.88 (CH_3), 20.79 (CH_3), 20.76 (CH_3), 20.67 (CH_3), 20.66 (CH_3), 20.65 (CH_3). HRMS (ESI) calcd for $C_{31}H_{42}NaO_{17}^+$ $[M+Na]^+$: 709.2314; Found: 709.2312.

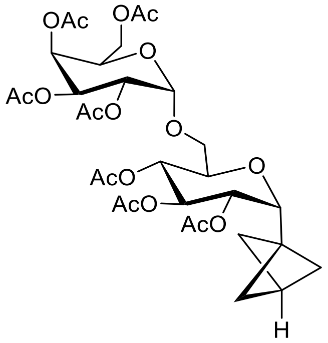

14, colorless syrup. 75.5 mg, 55% yield. >19:1 α/β. ^{1}H NMR (400 MHz, $CDCl_3$) δ 5.48 – 5.41 (m, 2H), 5.37 – 5.30 (m, 1H), 5.12 – 5.07 (m, 2H), 4.97 (dd, J = 9.9, 6.3 Hz, 1H), 4.86 (t, J = 9.4 Hz, 1H), 4.30 (t, J = 6.5 Hz, 1H), 4.13 – 3.97 (m, 4H), 3.62 (dd, J = 11.0, 6.3 Hz, 1H), 3.48 (dd, J = 11.0, 2.5 Hz, 1H), 2.56 (s, 1H), 2.11 (s, 3H), 2.09 (s, 3H), 2.04 – 2.01 (m, 12H), 2.00 (s, 3H), 1.96 (s, 3H), 1.91 (dd, J = 9.5, 1.9 Hz, 3H). ^{13}C NMR (101 MHz, $CDCl_3$) δ 170.6 (C_q), 170.5 (C_q), 170.3 (C_q), 170.2 (C_q), 170.0 (C_q), 169.8 (C_q), 169.7 (C_q), 96.1 (CH), 71.4 (CH), 71.0 (CH), 70.3 (CH), 69.5 (CH), 68.3 (CH), 68.2 (CH), 67.6 (CH), 66.8 (CH_2), 66.5 (CH), 61.9 (CH_2) , 52.2 (CH_2), 44.4 (C_q), 29.7 (CH), 20.9 (CH_3), 20.8 (CH_3), 20.75 (CH_3), 20.72 (CH_3). HRMS (ESI) calcd for $C_{31}H_{42}NaO_{17}^+$ $[M+Na]^+$: 709.2314; Found: 709.2316.

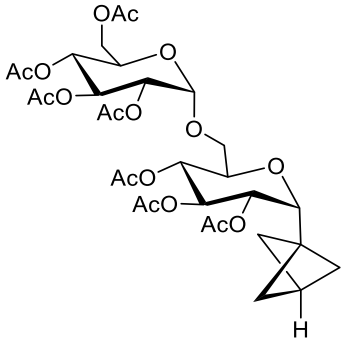

15, colorless syrup. 87.9 mg, 64% yield. >19:1 α/β. ^{1}H NMR (400 MHz, $CDCl_3$) δ 5.50 – 5.43 (m, 2H), 5.06 – 4.95 (m, 3H), 4.87 – 4.82 (m, 2H), 4.23 – 4.05 (m, 4H), 4.02 (d, J = 6.3 Hz, 1H), 3.62 (dd, J = 10.9, 6.5 Hz, 1H), 3.51 (dd, J = 10.9, 2.6 Hz, 1H), 2.57 (s, 1H), 2.08 (s, 3H), 2.08 (s, 3H), 2.04 – 1.98 (m, 18H), 1.92 (dd, J = 9.5, 1.9 Hz, 3H). ^{13}C NMR (101 MHz, $CDCl_3$) δ 170.8 (C_q), 170.3 (C_q), 170.2 (C_q), 170.1 (C_q), 169.9

(C_q), 169.75 (C_q), 169.72 (C_q), 95.6 (CH), 71.4 (CH), 71.4 (CH), 71.1 (CH), 70.8 (CH), 70.3 (CH), 70.1 (CH), 69.7 (CH), 68.5 (CH), 67.4 (CH), 67.2 (CH_2), 61.9 (CH_2), 52.3 (CH_2), 44.4 (C_q), 29.7 (CH), 20.9 (CH_3), 20.84 (CH_3), 20.83 (CH_3), 20.80 (CH_3), 20.7 (CH_3). HRMS (ESI) calcd for $C_{31}H_{42}NaO_{17}^+$ $[M+Na]^+$: 709.2314; Found: 709.2317.

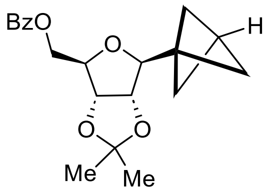

16, colorless syrup. 35.8 mg, 52% yield. only *β*. ^{1}H NMR (400 MHz, $CDCl_3$) δ 8.07 – 8.03 (m, 2H), 7.60 – 7.55 (m, 1H), 7.47 – 7.43 (m, 2H), 4.59 (dd, *J* = 6.7, 4.4 Hz, 1H), 4.51 (dd, *J* = 11.9, 4.2 Hz, 1H), 4.45 (dd, *J* = 6.7, 4.6 Hz, 1H), 4.38 (dd, *J* = 7.5, 4.3 Hz, 1H), 4.25 – 4.21 (m, 1H), 3.89 (d, *J* = 4.7 Hz, 1H), 2.53 (s, 1H), 1.80 – 1.72 (m, 6H), 1.55 (s, 3H), 1.35 (s, 3H). ^{13}C NMR (101 MHz, $CDCl_3$) δ 166.5 (C_q), 133.3 (CH), 130.0 (C_q), 129.8 (CH), 128.5 (CH), 114.7 (C_q), 83.6 (CH), 82.3 (CH), 82.2 (CH), 81.8 (CH), 64.7 (CH_2), 48.5 (CH_2), 44.6 (C_q), 28.7 (CH), 27.7 (CH_3), 25.8 (CH_3). HRMS (ESI) calcd for $C_{20}H_{24}NaO_5^+$ $[M+Na]^+$: 367.1516; Found: 367.1513.

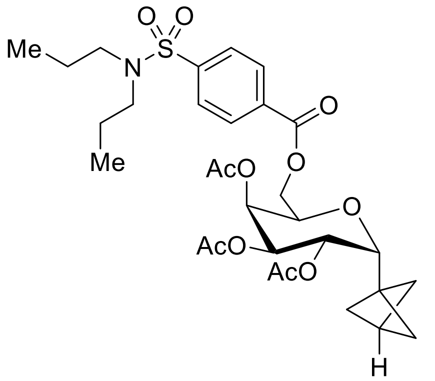

17, foam, 0.079 g, 63% yield. >19:1 *α*/*β*. ^{1}H NMR (300 MHz, $CDCl_3$) δ 8.14 (d, *J* = 8.3 Hz, 2H), 7.88 (d, *J* = 8.3 Hz, 2H), 5.52–5.48 (m, 1H), 5.37 (dd, *J* = 9.7 Hz, *J* = 3.2 Hz, 1H), 5.29 (dd, *J* = 9.7 Hz, *J* = 5.4 Hz, 1H), 4.56 (dd, *J* = 11.2 Hz, *J* = 7.7 Hz, 1H), 4.42 (ddd, *J* = 7.6 Hz, *J* = 5.0 Hz, *J* = 2.2 Hz, 1H), 4.23 (dd, *J* = 11.2 Hz, *J* = 5.0 Hz, 1H), 4.16 (d, *J* = 5.4 Hz, 1H), 3.14–3.07 (m, 4H), 2.53 (s, 1H), 2.14 (s, 3H), 2.08 (s,

3H), 2.04 (s, 3H), 1.96 (dd, *J* = 9.5 Hz, *J* = 1.8 Hz, 3H), 1.87 (dd, *J* = 9.5 Hz, *J* = 1.8 Hz, 1H), 1.61–1.49 (m, 4H), 0.87 (t, *J* = 7.4 Hz, 6H) ^{13}C NMR (75 MHz, $CDCl_3$) δ 170.2 (C_q), 170.01 (C_q), 169.97 (C_q), 164.9 (C_q), 144.6 (C_q), 132.9 (C_q), 130.4 (CH), 127.2 (CH), 70.7 (CH), 69.5 (CH), 68.8 (CH), 67.9 (CH), 67.7 (CH), 62.9 (CH_2), 51.9 (CH_2), 50.1 (CH_2), 44.3 (C_q), 29.3 (C_q), 22.0 (CH_2), 20.9 (CH_3), 20.8 (CH_3), 20.8 (CH_3), 11.2 (CH_3). HRMS (ESI) calcd for $C_{30}H_{41}NNaO_{11}S^+$ [M+Na]$^+$: 646.2293; Found: 646.2292.

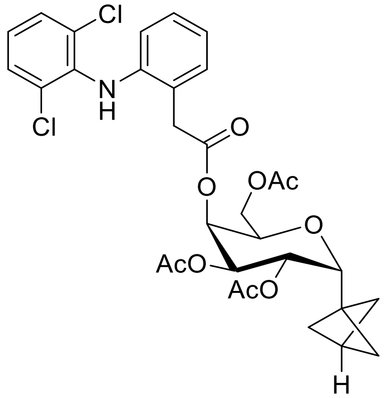

18, foam, 0.072 g, 57% yield. >19:1 *α*/*β*. ^{1}H NMR (300 MHz, $CDCl_3$) δ 7.33 (d, *J* = 8.0 Hz, 2H), 7.27 (dd, *J* = 7.3, 1.6 Hz, 1H), 7.11 (td, *J* = 7.7, 1.7 Hz, 1H), 6.97 (dd, *J* = 8.4, 7.8 Hz, 1H), 6.96 (td, *J* = 7.4, 1.2 Hz, 1H), 6.84 (s, 1H), 6.53 (d, *J* = 8.0 Hz, 1H), 5.47 (dd, *J* = 3.0, 1.9 Hz, 1H), 5.34–5.30 (m, 2H), 4.30 (ddd, *J* = 7.5, 5.5, 2.1 Hz, 1H), 4.21–4.12 (m, 2H), 4.02–3.80 (m, 3H), 2.54 (s, 1H), 2.05 (s, 3H), 2.01 (s, 3H), 1.98 (dd, *J* = 9.6, 1.8 Hz, 3H), 1.88 (dd, *J* = 9.5, 1.8 Hz, 3H), 1.74 (s, 3H). ^{13}C NMR (75 MHz, $CDCl_3$) δ 172.1 (C_q), 170.6 (C_q), 170.1 (C_q), 170.0 (C_q), 142.7 (C_q), 138.0 (C_q), 131.0 (CH), 129.6 (C_q), 129.0 (CH), 128.2 (CH), 124.3 (C_q), 124.2 (CH), 122.4, (CH) 118.6 (CH), 71.0 (CH), 69.4 (CH), 68.9 (CH), 68.8 (CH), 67.6 (CH), 62.0 (CH_2), 52.1 (CH_2), 44.4 (C_q), 38.2 (CH_2), 29.3 (CH), 21.0 (CH_3), 20.8 (CH_3), 20.4 (CH_3). HRMS (ESI) calcd for $C_{33}H_{31}Cl_2NNaO_9^+$ [M+Na]$^+$: 656.1425; Found: 656.1438.

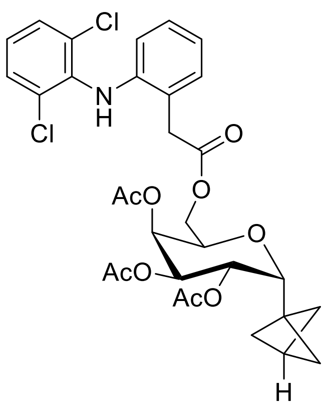

19, foam, 0.071 g, 56% yield. >19:1 *α*/*β*. ^{1}H NMR (300 MHz, $CDCl_3$) δ 7.34 (d, *J* = 8.1 Hz, 2H), 7.22 (dd, *J* = 7.5, 1.6 Hz, 1H), 7.12 (td, *J* = 7.7, 1.6 Hz, 1H), 6.98 (dd, *J* = 8.4, 7.7 Hz, 1H), 6.95 (td, *J* = 7.5, 1.2 Hz, 1H), 6.80 (s, 1H), 6.54 (d, *J* = 8.0 Hz, 1H), 5.42 (dd, *J* = 3.3, 1.9 Hz, 1H), 5.32 (dd, *J* = 9.5, 3.3 Hz, 1H), 5.24 (dd, *J* = 9.6, 5.3 Hz, 1H), 4.35–4.22 (m, 2H), 4.17–4.06 (m, 2H), 3.83 (s, 2H), 2.47 (s, 1H), 2.10 (s, 3H), 2.06 (s, 3H), 2.02 (s, 3H), 1.90 (dd, *J* = 9.6, 1.8 Hz, 3H), 1.82 (dd, *J* = 9.5, 1.8 Hz, 3H) ^{13}C NMR (75 MHz, $CDCl_3$) δ 172.1 (C_q), 170.2 (C_q), 170.00 (C_q), 169.96 (C_q), 142.8 (C_q), 137.9 (C_q), 131.1 (CH), 129.7 (C_q), 129.0 (CH), 128.2 (CH), 124.2 (CH), 124.1 (C_q), 122.2 (CH), 118.5 (CH), 70.6 (CH), 69.7 (CH), 68.8 (CH), 68.0 (CH), 67.7 (CH), 62.9 (CH_2), 51.9 (CH_2), 44.2 (C_q), 38.4 (CH_2), 29.2 (CH), 21.0 (CH_3), 20.9 (CH_3), 20.8 (CH_3). HRMS (ESI) calcd for $C_{33}H_{31}Cl_2NNaO_9^+$ $[M+Na]^+$: 656.1425; Found: 656.1439.

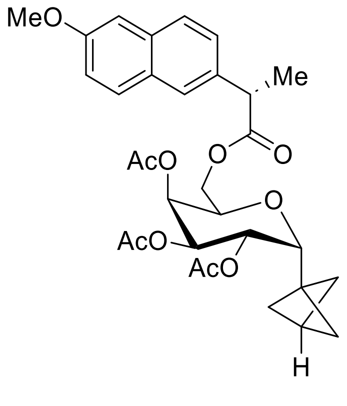

20, foam, 0.087 g, 77% yield. >19:1 *α*/*β*. ^{1}H NMR (300 MHz, $CDCl_3$) δ 7.73–7.64 (m, 3H), 7.39 (dd, *J* = 8.5, 1.9 Hz, 1H), 7.17–7.09 (m, 2H), 5.37 (dd, *J* = 3.2, 2.0 Hz, 1H), 5.29 (dd, *J* = 9.5, 3.2 Hz, 1H), 5.21 (dd, *J* = 9.5, 5.2 Hz, 1H), 4.24–4.16 (m, 2H), 4.11–

4.02 (m, 2H), 3.91 (s, 3H), 3.86 (q, J = 7.0 Hz, 1H), 2.45 (s, 1H), 2.09 (s, 3H), 2.05 (s, 3H), 2.01 (s, 3H), 1.86 (dd, J = 9.5, 1.8 Hz, 3H), 1.76 (dd, J = 9.5, 1.8 Hz, 3H), 1.57 (d, J = 7.2 Hz, 3H). ^{13}C NMR (75 MHz, $CDCl_3$) δ 174.4 (C_q), 170.2 (C_q), 169.99 (C_q), 169.96 (C_q), 157.8 (C_q), 135.3 (C_q), 133.9 (C_q), 129.4 (CH), 129.1 (C_q), 127.3 (CH), 126.3 (CH), 126.2 (CH), 119.1 (CH), 105.7 (CH), 70.5 (CH), 69.7 (CH), 68.9 (CH), 67.8 (CH), 67.7 (CH), 62.0 (CH_2), 55.4 (CH_3), 51.9 (CH_2), 45.4 (CH), 44.2 (C_q), 29.2 (CH), 21.0 (CH_3), 20.9 (CH_3), 20.8 (CH_3), 18.5 (CH_3). HRMS (ESI) calcd for $C_{31}H_{36}NaO_{10}^+$ $[M+Na]^+$: 591.2201; Found: 591.2196.

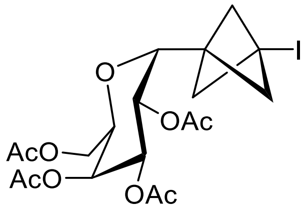

21, colorless syrup. 89.1 mg, 85% yield, >19:1 α/β (The α-configuration was confirmed by NOESY NMR analysis). ^{1}H NMR (600 MHz, $CDCl_3$) δ 5.35 (t, J = 2.7 Hz, 1H), 5.22–5.16 (m, 2H), 4.24 (dd, J = 11.7, 7.8 Hz, 1H), 4.20–4.18 (m, 1H), 4.16–4.13 (m, 1H), 3.95 (dd, J = 11.6, 4.9 Hz, 1H), 2.40 (dd, J = 9.3, 1.9 Hz, 3H), 2.33 (dd, J = 9.3, 1.9 Hz, 3H), 2.07 (s, 3H), 2.05 (s, 3H), 2.03 (s, 3H), 2.00 (s, 3H). ^{13}C NMR (126 MHz, $CDCl_3$) δ 170.6 (C_q), 170.0 (C_q), 169.7 (C_q), 169.7 (C_q), 70.2 (CH), 70.1 (CH), 68.3 (CH), 67.3 (CH), 61.4 (CH_2), 61.0 (CH_2), 47.1 (C_q), 20.9 (CH_3), 20.8 (CH_3), 20.8 (CH_3), 20.7 (CH_3), 5.9 (C_q). HRMS (ESI) calcd for $C_{19}H_{25}INaO_9^+$ $[M+Na]^+$: 547.0435; Found: 547.0432.

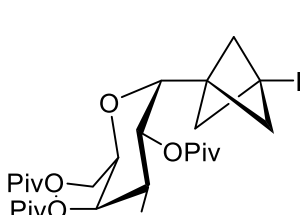

22, white foam. 119.0 mg, 86% yield, >19:1 α/β. ^{1}H NMR (300 MHz, $CDCl_3$) δ 5.36 (t, J = 2.8 Hz, 1H), 5.29–5.18 (m, 2H), 4.44–4.33 (m, 1H), 4.24–4.13 (m, 2H), 3.83 (dd,

J = 11.6, 4.6 Hz, 1H), 2.44–2.33 (m, 6H), 1.19 (s, 9H), 1.18 (s, 9H), 1.17 (s, 9H), 1.13 (s, 9H). ^{13}C NMR (75 MHz, $CDCl_3$) δ 178.1, 177.2, 177.1, 176.9, 70.7, 70.3, 68.4, 67.4, 67.1, 61.2, 47.3, 39.1, 39.0, 38.8, 38.8, 27.4, 27.3, 27.22, 27.21, 5.9. HRMS (ESI) calcd for $C_{31}H_{49}INaO_9^+$ $[M+Na]^+$: 715.2313; Found: 715.2305.

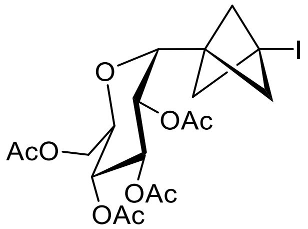

23, white foam. 84.9 mg, 81% yield, 11:1 *α/β.* ^{1}H NMR (300 MHz, $CDCl_3$) δ 5.30 (t, J = 9.1 Hz, 1H), 5.01 (dd, J = 9.5, 5.9 Hz, 1H), 4.88 (t, J = 9.0 Hz, 1H), 4.17 (dd, J = 12.7, 6.0 Hz, 2H), 4.05–3.93 (m, 2H), 2.50–2.35 (m, 6H), 2.05 (s, 3H), 2.03 (s, 3H), 2.00 (s, 6H).

Data are in accordance with literature[1].

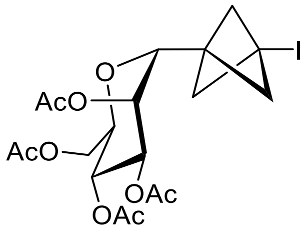

24, colorless syrup. 91.2 mg, 87% yield, only *α.* ^{1}H NMR (300 MHz, $CDCl_3$) δ 5.18–5.04 (m, 3H), 4.38 (dd, J = 12.0, 7.0 Hz, 1H), 4.07–3.89 (m, 3H), 2.41 (dd, J = 9.4, 1.8 Hz, 3H), 2.34 (dd, J = 9.3, 1.8 Hz, 3H), 2.07 (s, 3H), 2.07 (s, 3H), 2.06 (s, 3H), 2.02 (s, 3H). ^{13}C NMR (75 MHz, $CDCl_3$) δ 170.6 (C_q), 170.0 (C_q), 169.9 (C_q), 169.7 (C_q), 72.7 (CH), 72.6 (CH), 68.6 (CH), 68.4 (CH), 66.9 (CH), 62.1 (CH_2), 60.2 (CH_2), 47.2 (C_q), 21.0 (CH_3), 20.9 (CH_3), 20.83 (CH_3), 20.77 (CH_3), 5.4 (C_q). HRMS (ESI) calcd for $C_{19}H_{25}INaO_9^+$ $[M+Na]^+$: 547.0435; Found: 547.0428.

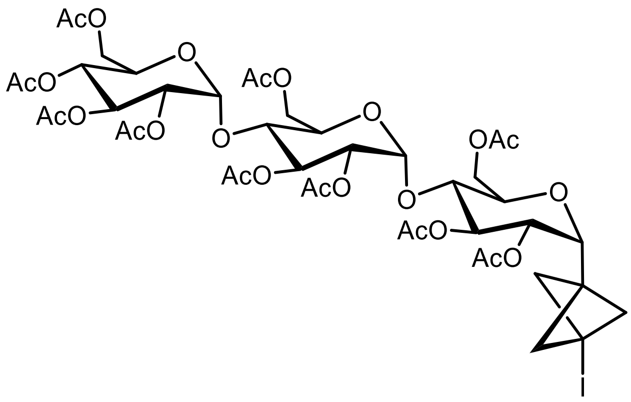

25, colorless syrup. 125.5 mg, 57% yield, >19:1 *α/β*. ^{1}H NMR (300 MHz, $CDCl_3$) δ 5.42–5.25 (m, 3H), 5.14 (d, *J* = 3.9 Hz, 1H), 5.07–4.98 (m, 2H), 4.94–4.87 (m, 1H), 4.83 (dd, *J* = 10.5, 4.0 Hz, 1H), 4.73 (dd, *J* = 10.3, 3.9 Hz, 1H), 4.42 (dd, *J* = 12.2, 2.3 Hz, 1H), 4.32–3.97 (m, 8H), 3.96–3.85 (m, 2H), 3.55 (dd, *J* = 7.4, 4.2 Hz, 1H), 2.45–2.30 (m, 6H), 2.12 (s, 3H), 2.10 (s, 3H), 2.09 (s, 3H), 2.05 (s, 3H), 2.01–1.98 (m, 12H), 1.96 (s, 3H), 1.94 (s, 3H). ^{13}C NMR (75 MHz, $CDCl_3$) δ 170.55 (C_q), 170.52 (C_q), 170.42 (C_q), 170.39 (C_q), 170.0 (C_q), 169.9 (C_q), 169.8 (C_q), 169.6 (C_q), 169.4 (C_q), 96.6 (CH), 95.9 (CH), 77.4 (CH), 74.9 (CH), 72.9 (CH), 72.0 (CH), 71.8 (CH), 70.4 (CH), 70.1 (CH), 70.0 (CH), 69.6 (CH), 69.4 (CH), 69.1 (CH), 68.8 (CH), 68.6 (CH), 67.9 (CH), 62.9 (CH_2), 62.5 (CH_2), 61.4 (CH_2), 60.3 (CH_2), 47.0 (C_q), 20.91 (CH_3), 20.88 (CH_3), 20.8 (CH_3), 20.7 (CH_3), 20.64 (CH_3), 20.62 (CH_3), 20.5 (CH_3), 6.5 (C_q). HRMS (ESI) calcd for $C_{43}H_{57}INaO_{25}^{+}$ $[M+Na]^{+}$: 1123.2126; Found: 1123.2123.

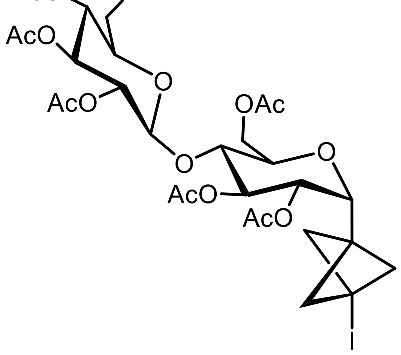

26, colorless syrup. 100.8 mg, 62% yield, >19:1 *α/β*. ^{1}H NMR (300 MHz, $CDCl_3$) δ 5.31 (dd, *J* = 8.3, 6.9 Hz, 1H), 5.16–4.99 (m, 2H), 4.96–4.84 (m, 2H), 4.52 (d, *J* = 7.9

Hz, 1H), 4.36–4.21 (m, 2H), 4.13–3.97 (m, 3H), 3.88 (ddd, *J* = 8.9, 6.4, 2.7 Hz, 1H), 3.66 (ddd, *J* = 9.8, 4.5, 2.3 Hz, 1H), 3.53 (dd, *J* = 8.4, 6.9 Hz, 1H), 2.45–2.31 (m, 6H), 2.08 (s, 3H), 2.05 (s, 3H), 2.03 (s, 3H), 2.01 (s, 6H), 1.98 (s, 3H), 1.95 (s, 3H). ^{13}C NMR (75 MHz, $CDCl_3$) δ 170.6 (C_q), 170.4 (C_q), 170.3 (C_q), 169.8 (C_q), 169.6 (C_q), 169.4 (C_q), 169.2 (C_q), 101.1 (CH), 76.7 (CH), 73.0 (CH), 72.1 (CH), 72.1 (CH), 71.6 (CH), 70.4 (CH), 69.9 (CH), 68.8 (CH), 67.9 (CH), 62.3 (CH_2), 61.7 (CH_2), 60.9 (CH_2), 47.2 (C_q), 20.9 (CH_3), 20.8 (CH_3), 20.74 (CH_3), 20.73 (CH_3), 20.64 (CH_3), 20.63 (CH_3), 6.1 (C_q). HRMS (ESI) calcd for $C_{31}H_{41}INaO_{17}^+$ $[M+Na]^+$: 835.1281; Found: 835.1278.

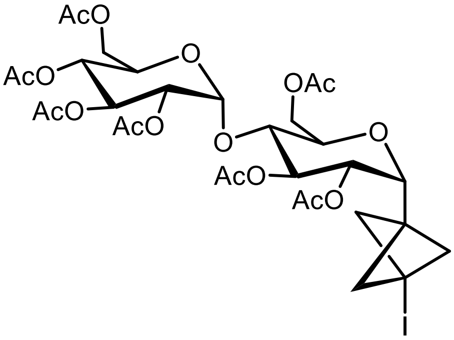

27, colorless syrup. 115.4 mg, 71% yield, 13:1 *α/β*. ^{1}H NMR (300 MHz, $CDCl_3$) δ 5.38–5.23 (m, 2H), 5.11–4.96 (m, 2H), 4.90–4.79 (m, 2H), 4.29–4.13 (m, 3H), 4.09–3.99 (m, 4H), 3.59 (dd, *J* = 7.1, 5.1 Hz, 1H), 2.42–2.29 (m, 6H), 2.09 (s, 3H), 2.07 (s, 3H), 2.05 (s, 3H), 2.01 (s, 3H), 2.00 (s, 3H), 1.99 (s, 3H), 1.96 (s, 3H). ^{13}C NMR (75 MHz, $CDCl_3$) δ 170.6 (C_q), 170.5 (C_q), 170.3 (C_q), 170.0 (C_q), 169.9 (C_q), 169.5 (C_q), 169.4 (C_q), 96.6 (CH), 77.4 (CH), 74.0 (CH), 72.1 (CH), 70.2 (CH), 70.0(CH), 69.5 (CH), 69.4 (CH), 69.3 (CH), 68.5 (CH), 68.2 (CH), 62.5 (CH_2), 61.7 (CH_2), 60.4 (CH_2), 47.0 (C_q), 20.91 (CH_3), 20.87 (CH_3), 20.8 (CH_3), 20.69 (CH_3), 20.65 (CH_3), 20.63 (CH_3), 20.56 (CH_3), 6.3 (C_q). HRMS (ESI) calcd for $C_{31}H_{41}INaO_{17}^+$ $[M+Na]^+$: 835.1281; Found: 835.1279.

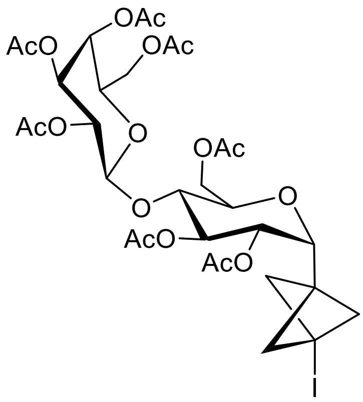

28, colorless syrup. 112.1 mg, 62% yield, >19:1 *α/β*. ^{1}H NMR (300 MHz, $CDCl_3$) δ 5.31–5.24 (m, 2H), 5.04 (dd, *J* = 10.4, 7.8 Hz, 1H), 4.93–4.83 (m, 2H), 4.45 (d, *J* = 7.9 Hz, 1H), 4.19 (dd, *J* = 11.8, 2.9 Hz, 1H), 4.13–3.98 (m, 4H), 3.89–3.80 (m, 2H), 3.55–3.48 (m, 1H), 2.41–2.26 (m, 6H), 2.08 (s, 3H), 2.04 (s, 3H), 2.01 (s, 3H), 1.99 (s, 3H), 1.98 (s, 3H), 1.98 (s, 3H), 1.90 (s, 3H). ^{13}C NMR (75 MHz, $CDCl_3$) δ 170.4 (C_q), 170.3 (C_q), 170.1 (C_q), 170.0 (C_q), 169.7 (C_q), 169.3 (C_q), 169.1 (C_q), 101.3 (CH), 76.1 (CH), 72.1 (CH), 70.9 (CH), 70.7 (CH), 70.0 (CH), 69.7 (CH), 69.0 (CH), 68.9 (CH), 66.7 (CH), 62.2 (CH_2), 60.9 (CH_2), 60.7 (CH_2), 47.1 (C_q), 20.8 (CH_3), 20.8 (CH_3), 20.6 (CH_3), 20.6 (CH_3), 20.6 (CH_3), 20.5 (CH_3), 6.1 (C_q). HRMS (ESI) calcd for $C_{31}H_{41}INaO_{17}^+$ $[M+Na]^+$: 835.1281; Found: 835.1280.

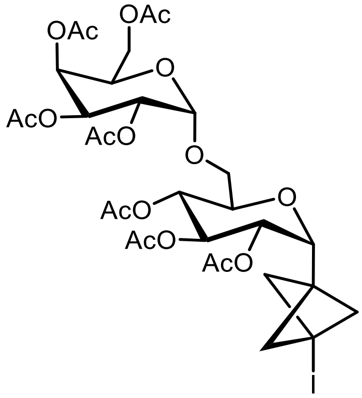

29, colorless syrup. 117.0 mg, 72% yield, >19:1 *α/β*. ^{1}H NMR (300 MHz, $CDCl_3$) δ 5.40 (dd, *J* = 3.4, 1.3 Hz, 1H), 5.35–5.24 (m, 2H), 5.06 (d, *J* = 8.7 Hz, 2H), 4.96 (dd, *J* = 9.8, 6.1 Hz, 1H), 4.89–4.80 (m, 1H), 4.19 (t, *J* = 6.4 Hz, 1H), 4.12–3.88 (m, 4H), 3.59 (dd, *J* = 11.1, 6.1 Hz, 1H), 3.43 (dd, *J* = 11.1, 2.7 Hz, 1H), 2.49 (dd, *J* = 9.3, 1.9 Hz,

3H), 2.39 (dd, J = 9.3, 1.9 Hz, 3H), 2.09 (s, 3H), 2.08 (s, 3H), 2.01 (s, 3H), 2.00 (s, 3H), 2.00 (s, 3H), 1.97 (s, 3H), 1.95 (s, 3H). ^{13}C NMR (75 MHz, $CDCl_3$) δ 170.5, 170.4, 170.2, 170.0, 169.9, 169.5, 169.4, 96.1, 77.4, 71.7, 71.0, 70.7, 69.9, 69.0, 68.1, 68.06, 67.4, 66.4, 61.8, 61.3, 47.3, 20.9, 20.8, 20.71, 20.68, 20.66, 5.9. HRMS (ESI) calcd for $C_{31}H_{41}INaO_{17}^+$ [M+Na]$^+$: 835.1281; Found: 835.1276.

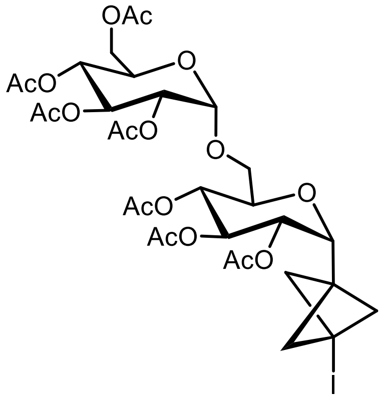

30, colorless syrup. 104.0 mg, 64% yield, >19:1 *α/β*. ^{1}H NMR (300 MHz, $CDCl_3$) δ 5.43 (t, J = 9.8 Hz, 1H), 5.31 (t, J = 9.2 Hz, 1H), 5.05–4.93 (m, 3H), 4.87–4.78 (m, 2H), 4.19–4.00 (m, 5H), 3.60 (dd, J = 11.0, 6.4 Hz, 1H), 3.46 (dd, J = 11.0, 2.7 Hz, 1H), 2.54–2.37 (m, 6H), 2.08 (s, 3H), 2.05 (s, 3H), 2.03 (s, 3H), 2.01 (s, 3H), 2.00 (s, 3H), 1.99 (s, 3H), 1.98 (s, 3H). ^{13}C NMR (75 MHz, $CDCl_3$) δ 170.7 (C_q), 170.3 (C_q), 169.99 (C_q), 169.97 (C_q), 169.7 (C_q), 169.6 (C_q), 169.5 (C_q), 95.8 (CH), 71.7 (CH), 71.0 (CH), 70.74 (CH), 70.71 (CH), 69.95 (CH), 69.89 (CH), 69.1 (CH), 68.5 (CH), 67.4 (CH), 66.8 (CH_2), 61.9 (CH_2), 61.4 (CH_2), 60.4 (CH_2), 47.3 (C_q), 20.84 (CH_3), 20.80 (CH_3), 20.76 (CH_3), 20.74 (CH_3), 20.71 (CH_3), 20.66 (CH_3), 20.6 (CH_3), 5.9 (C_q). HRMS (ESI) calcd for $C_{31}H_{41}INaO_{17}^+$ [M+Na]$^+$: 835.1281; Found: 835.1278.

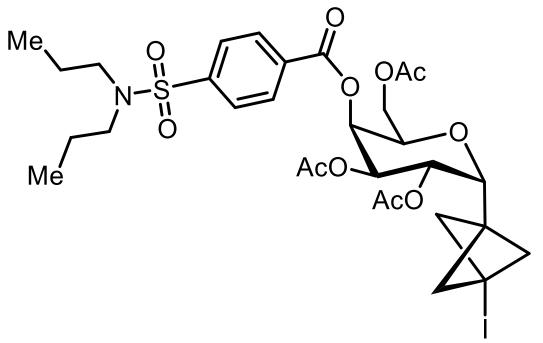

31, colorless syrup. 100.4 mg, 67% yield, >19:1 *α/β*. ^{1}H NMR (300 MHz, $CDCl_3$) δ 8.10 (d, *J* = 8.5 Hz, 2H), 7.87 (d, *J* = 8.5 Hz, 2H), 5.62 (s, 1H), 5.36–5.24 (m, 2H), 4.35–4.19 (m, 3H), 4.01 (q, *J* = 8.2 Hz, 1H), 3.12–3.01 (m, 4H), 2.50–2.33 (m, 6H), 2.06 (s, 3H), 2.01 (s, 3H), 1.98 (s, 3H), 1.61–1.45 (m, 4H), 0.85 (t, *J* = 7.4 Hz, 6H). ^{13}C NMR (75 MHz, $CDCl_3$) δ 170.5 (C_q), 169.8 (C_q), 169.7 (C_q), 164.3 (C_q), 144.9 (C_q), 132.2 (C_q), 130.5 (CH), 127.3 (CH), 70.6 (CH), 70.0 (CH), 68.8 (CH), 68.3 (CH), 67.4 (CH), 61.7 (CH_2), 61.1 (CH_2), 50.2 (CH_2), 47.1 (C_q), 22.1 (CH_2), 20.9 (CH_3), 20.7 (CH_3), 11.2 (CH_3), 5.7 (C_q). HRMS (ESI) calcd for $C_{30}H_{40}INNaO_{10}S^+$ $[M+Na]^+$: 772.1259; Found: 772.1252.

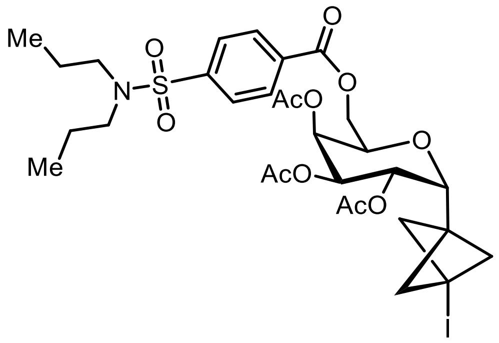

32, colorless syrup. 112.4 mg, 75% yield, >19:1 *α/β*. ^{1}H NMR (300 MHz, $CDCl_3$) δ 8.10 (d, *J* = 8.1 Hz, 2H), 7.86 (d, *J* = 8.2 Hz, 2H), 5.44 (t, *J* = 2.8 Hz, 1H), 5.29–5.15 (m, 2H), 4.66 (dd, *J* = 11.5, 7.9 Hz, 1H), 4.37–4.11 (m, 3H), 3.14–3.03 (m, 4H), 2.42–2.22 (m, 6H), 2.10 (s, 3H), 2.07 (s, 3H), 2.03 (s, 3H), 1.60–1.44 (m, 4H), 0.85 (t, *J* = 7.4 Hz, 6H). ^{13}C NMR (75 MHz, $CDCl_3$) δ 170.0 (C_q), 169.7 (C_q), 164.9 (C_q), 144.7 (C_q), 132.8 (C_q), 130.4 (CH), 127.2 (CH), 70.2 (CH), 69.9 (CH), 68.3 (CH), 67.23 (CH), 67.2 (CH), 62.1 (CH_2), 60.9 (CH_2), 50.0 (CH_2), 47.1 (C_q), 22.0 (CH_2), 20.9 (CH_3), 20.8 (CH_3), 20.7 (CH_3), 11.2 (CH_2), 5.8 (C_q). HRMS (ESI) calcd for $C_{30}H_{40}INNaO_{10}S^+$ $[M+Na]^+$: 772.1259; Found: 772.1251.

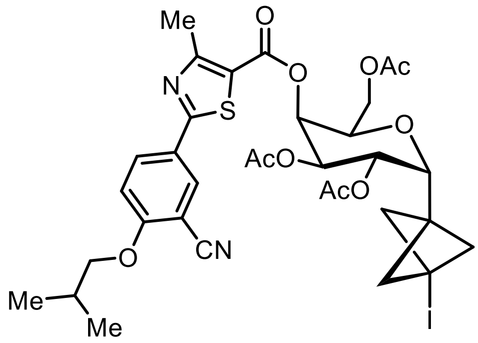

33, colorless syrup. 120.2 mg, 77% yield, >19:1 *α/β*. ^{1}H NMR (300 MHz, $CDCl_3$) δ 8.15 (d, *J* = 2.3 Hz, 1H), 8.04 (dd, *J* = 8.8, 2.3 Hz, 1H), 6.98 (d, *J* = 8.9 Hz, 1H), 5.54 (t, *J* = 2.7 Hz, 1H), 5.33–5.18 (m, 2H), 4.40–4.20 (m, 3H), 4.01 (dd, *J* = 11.2, 4.3 Hz, 1H), 3.86 (d, *J* = 6.5 Hz, 2H), 2.70 (s, 3H), 2.47–2.30 (m, 6H), 2.15 (p, *J* = 6.6 Hz, 1H), 2.06 (s, 3H), 2.03 (s, 3H), 2.01 (s, 3H), 1.04 (d, *J* = 6.7 Hz, 6H). ^{13}C NMR (75 MHz, $CDCl_3$) δ 170.5 (C_q), 169.7 (C_q), 169.6 (C_q), 168.2 (C_q), 162.6 (C_q), 162.4 (C_q), 160.8 (C_q), 132.7 (CH), 132.1 (CH), 125.7 (C_q), 120.3 (C_q), 115.2 (C_q), 112.7 (CH), 103.0 (C_q), 75.7 (CH_2), 70.2 (CH), 70.0 (CH), 68.3 (CH), 67.3 (CH), 61.5 (CH_2), 60.9 (CH_2), 47.0 (C_q), 28.1 (CH), 20.83 (CH_3), 20.77 (CH_3), 20.7 (CH_3), 19.0 (CH_3), 17.6 (CH_3), 5.8 (C_q). HRMS (ESI) calcd for $C_{33}H_{37}IN_2NaO_{10}S^+$ $[M+Na]^+$: 781.1286; Found: 781.1274.

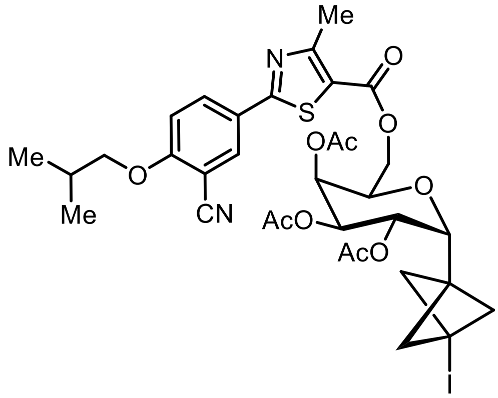

34, colorless syrup. 112.4 mg, 72% yield, >19:1 *α/β*. ^{1}H NMR (300 MHz, $CDCl_3$) δ 8.15 (d, *J* = 2.3 Hz, 1H), 8.05 (dd, *J* = 8.8, 2.3 Hz, 1H), 6.99 (d, *J* = 8.9 Hz, 1H), 5.41 (t, *J* = 3.1 Hz, 1H), 5.29–5.14 (m, 2H), 4.61 (dd, *J* = 11.6, 8.2 Hz, 1H), 4.26 (dd, *J* = 13.3, 3.9 Hz, 2H), 4.13 (dd, *J* = 11.6, 4.6 Hz, 1H), 3.87 (d, *J* = 6.5 Hz, 2H), 2.72 (s, 3H),

2.43–2.30 (m, 6H), 2.22–2.09 (m, 1H), 2.10 (s, 3H), 2.07 (s, 3H), 2.04 (s, 3H), 1.06 (d, J = 6.7 Hz, 6H). ^{13}C NMR (75 MHz, $CDCl_3$) δ 169.9 (C_q), 169.64 (C_q), 169.6 (C_q), 167.8 (C_q), 162.6 (C_q), 162.1 (C_q), 161.5 (C_q), 132.7 (CH), 132.2 (CH), 125.8 (C_q), 120.7 (C_q), 115.4 (C_q), 112.7 (CH), 103.0 (C_q), 75.7 (CH_2), 70.3 (CH), 69.7 (CH), 68.3 (CH), 67.2 (CH), 67.1 (CH), 61.7 (CH_2), 60.8 (CH_2), 47.0 (C_q), 28.2 (CH), 20.9 (CH_3), 20.8 (CH_3), 20.7 (CH_3), 19.1 (CH_3), 17.6 (CH_3), 5.9 (C_q). HRMS (ESI) calcd for $C_{33}H_{37}IN_2NaO_{10}S^+$ [M+Na]$^+$: 781.1286; Found: 781.1277.

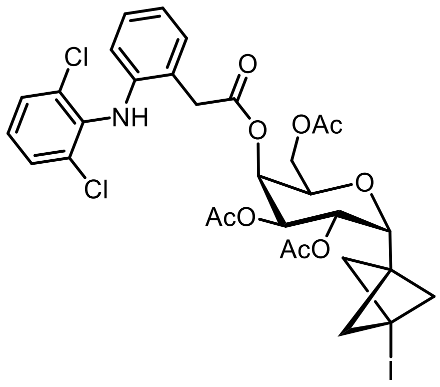

35, colorless syrup. 100.4 mg, 66% yield, >19:1 *α/β*. ^{1}H NMR (300 MHz, $CDCl_3$) δ 7.33 (d, J = 8.1 Hz, 2H), 7.29–7.18 (m, 1H), 7.10 (td, J = 7.7, 1.6 Hz, 1H), 7.03–6.90 (m, 2H), 6.75 (s, 1H), 6.53 (d, J = 8.0 Hz, 1H), 5.44 (t, J = 2.7 Hz, 1H), 5.33–5.16 (m, 2H), 4.29–4.16 (m, 3H), 3.98–3.77 (m, 3H), 2.46–2.33 (m, 6H), 2.06 (s, 3H), 2.01 (s, 3H), 1.76 (s, 3H). ^{13}C NMR (75 MHz, $CDCl_3$) δ 171.8 (C_q), 170.5 (C_q), 169.8 (C_q), 169.6 (C_q), 142.7 (C_q), 137.9 (C_q), 131.0 (CH), 129.6 (C_q), 129.0 (CH), 128.2 (CH), 124.2 (CH), 124.1 (C_q), 122.4 (CH), 118.6 (CH), 70.5 (CH), 69.9 (CH), 68.3 (CH), 68.2 (CH), 67.2 (CH), 61.4 (CH_2), 61.1 (CH_2), 47.2 (C_q), 38.1 (CH_2), 20.9 (CH_3), 20.8 (CH_3), 20.4 (CH_3), 5.8 (C_q). HRMS (ESI) calcd for $C_{31}H_{32}ICl_2NNaO_9^+$ [M+Na]$^+$: 782.0391; Found: 782.0389.

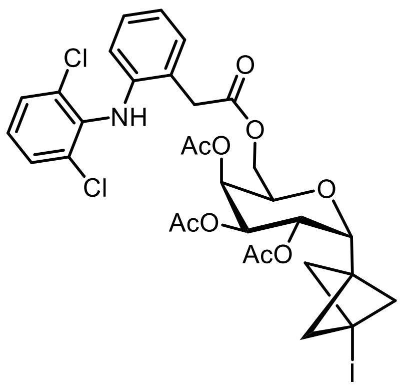

36, colorless syrup. 118.6 mg, 78% yield, >19:1 *α/β*. ^{1}H NMR (300 MHz, $CDCl_3$) δ 7.34 (d, *J* = 8.1 Hz, 2H), 7.24–7.11 (m, 2H), 7.02–6.94 (m, 2H), 6.75 (s, 1H), 6.55 (d, *J* = 7.9 Hz, 1H), 5.38 (t, *J* = 2.7 Hz, 1H), 5.23–5.15 (m, 2H), 4.39 (dd, *J* = 11.6, 8.2 Hz, 1H), 4.22–4.15 (m, 2H), 4.06 (dd, *J* = 11.6, 4.0 Hz, 1H), 3.82 (s, 2H), 2.31–2.20 (m, 6H), 2.08 (s, 3H), 2.06 (s, 3H), 2.02 (s, 3H). ^{13}C NMR (75 MHz, $CDCl_3$) δ 171.9 (C_q), 169.9 (C_q), 169.7 (C_q), 169.6 (C_q), 142.7 (C_q), 137.7 (C_q), 130.9 (CH), 129.6 (C_q), 129.0 (CH), 128.3 (CH), 124.3 (CH), 123.9 (C_q), 122.3 (CH), 118.4 (CH), 70.3 (CH), 69.9 (CH), 68.3 (CH), 67.4 (CH), 67.2 (CH), 62.4 (CH_2), 60.8 (CH_2), 46.9 (C_q), 38.4 (CH_2), 20.9 (CH_3), 20.8 (CH_3), 20.7 (CH_3), 6.0 (C_q). HRMS (ESI) calcd for $C_{31}H_{32}ICl_2NNaO_9^+$ $[M+Na]^+$: 782.0391; Found: 782.0391.

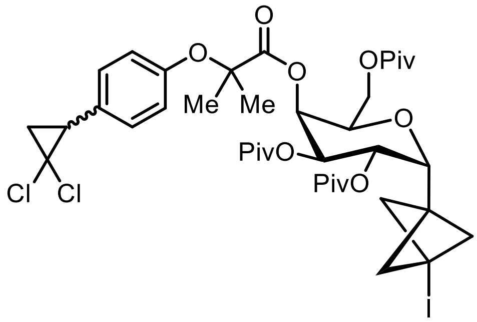

37, colorless syrup. 131.9 mg, 75% yield, >19:1 *α/β*. ^{1}H NMR (300 MHz, $CDCl_3$) δ 7.09 (d, *J* = 8.2 Hz, 2H), 6.80 (dd, *J* = 8.6, 1.6 Hz, 2H), 5.41 (t, *J* = 3.4 Hz, 1H), 5.24 (dd, *J* = 8.1, 3.1 Hz, 1H), 5.14 (dd, *J* = 8.2, 4.1 Hz, 1H), 4.51–4.35 (m, 1H), 4.15–4.03 (m, 2H), 3.54 (dd, *J* = 11.8, 4.4 Hz, 1H), 2.79 (dd, *J* = 10.6, 8.4 Hz, 1H), 2.39–2.29 (m, 6H), 1.96–1.83 (m, 1H), 1.78–1.71 (m, 1H), 1.60 (s, 3H), 1.58 (s, 3H), 1.21 (s, 9H),

1.18 (s, 9H), 1.15 (d, J = 1.3 Hz, 9H). ^{13}C NMR (75 MHz, $CDCl_3$) δ 178.1 (C_q), 177.1 (C_q), 177.0 (C_q), 172.9 (C_q), *154.74 (C_q), 154.72 (C_q) (two signals due to diastereomers)*, *129.9 (CH)*, *129.8 (CH) (two signals due to diastereomers)*, *128.61 (C_q)*, *128.56 (C_q) (two signals due to diastereomers)*, *119.0 (CH)*, *118.9 (CH) (two signals due to diastereomers)*, 79.3 (CH), 70.9 (CH), 69.4 (CH), *68.33 (CH)*, *68.31 (CH) (two signals due to diastereomers)*, 68.2 (CH), 67.4 (CH), 60.8 (CH_2), 60.3 (C_q), 47.0 (C_q), 39.0 (C_q), 38.8 (C_q), 38.84 (C_q), 34.77 (CH), 27.4 (CH_3), 27.3 (CH_3), 27.2 (CH_3), *26.30 (CH_3)*, *26.26 (CH_3) (two signals due to diastereomers)*, *25.89 (CH_2)*, *25.84 (CH_2) (two signals due to diastereomers)*, *25.02 (CH_3)*, *24.99 (CH_3) (two signals due to diastereomers)*, 6.0 (C_q). HRMS (ESI) calcd for $C_{39}H_{53}ICl_2NaO_{10}^+$ [M+Na]$^+$: 901.1953; Found: 901.1955.

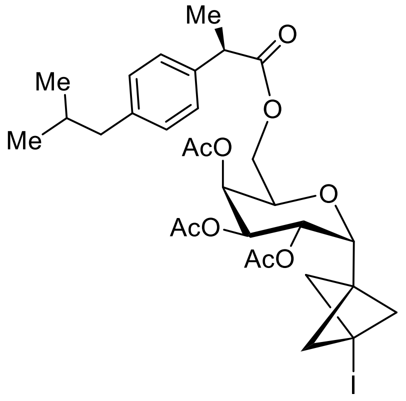

38, colorless syrup. 105.9 mg, 79% yield, >19:1 *α/β*. ^{1}H NMR (300 MHz, $CDCl_3$) δ 7.16 (d, J = 8.1 Hz, 2H), 7.07 (d, J = 8.1 Hz, 2H), 5.31 (d, J = 2.6 Hz, 1H), 5.18 (t, J = 2.4 Hz, 2H), 4.17 (d, J = 3.9 Hz, 1H), 4.15–3.97 (m, 2H), 3.72 (q, J = 7.0 Hz, 1H), 3.47 (dd, J = 11.1, 4.2 Hz, 1H), 2.42 (d, J = 7.3 Hz, 2H), 2.40–2.25 (m, 6H), 2.06 (s, 3H), 1.97 (s, 3H), 1.91 (s, 3H), 1.49 (d, J = 7.2 Hz, 3H), 0.85 (d, J = 6.6 Hz, 6H). ^{13}C NMR (75 MHz, $CDCl_3$) δ 173.7 (C_q), 170.4 (C_q), 169.69 (C_q), 169.65 (C_q), 141.0 (C_q), 137.1 (C_q), 129.5 (CH), 127.2 (CH), 70.1 (CH), 68.3 (CH), 67.4 (CH), 67.3 (CH), 61.0 (CH_2), 60.96 (CH_2), 47.1 (C_q), 45.2 (CH), 45.0 (CH_2), 30.2 (CH), 22.4 (CH_3), 22.3 (CH_3), 20.9 (CH_3), 20.72 (CH_3), 20.67 (CH_3), 18.0 (CH_3), 5.9 (C_q). HRMS (ESI) calcd for $C_{30}H_{39}INaO_9^+$ [M+Na]$^+$: 693.1531; Found: 693.1530.

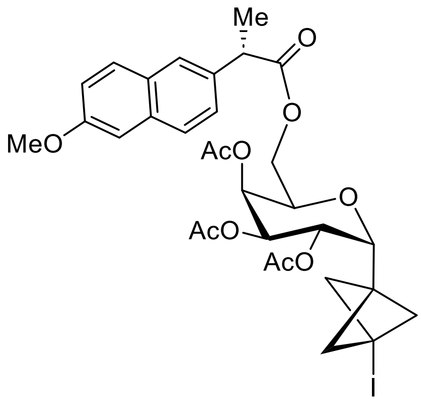

39, colorless syrup. 91.6 mg, 66% yield, >19:1 *α/β*. ^{1}H NMR (300 MHz, $CDCl_3$) δ 7.74–7.63 (m, 3H), 7.38 (dd, *J* = 8.4, 1.9 Hz, 1H), 7.18–7.10 (m, 2H), 5.34–5.30 (m, 1H), 5.17–5.13 (m, 2H), 4.36 (dd, *J* = 11.6, 8.0 Hz, 1H), 4.13–4.05 (m, 2H), 4.00–3.81 (m, 5H), 2.24–2.11 (m, 6H), 2.07 (s, 3H), 2.05 (s, 3H), 2.01 (s, 3H), 1.57 (d, *J* = 7.1 Hz, 3H). ^{13}C NMR (75 MHz, $CDCl_3$) δ 174.4 (C_q), 169.9 (C_q), 169.7 (C_q), 169.6 (C_q), 157.8 (C_q), 135.2 (C_q), 133.8 (C_q), 129.3 (CH), 129.0 (C_q), 127.4 (CH), 126.2 (CH), 126.1 (CH), 119.3 (CH), 105.8 (CH), 70.3 (CH), 69.7 (CH), 68.4 (CH), 67.2 (CH), 61.4 (CH_2), 60.8 (CH_2), 55.4 (CH_3), 46.9 (C_q), 45.4 (CH), 20.9 (CH_3), 20.8 (CH_3), 20.7 (CH_3), 18.4 (CH_3), 6.0 (C_q). HRMS (ESI) calcd for $C_{31}H_{35}INaO_{10}^+$ $[M+Na]^+$: 717.1167; Found: 717.1167.

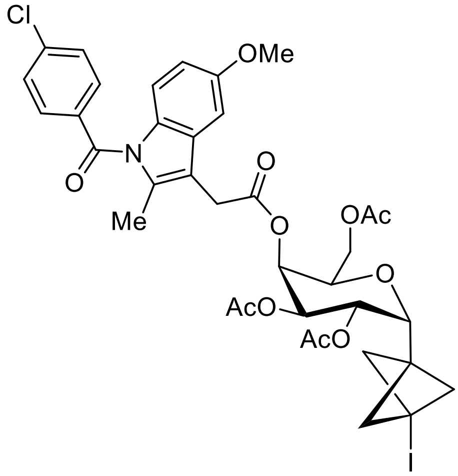

40, colorless syrup. 126.6 mg, 77% yield, >19:1 *α/β*. ^{1}H NMR (300 MHz, $CDCl_3$) δ 7.65 (d, *J* = 8.6 Hz, 2H), 7.44 (d, *J* = 8.5 Hz, 2H), 6.93 (d, *J* = 2.5 Hz, 1H), 6.85 (d, *J* = 9.0 Hz, 1H), 6.64 (dd, *J* = 9.0, 2.5 Hz, 1H), 5.36 (t, *J* = 2.8 Hz, 1H), 5.23–5.04 (m, 2H),

4.22–4.10 (m, 3H), 3.87–3.77 (m, 1H), 3.80 (s, 3H), 3.70 (s, 2H), 2.41–2.29 (m, 6H), 2.37 (s, 3H), 2.04 (s, 3H), 1.99 (s, 3H), 1.77 (s, 3H). ^{13}C NMR (75 MHz, $CDCl_3$) δ 170.4, 169.8, 169.6, 169.5, 168.3, 156.1, 139.3, 136.1, 133.9, 131.3, 130.8, 130.3, 129.1, 115.1, 112.0, 111.8, 101.0, 70.1, 68.3, 67.7, 67.2, 61.0, 60.9, 55.7, 47.0, 30.1, 20.9, 20.7, 20.4, 13.3, 5.8. HRMS (ESI) calcd for $C_{36}H_{37}ClINNaO_{11}^+$ [M+Na]$^+$: 844.0992; Found: 844.0986.

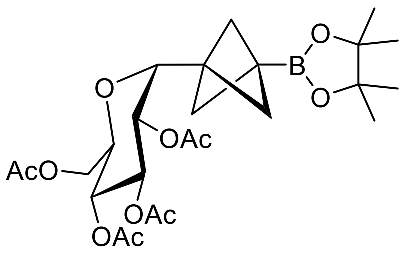

41, colorless syrup. 70.3 mg, 67% yield. >19:1 *α/β* (The *α*-configuration was confirmed by NOESY NMR analysis). ^{1}H NMR (400 MHz, $CDCl_3$) δ 5.41 (t, *J* = 9.5 Hz, 1H), 4.96 (dd, *J* = 9.9, 6.3 Hz, 1H), 4.85 (t, *J* = 9.2 Hz, 1H), 4.12–3.95 (m, 3H), 3.90 (d, *J* = 6.2 Hz, 1H), 2.06 (dd, *J* = 9.5, 2.0 Hz, 3H), 2.01 (s, 3H), 1.97–1.92 (m, 3H), 1.96 (s, 3H), 1.941 (s, 3H), 1.938 (s, 3H), 1.16 (s, 12H). ^{13}C NMR (100 MHz, $CDCl_3$) δ 170.6 (C_q), 169.9 (C_q), 169.7 (C_q), 169.5 (C_q), 83.5 (C_q), 71.2 (CH), 71.1 (CH), 70.5 (CH), 70.1 (CH), 69.0 (CH), 62.6 (CH_2), 53.2 (CH_2), 49.9 (C_q), 44.8 (C_q), 24.7 (CH_3), 20.71 (CH_3), 20.69 (CH_3), 20.65 (CH_3), 20.60 (CH_3). ^{11}B NMR (161 MHz, $CDCl_3$) δ 30.11. HRMS (ESI) calcd for $C_{25}H_{37}NaBO_{11}^+$ [M+Na]$^+$: 547.2324; Found: 547.2322.

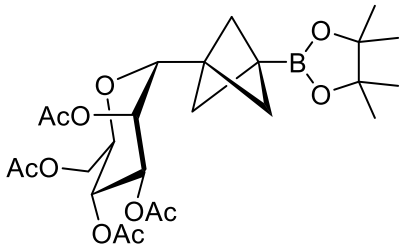

42, colorless syrup. 88.0 mg, 84% yield. >19:1 *α/β*. ^{1}H NMR (400 MHz, $CDCl_3$) δ 5.29–5.21 (m, 2H), 5.15 (t, *J* = 8.8 Hz, 1H), 4.27–4.19 (m, 1H), 4.10–4.00 (m, 2H), 3.73 (d, *J* = 2.5 Hz, 1H), 2.09 (s, 3H), 2.08 (s, 3H), 2.07–1.95 (m, 6H), 2.03 (s, 3H),

1.99 (s, 3H), 1.22 (s, 13H). ^{13}C NMR (100 MHz, $CDCl_3$) δ 170.8 (C_q), 170.3 (C_q), 170.1 (C_q), 169.8 (C_q), 83.7 (C_q), 75.2 (CH), 72.0 (CH), 69.4 (CH), 68.9 (CH), 66.8 (CH), 63.0 (CH_2), 52.2 (CH_2), 44.5 (C_q), 24.9 (CH_3), 21.1 (CH_3), 20.87 (CH_3), 20.86 (CH_3), 20.8 (CH_3). ^{11}B NMR (161 MHz, $CDCl_3$) δ 30.1. HRMS (ESI) calcd for $C_{25}H_{37}NaBO_{11}^+$ $[M+Na]^+$: 547.2324; Found: 547.2325.

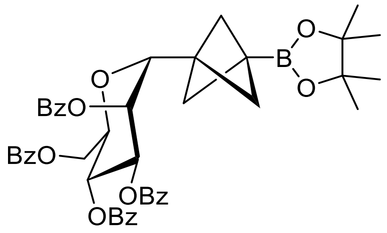

43, white foam. 93.9 mg, 65% yield. >19:1 *α/β*. ^1H NMR (300 MHz, $CDCl_3$) δ 8.12–7.82 (m, 8H), 7.62–7.24 (m, 12H), 6.05–5.94 (m, 1H), 5.84 (dd, *J* = 9.4, 3.2 Hz, 1H), 5.76 (t, *J* = 2.9 Hz, 1H), 4.68–4.49 (m, 3H), 4.09 (d, *J* = 2.5 Hz, 1H), 2.24 (dd, *J* = 9.6, 2.0 Hz, 3H), 2.16 (dd, *J* = 9.6, 2.0 Hz, 3H), 1.26 (s, 12H). ^{13}C NMR (75 MHz, $CDCl_3$) δ 166.4 (C_q), 165.9 (C_q), 165.7 (C_q), 165.5 (C_q), 133.6 (CH), 133.41 (CH), 133.37 (CH), 133.1 (CH), 129.99 (C_q), 129.89 (CH), 129.88 (CH), 129.85 (CH), 129.7 (C_q), 129.2 (CH), 128.64 (CH), 128.58 (CH), 128.54 (CH), 128.49 (CH), 83.7 (C_q), 75.5 (CH), 72.2 (CH), 70.5 (CH), 70.1 (CH), 67.4 (CH), 63.4 (CH_2), 52.5 (CH_2), 44.7 (C_q), 24.9 (C_q). ^{11}B NMR (161 MHz, $CDCl_3$) δ 30.4. HRMS (ESI) calcd for $C_{45}H_{45}NaBO_{11}^+$ $[M+Na]^+$: 795.2954; Found: 795.2941.

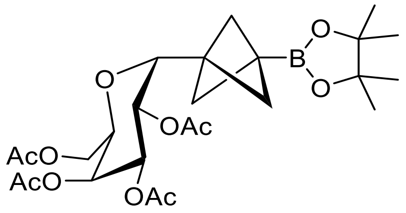

44, colorless syrup. 84.9 mg, 81% yield. >19:1 *α/β* (The *α*-configuration was confirmed by NOESY NMR analysis). ^1H NMR (400 MHz, $CDCl_3$) δ 5.39–5.31 (m, 2H), 5.24 (dd, *J* = 9.7, 5.7 Hz, 1H), 4.29–4.23 (m, 1H), 4.15 (dd, *J* = 11.4, 7.5 Hz, 1H), 3.99 (dd, *J* = 10.5, 5.7 Hz, 2H), 2.10 (s, 3H), 2.08–2.03 (m, 9H), 2.00 (s, 3H), 1.96 (dd, *J* = 9.6, 2.0

Hz, 3H), 1.22 (s, 12H). ^{13}C NMR (100 MHz, $CDCl_3$) δ 170.7 (C_q), 170.3 (C_q), 170.1 (C_q), 170.0 (C_q), 83.6 (C_q), 71.0 (CH), 69.6 (CH), 68.8(CH), 68.1 (CH), 67.7 (CH), 62.1 (CH_2), 53.1 (CH_2), 44.9 (C_q), 24.9 (CH_2), 21.0 (CH_2), 20.9 (CH_2), 20.82 (CH_2), 20.77 (CH_2). ^{11}B NMR (161 MHz, $CDCl_3$) δ 30.0. HRMS (ESI) calcd for $C_{25}H_{37}NaBO_{11}^+$ $[M+Na]^+$: 547.2324; Found: 547.2325.

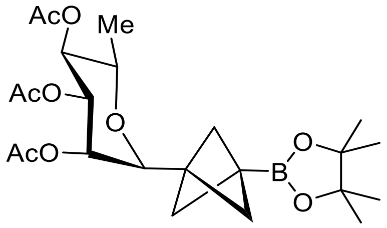

45, colorless syrup. 55.9 mg, 60% yield. >19:1 *α/β* (The *α*-configuration was confirmed by NOESY NMR analysis). ^{1}H NMR (400 MHz, $CDCl_3$) δ 5.29 (dd, *J* = 3.4, 2.3 Hz, 1H), 5.19 (dd, *J* = 9.6, 3.4 Hz, 1H), 5.01 (t, *J* = 9.3 Hz, 1H), 3.97–3.88 (m, 1H), 3.66 (d, *J* = 2.3 Hz, 1H), 2.11 (s, 3H), 2.06 (dd, *J* = 9.6, 2.0 Hz, 3H), 2.04 (s, 3H), 1.99 (s, 3H), 1.98 (dd, *J* = 9.6, 2.1 Hz, 3H), 1.22 (s, 12H), 1.18 (d, *J* = 6.2 Hz, 3H). ^{13}C NMR (100 MHz, $CDCl_3$) δ 170.5 (C_q), 170.3 (C_q), 170.1 (C_q), 83.6 (C_q), 71.3 (CH), 69.8 (CH), 69.7 (CH), 69.3 (CH), 52.4 (CH_2), 44.8 (C_q), 24.9 (CH_3), 21.2 (CH_3), 21.0 (CH_3), 20.9 (CH_3), 17.9 (CH_3). ^{11}B NMR (161 MHz, $CDCl_3$) δ 30.3. HRMS (ESI) calcd for $C_{23}H_{35}BO_9^+$ $[M+Na]^+$: 489.2268; Found: 489.2267.

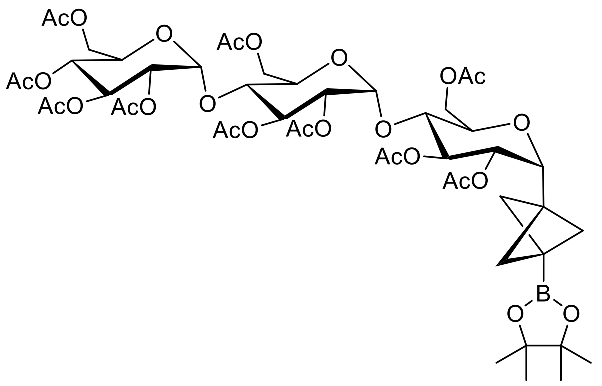

46, colorless syrup. 103.5 mg, 47% yield. >19:1 *α/β*. ^{1}H NMR (300 MHz, $CDCl_3$) δ 5.46–4.71 (m, 9H), 4.46 (dd, *J* = 12.3, 2.4 Hz, 1H), 4.32–3.82 (m, 10H), 3.65 (dd, *J* =

8.2, 5.7 Hz, 1H), 2.14 (s, 3H), 2.14 (s, 3H), 2.12–1.96 (m, 30H), 1.24 (s, 12H). ^{13}C NMR (100 MHz, $CDCl_3$) δ 170.8 (C_q), 170.71 (C_q) , 170.68(C_q), 170.6 (C_q), 170.3 (C_q), 170.0 (C_q) , 169.83(C_q), 169.80 (C_q), 169.6 (C_q), 96.4 (CH_2), 95.9 (CH_2), 83.6 (C_q), 77.5 (CH), 77.2 (CH), 76.8 (CH), 75.0 (CH), 73.0 (CH), 72.0 (CH), 71.4 (CH), 71.3 (CH), 70.52 (CH), 70.50 (CH), 70.4 (CH), 70.2 (CH), 69.6 (CH), 68.9 (CH), 68.6 (CH), 68.0 (CH), 63.5 (CH_2), 62.6 (CH_2), 61.5 (CH_2), 52.4 (CH_2), 51.2 (C_q), 44.7 (C_q), 24.9 (CH_3), 21.02 (CH_3), 21.01 (CH_3), 20.9 (CH_3), 20.81 (CH_3), 20.75 (CH_3), 20.74 (CH_3), 20.67 (CH_3). ^{11}B NMR (161 MHz, $CDCl_3$) δ 31.6. HRMS (ESI) calcd for $C_{49}H_{69}BNaO_{27}^+$ $[M+Na]^+$: 1123.4026; Found: 1123.4031.

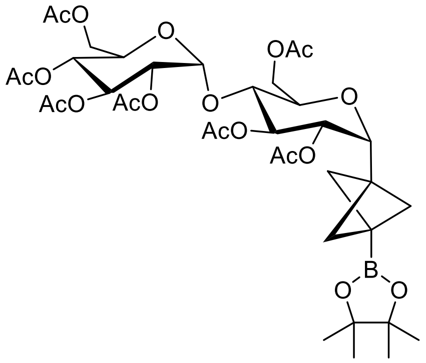

47, colorless syrup. 99.1 mg, 61% yield. >19:1 *α/β*. ^{1}H NMR (400 MHz, $CDCl_3$) δ 5.40–5.28 (m, 3H), 5.05 (t, J = 9.7 Hz, 1H), 4.96–4.85 (m, 2H), 4.31–4.20 (m, 3H), 4.16–4.10 (m, 1H), 4.10–4.04 (m, 2H), 3.85 (d, J = 5.3 Hz, 1H), 3.70 (dd, J = 7.9, 6.4 Hz, 1H), 2.12 (s, 3H), 2.09 (s, 3H), 2.08 (s, 3H), 2.07 (dd, J = 9.6, 1.9 Hz, 3H), 2.06 (s, 3H), 2.03 (s, 3H), 2.02 (s, 3H), 2.00 (s, 3H), 1.98 (dd, J = 9.6, 1.9 Hz, 3H), 1.24 (s, 12H). ^{13}C NMR (100 MHz, $CDCl_3$) δ 170.7 (C_q), 170.6 (C_q), 170.2 (C_q), 170.1 (C_q), 169.8 (C_q), 169.6 (C_q), 96.3 (CH), 83.7 (C_q), 74.1 (CH), 71.5 (CH), 70.5 (CH), 70.3 (CH), 70.1 (CH), 69.7 (CH), 68.5 (CH), 68.3 (CH), 63.1 (CH_2), 61.7 (CH_2), 52.5 (CH_2), 44.8 (C_q), 24.9 (CH_3), 21.1 (CH_3), 21.0 (CH_3), 20.83 (CH_3), 20.77 (CH_3), 20.75 (CH_3), 20.74 (CH_3). ^{11}B NMR (161 MHz, $CDCl_3$) δ 30.9. HRMS (ESI) calcd for $C_{37}H_{53}NaBO_{19}^+$ $[M+Na]^+$: 835.3172; Found: 835.3182.

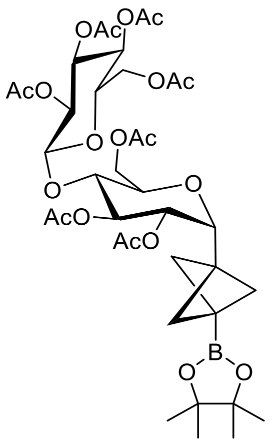

48, colorless syrup. 100.8 mg, 62% yield. >19:1 *α/β*. ^{1}H NMR (500 MHz, $CDCl_3$) δ 5.42 (dd, *J* = 9.4, 8.0 Hz, 1H), 5.12 (t, *J* = 9.4 Hz, 1H), 5.04 (t, *J* = 9.7 Hz, 1H), 4.95 (dd, *J* = 9.4, 6.0 Hz, 1H), 4.90 (dd, *J* = 9.4, 8.0 Hz, 1H), 4.48 (d, *J* = 8.0 Hz, 1H), 4.37–4.31 (m, 2H), 4.09–3.96 (m, 3H), 3.86 (d, *J* = 6.0 Hz, 1H), 3.64 (ddd, *J* = 9.9, 4.4, 2.3 Hz, 1H), 3.55 (dd, *J* = 9.2, 8.0 Hz, 1H), 2.10 (s, 3H), 2.08 (dd, *J* = 9.4, 2.0 Hz, 3H), 2.07 (s, 3H), 2.02 (s, 3H), 2.02 (s, 3H), 2.00 (s, 3H), 1.99 (s, 3H), 1.97 (dd, *J* = 9.4, 2.0 Hz, 3H), 1.96 (s, 3H), 1.22 (s, 12H). ^{13}C NMR (126 MHz, $CDCl_3$) δ 170.7 (C_q), 170.6 (C_q), 170.4 (C_q), 170.1 (C_q), 169.7 (C_q), 169.5 (C_q), 169.2 (C_q), 101.1 (CH), 83.6 (C_q), 73.1 (CH), 72.0 (CH), 71.7 (CH), 71.6 (CH), 71.0 (CH), 70.9 (CH), 69.5 (CH), 67.9 (CH), 62.7 (CH_2), 61.7 (CH_2), 53.0 (CH_2), 44.8 (C_q), 24.9 (CH_3), 21.0 (CH_3), 20.9 (CH_3), 20.79 (CH_3), 20.76 (CH_3), 20.7 (CH_3). ^{11}B NMR (161 MHz, $CDCl_3$) δ 30.9. HRMS (ESI) calcd for $C_{37}H_{53}NaBO_{19}^+$ $[M+Na]^+$: 835.3172; Found: 835.3174.

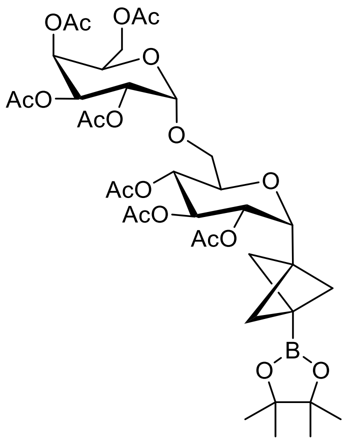

49, colorless syrup. 117.0 mg, 72% yield. >19:1 *α/β*. ^{1}H NMR (400 MHz, $CDCl_3$) δ

5.50–5.43 (m, 2H), 5.35–5.29 (m, 1H), 5.13–5.07 (m, 2H), 5.00–4.87 (m, 2H), 4.28–4.21 (m, 1H), 4.11–3.99 (m, 3H), 3.90 (d, J = 6.2 Hz, 1H), 3.61 (dd, J = 11.2, 5.6 Hz, 1H), 3.48 (dd, J = 11.4, 2.7 Hz, 1H), 2.14–2.10 (m, 9H), 2.03–1.98 (m, 3H), 2.03 (s, 3H), 2.02 (s, 3H), 2.00 (s, 3H), 1.99 (s, 3H), 1.96 (s, 3H), 1.21 (s, 12H). ^{13}C NMR (101 MHz, $CDCl_3$) δ 170.7 (C_q), 170.5 (C_q), 170.3 (C_q), 170.2 (C_q), 169.91 (C_q), 169.86 (C_q), 169.6 (C_q), 96.3 (CH), 83.6 (C_q), 71.4 (CH), 71.3 (CH), 71.1 (CH), 70.3 (CH), 69.5 (CH), 68.3 (CH), 68.1 (CH), 67.6 (CH), 66.8 (CH_2), 66.5 (CH), 61.9 (CH_2), 53.3 (CH_2), 50.1 (C_q), 45.0 (C_q), 24.9 (CH_3), 21.0 (CH_3), 20.85 (CH_3), 20.82 (CH_3), 20.75 (CH_3). ^{11}B NMR (161 MHz, $CDCl_3$) δ 31.1. HRMS (ESI) calcd for $C_{37}H_{53}NaBO_{19}^+$ $[M+Na]^+$: 835.3172; Found: 835.3176.

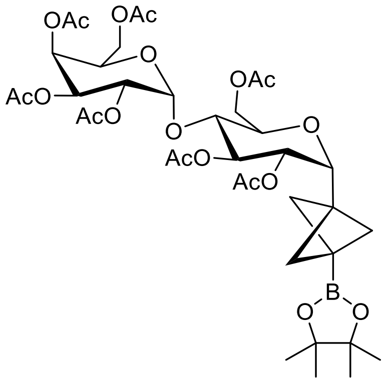

50, colorless syrup. 104.0 mg, 64% yield. >19:1 *α/β*. ^{1}H NMR (300 MHz, $CDCl_3$) δ 5.44 (dd, J = 9.2, 7.9 Hz, 1H), 5.33 (d, J = 2.3 Hz, 1H), 5.10 (dd, J = 10.4, 7.8 Hz, 1H), 5.03–4.89 (m, 2H), 4.47 (d, J = 7.9 Hz, 1H), 4.31 (dd, J = 11.5, 2.3 Hz, 1H), 4.13–3.97 (m, 4H), 3.91–3.81 (m, 2H), 3.58 (dd, J = 8.8, 7.9 Hz, 1H), 2.14 (s, 3H), 2.10 (s, 3H), 2.08 (dd, J = 9.4, 1.9 Hz, 3H), 2.04 (s, 3H), 2.04 (s, 3H), 2.03 (s, 3H), 1.97 (dd, J = 9.4, 1.9 Hz, 3H), 1.95 (s, 3H), 1.23 (s, 12H). ^{13}C NMR (75 MHz, $CDCl_3$) δ 170.7 (C_q), 170.5 (C_q), 170.3 (C_q), 170.22 (C_q), 170.16 (C_q), 169.6 (C_q), 169.2 (C_q), 101.3 (CH), 83.7 (C_q), 76.9 (CH), 71.7 (CH), 71.2 (CH), 70.9 (CH), 70.8 (CH), 69.8 (CH), 69.2 (CH), 66.9 (CH), 62.8 (CH_2), 61.1 (CH_2), 53.0 (CH_2), 44.9 (C_q), 24.9 (CH_3), 21.01 (CH_3), 20.98 (CH_3), 20.95 (CH_3), 20.8 (CH_3), 20.7 (CH_3). ^{11}B NMR (161 MHz, $CDCl_3$) δ 31.3. HRMS (ESI) calcd for $C_{37}H_{53}NaBO_{19}^+$ $[M+Na]^+$: 835.3172; Found: 835.3177.

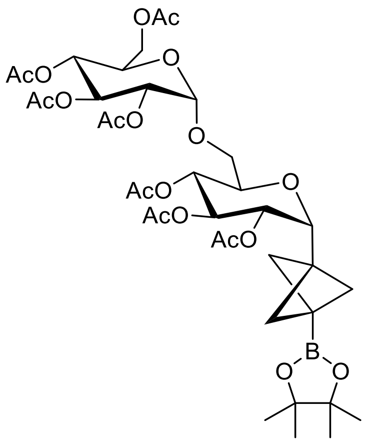

51, colorless syrup. 87.7 mg, 54% yield. >19:1 *α/β*. ^{1}H NMR (400 MHz, $CDCl_3$) δ 5.47 (ddd, *J* = 10.3, 9.1, 6.5 Hz, 2H), 5.10–4.84 (m, 5H), 4.25–4.20 (m, 1H), 4.12–4.04 (m, 3H), 3.91 (d, *J* = 6.2 Hz, 1H), 3.63 (dd, *J* = 11.2, 5.6 Hz, 1H), 3.51 (dd, *J* = 11.2, 2.9 Hz, 1H), 2.14 (dd, *J* = 9.6, 2.1 Hz, 3H), 2.12 (s, 3H), 2.09 (s, 3H), 2.04–1.99 (m, 3H), 2.03 (s, 3H), 2.03 (s, 6H), 2.01 (s, 3H), 1.99 (s, 3H), 1.22 (s, 13H). ^{13}C NMR (101 MHz, $CDCl_3$) δ 170.8 (C_q), 170.5 (C_q), 170.2 (C_q), 170.0 (C_q), 169.9 (C_q), 169.8 (C_q), 169.7 (C_q), 96.0 (CH), 83.6 (C_q), 71.4 (CH), 71.3 (CH), 71.2 (CH), 70.8 (CH), 70.4 (CH), 70.3 (CH), 69.7 (CH), 68.6 (CH), 67.4 (CH_2), 67.3 (CH), 62.0 (CH_2), 53.3 (CH), 50.2 (C_q), 45.0 (C_q), 24.9 (CH_3), 20.88, 20.85, 20.83, 20.79. ^{11}B NMR (161 MHz, $CDCl_3$) δ 30.9. HRMS (ESI) calcd for $C_{37}H_{53}NaBO_{19}^+$ $[M+Na]^+$: 835.3172; Found: 835.3176.

Gram-scale reaction and modifications of glycosyl BCP-I/-Bpin

Gram-scale reaction of 21

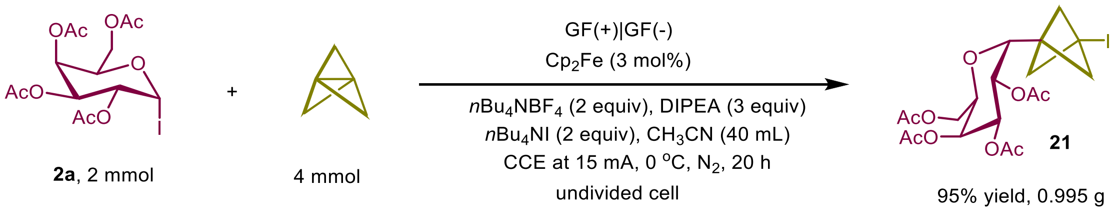

The electrolysis was carried out in an undivided cell equipped with a graphite felt (GF) anode (25 mm × 40 mm × 2 mm) and a GF cathode (25 mm × 40 mm × 2 mm). In a Schlenk tube, tetrabutylammonium tetrafluoroborate (4 mmol, 1.317 g), ferrocene (0.06

mmol, 11.2 mg) and the **2a** (2 mmol, 1.832 g) were added, followed by three cycles of evacuation and backfilling with nitrogen using a dual manifold Schlenk line. Under a nitrogen atmosphere, [1.1.1]propellane (4 mmol, 8 mL, 0.5 M), DIPEA (6 mmol, 0.774 g) and CH_3CN (35 mL) were added via syringe. Electrolysis was performed under a constant current of 15 mA for 10 minutes, after which tetrabutylammonium iodide (4 mmol, 1.478 g) dissolved in 5 mL of CH_3CN was added to the reaction mixture via syringe. The reaction was then continued for 20 hours at 0 °C. After completion, the reaction mixture was transferred to a flask, and the electrodes were rinsed with DCM (3 × 10.0 mL). The solvent was then removed under reduced pressure. The crude product was initially analyzed by NMR to determine the ratio of *α*- and *β*-products, followed by purification via column chromatography on silica gel (*n*-hexane/EtOAc) to afford the desired product **21** (0.995 g, 95% yield, *α*/*β* > 19:1).

Gram-scale reaction of 41

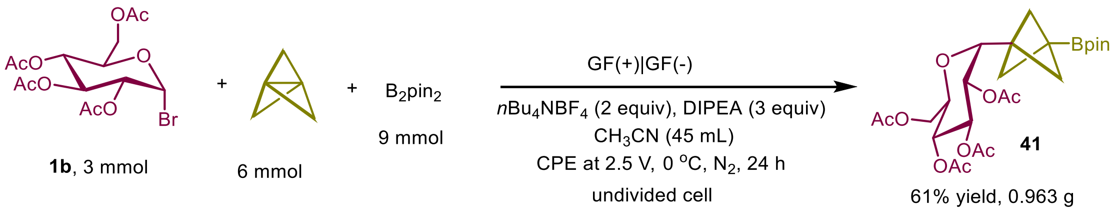

The electrolysis was carried out in an undivided cell equipped with a graphite felt (GF) anode (25 mm × 40 mm × 2 mm) and a GF cathode (25 mm × 40 mm × 2 mm). In a Schlenk tube, tetrabutylammonium tetrafluoroborate (6 mmol, 1.976 g), B_2pin_2 (9 mmol, 2.286 g) and the **1b** (3 mmol, 1.233 g) were added, followed by three cycles of evacuation and backfilling with nitrogen using a dual manifold Schlenk line. Under a nitrogen atmosphere, [1.1.1]propellane (6 mmol, 12 mL, 0.5 M), DIPEA (9 mmol, 1.161 g) and CH_3CN (45 mL) were added via syringe. Electrolysis was performed under a constant potential of 2.5 V for 24 hours at 0 °C. After completion, the reaction mixture was transferred to a flask, and the electrodes were rinsed with DCM (3 × 10.0 mL). The solvent was then removed under reduced pressure. The crude product was initially

analyzed by NMR to determine the ratio of *α*- and *β*-products, followed by purification via column chromatography on silica gel (*n*-hexane/EtOAc) to afford the desired product **41** (0.963 g, 61% yield, *α*/*β* > 19:1).

Modifications of glycosyl BCP−I (21) and glycosyl BCP−Bpin (41)

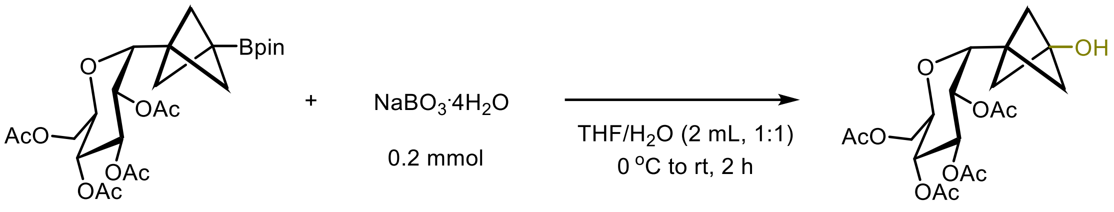

In a tube (10 mL) equipped with a magnetic stirring bar, **41** (79.5 mg, 0.2 mmol) was dissolved in THF/H_2O (1 mL/1 mL). The reaction mixture was cooled to 0 °C, and $NaBO_3 \cdot 4H_2O$ (20.4 mg, 0.2 mmol) was added. The reaction mixture was allowed to stir at room temperature for 2 h. The reaction mixture was then quenched by addition of a saturated aqueous $Na_2S_2O_3$ solution and extracted with EtOAc (3 x 5 mL). The combined organic layer was dried over $MgSO_4$ followed by filtration. The crude material was purified by silica gel chromatography (hexanes/ethyl acetate) to afford **52**.

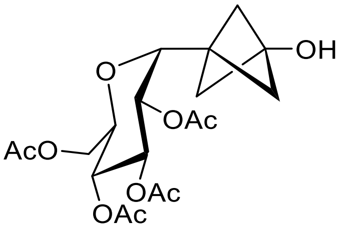

52, colorless syrup. 71.3 mg, 86% yield. ^{1}H NMR (300 MHz, $CDCl_3$) δ 5.38 (t, J = 9.2 Hz, 1H), 5.05 (dd, J = 9.7, 6.0 Hz, 1H), 4.93 (t, J = 9.1 Hz, 1H), 4.33 (d, J = 6.0 Hz, 1H), 4.17 (dd, J = 12.0, 5.5 Hz, 1H), 4.09–3.98 (m, 2H), 2.98 (brs, 1H), 2.10 (dd, J = 9.4, 1.4 Hz, 3H), 2.07 (s, 3H), 2.03 (s, 3H), 2.02 (s, 3H), 2.02 (s, 6H), 2.00 (dd, J = 9.4, 1.4 Hz, 3H). ^{13}C NMR (75 MHz, $CDCl_3$) δ 170.9 (C_q), 170.2 (C_q), 169.8 (C_q), 169.7 (C_q), 71.0 (CH), 70.5 (CH), 70.1 (CH), 70.0 (CH), 68.8 (CH), 63.3 (C_q), 62.6 (CH_2), 55.6 (CH_2), 30.5 (C_q), 20.83 (CH_3), 20.81 (CH_3), 20.77 (CH_3). HRMS (ESI) calcd for $C_{19}H_{26}NaO_{10}^+$ $[M+Na]^+$: 437.1418; Found: 437.1419.

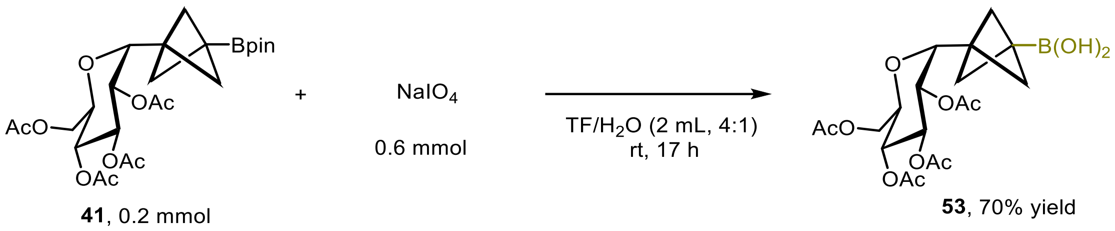

According to the reported method[2]. To a solution of **41** (79.5 mg, 0.2 mmol) in 4:1 THF/H_2O (2 mL) at room temperature, $NaIO_4$ (129.6 mg, 3.0 equiv.) was added. The solution was stirred at room temperature, under air for 17 hours. The reaction mixture was then diluted with brine and extracted with EtOAc (x3). The combined organics were washed with water and brine, dried over Na_2SO_4, and concentrated under reduced pressure. The resultant crude alkylboronic acids have been reported to proceed to the cross-coupling step without further purification, and the yield of deprotection was estimated to be quantitative[2]. Here we obtained product **53** by GPC purification.

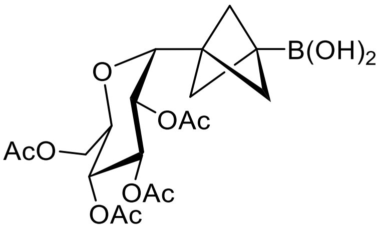

53, colorless syrup. 61.9 mg, 70% yield. ^{1}H NMR (400 MHz, $CDCl_3$) δ 5.43–5.34 (m, 1H), 5.06 (dd, J = 9.6, 6.1 Hz, 1H), 4.96–4.89 (m, 1H), 4.33 (d, J = 6.0 Hz, 1H), 4.17 (dd, J = 12.0, 5.5 Hz, 1H), 4.10–3.98 (m, 2H), 2.12–2.08 (m, 3H), 2.07 (s, 3H), 2.04–2.00 (m, 12H). ^{13}C NMR (101 MHz, $CDCl_3$) δ 170.8 (C_q), 170.2 (C_q), 169.8 (C_q), 169.7 (C_q), 71.0 (CH), 70.5 (CH), 70.1 (CH), 70.0 (CH), 68.8 (CH), 63.3 (C_q), 62.6 (CH_2), 55.6 (CH_2), 30.5 (C_q), 20.84 (CH_3), 20.82 (CH_3), 20.78 (CH_3). ^{11}B NMR (161 MHz, $CDCl_3$) δ -5.7. HRMS (ESI) calcd for $C_{19}H_{27}NaBO_{11}^+$ $[M+Na]^+$: 465.1540; Found: 465.1728.

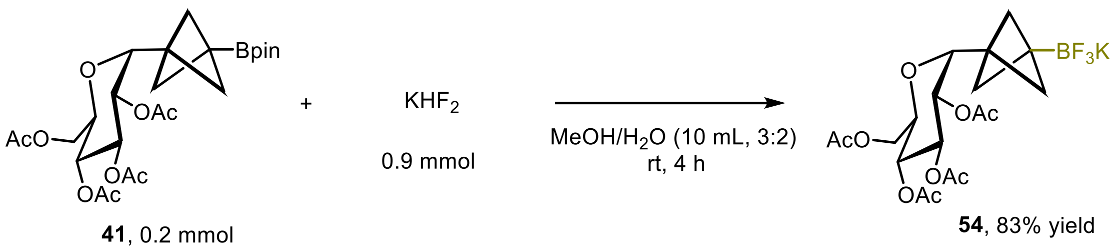

In a tube (10 mL) equipped with a magnetic stirring bar, **41** (79.5 mg, 0.2 mmol) was dissolved in MeOH (6 mL)/H_2O (4 mL), and then KHF_2 (70.2 mg) was added to the mixture at room temperature. The reaction mixture was stirred vigorously at room temperature for 4 h. After 4 h, the solvents were evaporated to dryness under reduced pressure. The resulting crude material was extracted with hot acetone (3×50 mL), followed by filtration. The combined filtrates were concentrated and then triturated with approximately 10 mL of Et_2O. The resultant precipitate was collected by vacuum filtration and dried under vacuum to afford **54**.

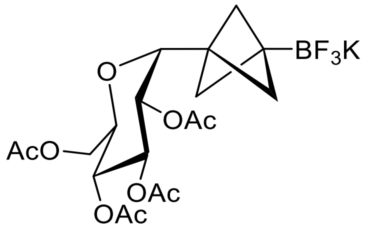

54, colorless syrup. 83.7 mg, 83% yield. ^{1}H NMR (500 MHz, DMSO-*d6*) δ 5.46 (t, *J* = 9.5 Hz, 1H), 4.82–4.74 (m, 2H), 4.13–4.08 (m, 1H), 4.04 (dd, *J* = 12.0, 5.8 Hz, 1H), 3.96 (dd, *J* = 12.0, 2.6 Hz, 1H), 3.73 (d, *J* = 6.0 Hz, 1H), 1.99 (s, 3H), 1.98 (s, 3H), 1.98 (s, 3H), 1.97 (s, 3H), 1.57 (d, *J* = 9.0 Hz, 3H), 1.45 (d, *J* = 9.0 Hz, 3H).^{13}C NMR (126 MHz, DMSO-*d6*) δ 170.1 (C_q), 169.7 (C_q), 169.6 (C_q), 169.5 (C_q), 71.1 (CH), 70.9 (CH), 69.7 (CH), 69.3 (CH), 68.8 (CH), 62.5 (CH_2), 51.2 (CH_2), 48.42 (q, *J* = 4.5 Hz, C_q), 41.52 (q, *J* = 2.3 Hz, C_q), 20.6 (CH_3), 20.51 (CH_3), 20.48 (CH_3), 20.4 (CH_3). ^{11}B NMR (161 MHz, DMSO-*d6*) δ 0.3. ^{19}F NMR (282 MHz, DMSO-*d6*) δ -143.0. HRMS (ESI) calcd for $C_{19}H_{25}BF_3NaO_9^-$ $[M-K^+]^-$: 465.1553; Found: 465.1556.

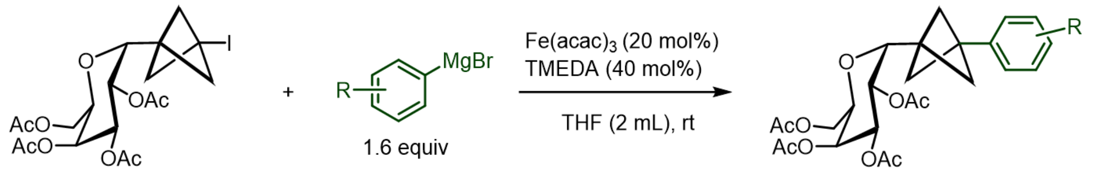

According to the reported method[3]. To a flame-dried vial was added **21** (0.2 mmol, 104.9 mg) and Fe(acac)$_3$ (14 mg, 20 mol%, 0.04 mmol). The vial was then evacuated and refilled with N_2 three times. To this was added THF (0.2 mL) and TMEDA (12 μL, 40 mol%, 0.08 mmol), and the resulting mixture was stirred for 5 min. The Grignard reagent (1.6 equiv., 0.32 mmol) was then added via syringe pump at a rate of 0.7 mL/h (over approximately 45 mins) at the specified temperature. The reaction was stirred for a further 1 h, then quenched by addition of aqueous HCl (5 mL, 1 M) or aqueous NH_4Cl (5 mL, saturated). The layers were separated, and the aqueous layer was extracted with Et_2O (3 × 10 mL). The combined organic layers were washed with brine, dried over $MgSO_4$ and concentrated in vacuo. The crude product was purified by column chromatography to obtain the arylation products.

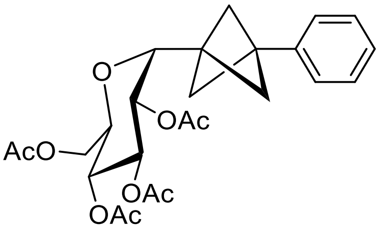

55, colorless syrup. 42.7 mg, 45% yield. ^{1}H NMR (300 MHz, $CDCl_3$) δ 7.33–7.27 (m, 2H), 7.25–7.17 (m, 3H), 5.46–5.28 (m, 3H), 4.35–4.20 (m, 3H), 4.04 (dd, *J* = 11.2, 5.0 Hz, 1H), 2.20 (dd, *J* = 9.5, 1.6 Hz, 3H), 2.12 (s, 3H), 2.11–2.02 (m, 12H). ^{13}C NMR (75 MHz, $CDCl_3$) δ 170.7 (C_q), 170.2 (C_q), 170.02 (C_q), 169.99 (C_q), 140.0 (C_q), 128.3 (CH), 126.8 (CH), 126.0 (CH), 70.5 (CH), 69.8 (CH), 68.9 (CH), 67.9 (CH), 67.7 (CH), 61.9 (CH_2), 53.6 (CH_2), 43.3 (C_q), 38.0 (C_q), 21.0 (CH_3), 20.84 (CH_3), 20.77 (CH_3). HRMS (ESI) calcd for $C_{25}H_{30}NaO_9^+$ [M+Na]$^+$: 497.1782; Found: 497.1779.

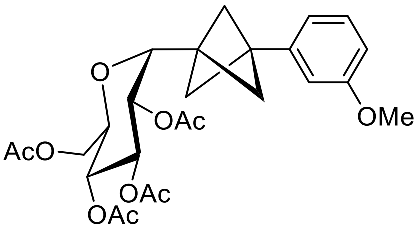

56, colorless syrup. 43.4 mg, 43% yield. ^{1}H NMR (300 MHz, $CDCl_3$) δ 7.22 (t, *J* = 7.8 Hz, 1H), 6.82–6.70 (m, 3H), 5.48–5.28 (m, 3H), 4.36–4.19 (m, 3H), 4.05 (dd, *J* = 11.1, 4.8 Hz, 1H), 3.80 (s, 3H), 2.19 (dd, *J* = 9.6, 1.7 Hz, 3H), 2.13–2.07 (m, 3H), 2.12 (s, 3H), 2.08 (s, 3H), 2.06 (s, 3H), 2.04 (s, 3H). ^{13}C NMR (75 MHz, $CDCl_3$) δ 170.7 (C_q), 170.2 (C_q), 170.03 (C_q), 169.98 (C_q), 159.7 (C_q), 141.6 (C_q), 129.4 (CH), 118.4 (CH), 112.2 (CH), 111.8 (CH), 70.5 (CH), 69.8 (CH), 68.8 (CH), 67.9 (CH), 67.7 (CH), 61.9 (CH_2), 55.3 (CH_3), 53.6 (CH_2), 43.2 (C_q), 38.0 (C_q), 21.0 (CH_3), 20.84 (CH_3), 20.76 (CH_3). HRMS (ESI) calcd for $C_{26}H_{32}NaO_{10}^+$ $[M+Na]^+$: 527.1888; Found: 527.1887.

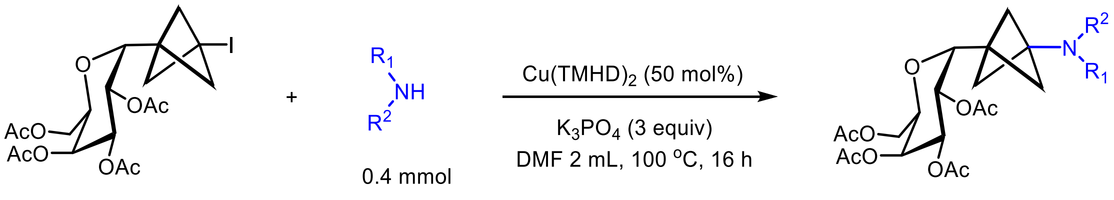

According to the reported method[4]. Inside of an argon-filled glovebox, nitrogen nucleophile (0.2 mmol, 1.0 eq.), **21** (0.4 mmol, 209.7 mg), $Cu(TMHD)_2$ (0.1 mmol, 43.01 mg, 50 mol%) and K_3PO_4 (0.6 mmol, 127.36 mg, 3.0 eq.) were weighed into an 8 mL vial and dissolved in 2 mL DMF at rt. The reaction was sealed and stirred at 100ºC for 16 hours. After the reaction was completed, the mixture was allowed to cool down to room temperature. Water was added (2 mL). Then, the crude mixture was extracted with AcOEt (3 x 2 mL). All organic layers were combined, dried over $MgSO_4$ and the solvent was removed under vacuum to afford a crude product, which was redissolved in MeOH (2 mL). The resulting solution was filtered through a scavenger pad (SiliaMet Imidazole, 0.5 g, 1.2 mmol/g) to remove the residual copper catalyst. The solvent was

removed under vacuum and the crude product was purified via flash column chromatography to obtain the amination products.

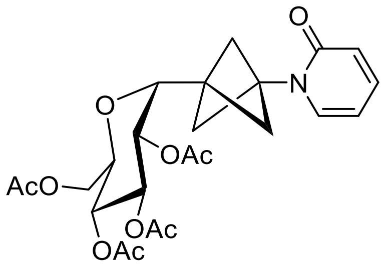

57, colorless syrup. 49.1 mg, 50% yield. ^{1}H NMR (300 MHz, $CDCl_3$) δ 8.17–8.12 (m, 1H), 7.60–7.52 (m, 1H), 6.93–6.86 (m, 1H), 5.45–5.40 (m, 1H), 5.36–5.27 (m, 2H), 4.46 (t, *J* = 2.5 Hz, 1H), 4.31–4.18 (m, 2H), 4.05 (dd, *J* = 10.5, 4.3 Hz, 1H), 2.42 (dd, *J* = 9.2, 0.8 Hz, 3H), 2.33 (dd, *J* = 9.3, 1.0 Hz, 3H), 2.12 (s, 3H), 2.08 (s, 3H), 2.06 (s, 3H), 2.03 (s, 3H). ^{13}C NMR (75 MHz, $CDCl_3$) δ 170.7 (C_q), 170.3 (C_q), 170.0 (C_q), 163.2 (C_q), 147.2 (CH), 138.8 (CH), 117.7 (CH), 111.6 (CH), 69.7 (CH), 68.8 (CH), 67.8 (CH), 67.7 (CH), 64.8 (CH_2), 61.9 (C_q), 55.2 (CH_2), 33.8 (C_q), 21.0 (CH_3), 20.9 (CH_3), 20.8 (CH_3). HRMS (ESI) calcd for $C_{24}H_{29}NNaO_{10}^{+}$ $[M+Na]^{+}$: 514.1684; Found: 514.1680.

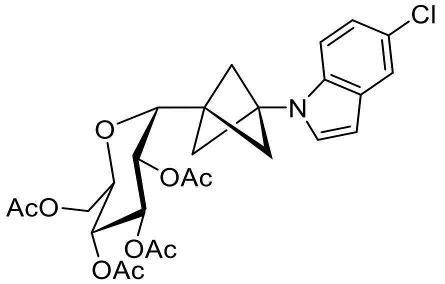

58, colorless syrup. 55.9 mg, 51% yield. ^{1}H NMR (300 MHz, $CDCl_3$) δ 7.50 (d, *J* = 8.4 Hz, 1H), 7.45 (s, 1H), 7.08 (dd, *J* = 8.4, 1.8 Hz, 1H), 7.01 (d, *J* = 3.3 Hz, 1H), 6.45 (dd, *J* = 3.2, 0.9 Hz, 1H), 5.46 (t, *J* = 2.3 Hz, 1H), 5.36–5.32 (m, 2H), 4.48–4.26 (m, 3H), 4.04 (dd, *J* = 11.1, 4.3 Hz, 1H), 2.52 (dd, *J* = 9.5, 1.5 Hz, 3H), 2.45 (dd, *J* = 9.5, 1.6 Hz, 3H), 2.13 (s, 3H), 2.12 (s, 3H), 2.08 (s, 3H), 2.07 (s, 3H). ^{13}C NMR (75 MHz, $CDCl_3$) δ 170.8 (C_q), 170.1 (C_q), 169.93 (C_q), 169.88 (C_q), 136.2 (C_q), 127.9 (C_q), 127.9 (C_q), 126.7 (CH), 122.0 (CH), 120.6 (CH), 110.8 (CH), 102.2 (CH), 70.3 (CH), 69.2 (CH),

68.7 (CH), 67.6 (CH), 67.4 (CH), 61.4 (CH_2), 54.4 (CH_2), 50.5 (C_q), 36.1 (C_q), 21.1 (CH_3), 20.91 (CH_3), 20.91 (CH_3), 20.8 (CH_3). HRMS (ESI) calcd for $C_{27}H_{30}ClNNaO_9^+$ $[M+Na]^+$: 570.1501; Found: 570.1498.

Mechanistic studies

Radical trap experiment

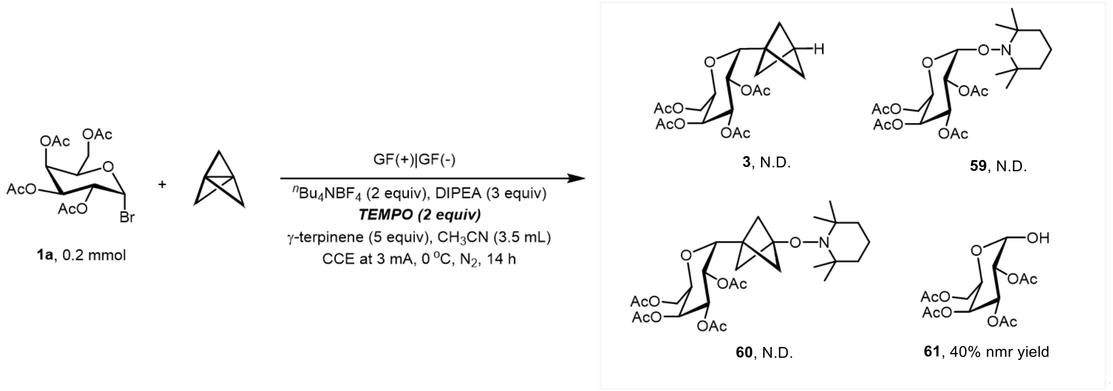

Scheme S1. Radical trap experiment 1. Reaction conditions: general procedure A, **1a** (0.2 mmol), [1.1.1]propellane (0.4 mmol), TEMPO (0.4 mmol), γ-terpinene (1.0 mmol), DIPEA (0.6 mmol), *n*Bu_4NBF_4 (0.4 mmol), CH_3CN (3.5 mL) at 0 °C 14 h under N_2, GF as anode and cathode, CCE at 3.0 mA.

Herein, we selected the galactosyl BCP hydrogenation reaction as a model system to probe the generation of both galactosyl and BCP radicals. Initially, we directly added 2 equivalents of TEMPO to the reaction mixture. Under standard conditions, no formation of the desired product **3** was observed, indicating that TEMPO effectively intercepted the radical intermediates, consistent with our proposed mechanism. However, neither the galactosyl TEMPO adduct **59** nor the galactosyl BCP–TEMPO adduct **60** could be detected. Instead, we observed a significant amount of 1-position hydrolyzed byproducts **61** (Scheme S1). We hypothesize that the galactosyl TEMPO adduct **59**, once formed, may be unstable under the electrochemical conditions and undergo rapid hydrolysis. To further investigate, the reaction was conducted without γ-terpinene and was interrupted after 4 hours, at which point we presumed that galactosyl

radicals and galactosyl BCP–radicals were present in the system. Subsequently, 2 equivalents of TEMPO were added, and the mixture was stirred for an additional 2 hours without applying an electric current. Under these modified conditions, we successfully detected the galactosyl TEMPO adduct **59**. (Fig. S1)

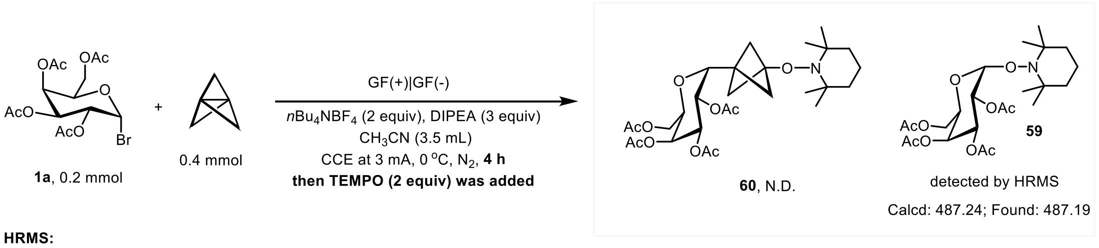

HRMS:

Acquisition Parameter

Source Type	ESI	Ion Polarity	Positive	Set Nebulizer	1.2 Bar
Focus	Not active			Set Dry Heater	180 °C
Scan Begin	50 m/z	Set Capillary	4500 V	Set Dry Gas	4.0 l/min
Scan End	1600 m/z	Set End Plate Offset	-500 V	Set Divert Valve	Source

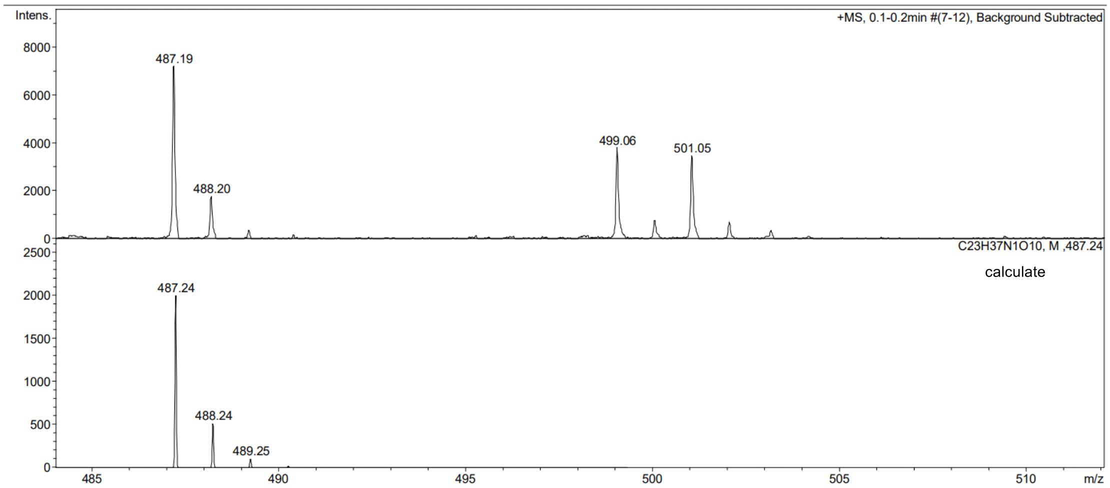

Fig. S1. Radical trap experiment 2. The reaction procedure is the same as general procedure A, except that TEMPO (0.4 mmol) is added after 4 hours of constant current electrolysis, followed by an additional 2-hour reaction at room temperature without current.

Cyclic Voltammetry studies

CV measurements were conducted with a Metrohm Autolab PGSTAT204 potentiostat and Nova 2.1 software. A glassy carbon working electrode (disk, diameter: 3mm), a coiled platinum wire counter electrode, and a SCE reference electrode were employed. The voltammograms were recorded at room temperature in CH_3CN, at a substrate concentration of 20 mM and 0.1 M $n$$Bu_4NBF_4$ as supporting electrolytes. All solutions

were degassed with N_2 before the measurement and an overpressure of protective gas was maintained throughout the experiment. The scan rate is 100 mV/s. The respective figures and descriptions indicate deviations from the general experimental conditions.

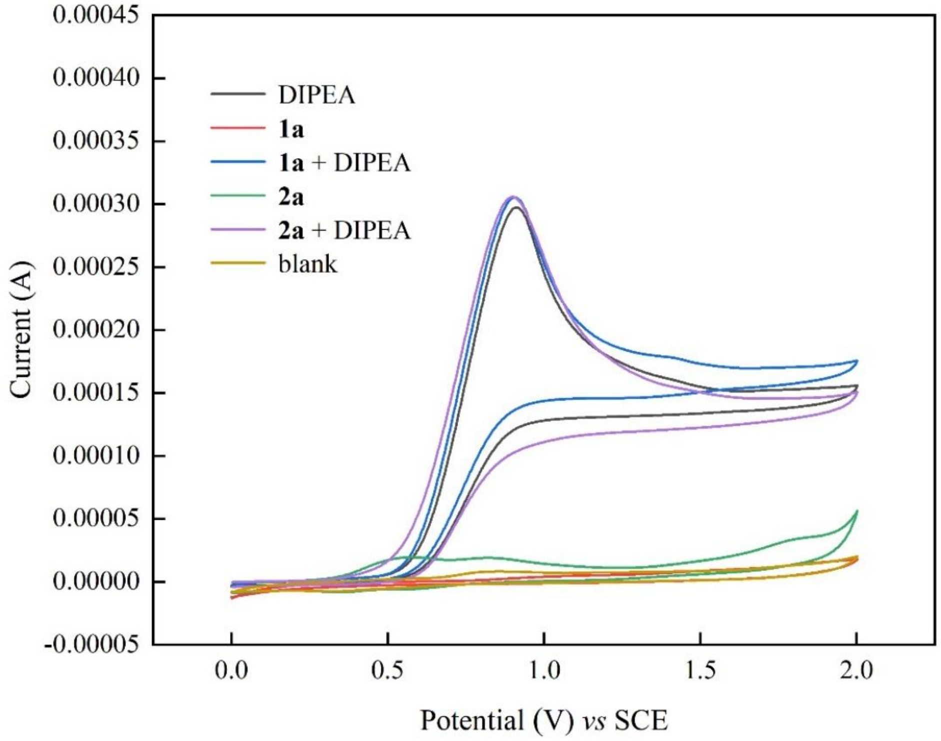

Fig. S2. Cyclic voltammograms of DIPEA and galactosyl substrates.

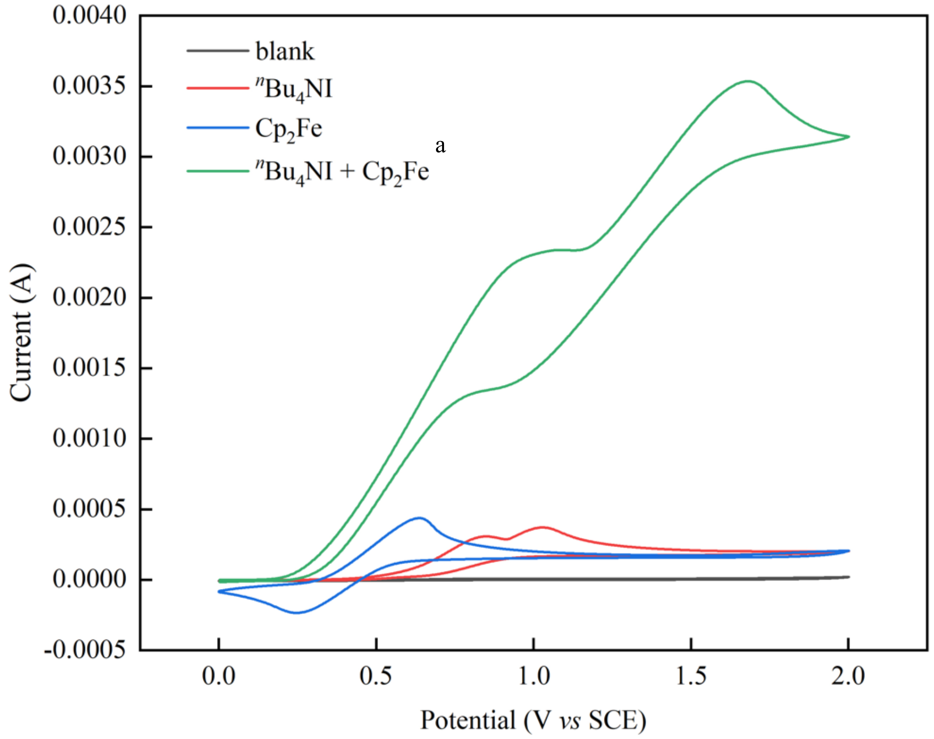

Fig. S3. Cyclic voltammograms of Cp_2Fe and $n$$Bu_4$NI. [a] $n$$Bu_4$NI (200 mM).

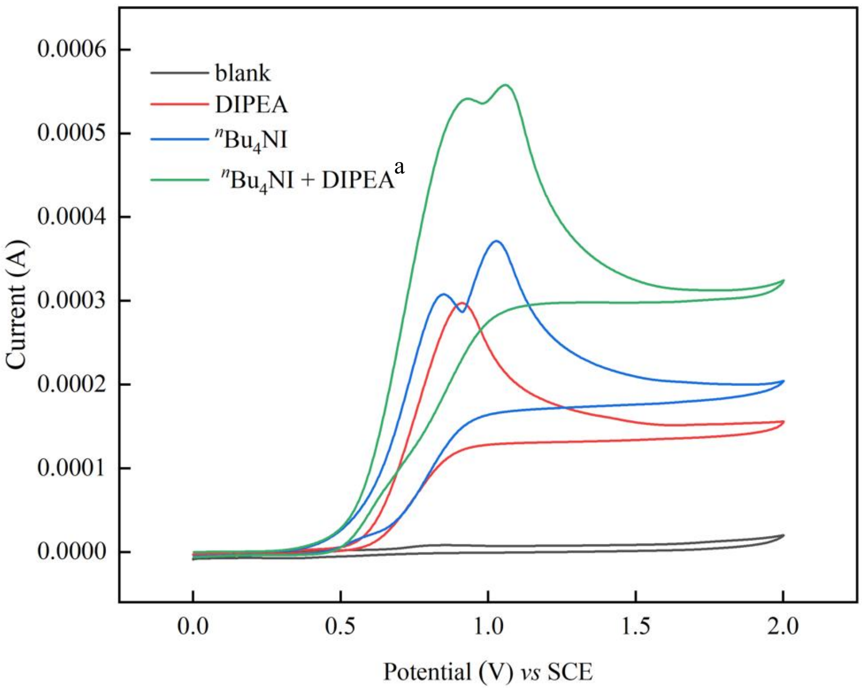

Fig. S4. Cyclic voltammograms of DIPEA and $n$$Bu_4$NI. [a] $n$$Bu_4$NI (200 mM).

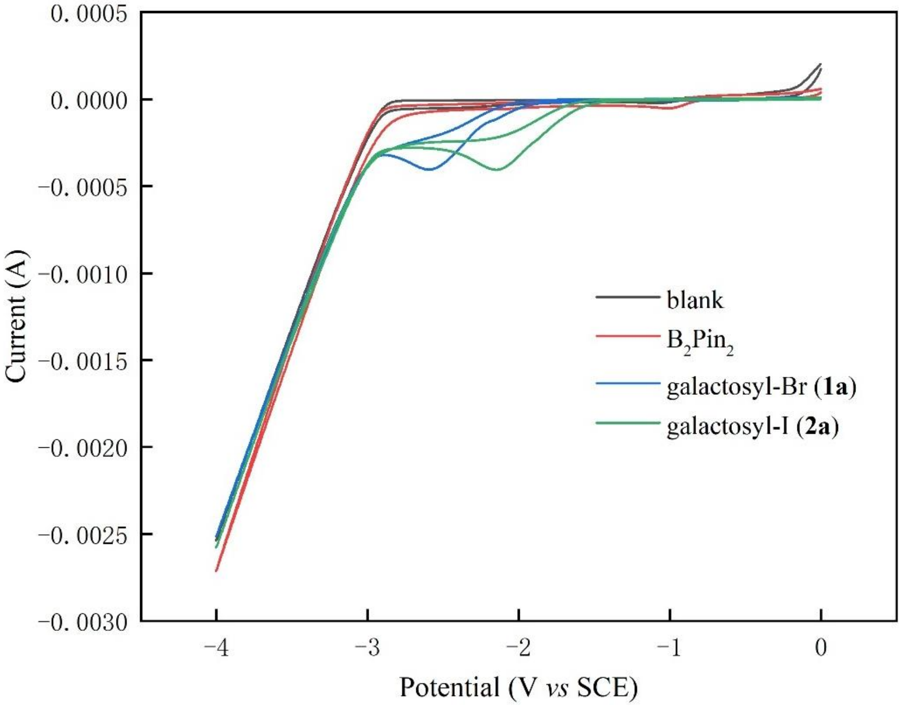

Fig. S5. Cyclic voltammograms of B_2pin_2 and glycosyl halide.

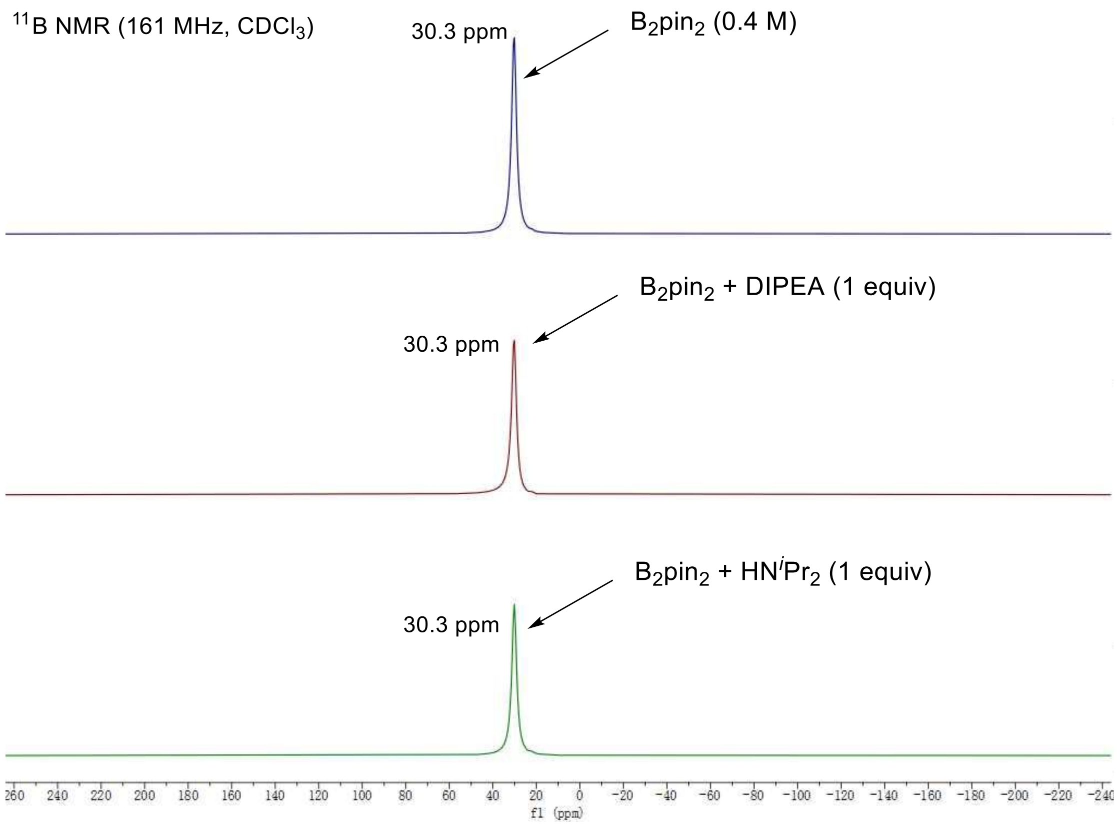

Fig. S6. ^{11}B NMR experiment.

To gain mechanistic insights into the formation of glycosyl BCP–Bpin products, we investigated several plausible pathways. Given that typical alkyl radicals exhibit low reactivity toward B_2Pin_2, we first considered whether electrochemical reduction of B_2Pin_2 at the cathode could generate a more reactive B_2Pin_2 radical anion, thereby enabling the desired transformation. However, cyclic voltammetry analysis of B_2Pin_2 (Fig. S5) revealed no reduction peak within the range of 0 to –3 V, consistent with previous observations reported by Baran[5]. This pathway was therefore ruled out.

Molander and co-workers[6] demonstrated that BCP radicals possess partial sp^2 character, which enables polarity-matched borylation with electron-rich Bpin acceptors. Their competition experiments further revealed that radicals with increased *s* character (sp^2 character) preferentially couple with Bpin over Bcat, supporting the role of the radical's electronic structure in promoting efficient borylation. In their system, coordination by Lewis-basic additives (e.g., phthalimide from redox-active ester intermediates) further activates B_2Pin_2 by weakening its B–B bond, aiding radical

capture. Therefore, we next hypothesized that B_2Pin_2 might form a Lewis-basic additives with DIPEA or with the ethylisopropylamine generated upon hydrolysis of DIPEA following XAT, which could potentially weaken the B–B bond and facilitate reaction with radicals. To test this, we performed ^{11}B NMR spectroscopy on B_2Pin_2 alone, B_2Pin_2 with DIPEA, and B_2Pin_2 with HN*i*Pr_2 (Fig. S6). In all cases, no new boron resonance was observed, suggesting that no detectable complexation occurred.

Based on these findings, we propose that the reaction proceeds due to the unique reactivity of the BCP radical, which, unlike conventional alkyl radicals, may exhibit partial *sp²*-like character that facilitates efficient coupling with B_2Pin_2, as previously described by Molander and co-workers[6].

Cathodic Process Study

In a parallel experiment, after completion of the reaction, a sample of the headspace (1 mL) was carefully collected using a gas syringe. The sample was directly analyzed via gas chromatography. The H_2 peak should be observed at a retention time of 1.56 min, as confirmed by the comparison with pure hydrogen gas as reference sample. However, no corresponding signal was detected in the reaction mixture under the current conditions (Fig. S7–S9).

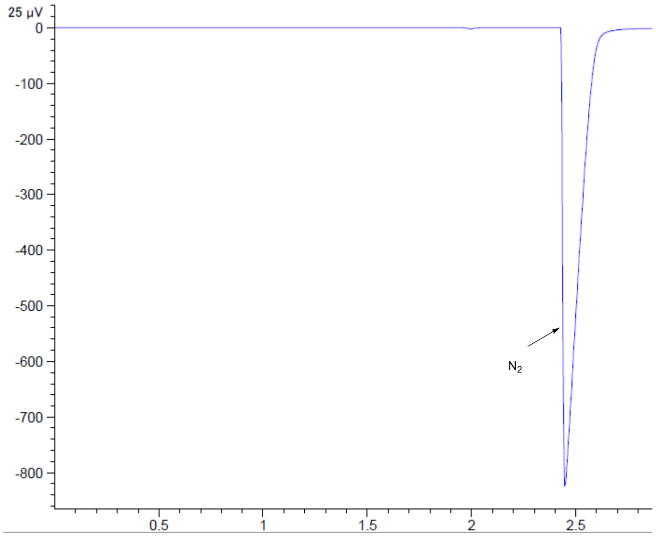

Fig. S7. GC analysis of reaction of H–BCP glycoside.

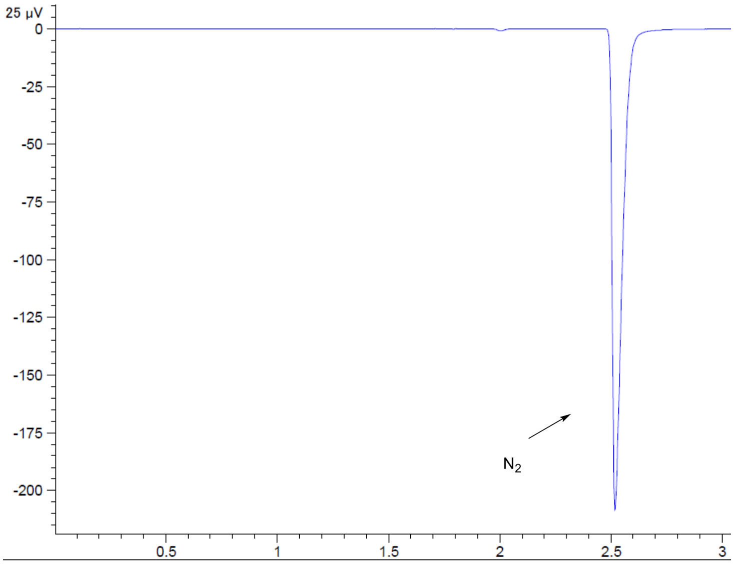

Fig. S8. GC analysis of reaction of I–BCP glycoside.

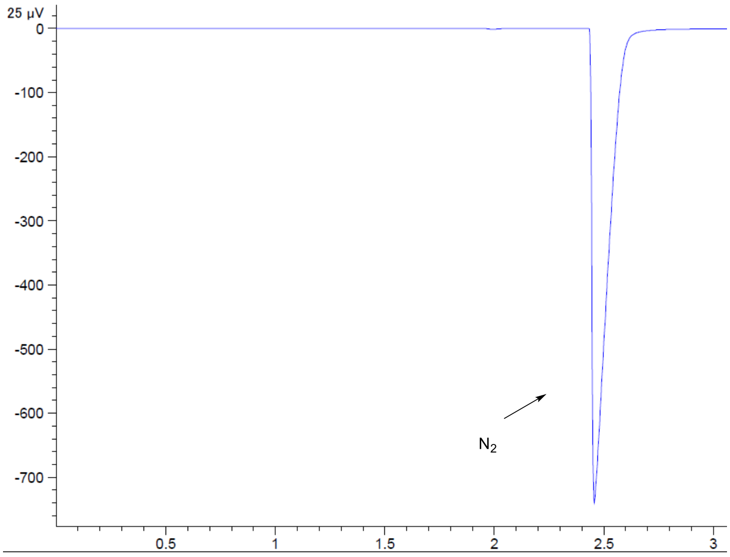

Fig. S9. GC analysis of borylation reaction.

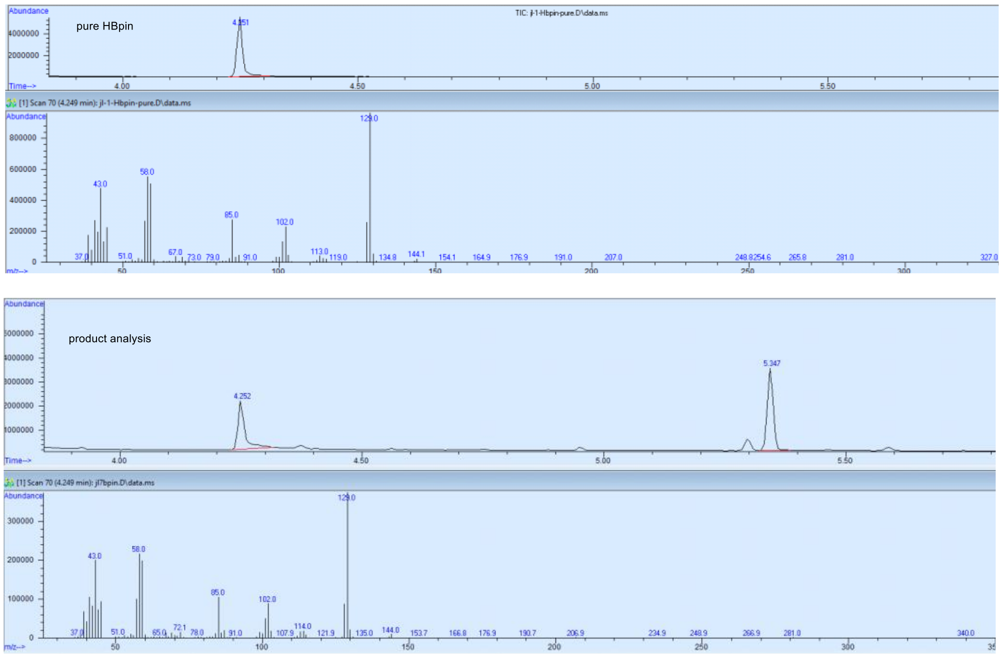

Fig. S10. Detection of HBpin by GC-MS of borylation reaction.

In this part, we explored the possible cathodic reactions in different transformations. For all three reactions (H–, I–, Bpin–BCP glycoside), no hydrogen gas was detected by GC analysis, ruling out proton reduction as the dominant cathodic pathway (Fig. S7–S9). To further probe the feasibility of direct electroreduction of glycosyl halides, control experiments were performed in the absence of DIPEA. The formation of H– and I–BCP glycosides in 25% (Table S1, entry 11) and 38% yields (Table S2, entry 5), respectively, supports the generation of glycosyl radicals via cathodic reduction. This is further supported by cyclic voltammetry measurements, which showed reduction potentials of –2.55 V (galactosyl bromide) and –2.15 V (galactosyl iodide) *vs.* SCE (Fig. S5). In the borylation reaction, we detected HBpin as a byproduct by GC-MS (Fig. S10). We therefore propose that unreacted Bpin radicals, formed upon borylation, are reduced at the cathode to generate HBpin.

In conclusion, these results indicate that glycosyl halides likely serve as the primary cathodic substrates in the formation of H– and I–BCP glycosides, whereas in the borylation reaction, the Bpin radical is more likely reduced at the cathode.

Copies of NMR spectra

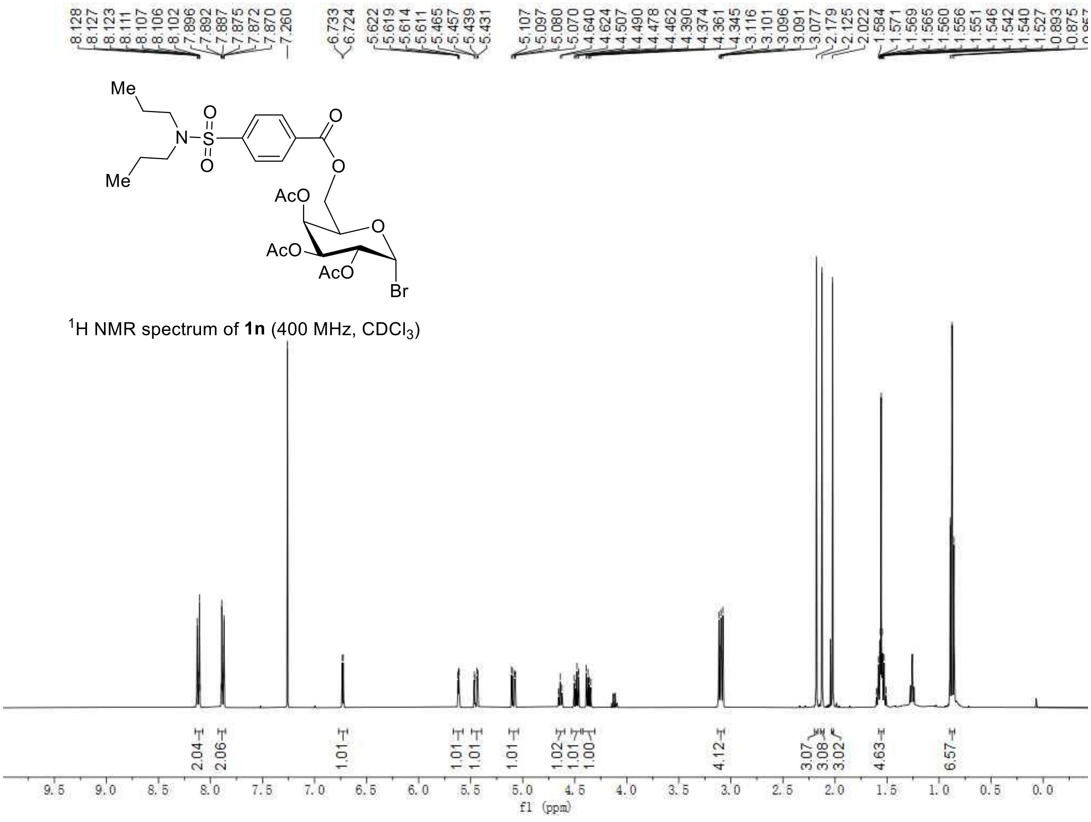

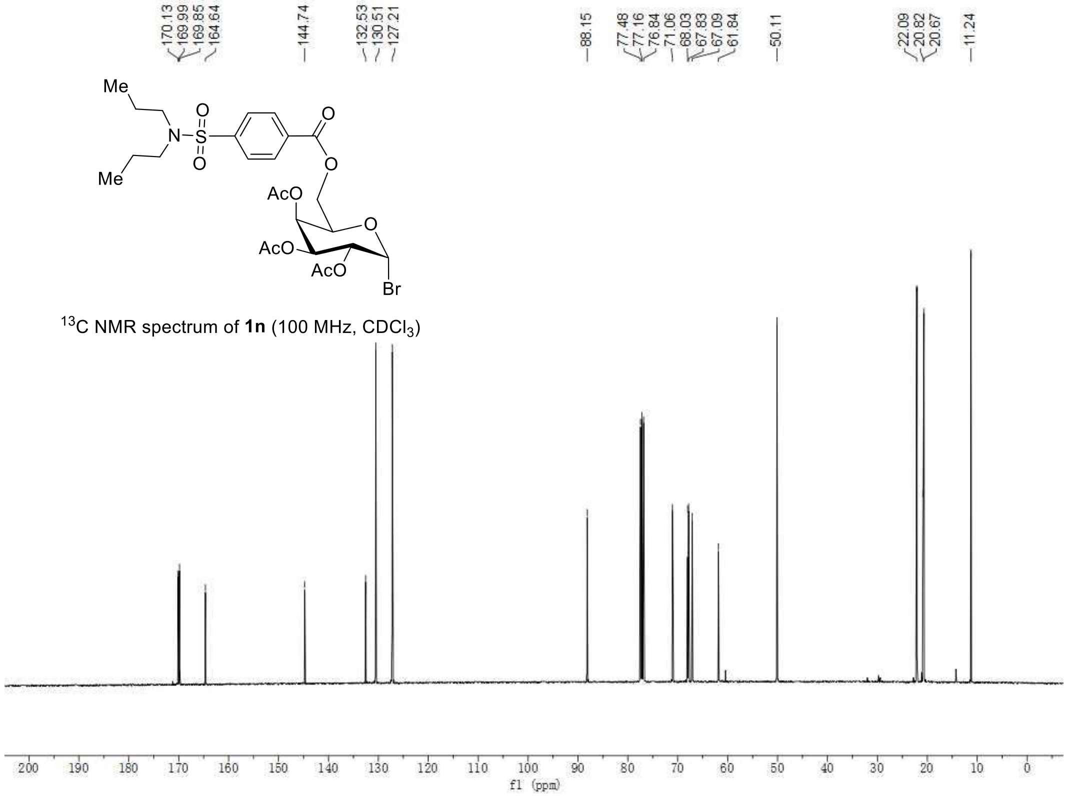

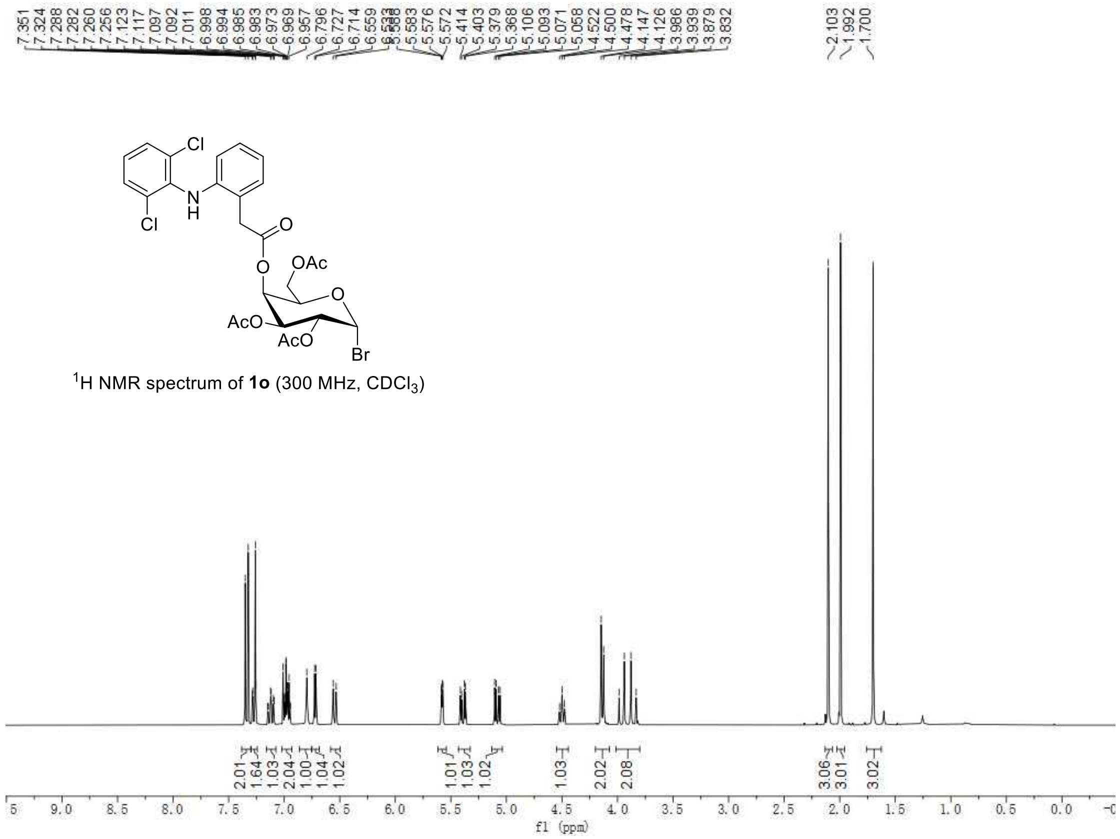

^{1}H NMR spectrum of **1o** (300 MHz, $CDCl_3$)

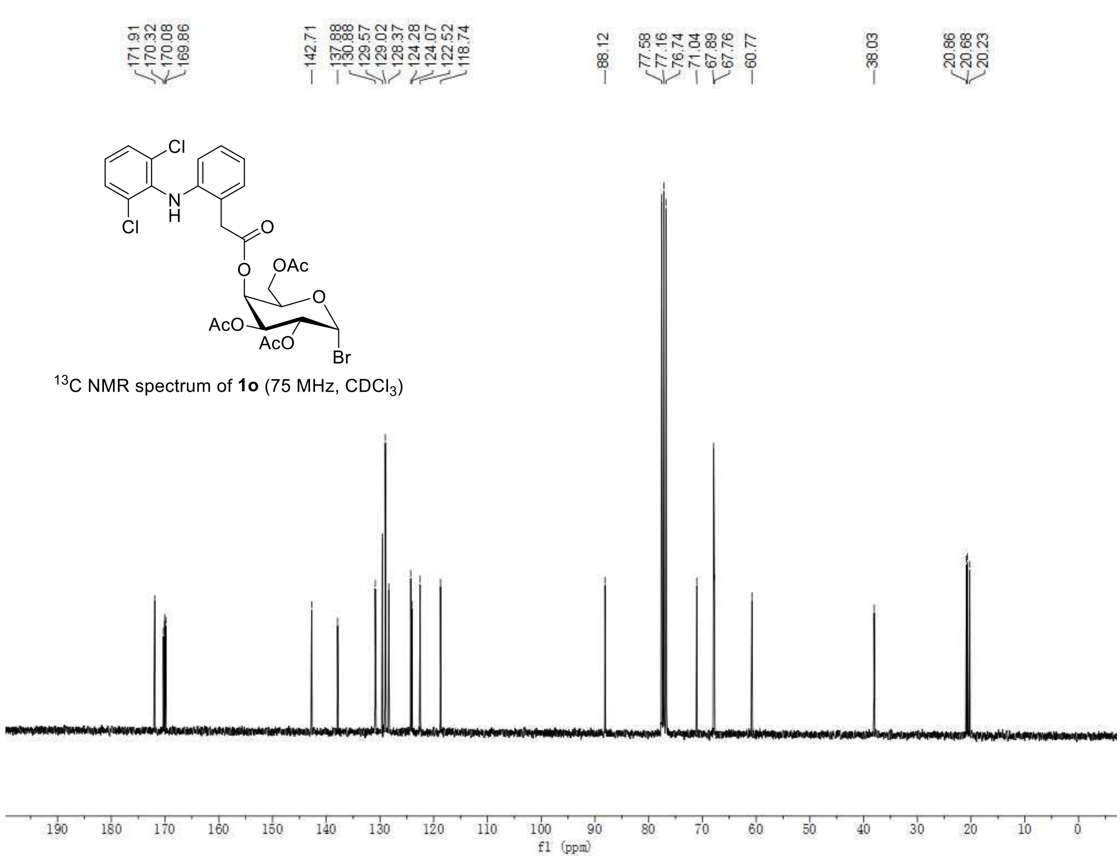

^{13}C NMR spectrum of **1o** (75 MHz, $CDCl_3$)

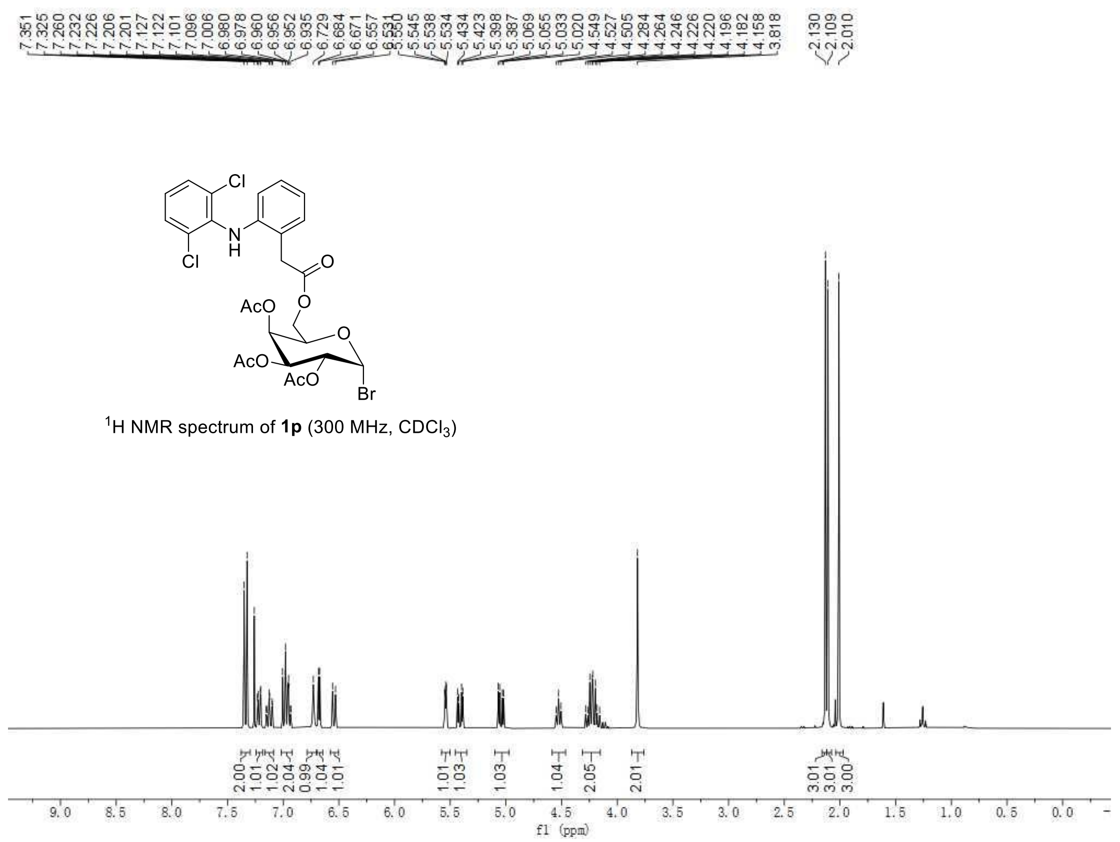

^{1}H NMR spectrum of **1p** (300 MHz, $CDCl_3$)

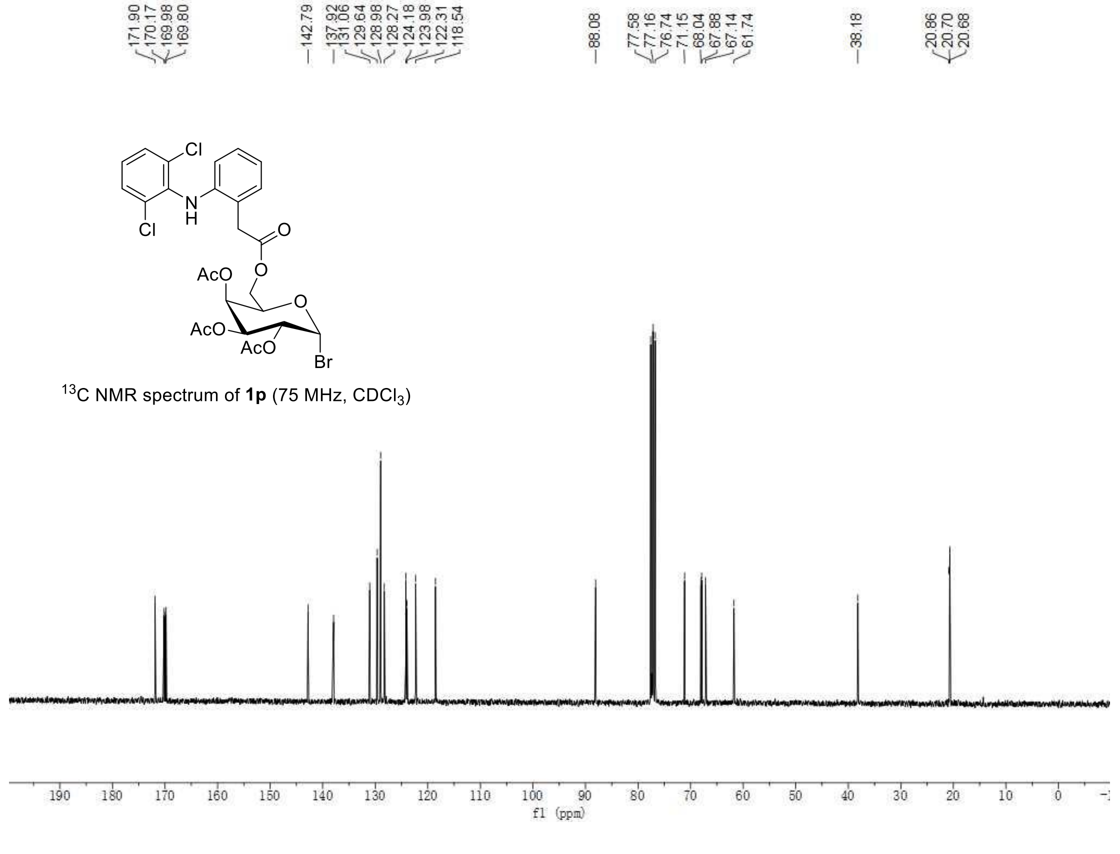

^{13}C NMR spectrum of **1p** (75 MHz, $CDCl_3$)

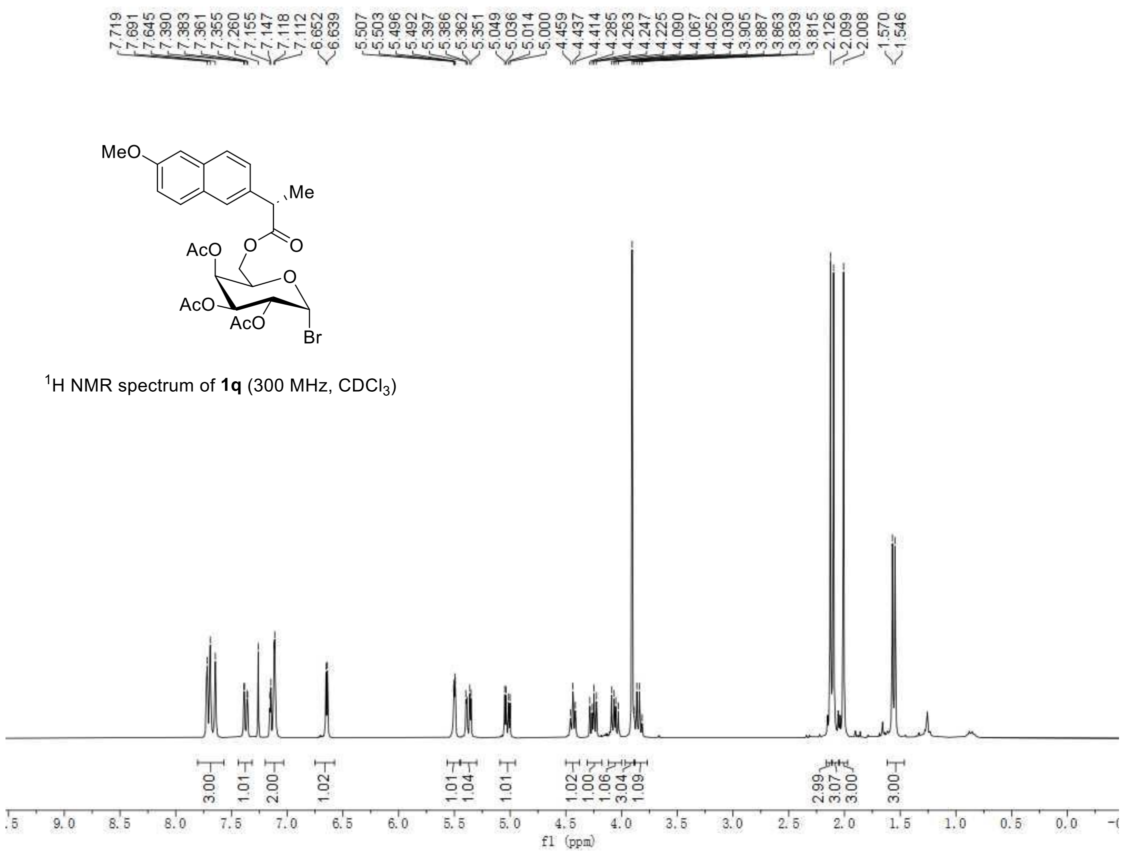

^{1}H NMR spectrum of **1q** (300 MHz, $CDCl_3$)

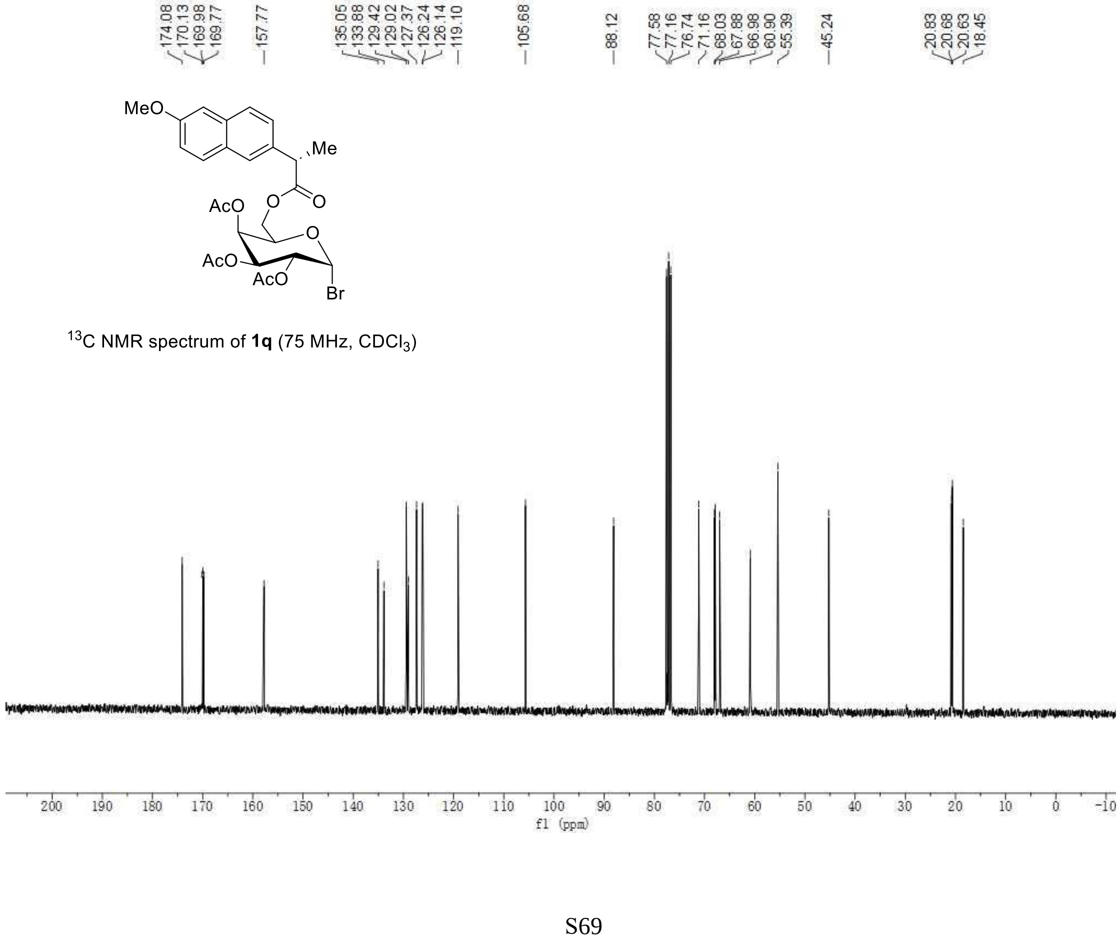

^{13}C NMR spectrum of **1q** (75 MHz, $CDCl_3$)

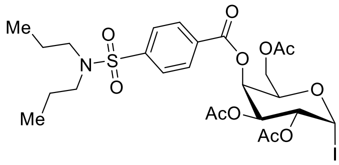

^{1}H NMR spectrum of **2k** (300 MHz, $CDCl_3$)

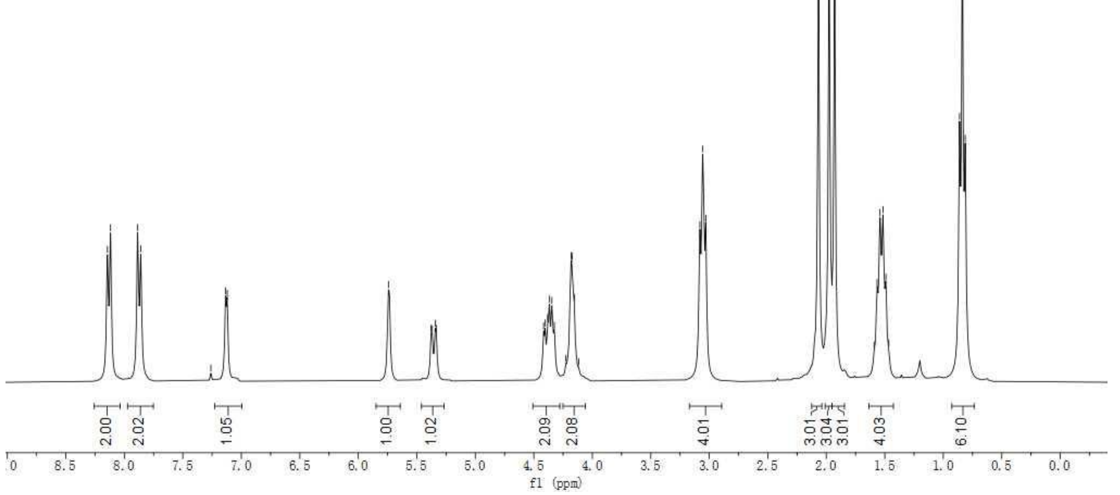

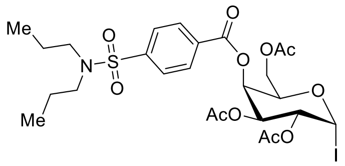

^{13}C NMR spectrum of **2k** (75 MHz, $CDCl_3$)

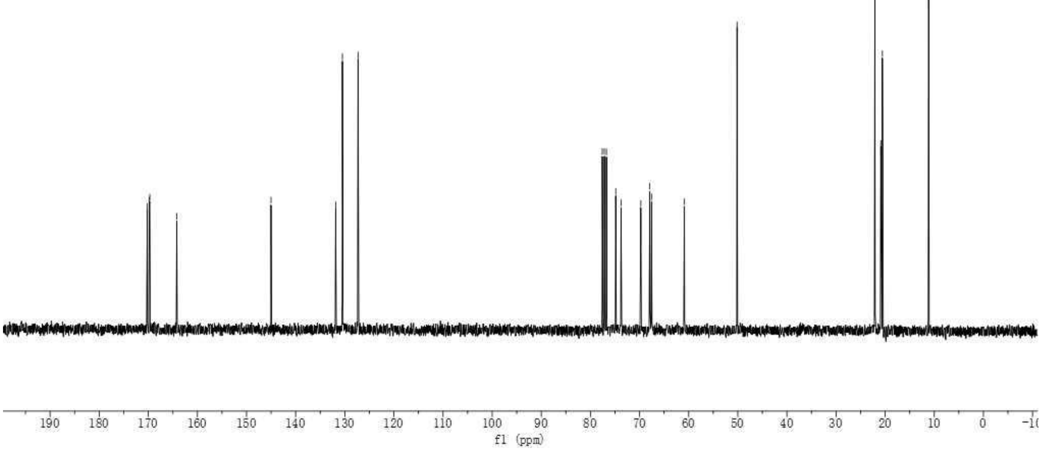

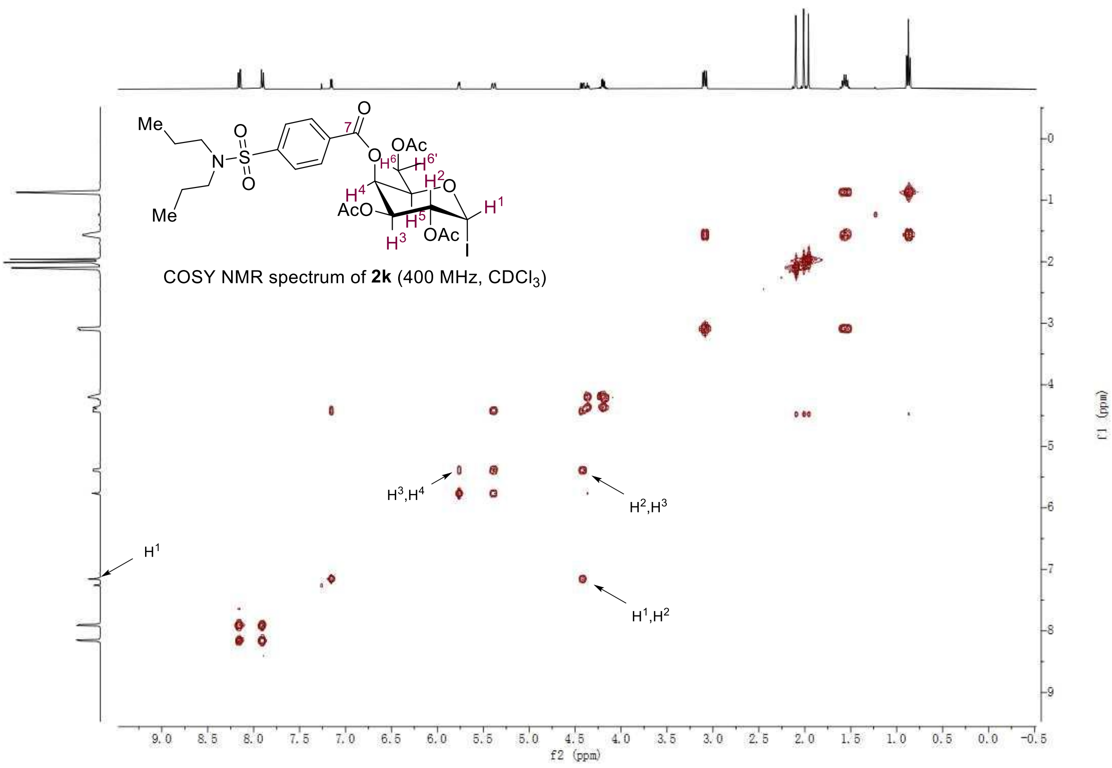
Me
Me
OAc
AcO
OAc
I
H[1]
H[2]
H[3]
H[4]
H[5]
H[6]
H[6']
7
COSY NMR spectrum of 2k (400 MHz, CDCl3)
H[3],H[4]
H[2],H[3]
H[1]
H[1],H[2]
f2 (ppm)
f1 (ppm)

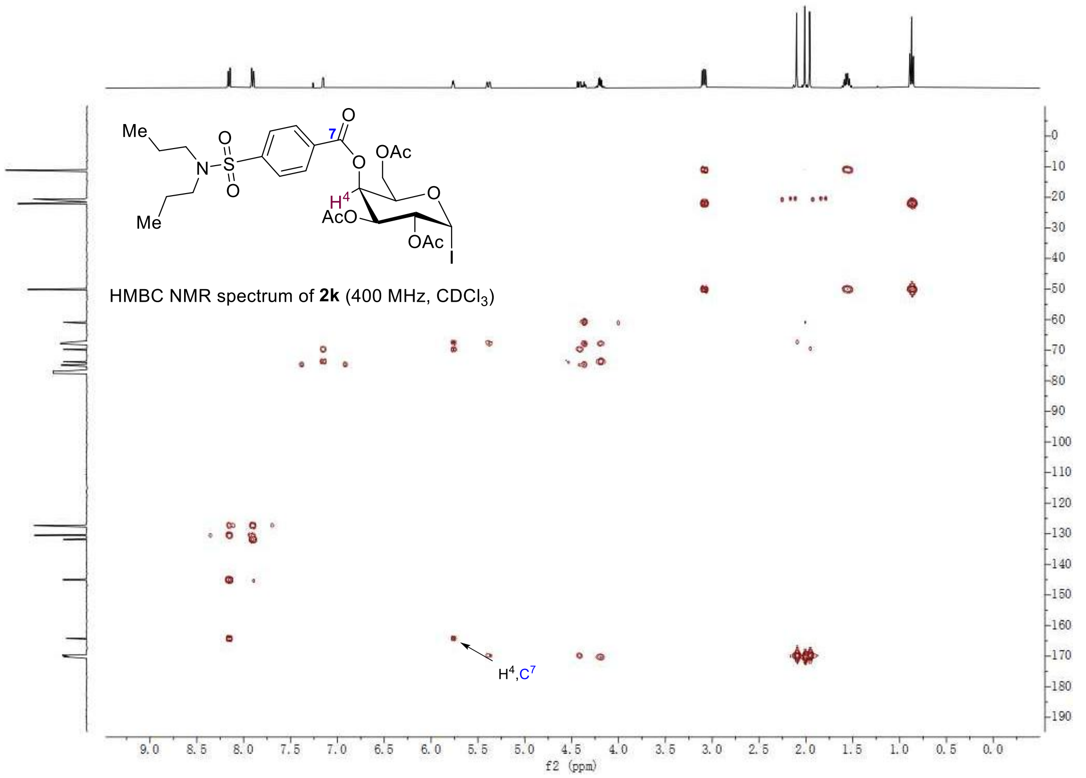
Me
Me
OAc
AcO
OAc
I
H[4]
7
HMBC NMR spectrum of 2k (400 MHz, CDCl3)
H[4],C[7]
f2 (ppm)

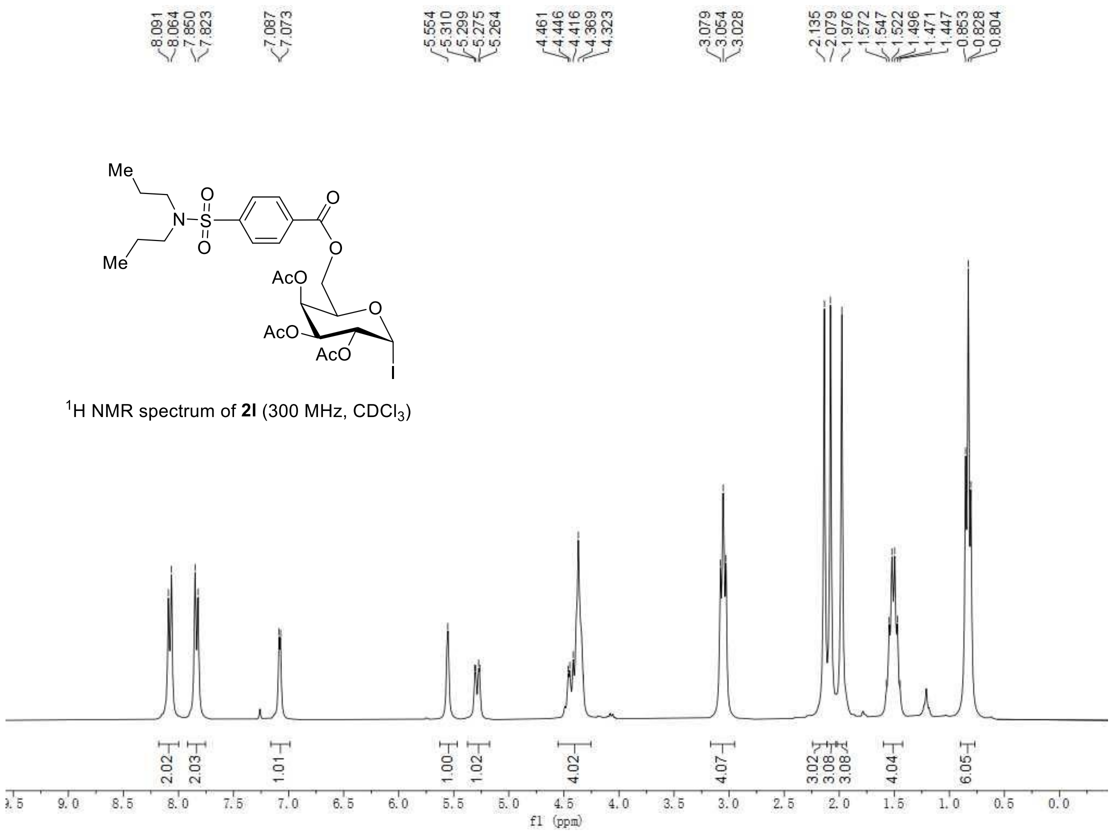

^{1}H NMR spectrum of **2l** (300 MHz, $CDCl_3$)

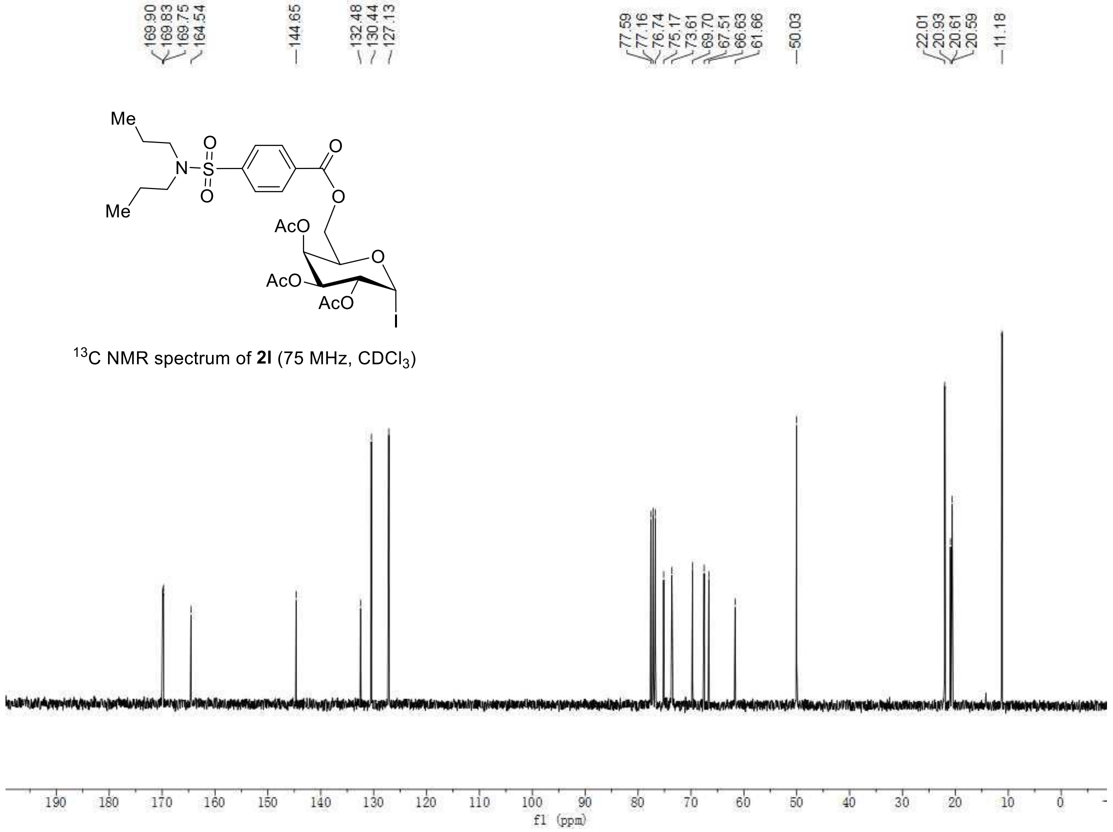

^{13}C NMR spectrum of **2l** (75 MHz, $CDCl_3$)

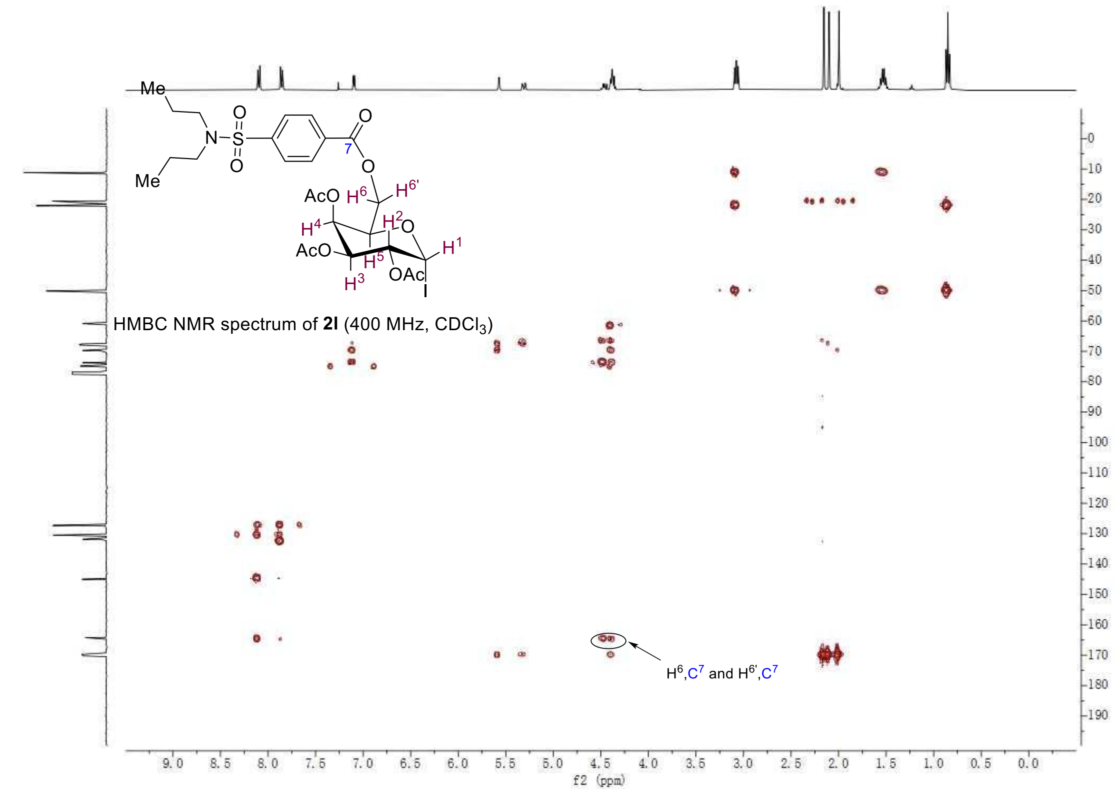

HMBC NMR spectrum of **2l** (400 MHz, $CDCl_3$)

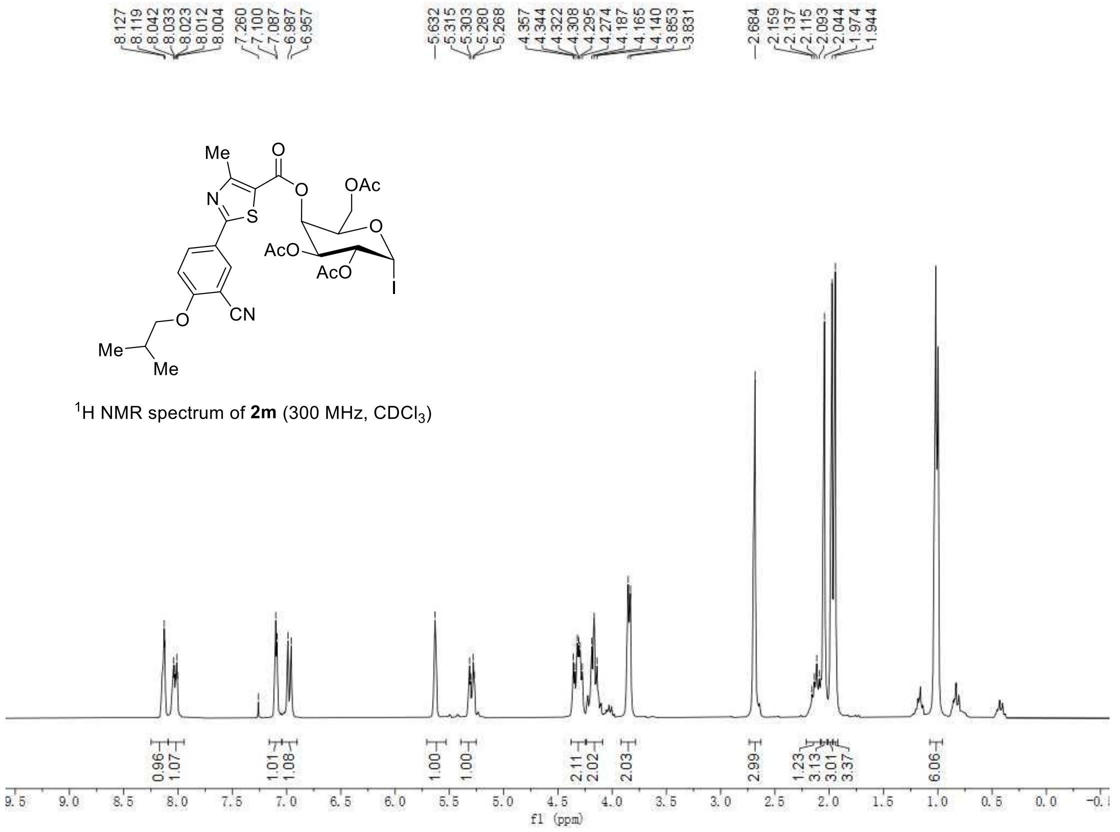

^{1}H NMR spectrum of **2m** (300 MHz, $CDCl_3$)

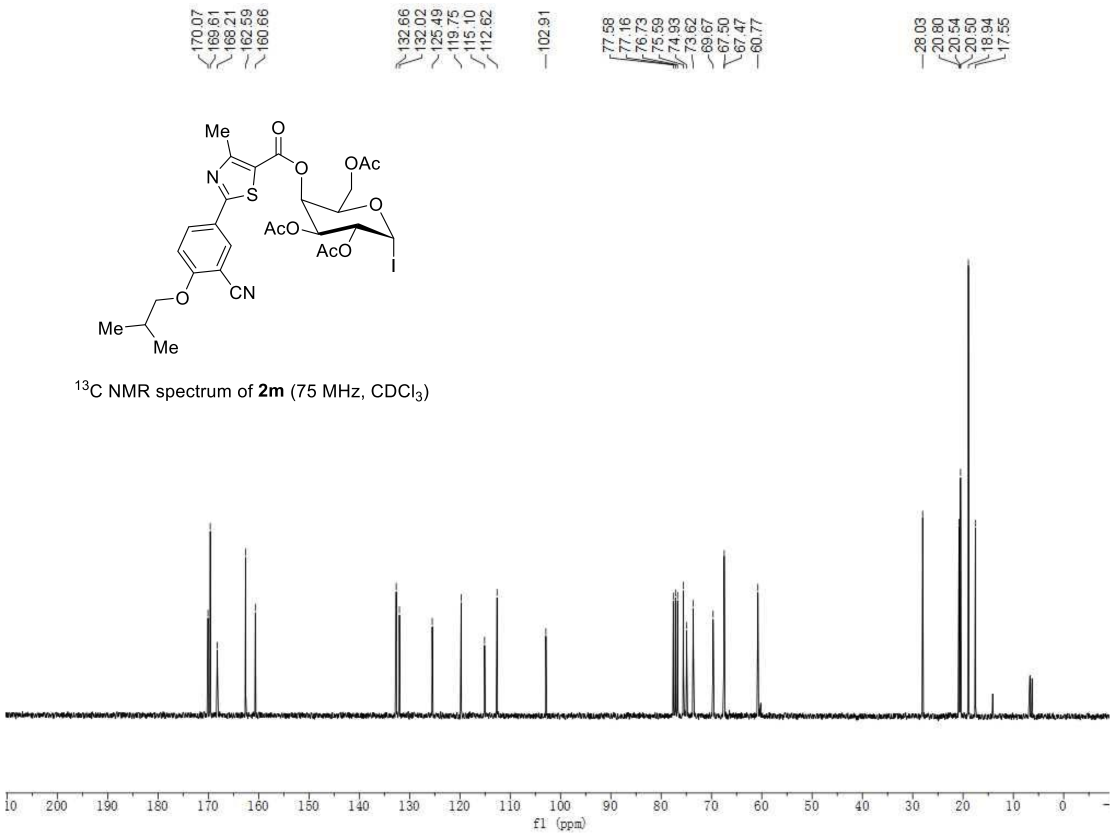

^{13}C NMR spectrum of **2m** (75 MHz, $CDCl_3$)

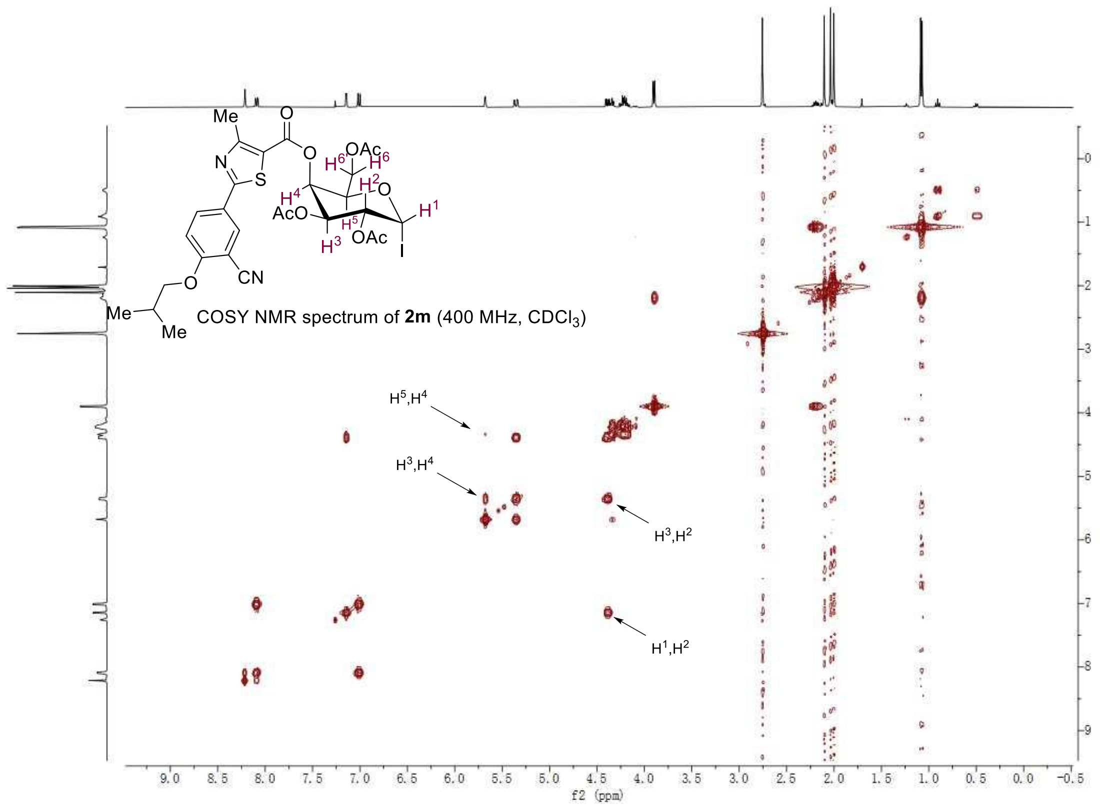

COSY NMR spectrum of **2m** (400 MHz, $CDCl_3$)

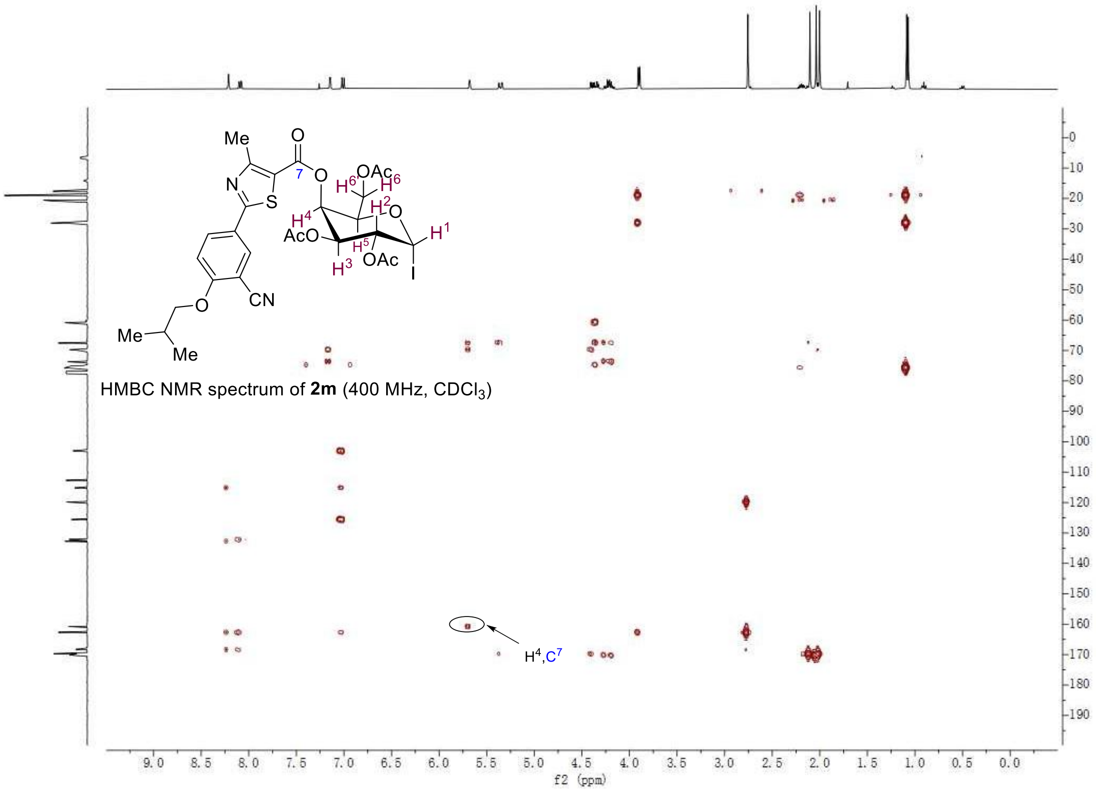

HMBC NMR spectrum of **2m** (400 MHz, $CDCl_3$)

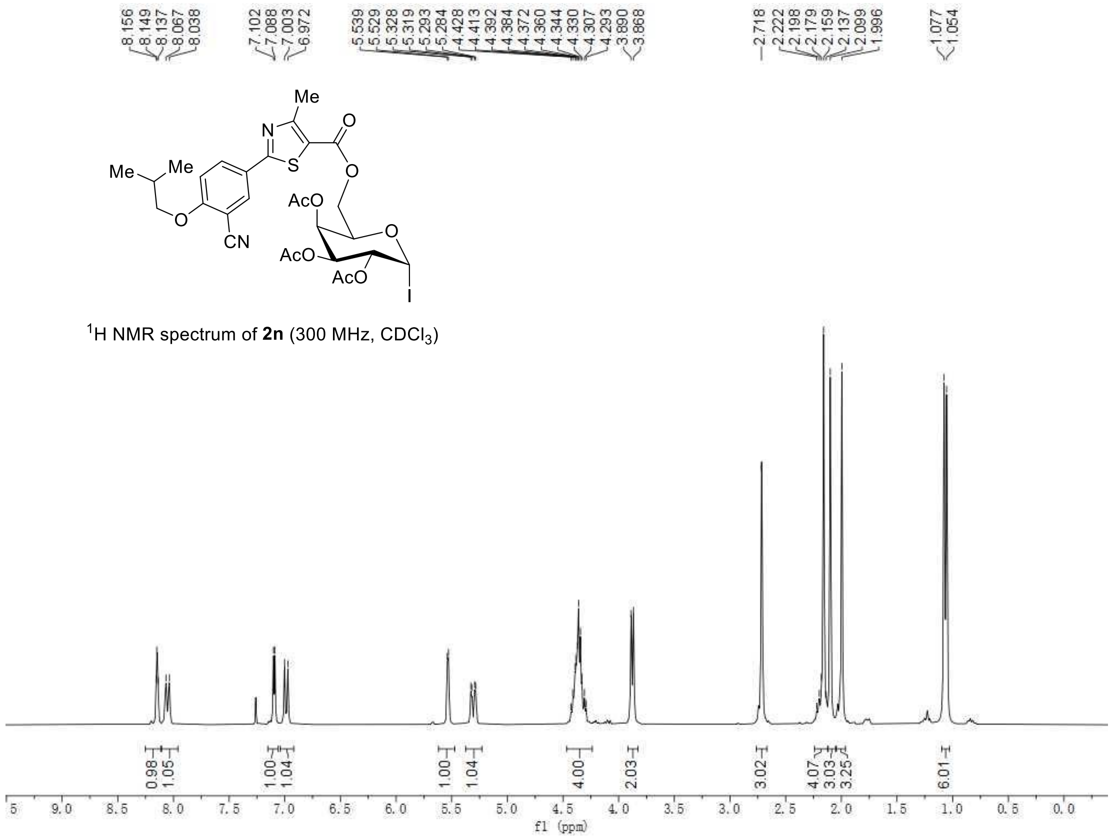

^1H NMR spectrum of **2n** (300 MHz, $CDCl_3$)

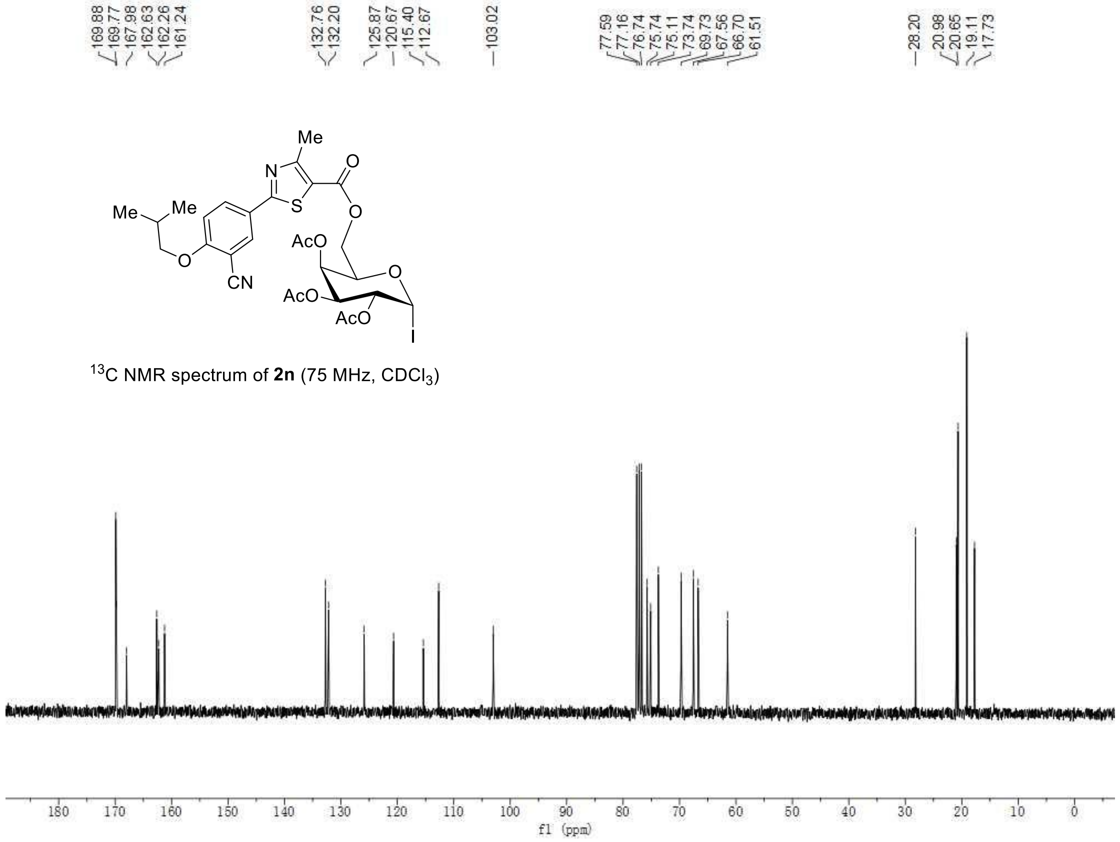

^{13}C NMR spectrum of **2n** (75 MHz, $CDCl_3$)

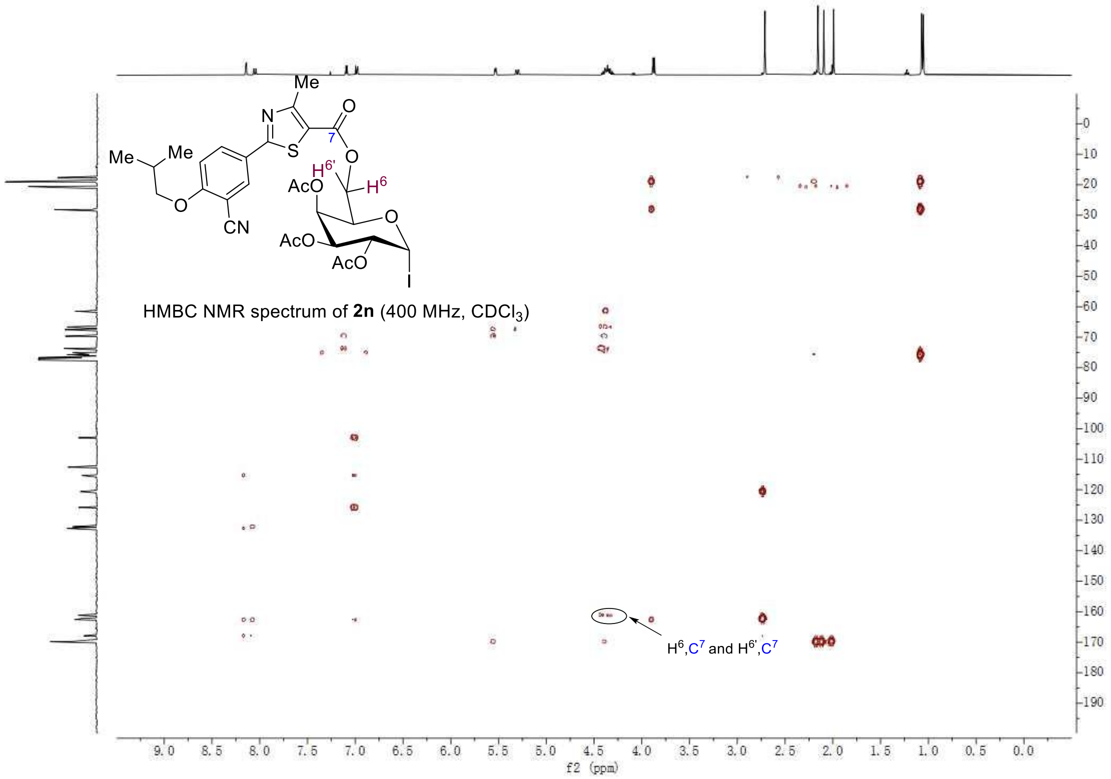

HMBC NMR spectrum of **2n** (400 MHz, $CDCl_3$)

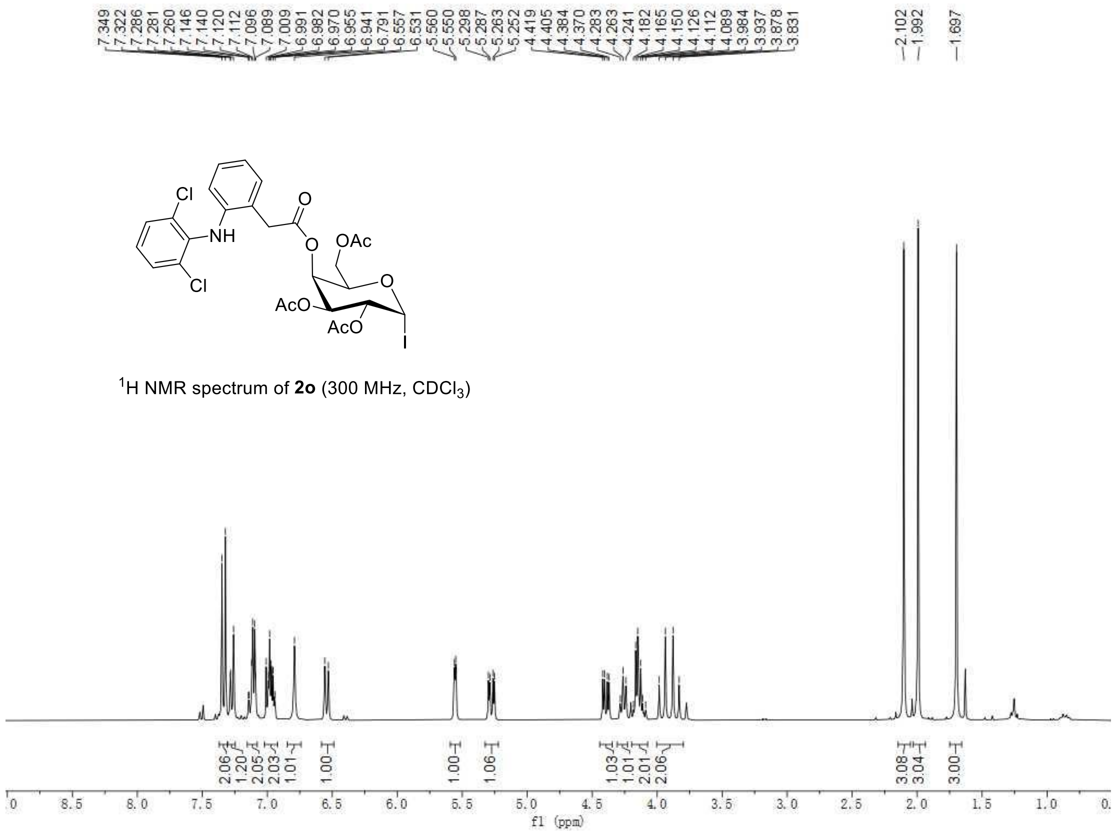

^{1}H NMR spectrum of **2o** (300 MHz, $CDCl_3$)

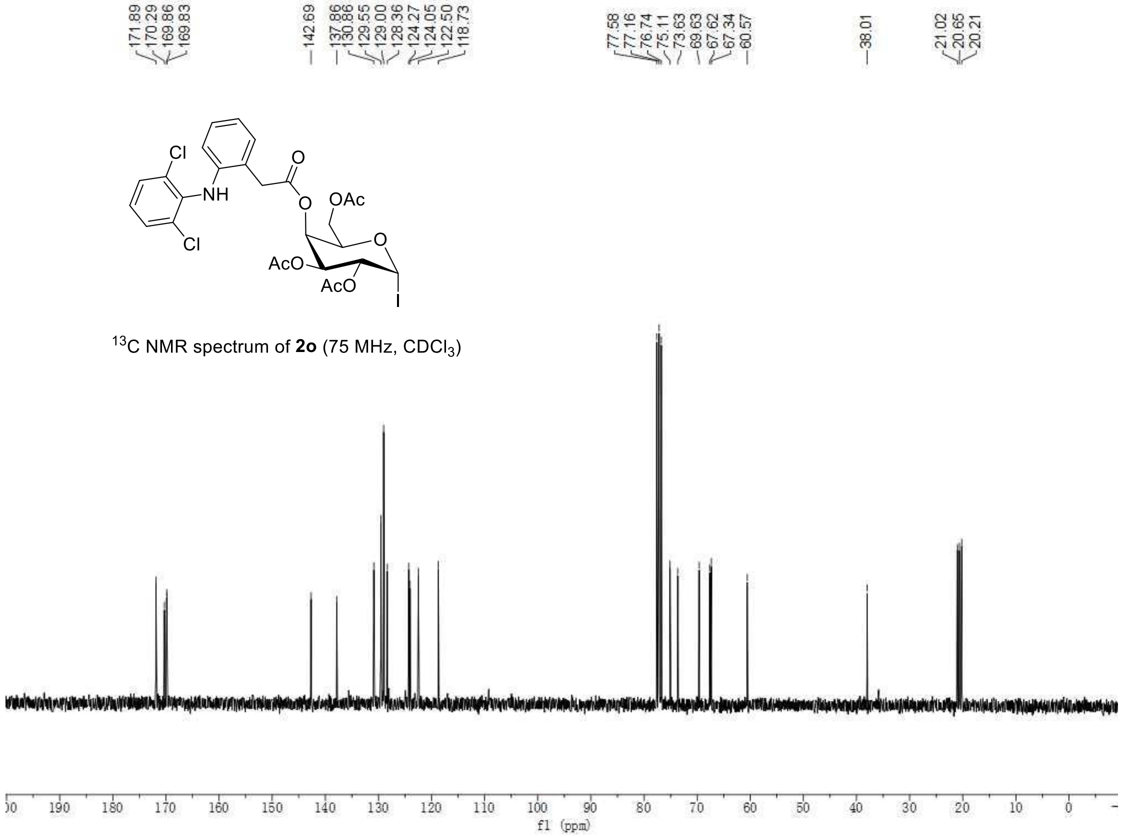

^{13}C NMR spectrum of **2o** (75 MHz, $CDCl_3$)

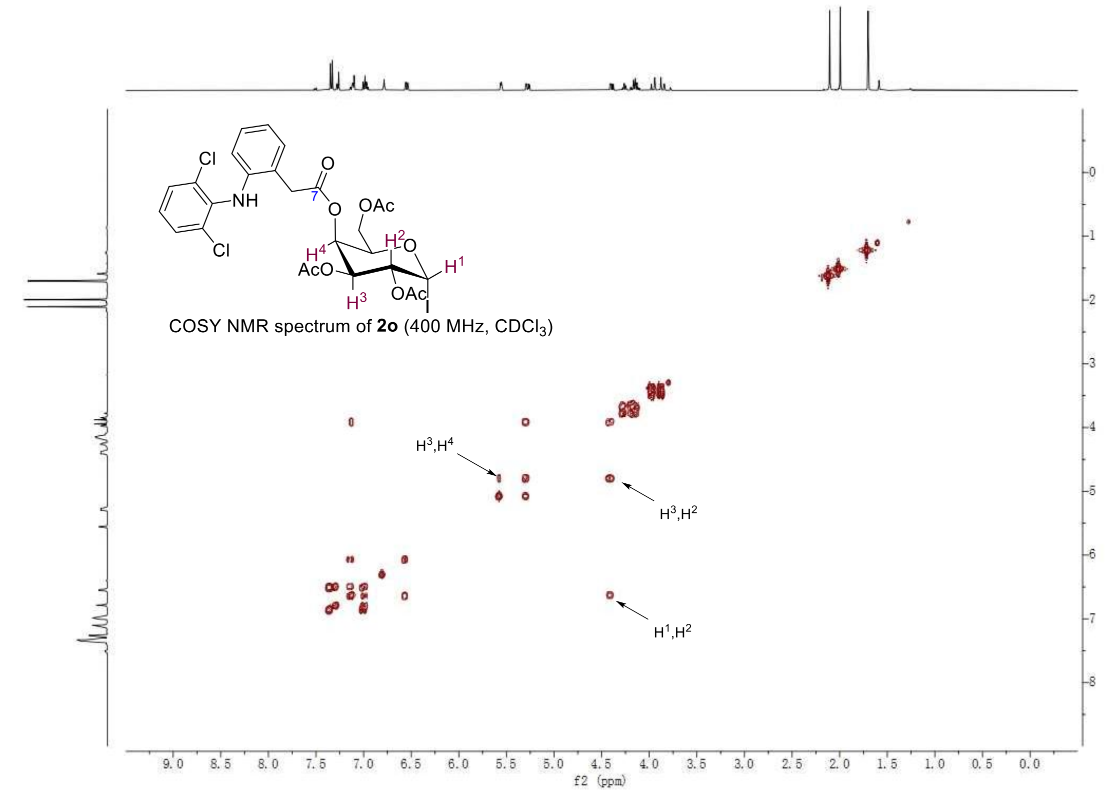

COSY NMR spectrum of **2o** (400 MHz, $CDCl_3$)

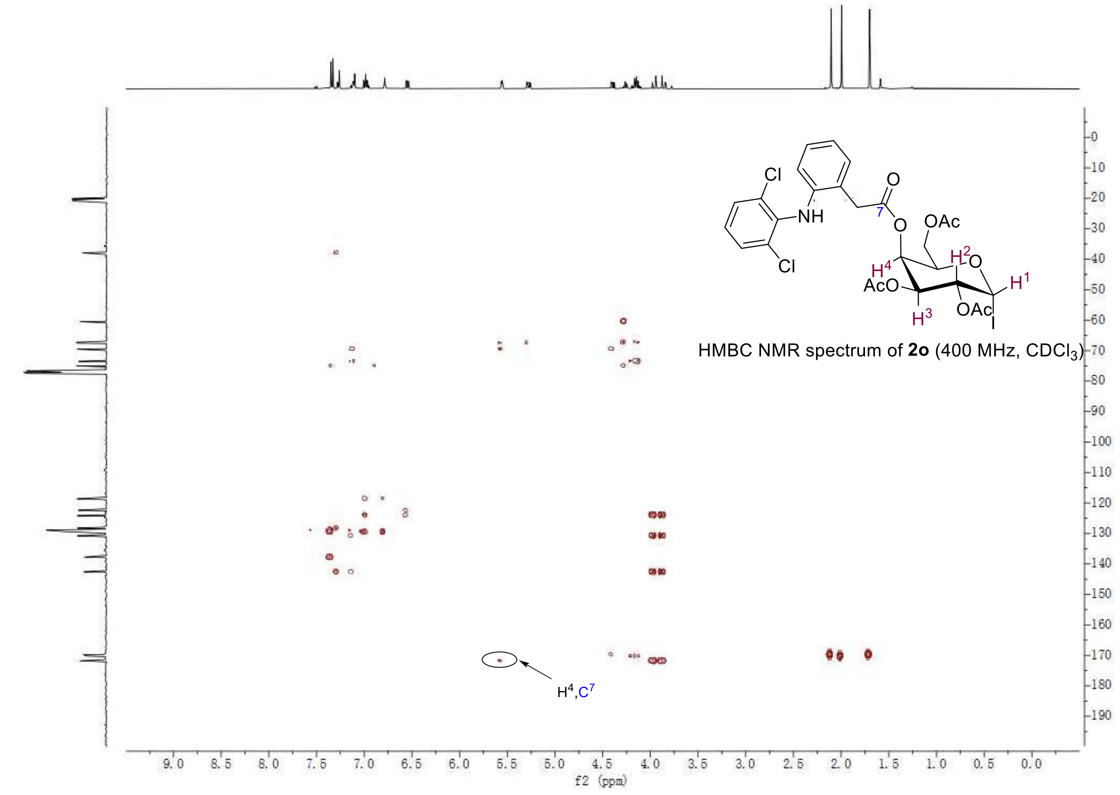

HMBC NMR spectrum of **2o** (400 MHz, $CDCl_3$)

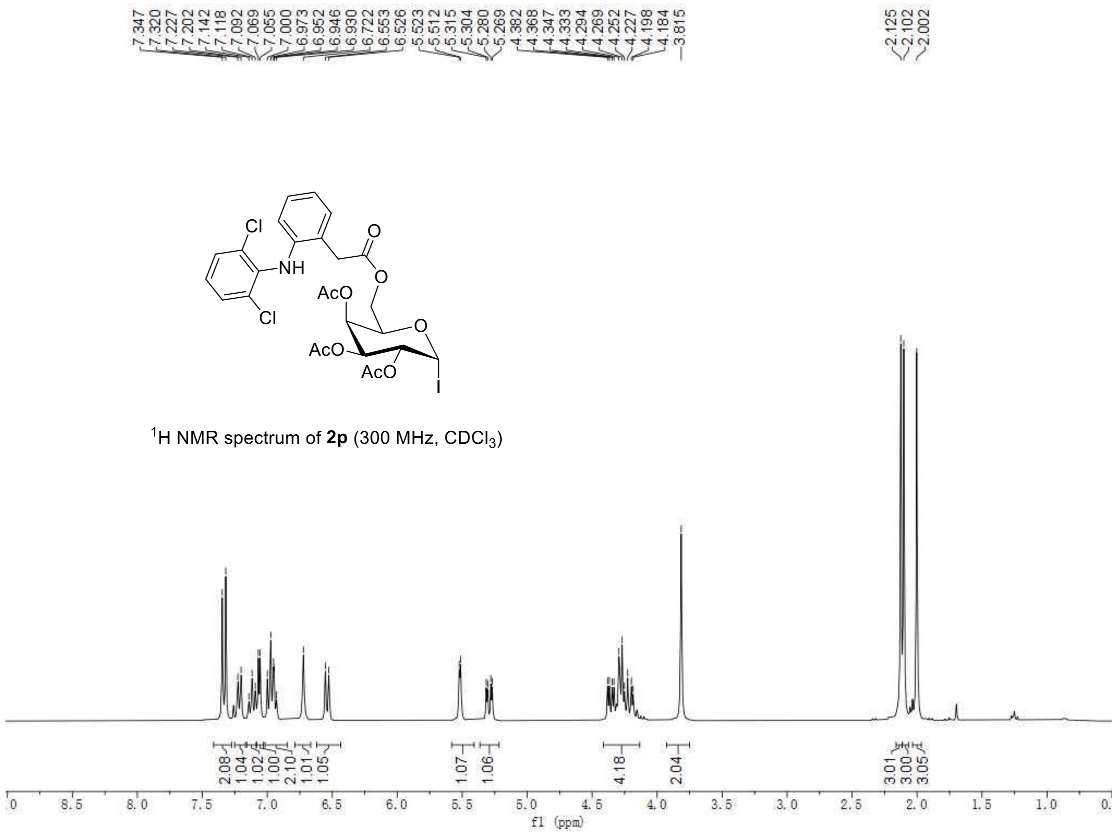

^{1}H NMR spectrum of **2p** (300 MHz, $CDCl_3$)

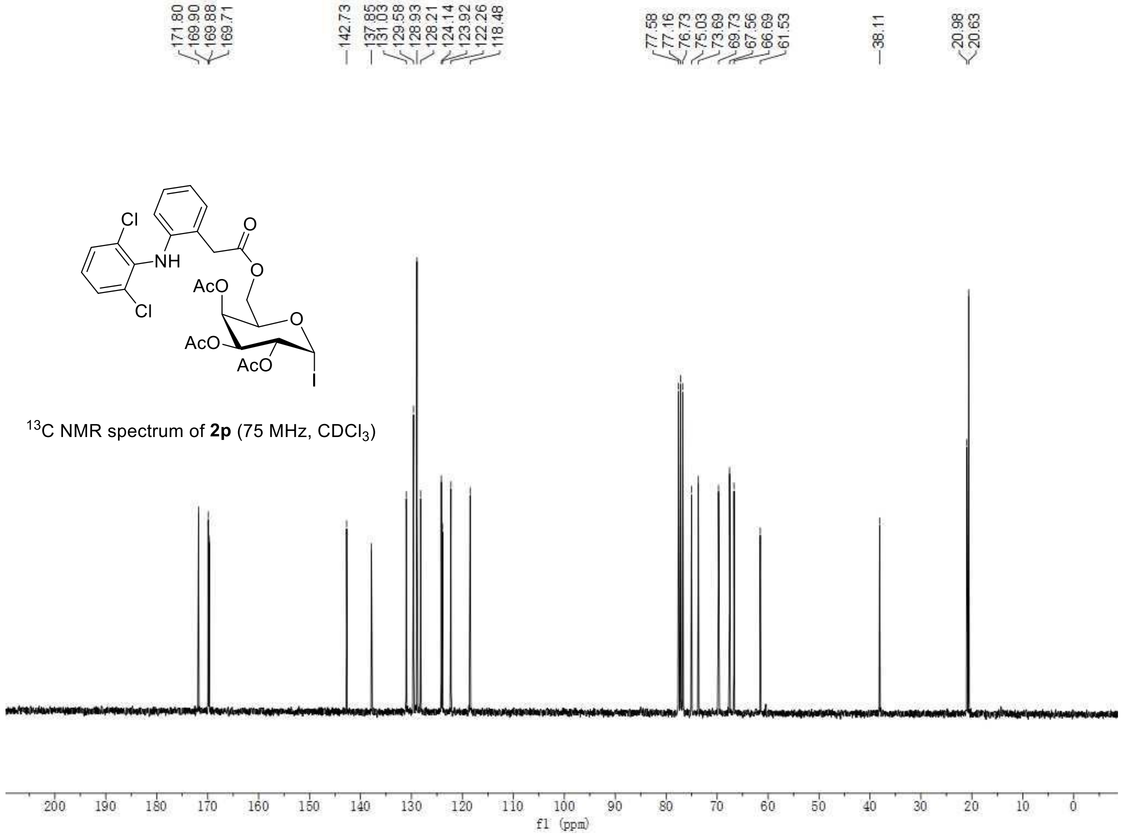

^{13}C NMR spectrum of **2p** (75 MHz, $CDCl_3$)

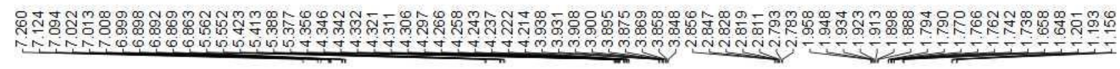

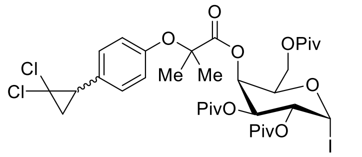

^{1}H NMR spectrum of **2q** (300 MHz, $CDCl_3$)

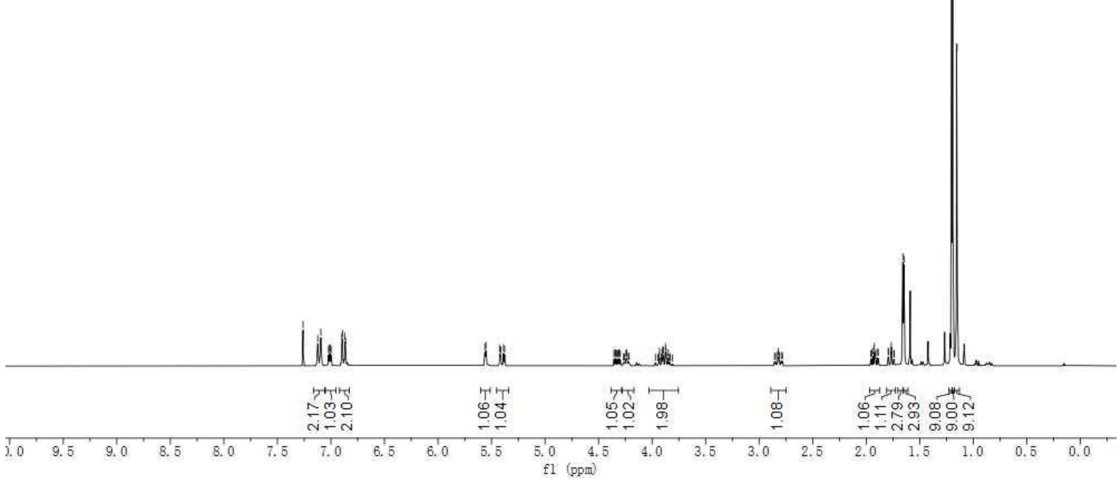

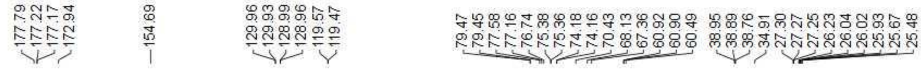

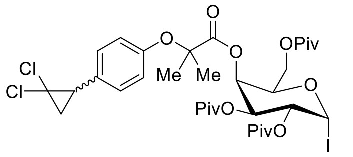

^{13}C NMR spectrum of **2q** (75 MHz, $CDCl_3$)

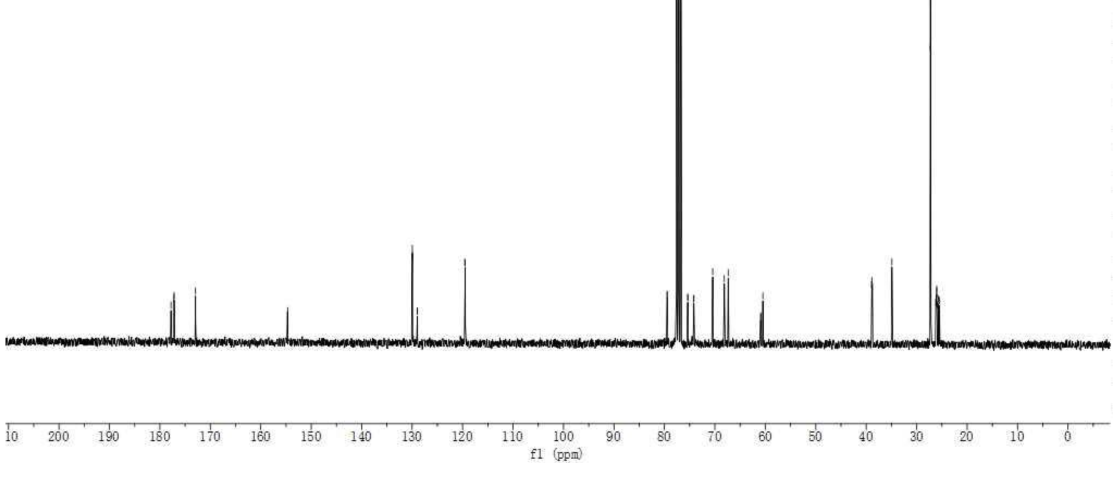

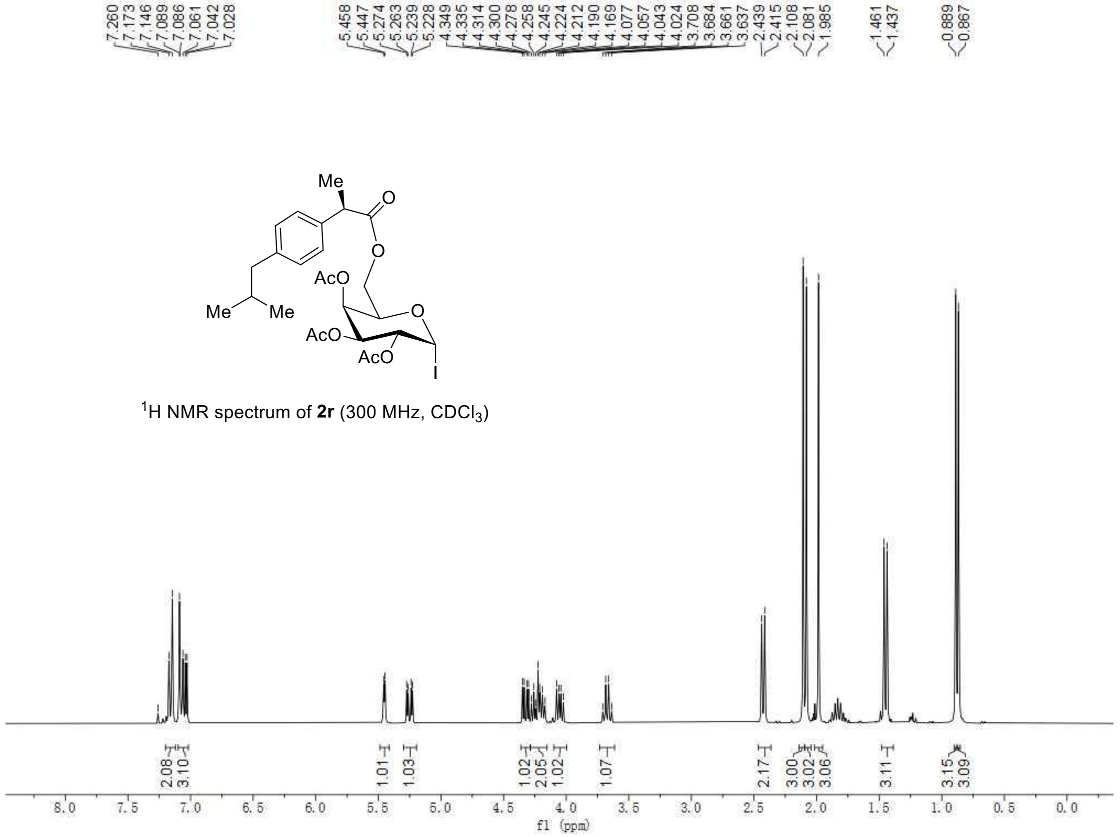

^{1}H NMR spectrum of **2r** (300 MHz, $CDCl_3$)

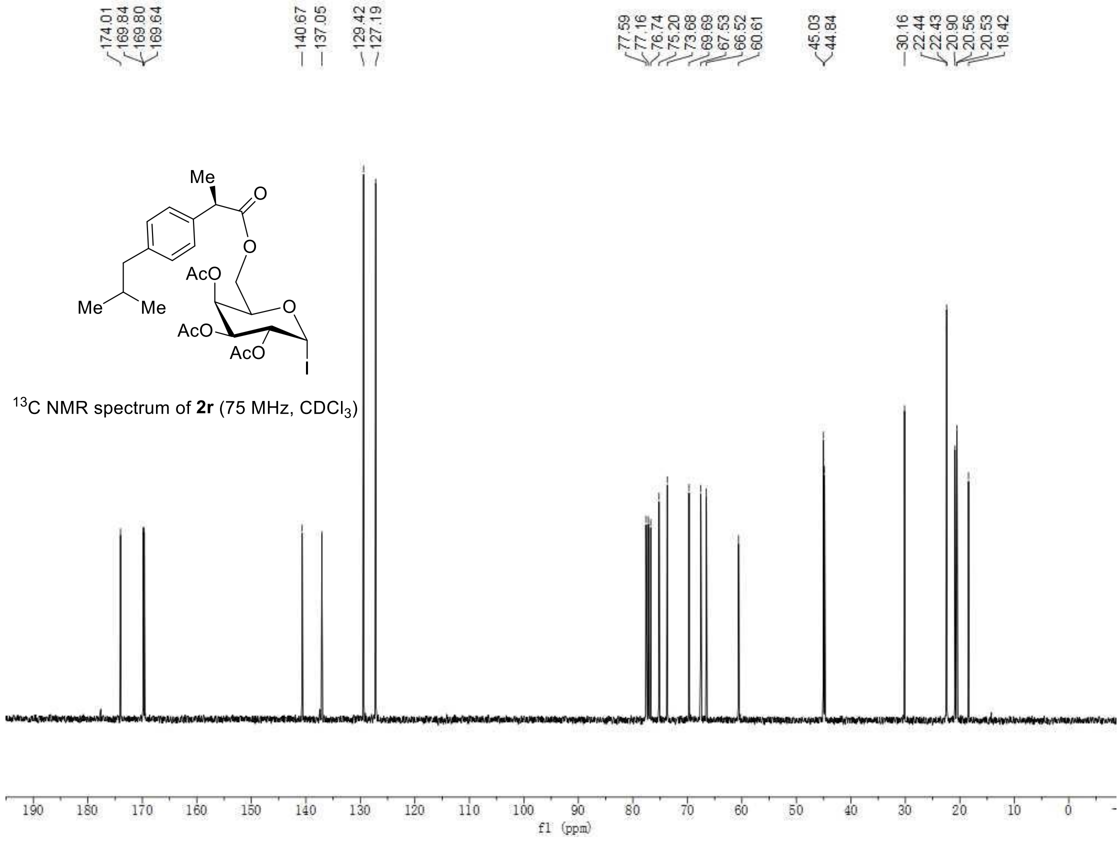

^{13}C NMR spectrum of **2r** (75 MHz, $CDCl_3$)

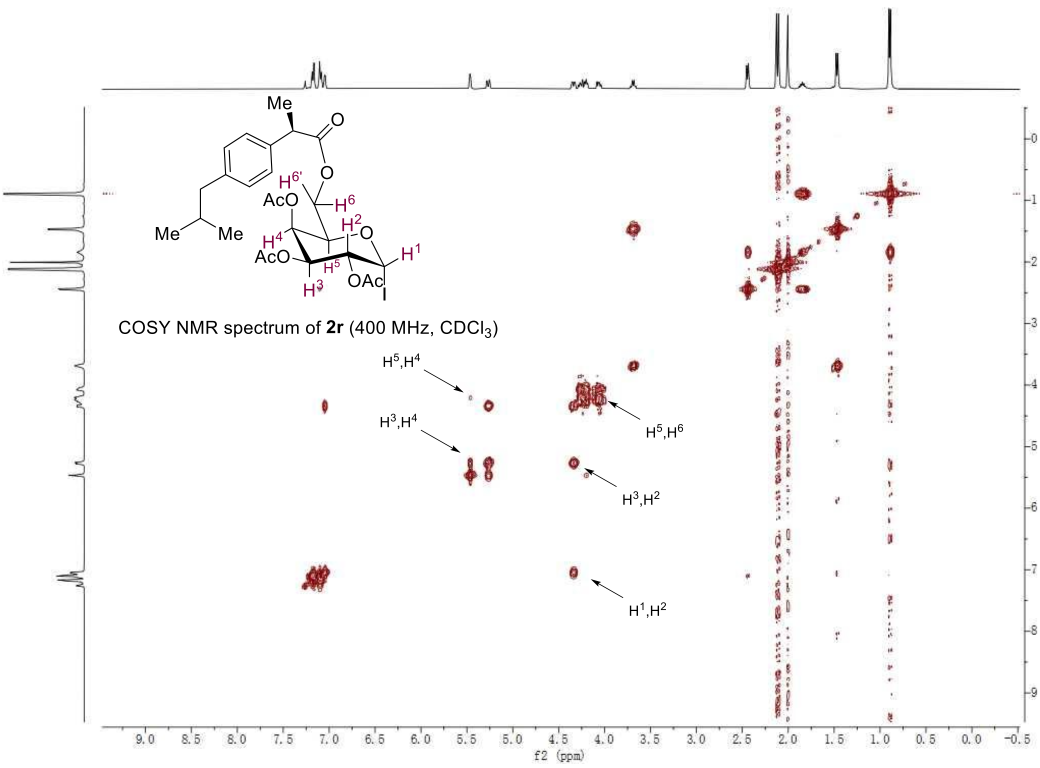
Me
O
O
H6'
H6
AcO
H2
O
Me
Me
H4
H1
AcO
H5
OAc
H3
I
COSY NMR spectrum of 2r (400 MHz, CDCl3)
H5,H4
H3,H4
H5,H6
H3,H2
H1,H2
f2 (ppm)
f1 (ppm)

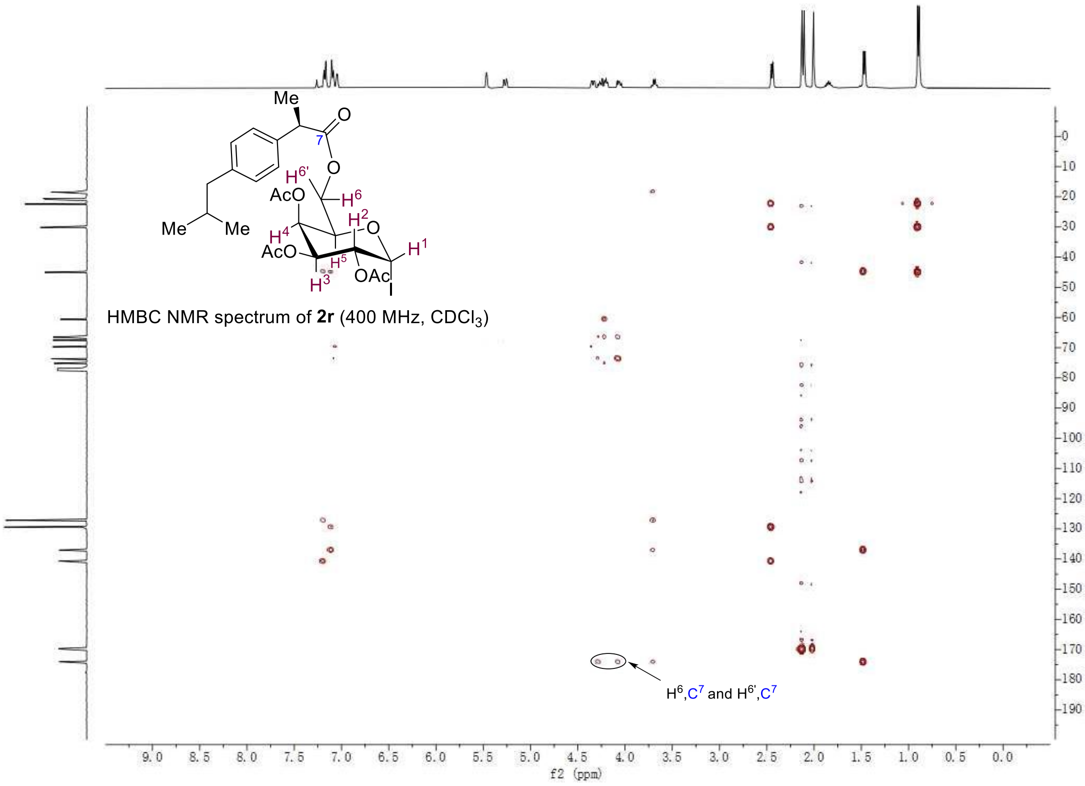
Me
O
7
O
H6'
H6
AcO
H2
O
Me
Me
H4
H1
AcO
H5
OAc
H3
I
HMBC NMR spectrum of 2r (400 MHz, CDCl3)
H6,C7 and H6',C7
f2 (ppm)

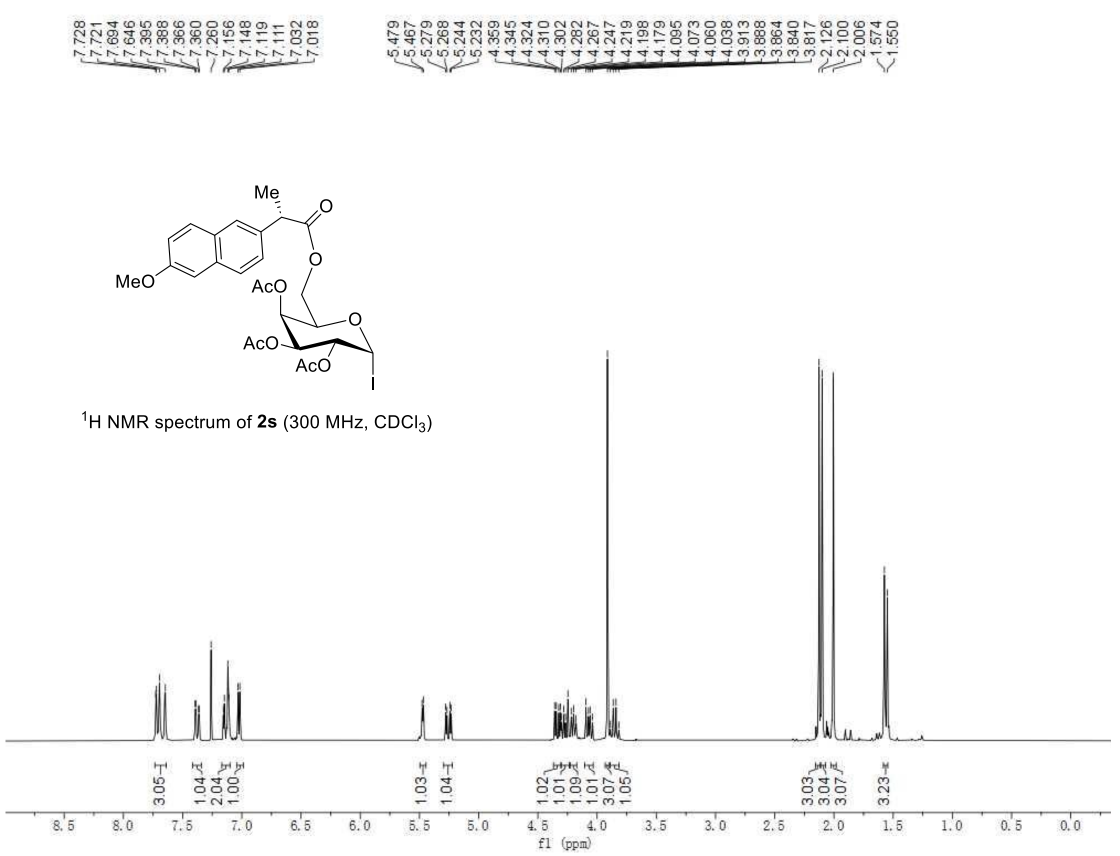

^{1}H NMR spectrum of **2s** (300 MHz, $CDCl_3$)

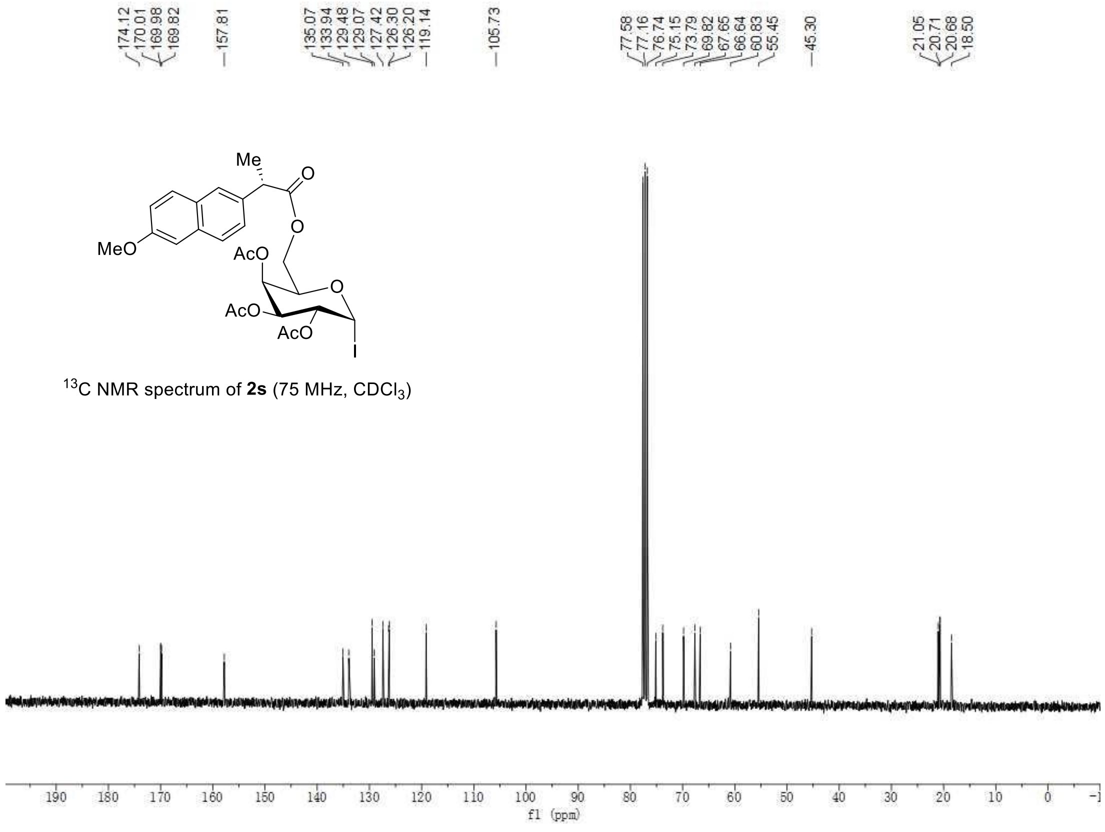

^{13}C NMR spectrum of **2s** (75 MHz, $CDCl_3$)

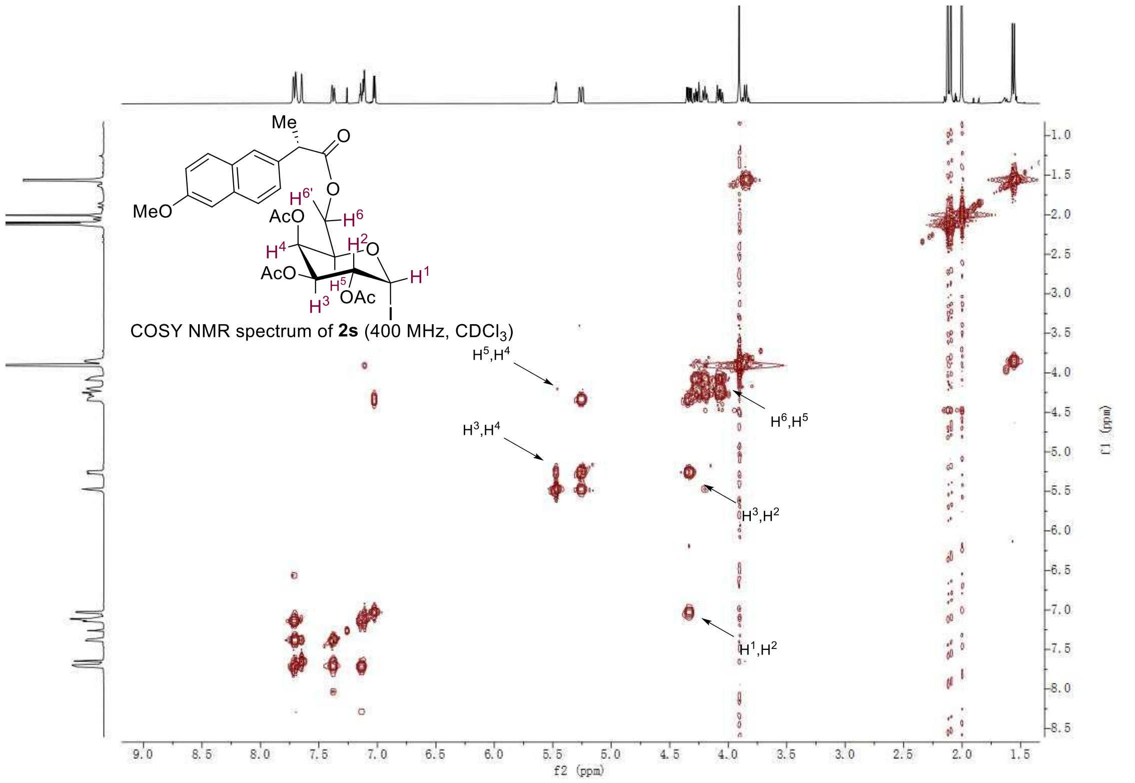

Me
O
MeO
H6'
O
H6
AcO
H4
H2
O
AcO
H5
OAc
H1
H3
COSY NMR spectrum of 2s (400 MHz, CDCl3)
H5,H4
H6,H5
H3,H4
H3,H2
H1,H2
f2 (ppm)
f1 (ppm)

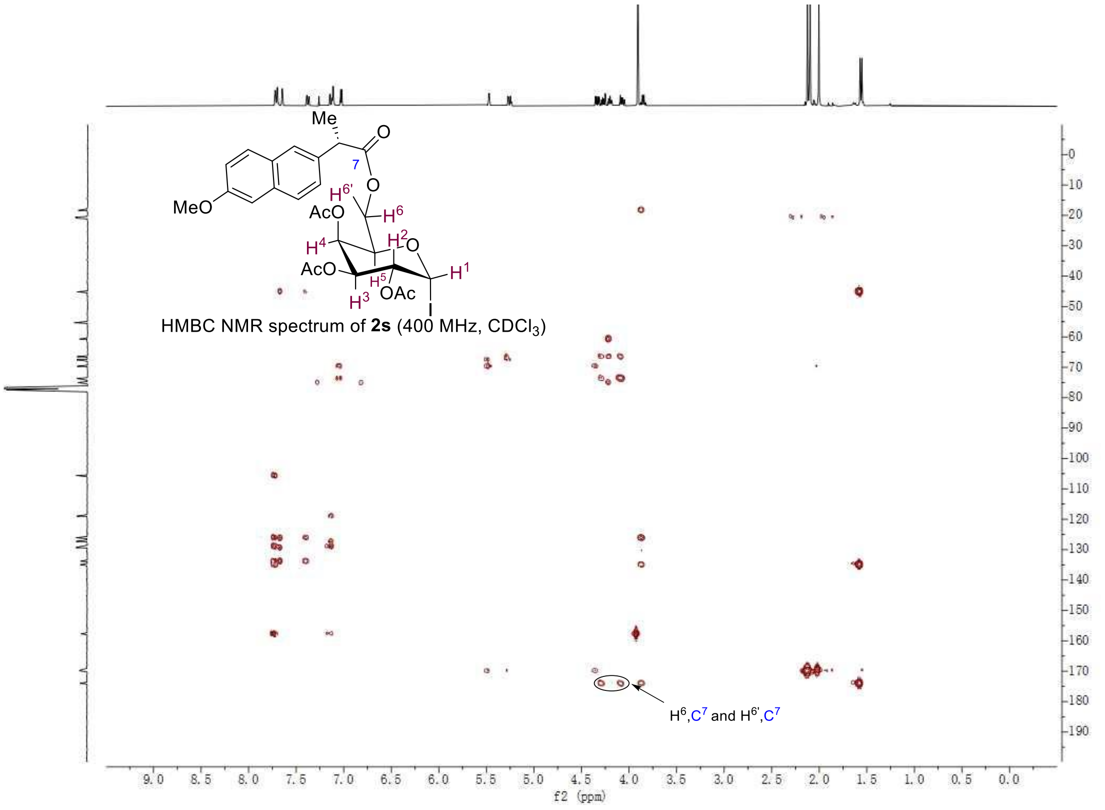

Me
O
7
MeO
O
H6'
H6
AcO
H4
H2
O
AcO
H5
H1
OAc
H3
HMBC NMR spectrum of 2s (400 MHz, CDCl3)
H6,C7 and H6',C7
f2 (ppm)

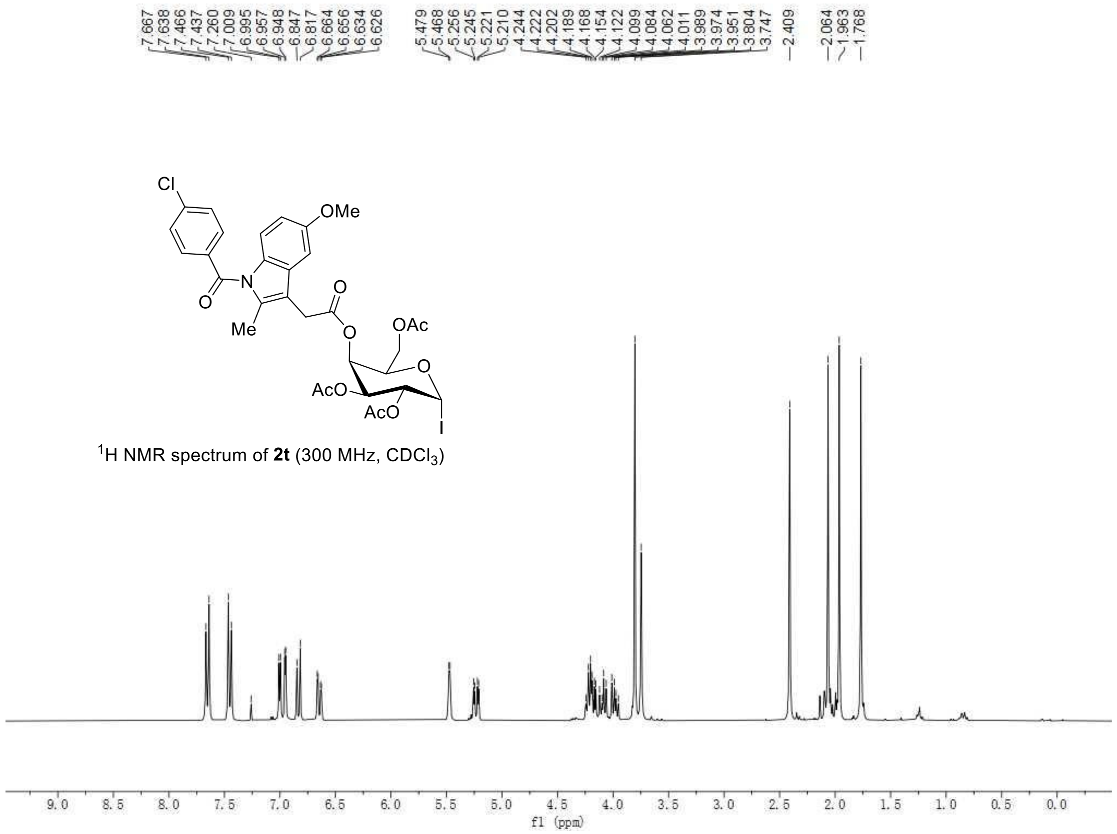

^{1}H NMR spectrum of **2t** (300 MHz, $CDCl_3$)

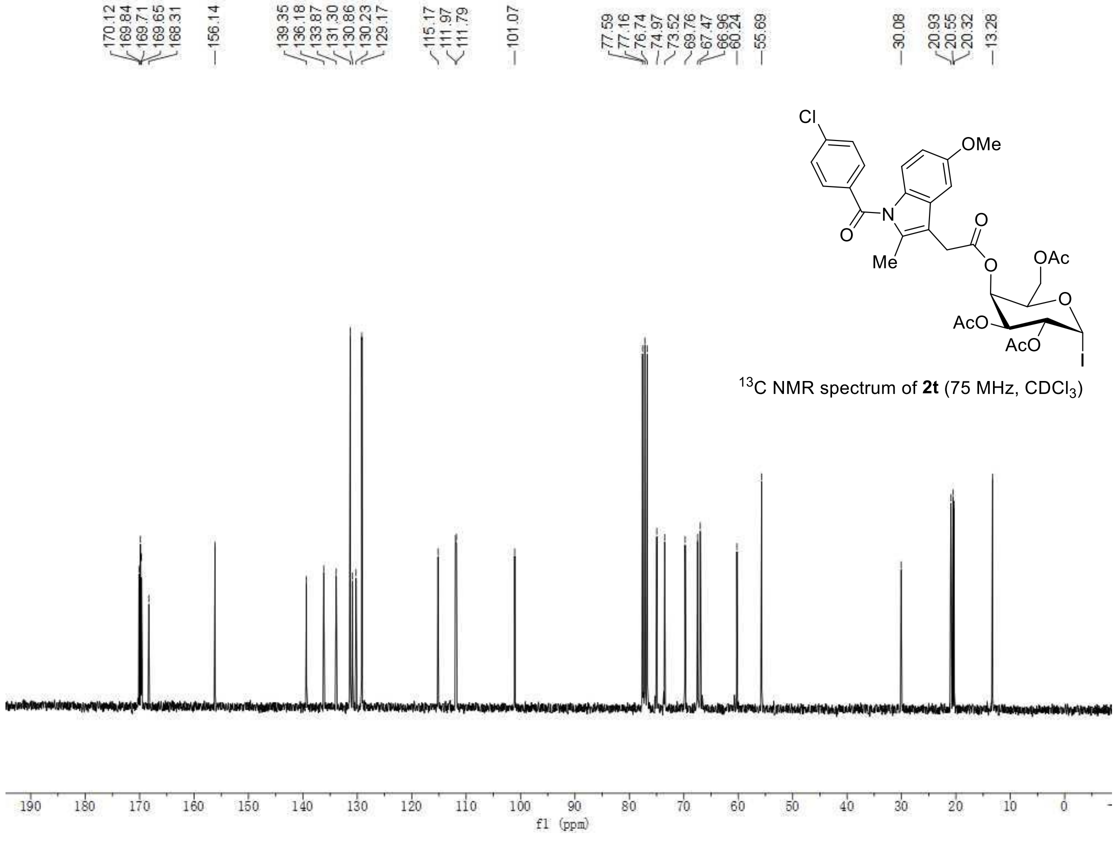

^{13}C NMR spectrum of **2t** (75 MHz, $CDCl_3$)

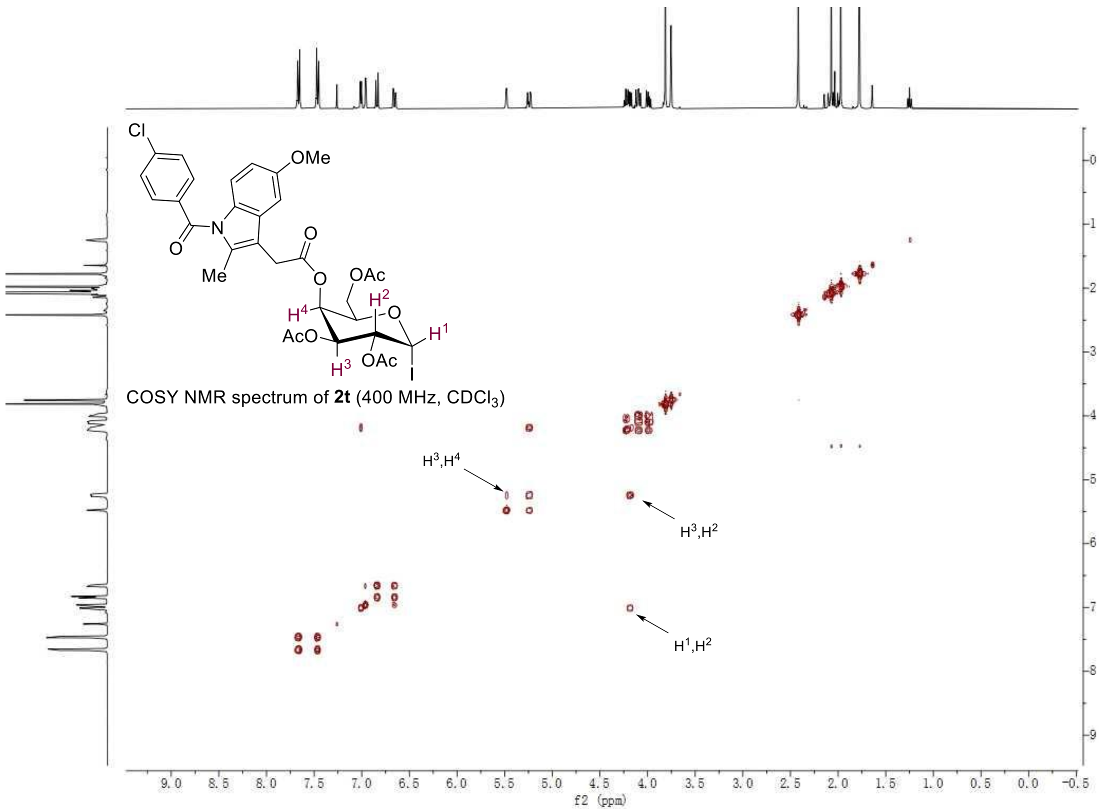

COSY NMR spectrum of **2t** (400 MHz, $CDCl_3$)

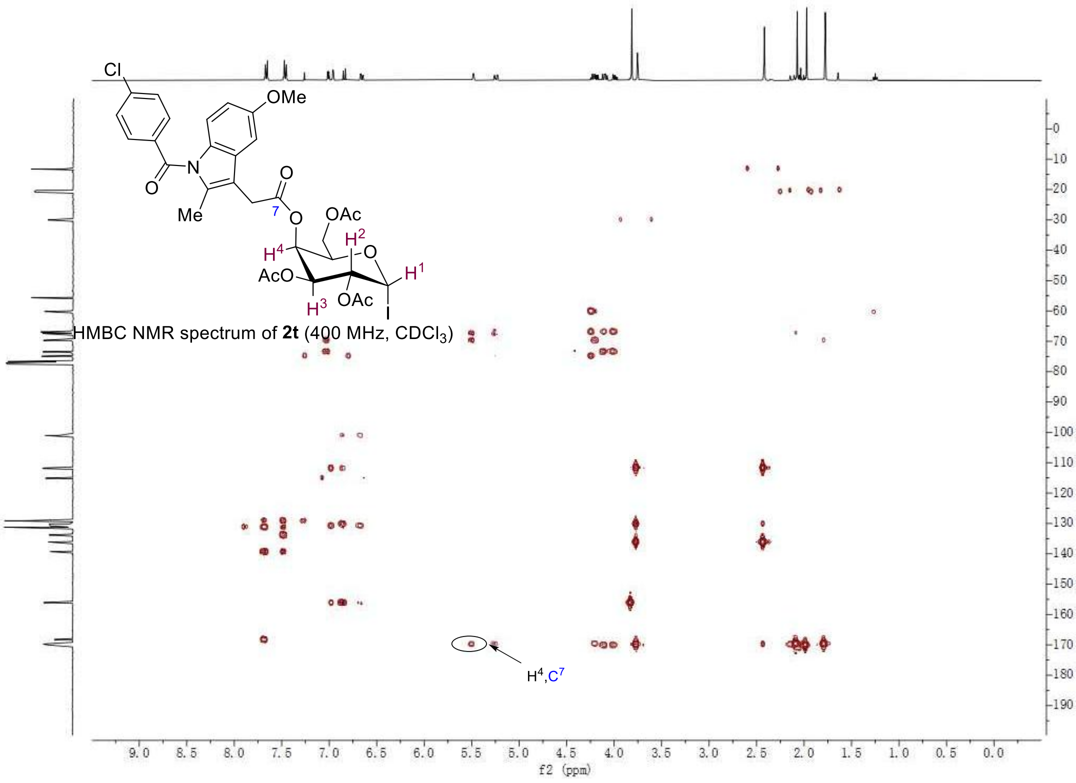

HMBC NMR spectrum of **2t** (400 MHz, $CDCl_3$)

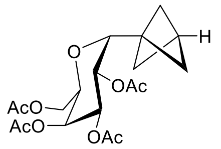

^{1}H NMR spectrum of **3** (600 MHz, $CDCl_3$)

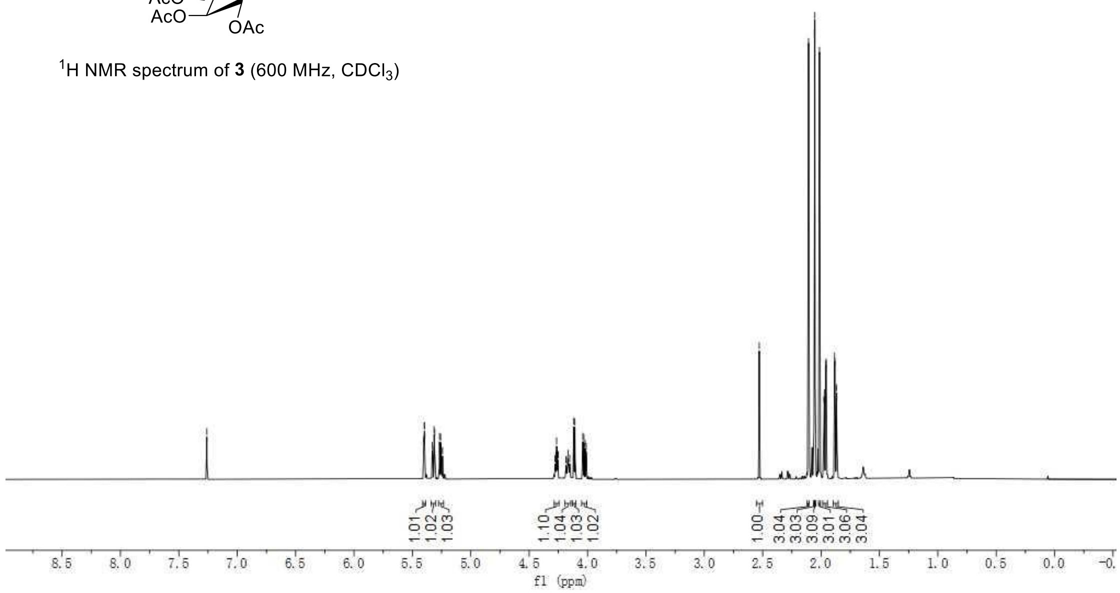

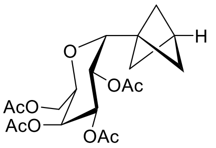

^{13}C NMR spectrum of **3** (126 MHz, $CDCl_3$)

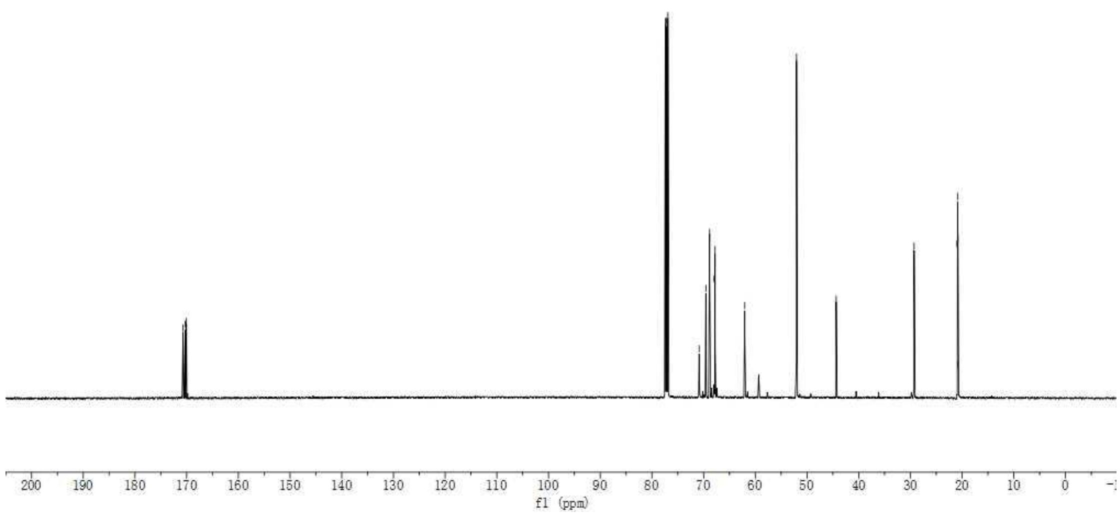

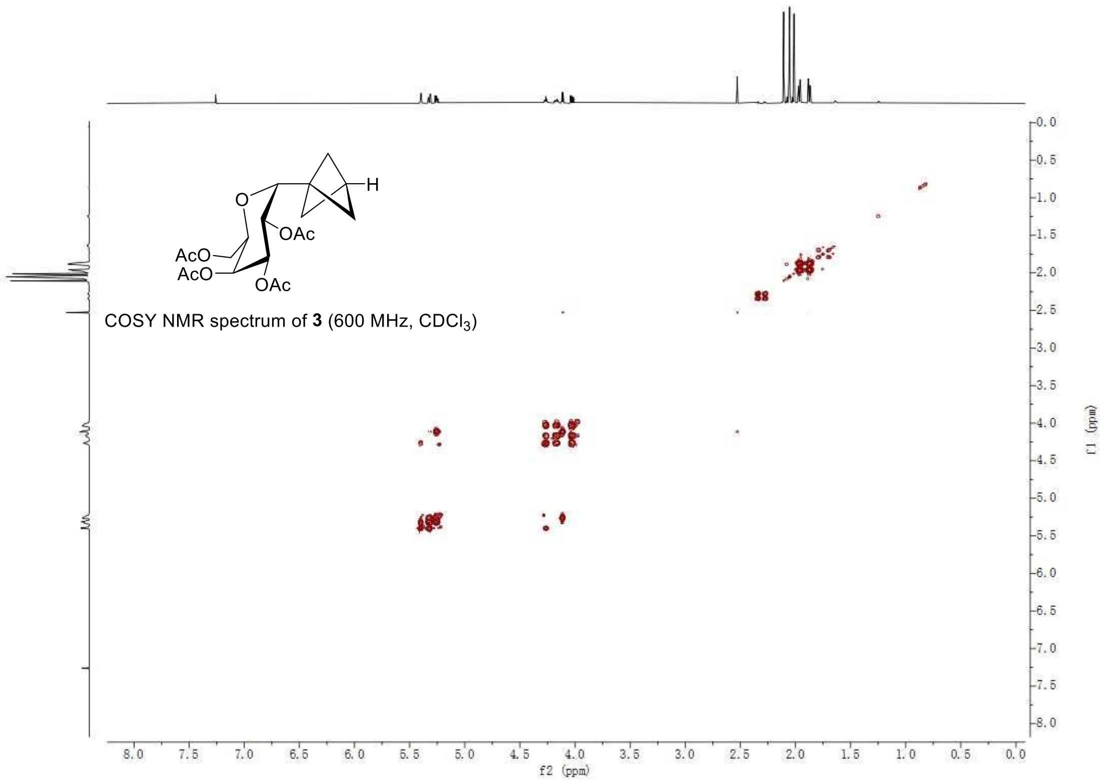
O
AcO
AcO
OAc
OAc
H
COSY NMR spectrum of 3 (600 MHz, CDCl3)
f2 (ppm)
f1 (ppm)

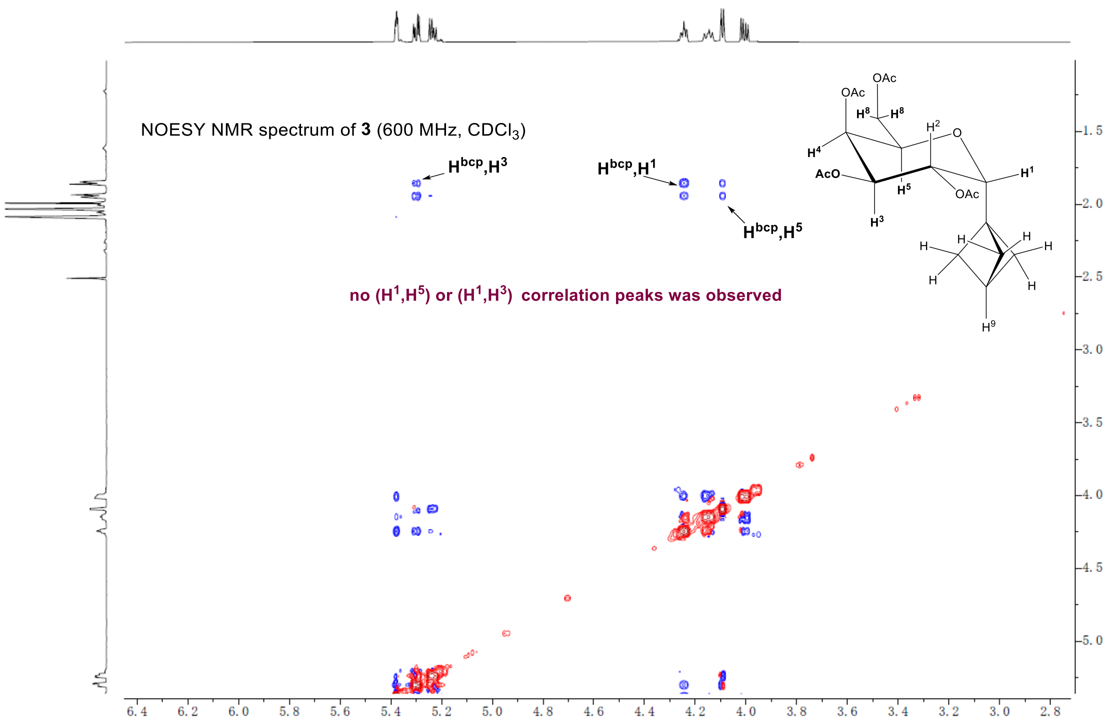
NOESY NMR spectrum of 3 (600 MHz, CDCl3)
Hbcp,H3
Hbcp,H1
Hbcp,H5
no (H1,H5) or (H1,H3) correlation peaks was observed
OAc
OAc
H8
H8
H2
O
H4
AcO
H5
OAc
H1
H3
H9

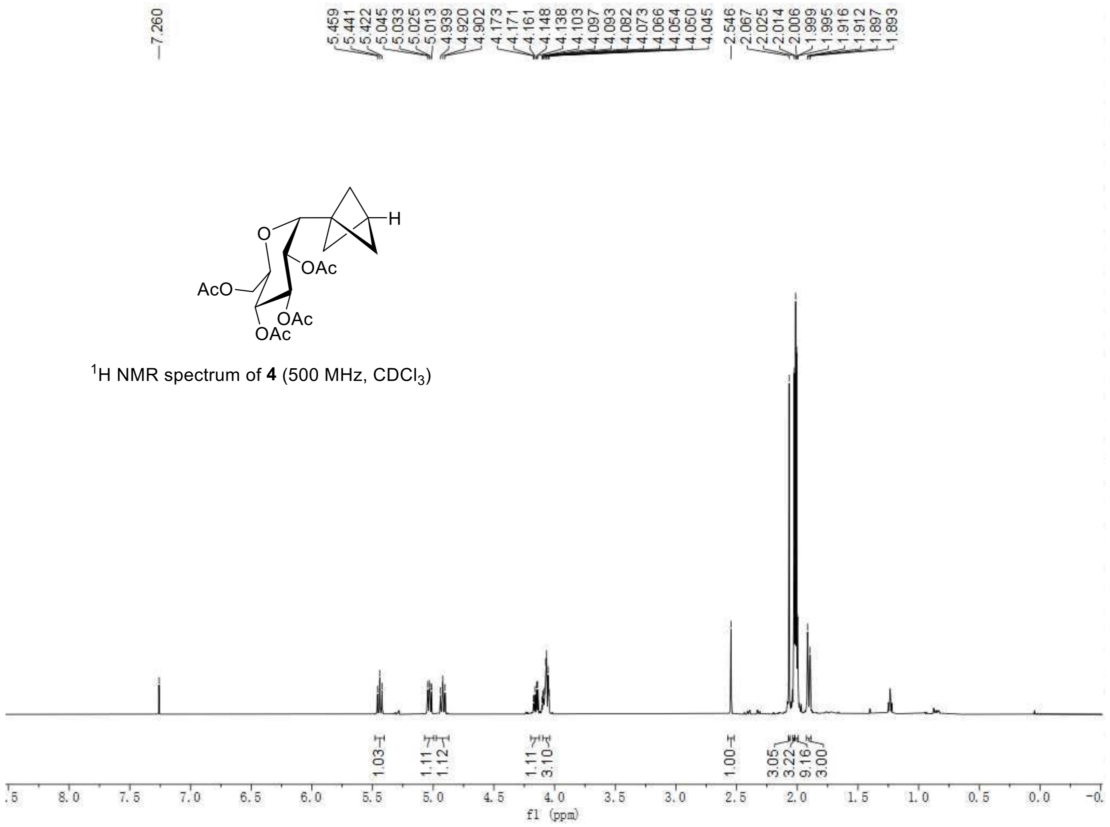

^{1}H NMR spectrum of **4** (500 MHz, $CDCl_3$)

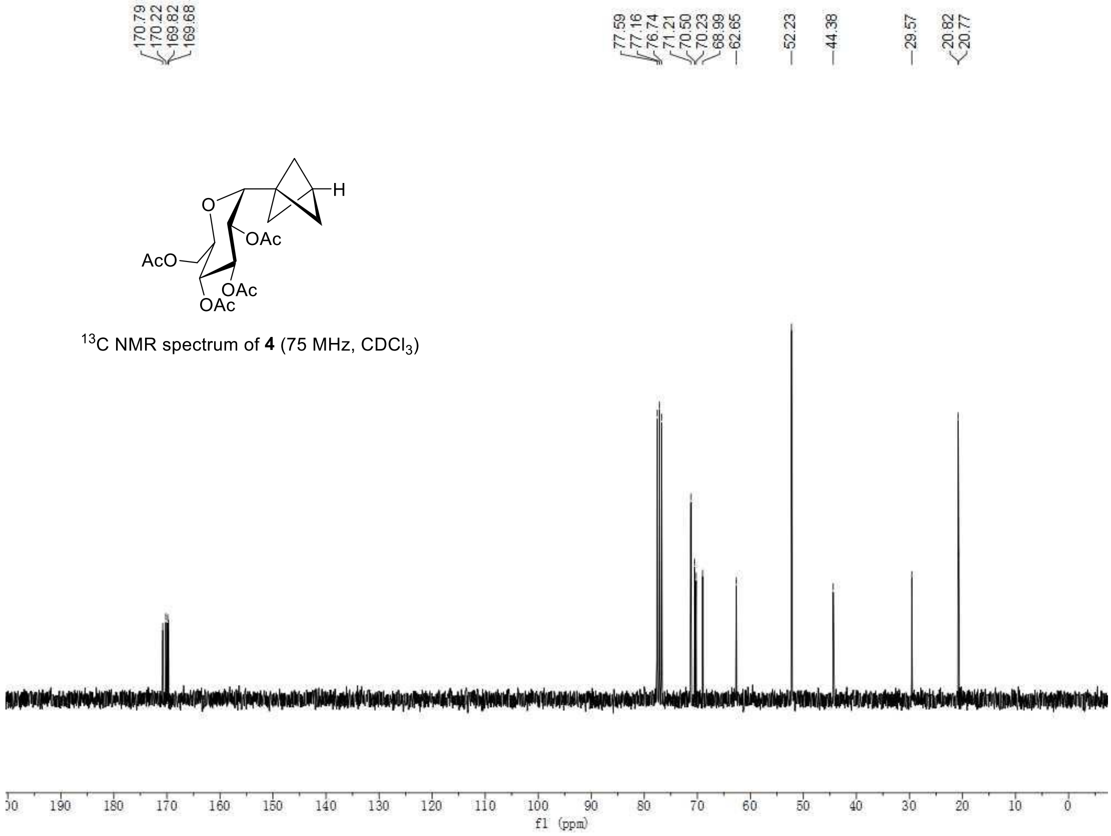

^{13}C NMR spectrum of **4** (75 MHz, $CDCl_3$)

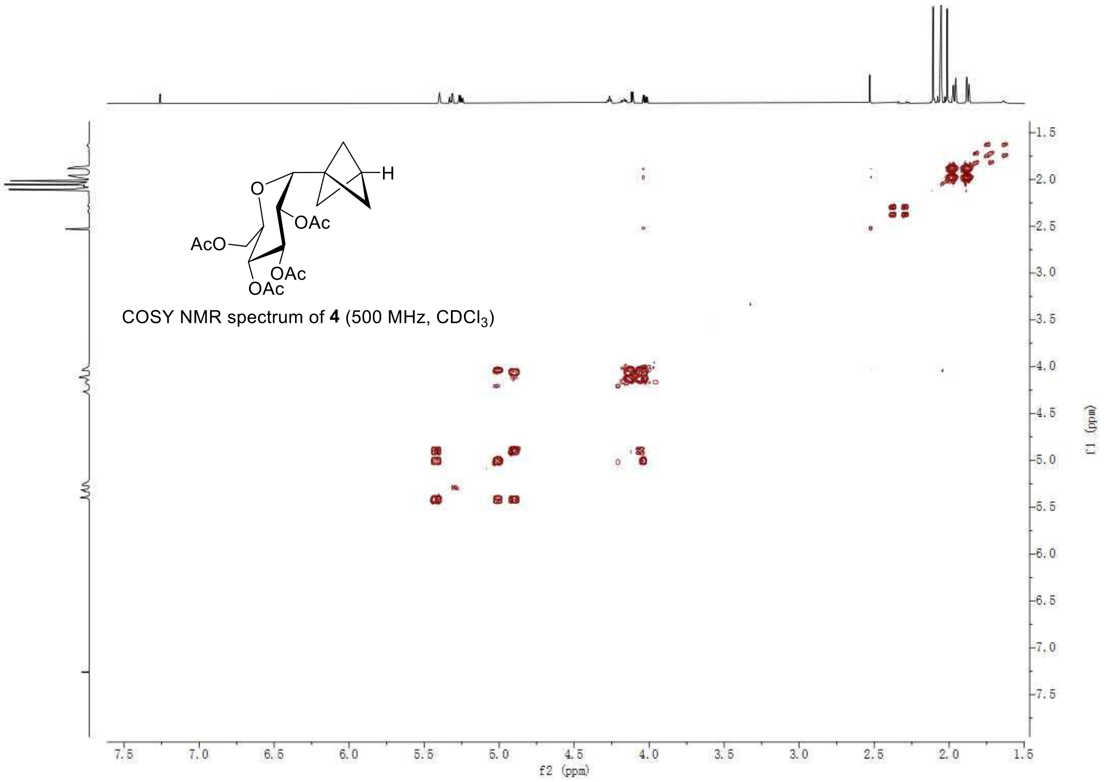

COSY NMR spectrum of **4** (500 MHz, $CDCl_3$)

NOESY NMR spectrum of **4** (500 MHz, $CDCl_3$)

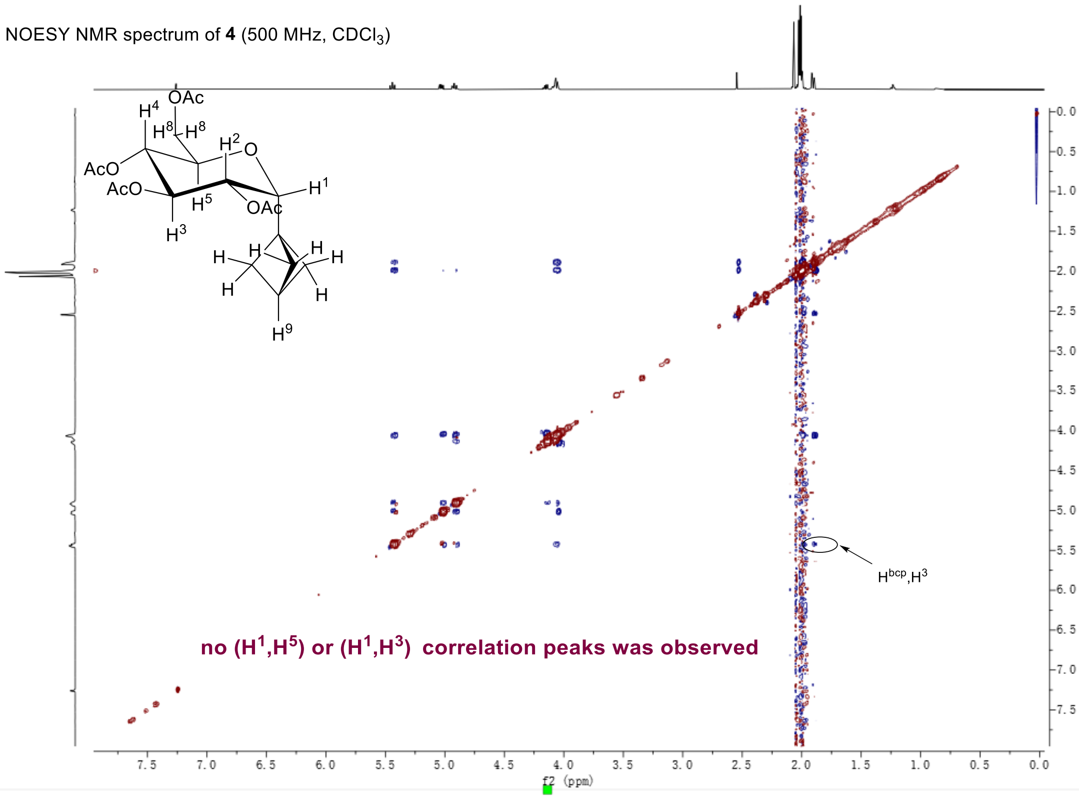

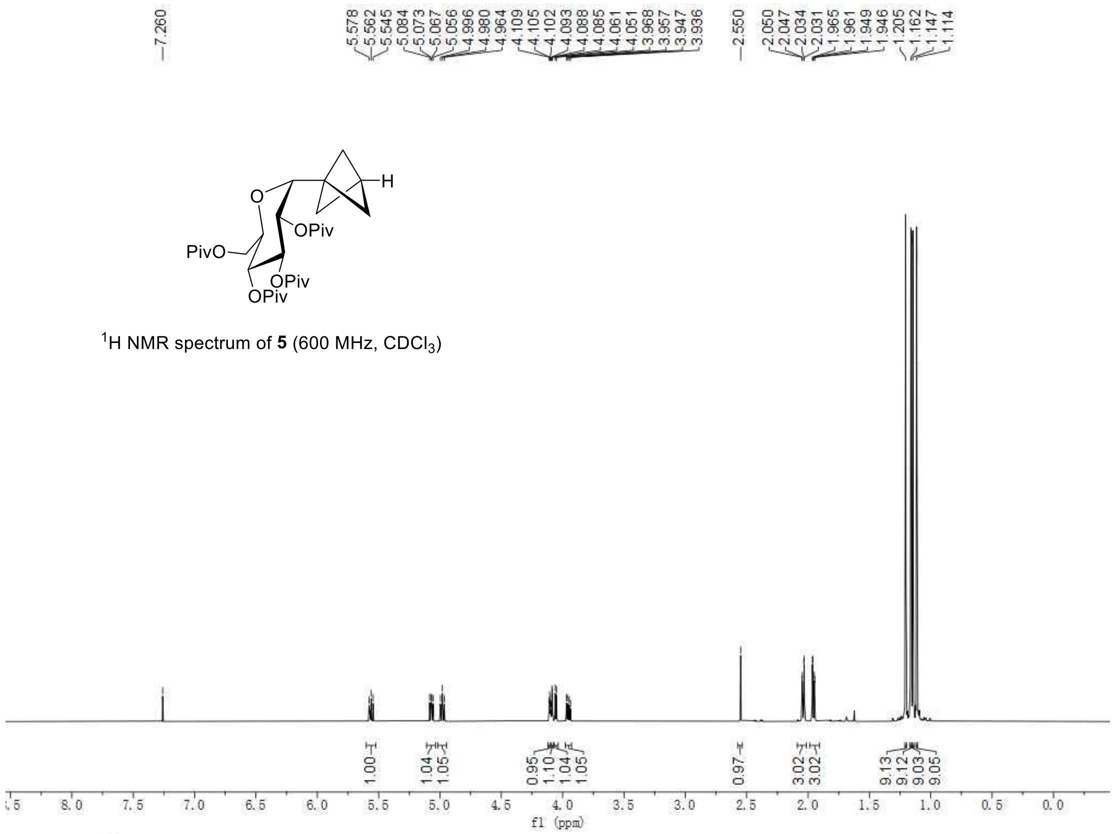

^{1}H NMR spectrum of **5** (600 MHz, $CDCl_3$)

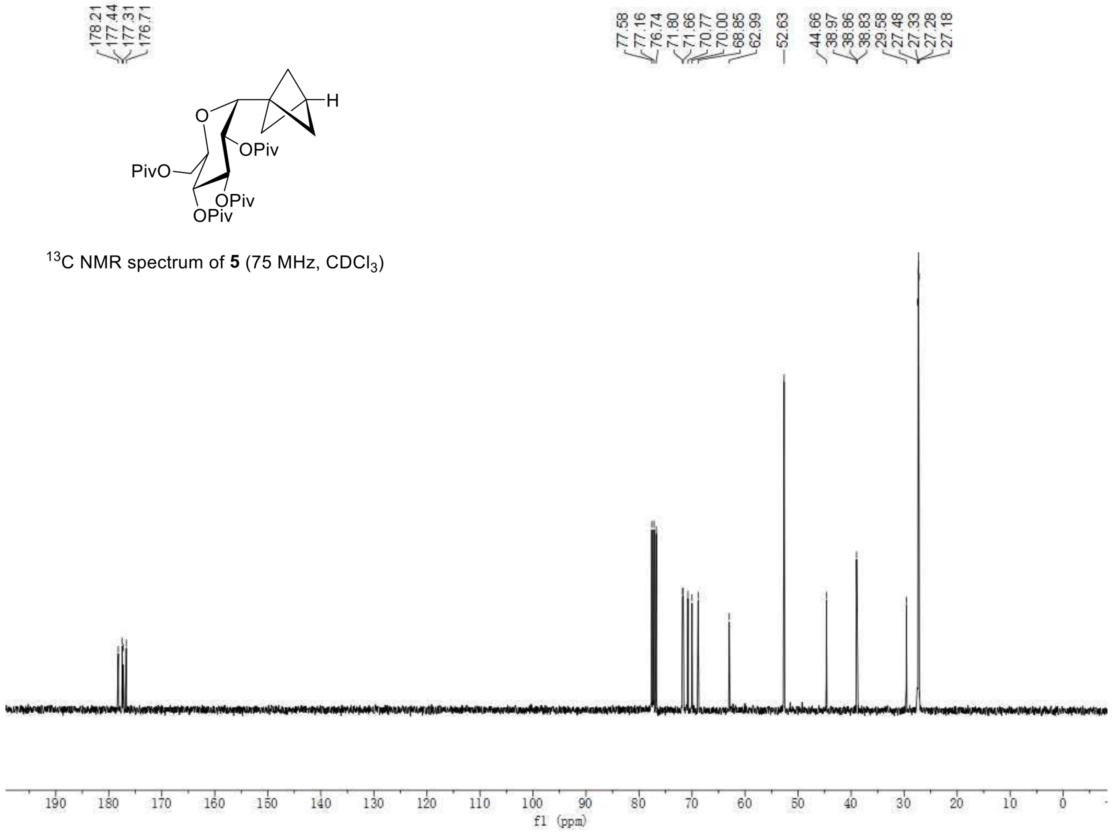

^{13}C NMR spectrum of **5** (75 MHz, $CDCl_3$)

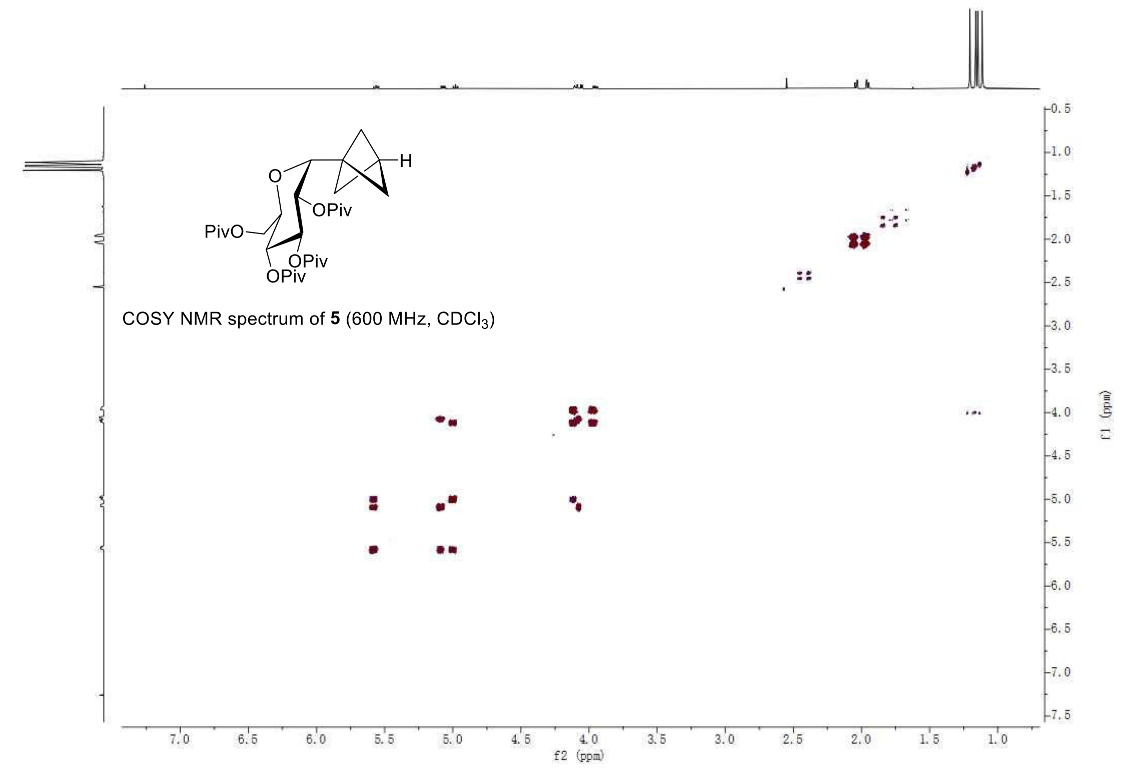

OPiv
PivO
OPiv
OPiv
O
H
COSY NMR spectrum of 5 (600 MHz, CDCl3)
f2 (ppm)
f1 (ppm)

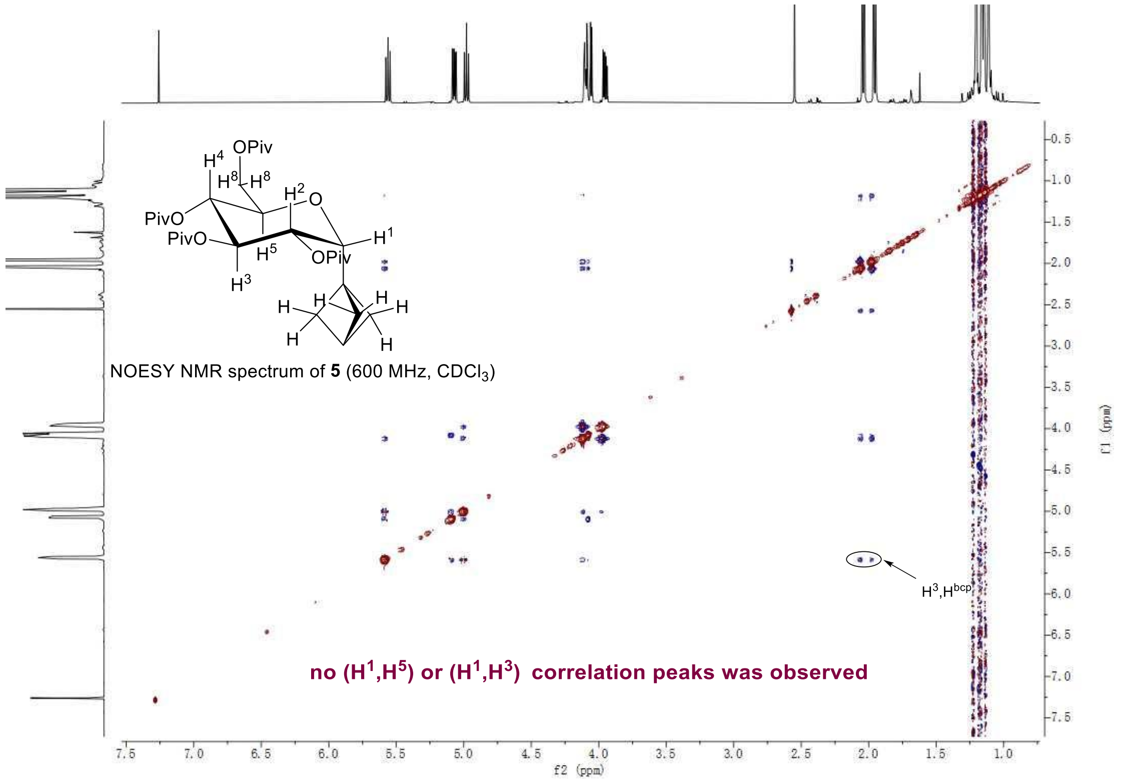

NOESY NMR spectrum of 5 (600 MHz, CDCl3)
H3,Hbcp
no (H1,H5) or (H1,H3) correlation peaks was observed
f2 (ppm)
f1 (ppm)

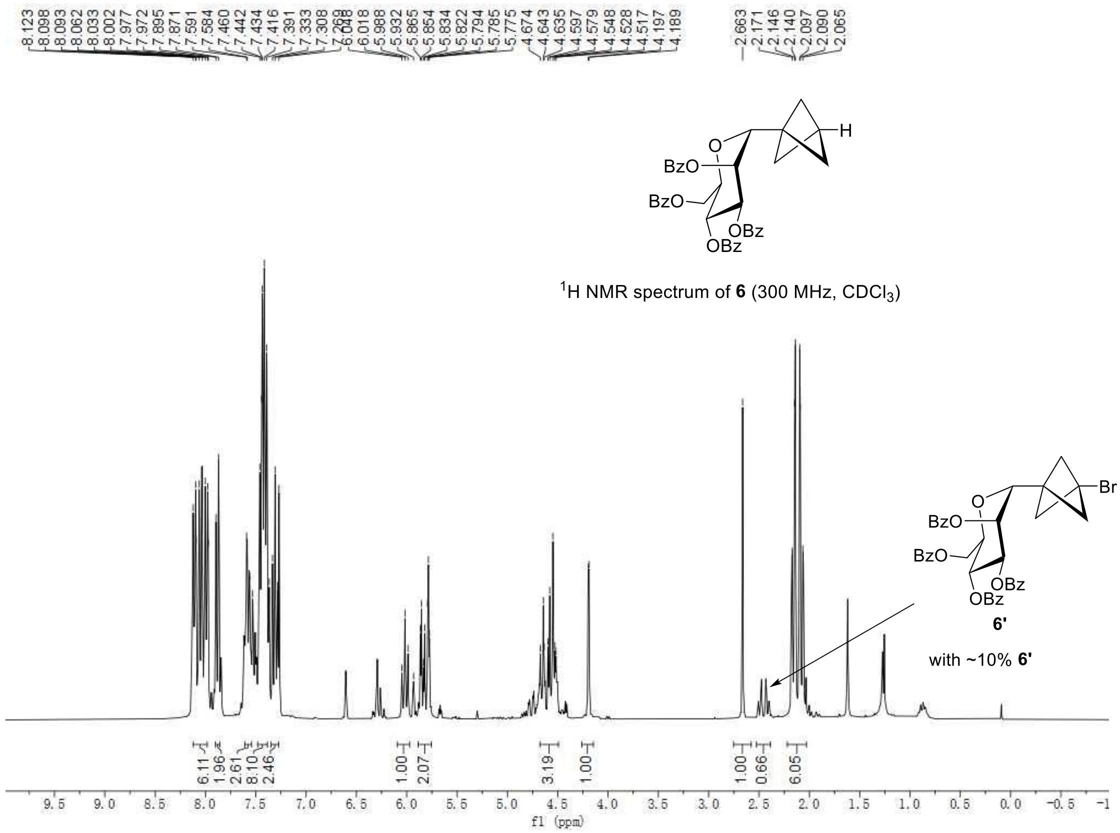

^{1}H NMR spectrum of **6** (300 MHz, $CDCl_3$)

with ~10% **6'**

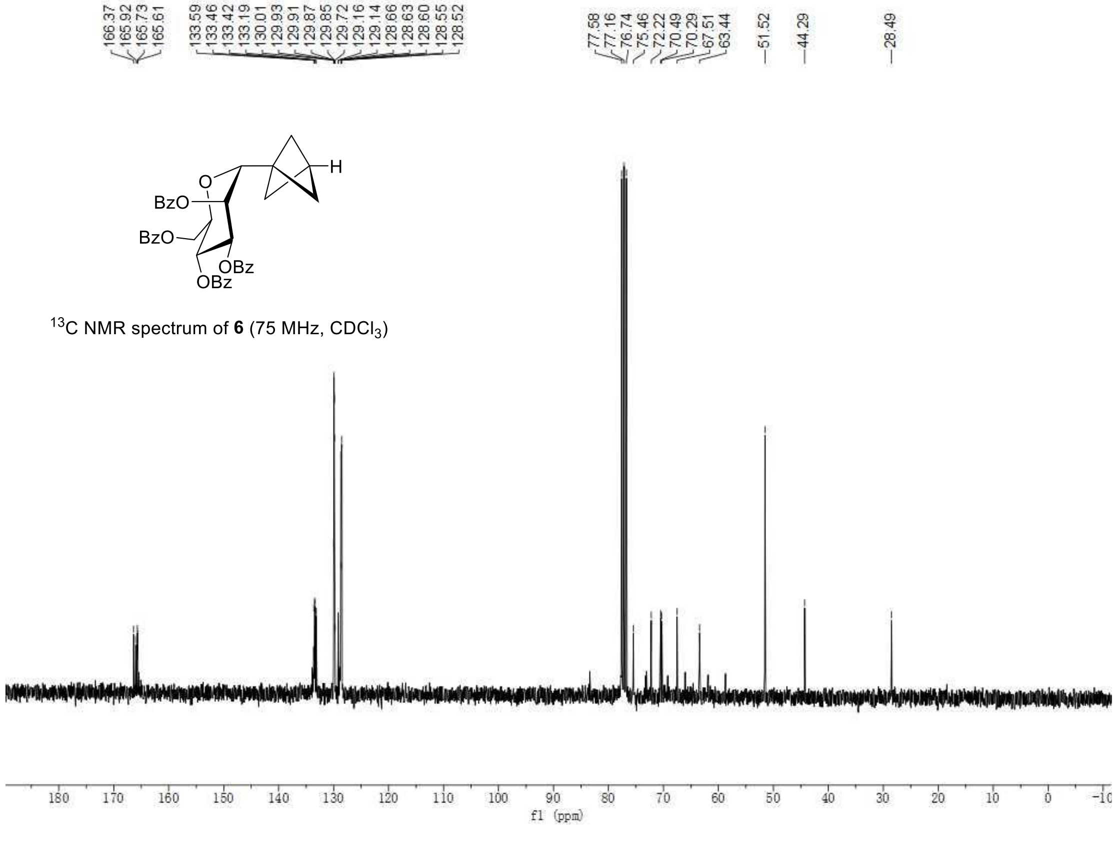

^{13}C NMR spectrum of **6** (75 MHz, $CDCl_3$)

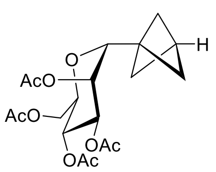

^{1}H NMR spectrum of **7** (300 MHz, $CDCl_3$)

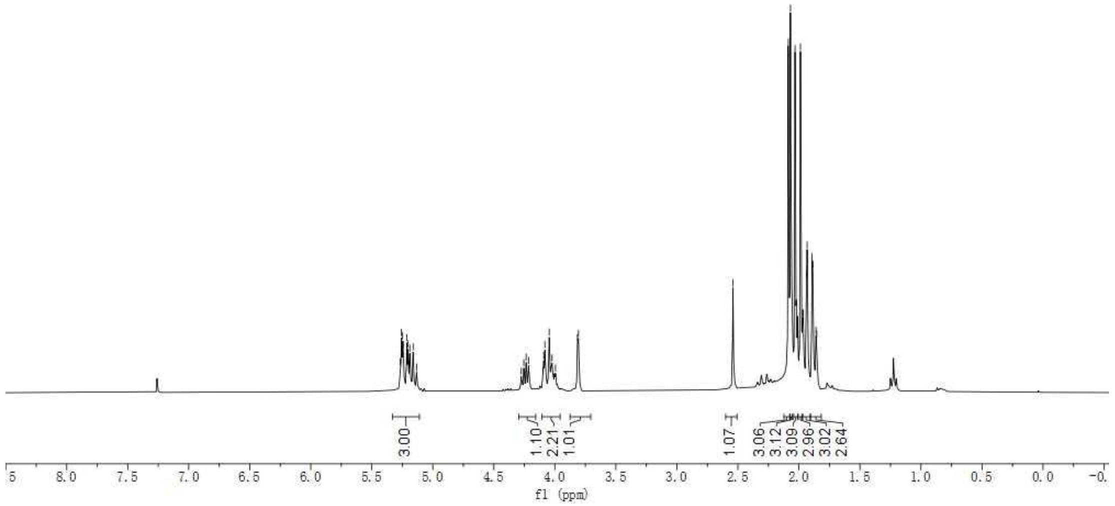

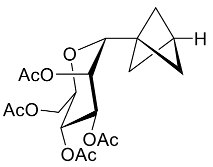

^{13}C NMR spectrum of **7** (75 MHz, $CDCl_3$)

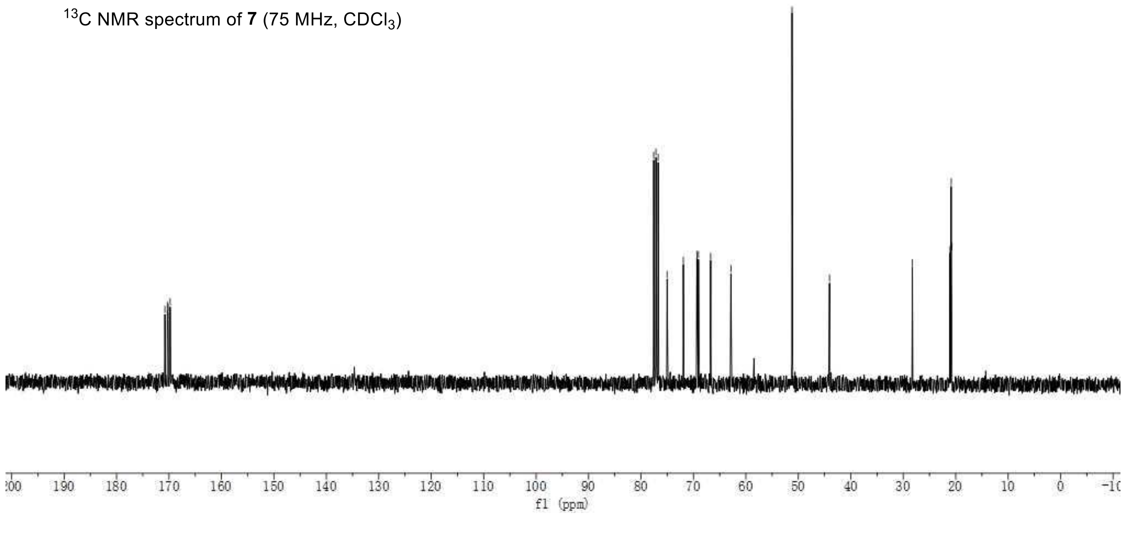

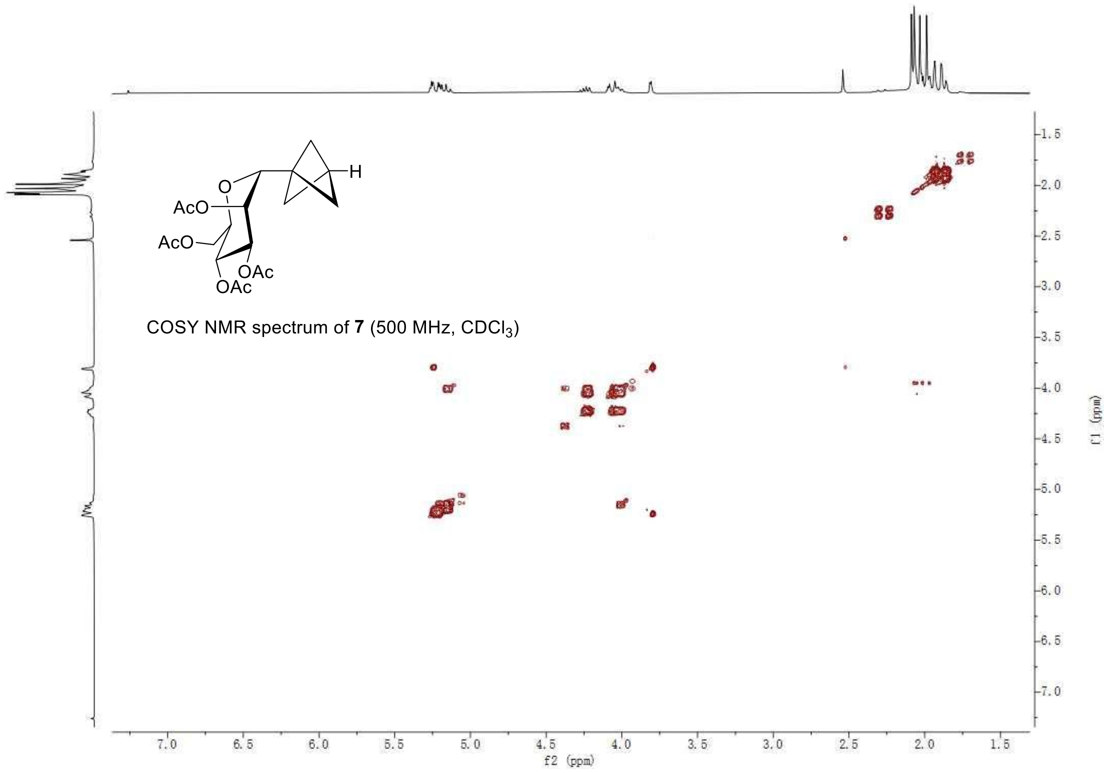

COSY NMR spectrum of **7** (500 MHz, $CDCl_3$)

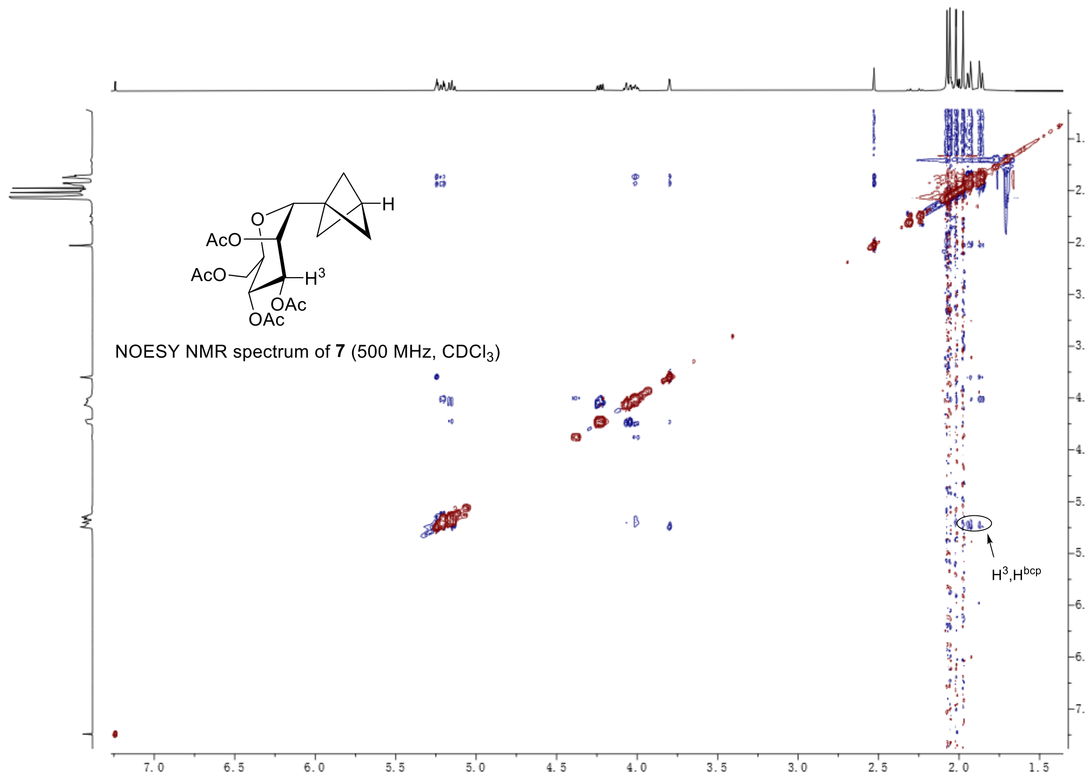

NOESY NMR spectrum of **7** (500 MHz, $CDCl_3$)

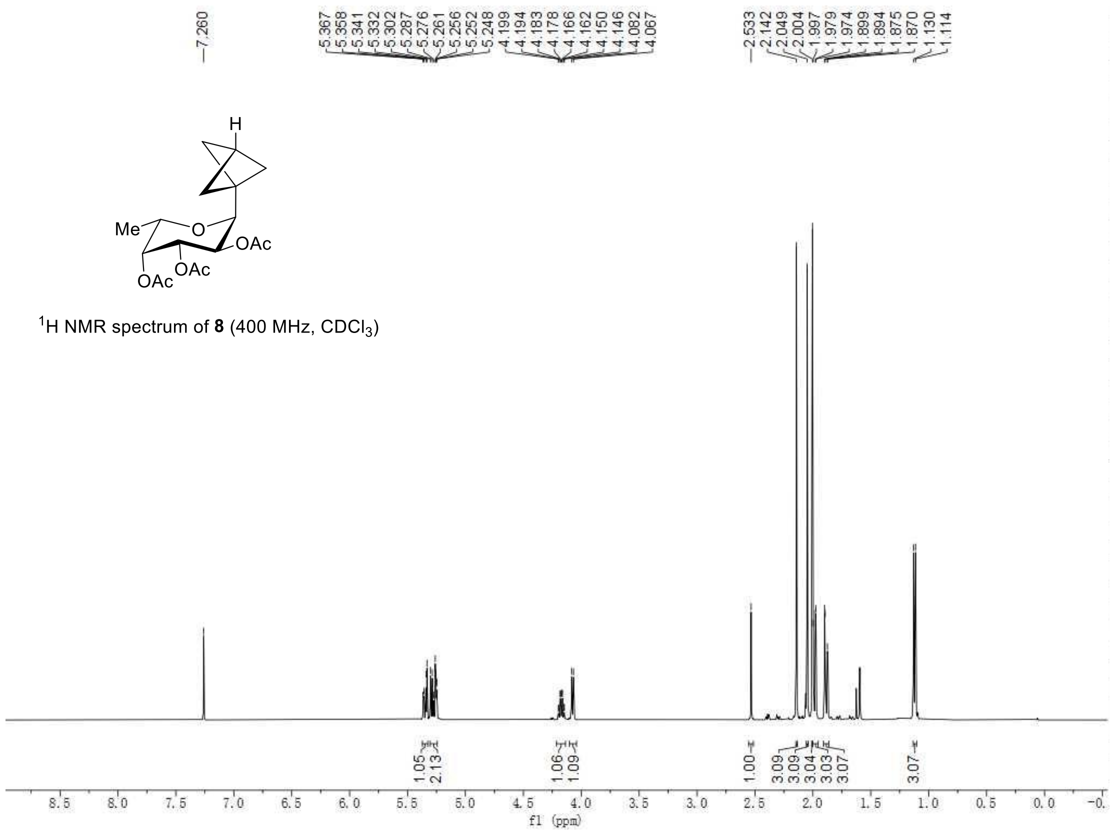

^{1}H NMR spectrum of **8** (400 MHz, $CDCl_3$)

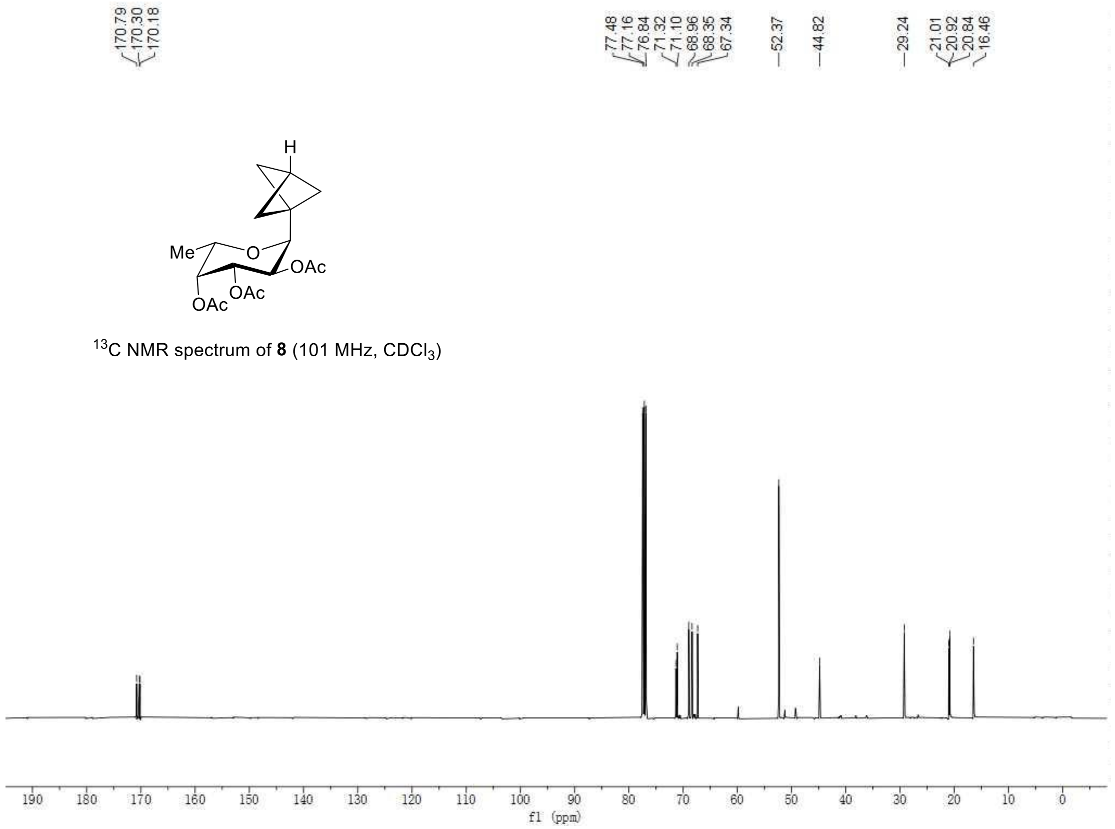

^{13}C NMR spectrum of **8** (101 MHz, $CDCl_3$)

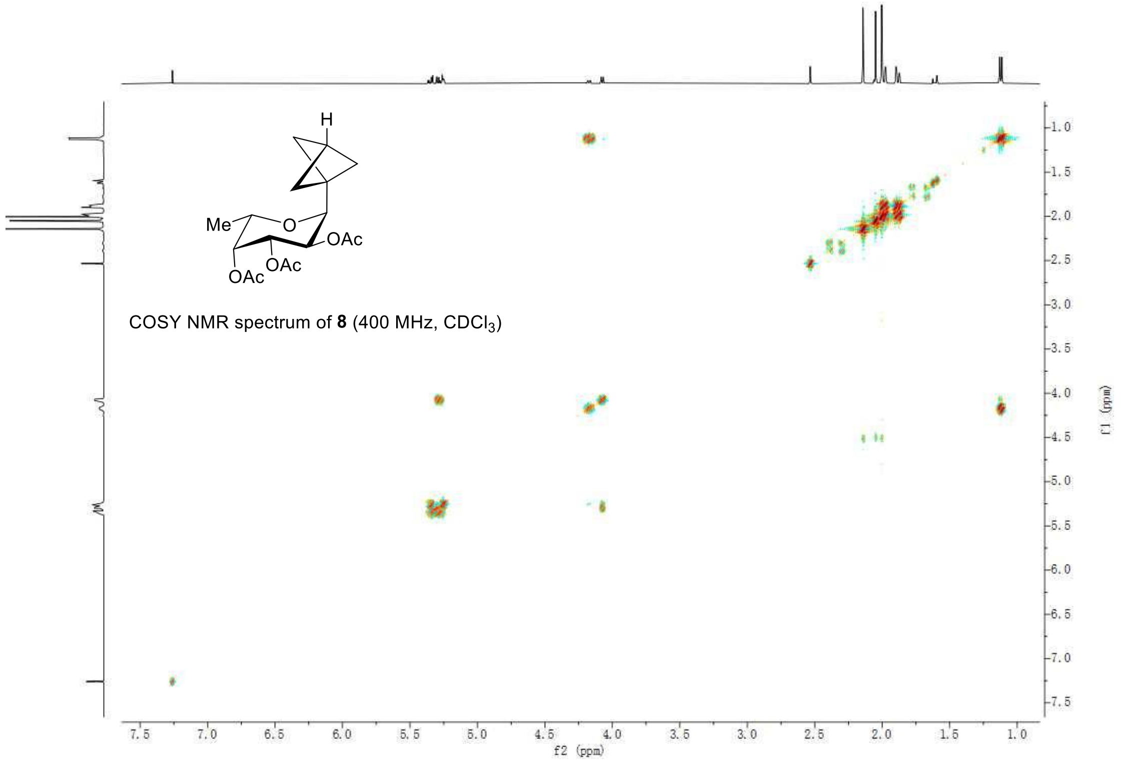
H
Me
O
OAc
OAc
OAc
COSY NMR spectrum of 8 (400 MHz, CDCl3)
f2 (ppm)
f1 (ppm)

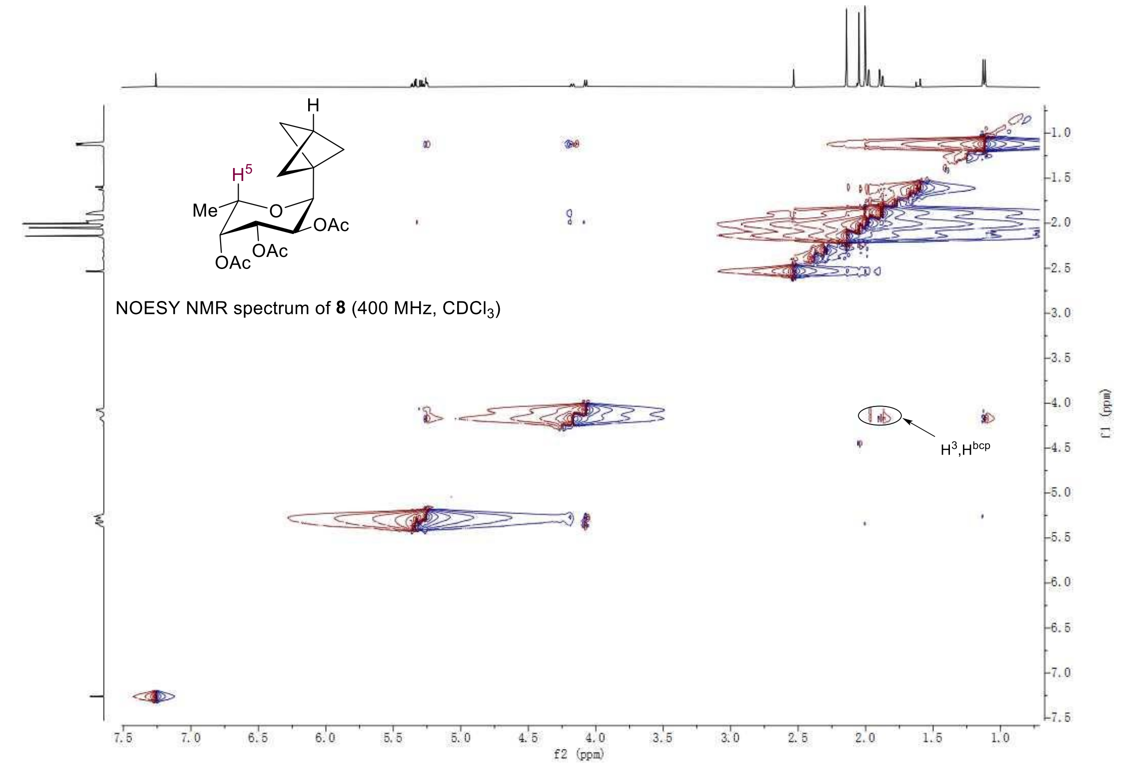
H
H5
Me
O
OAc
OAc
OAc
NOESY NMR spectrum of 8 (400 MHz, CDCl3)
H3,Hbcp
f2 (ppm)
f1 (ppm)

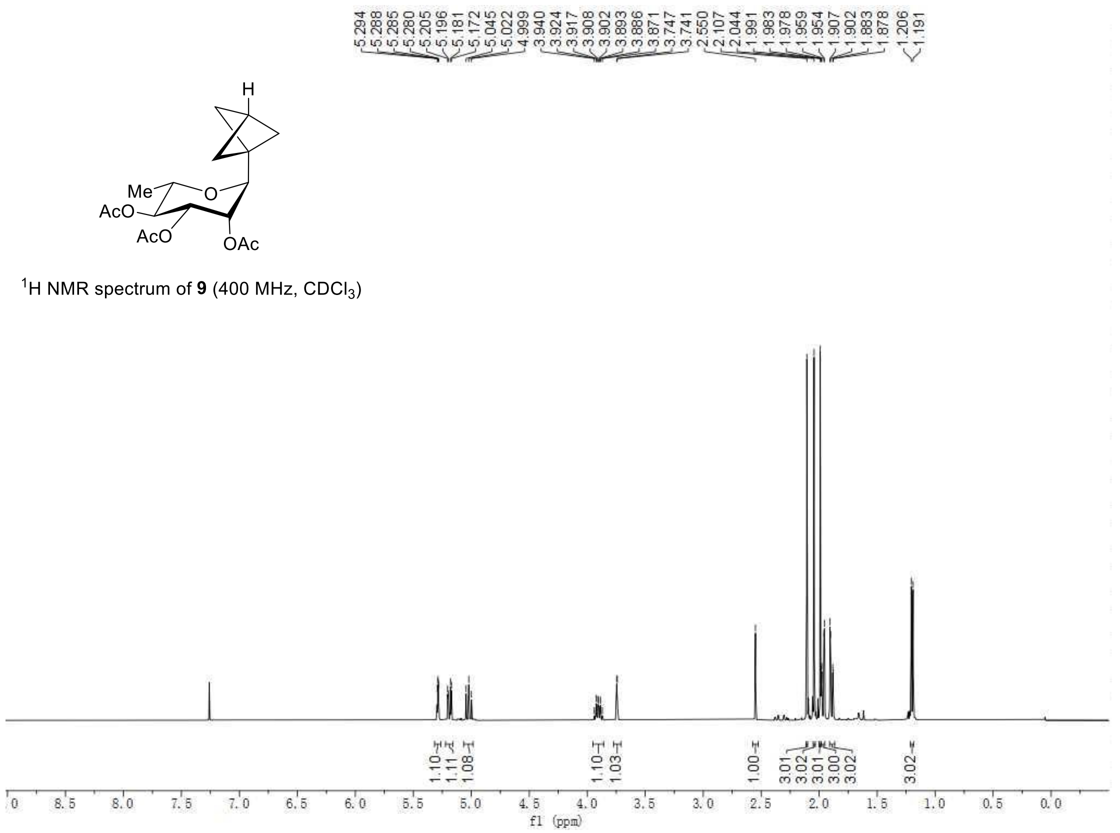

^{1}H NMR spectrum of **9** (400 MHz, $CDCl_3$)

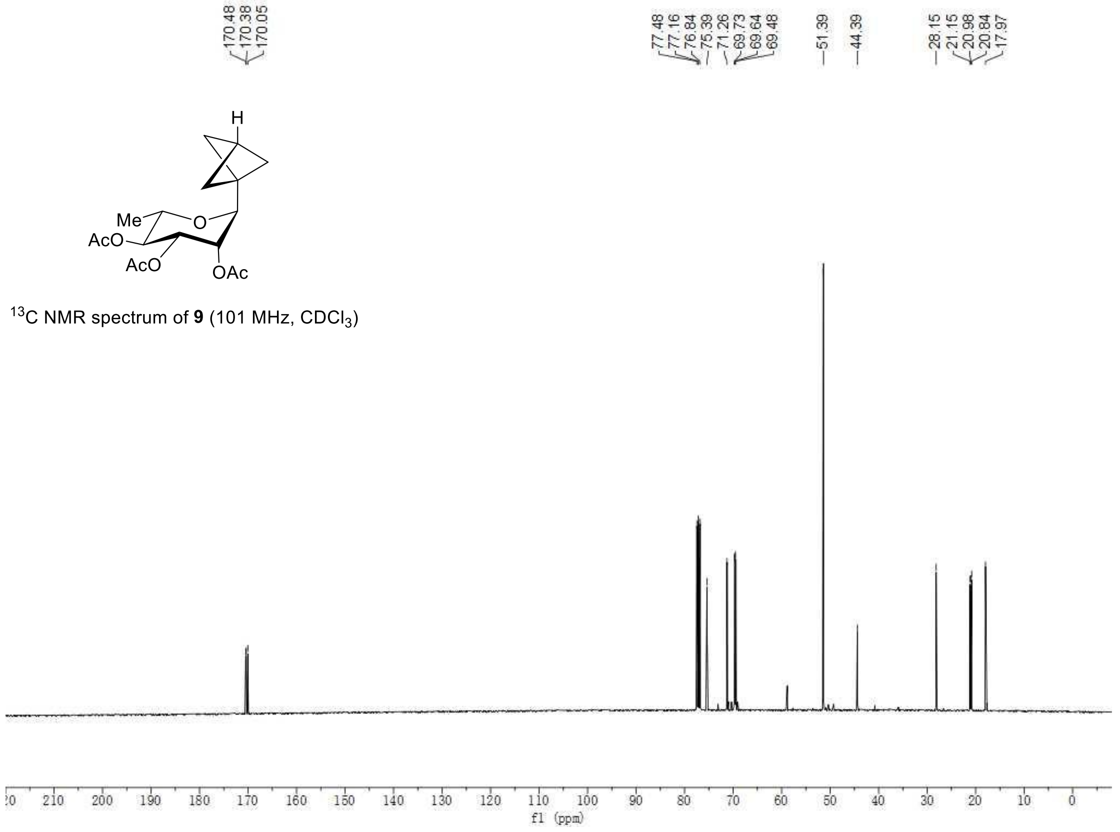

^{13}C NMR spectrum of **9** (101 MHz, $CDCl_3$)

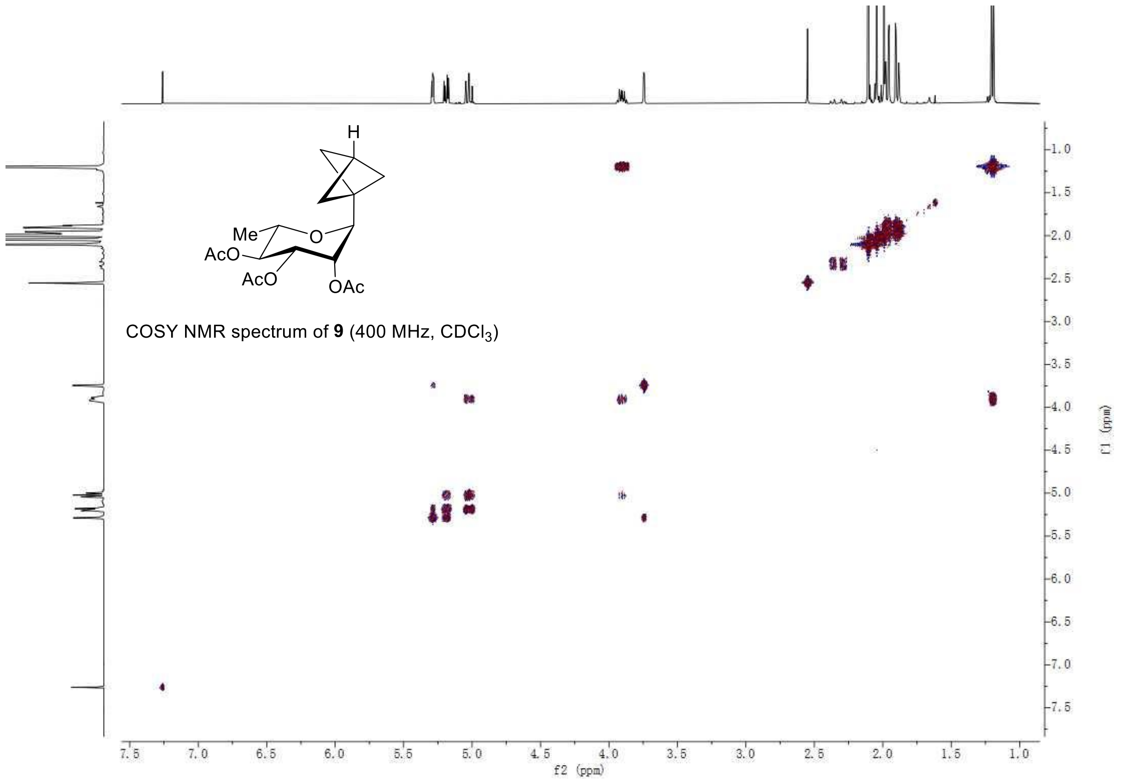
H
Me
O
AcO
AcO
OAc
COSY NMR spectrum of 9 (400 MHz, CDCl$_3$)
f2 (ppm)
f1 (ppm)

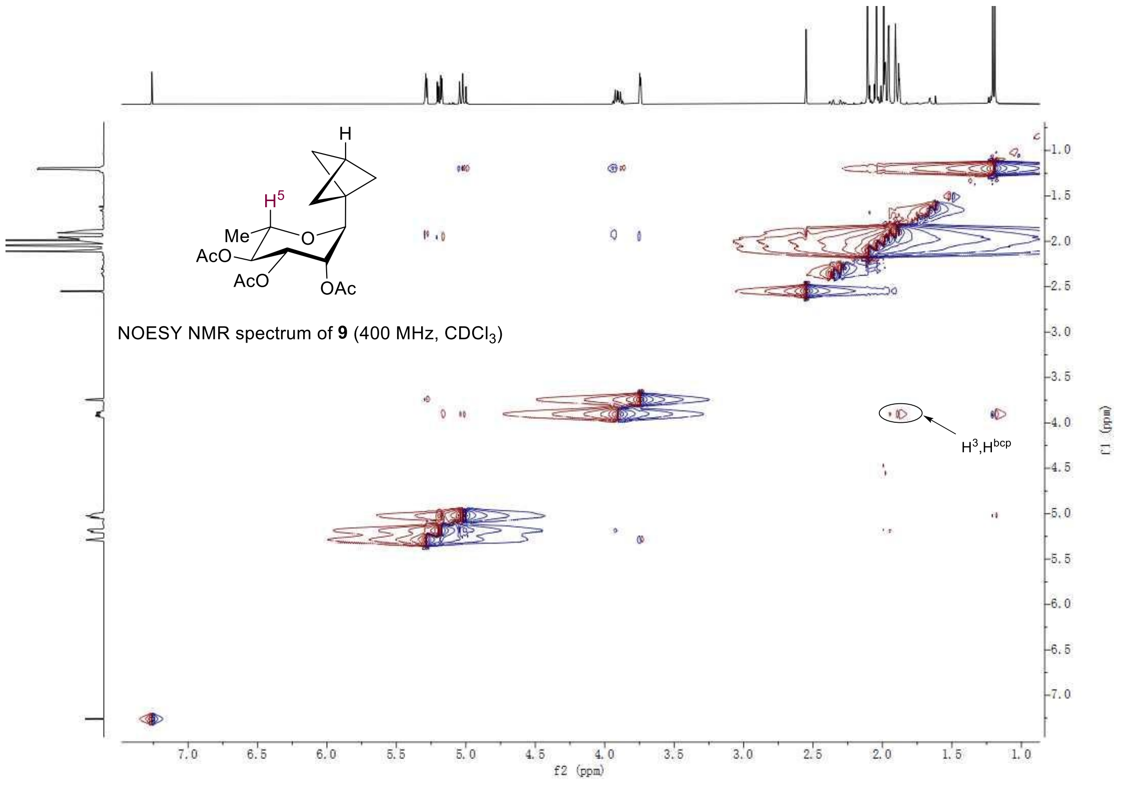
H
H^5
Me
O
AcO
AcO
OAc
NOESY NMR spectrum of 9 (400 MHz, CDCl$_3$)
H^3,H^{bcp}
f2 (ppm)
f1 (ppm)

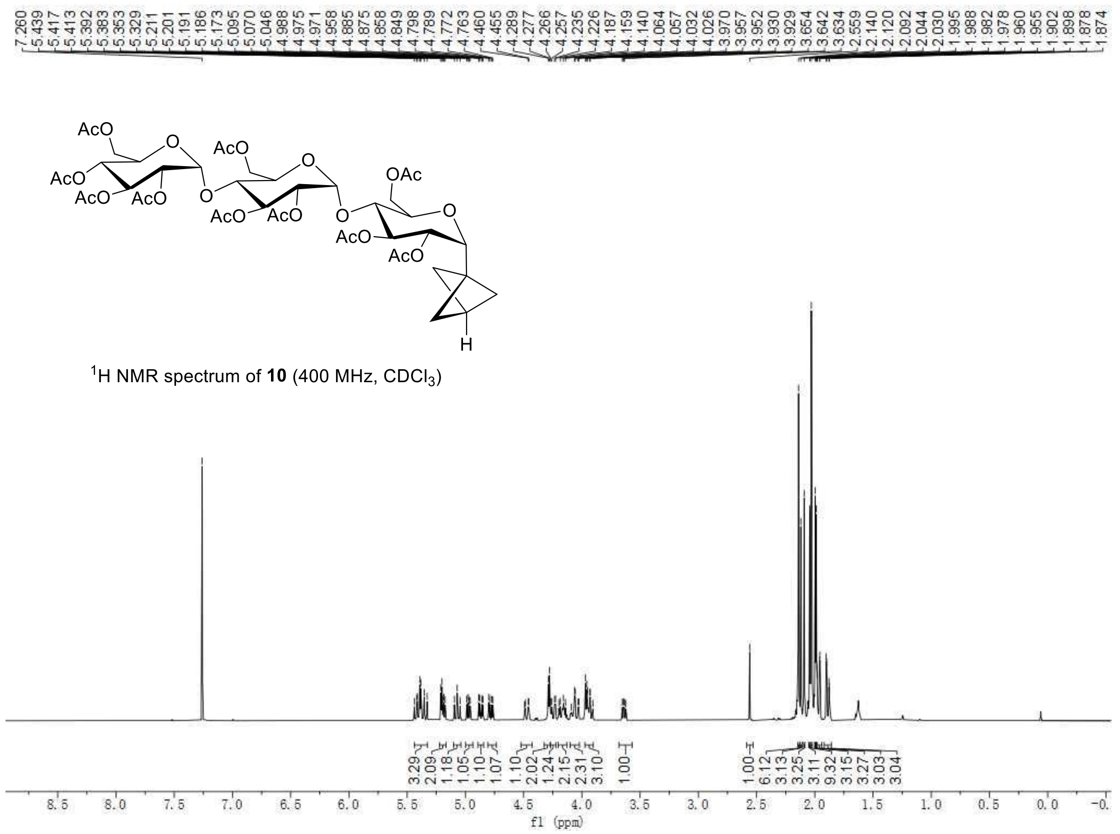

^{1}H NMR spectrum of **10** (400 MHz, $CDCl_3$)

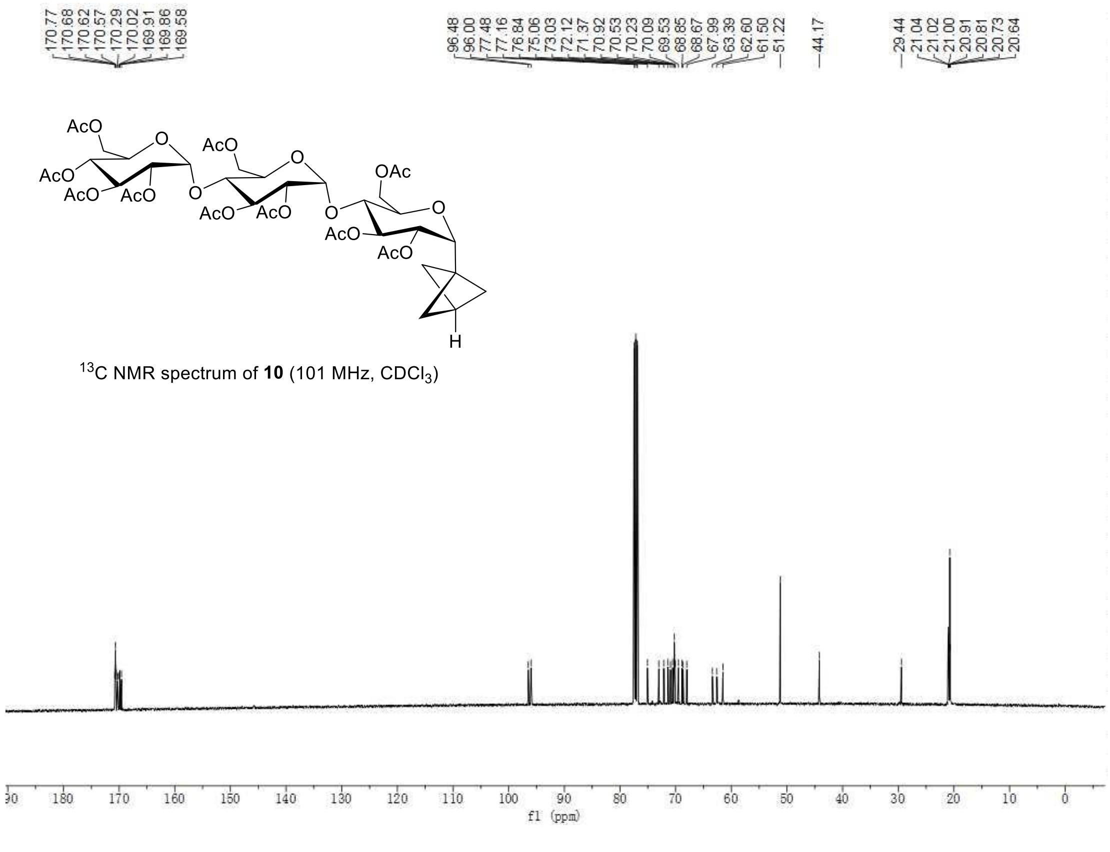

^{13}C NMR spectrum of **10** (101 MHz, $CDCl_3$)

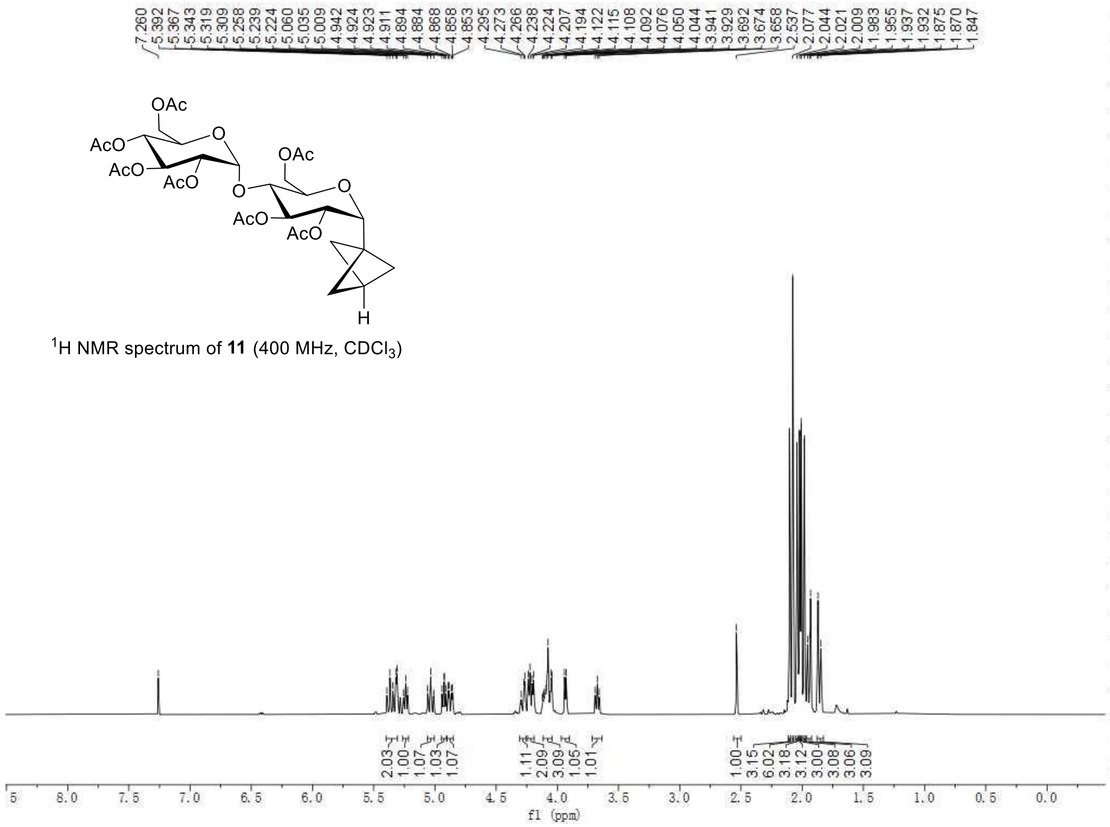

^{1}H NMR spectrum of **11** (400 MHz, $CDCl_3$)

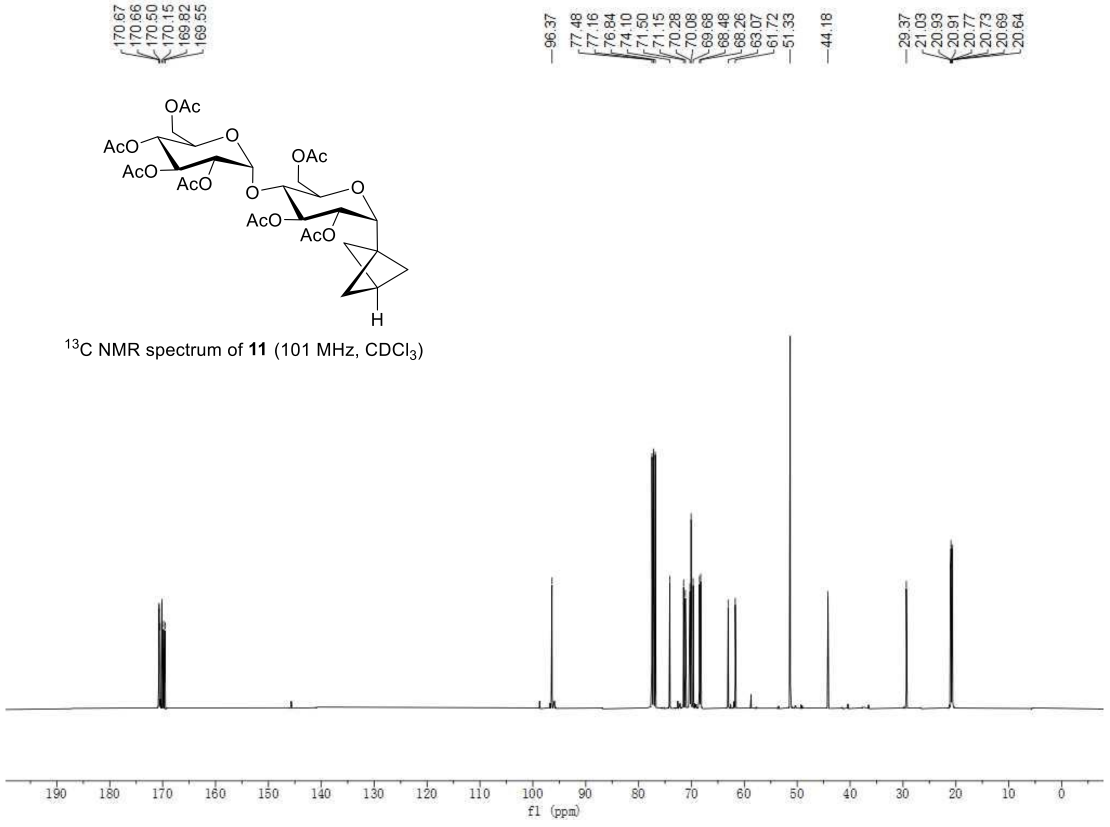

^{13}C NMR spectrum of **11** (101 MHz, $CDCl_3$)

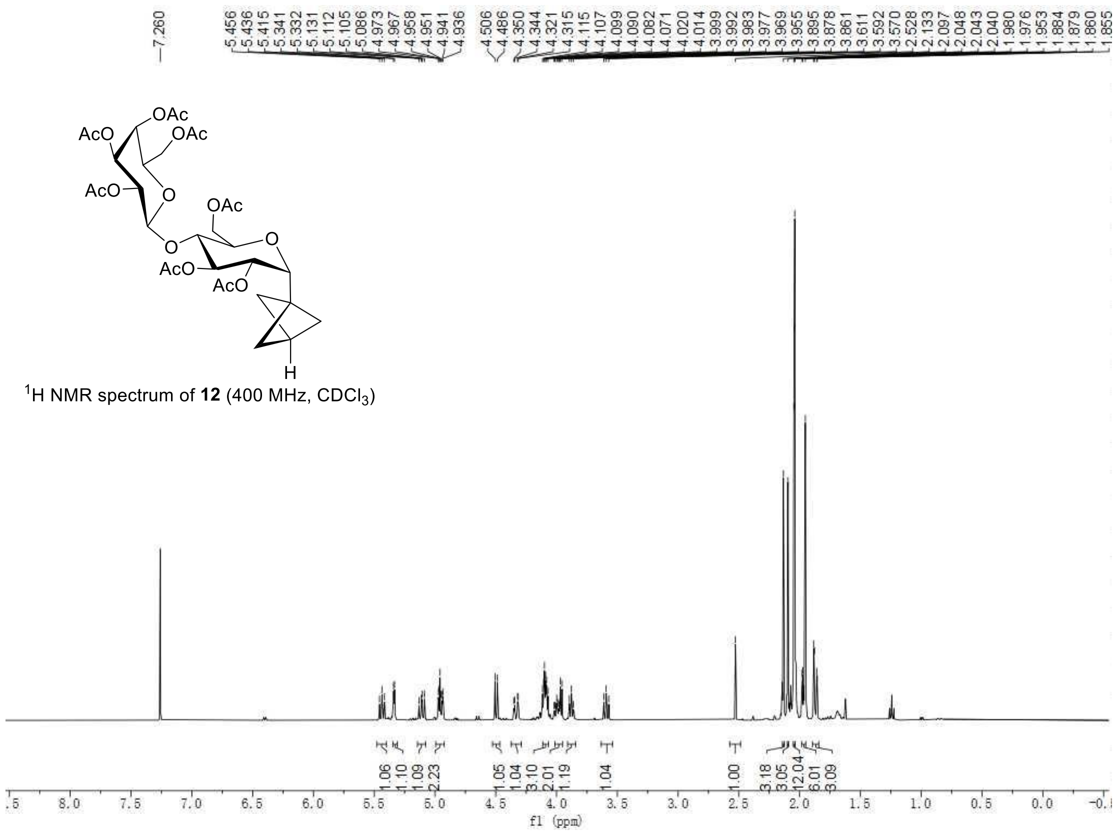

^{1}H NMR spectrum of **12** (400 MHz, $CDCl_3$)

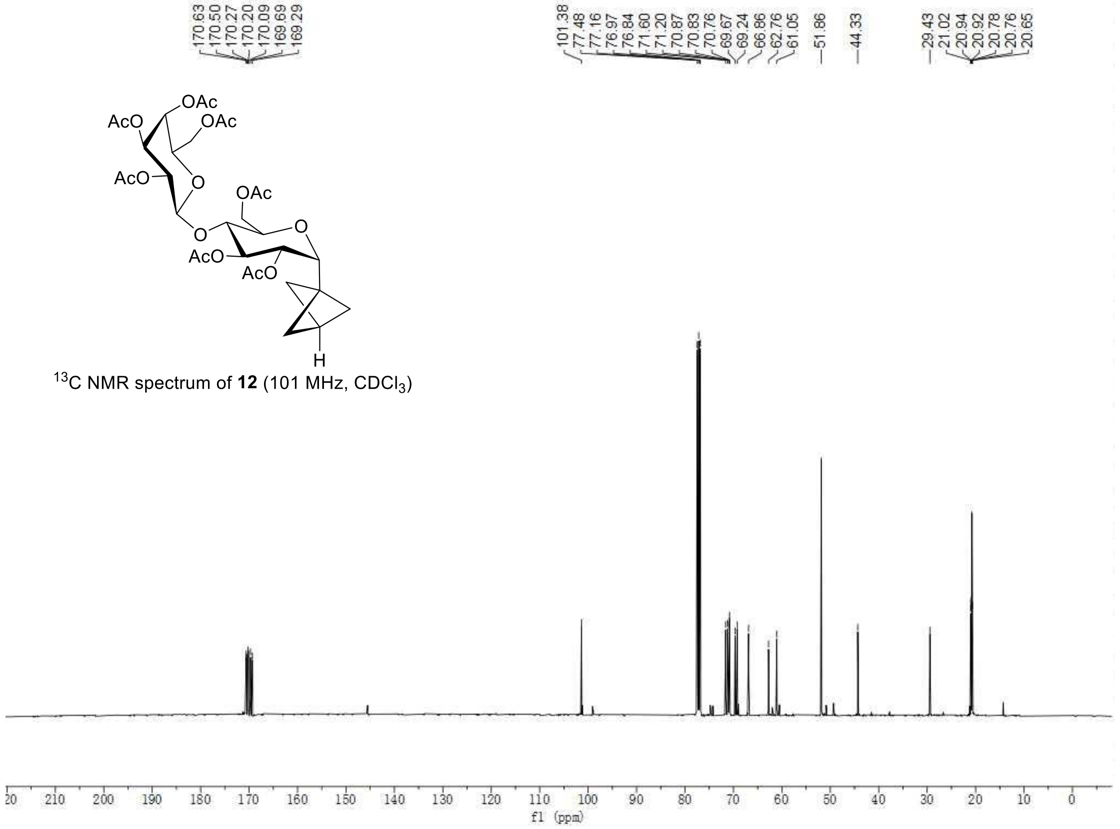

^{13}C NMR spectrum of **12** (101 MHz, $CDCl_3$)

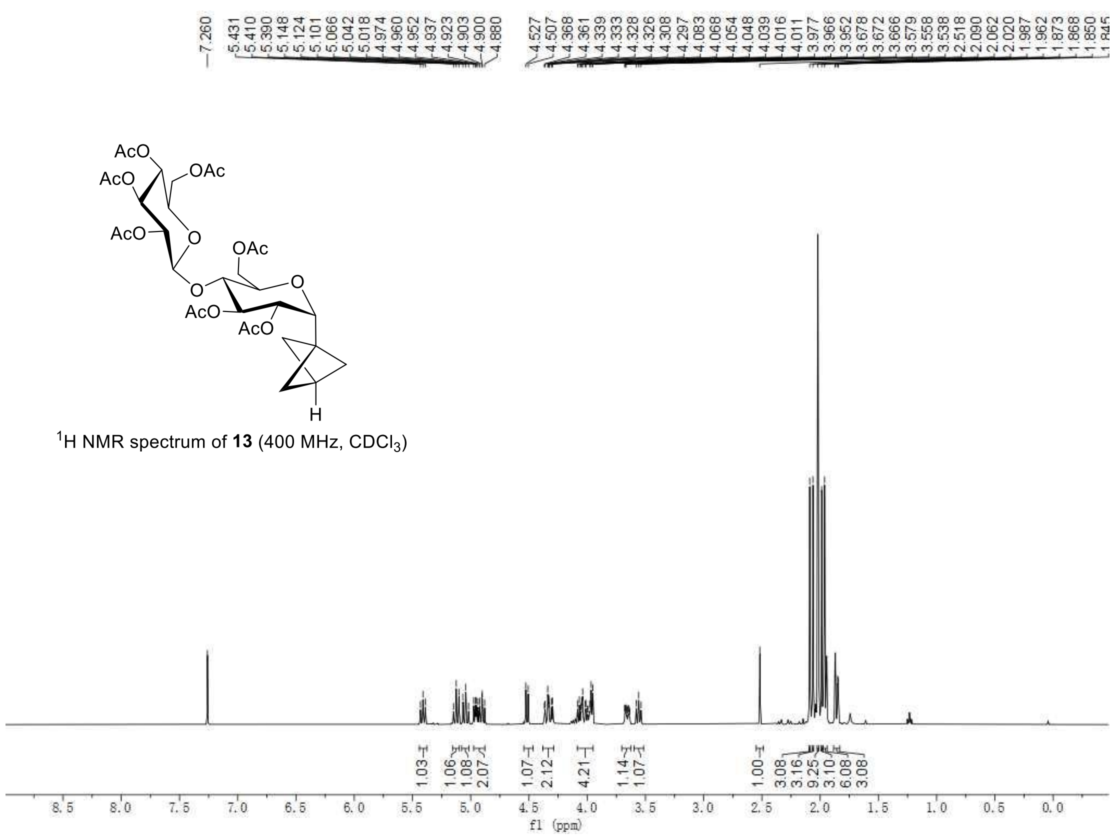

^{1}H NMR spectrum of **13** (400 MHz, $CDCl_3$)

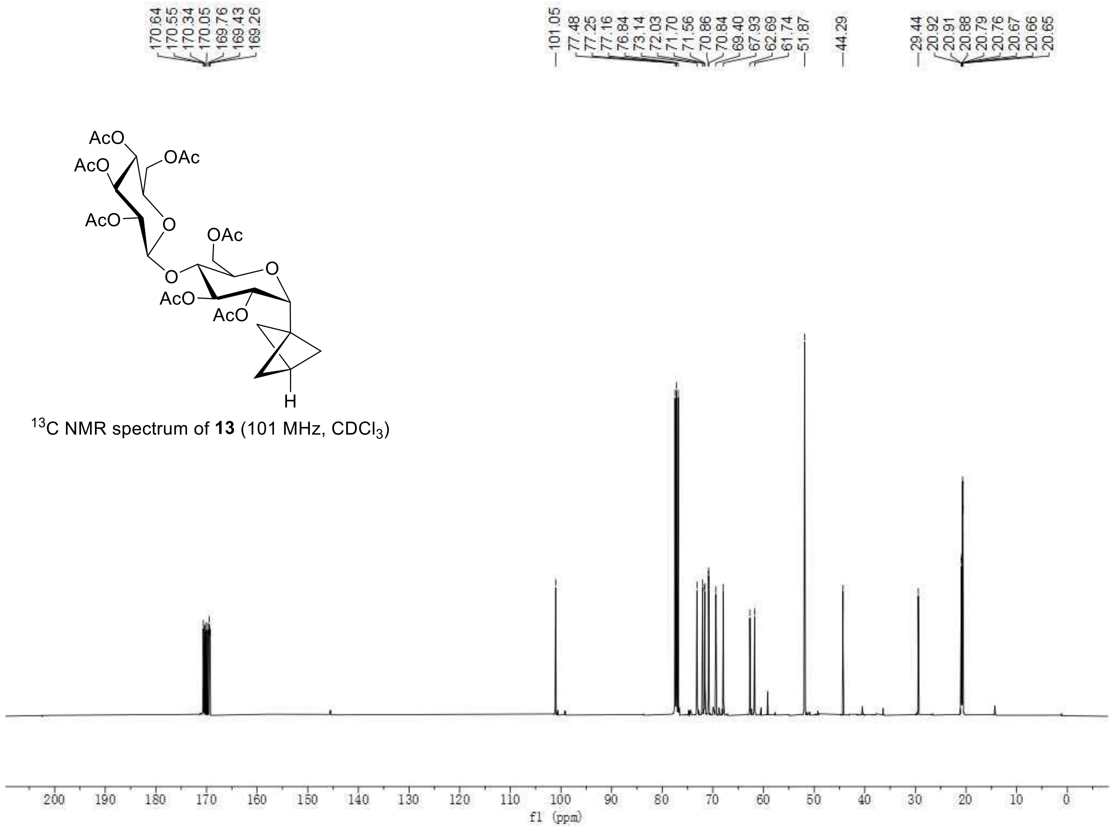

^{13}C NMR spectrum of **13** (101 MHz, $CDCl_3$)

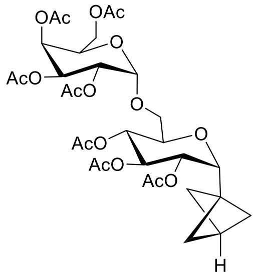

^{1}H NMR spectrum of **14** (400 MHz, $CDCl_3$)

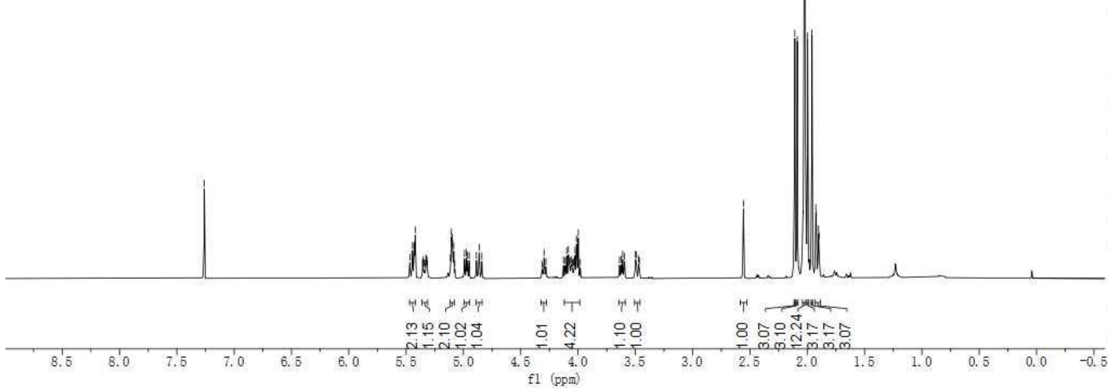

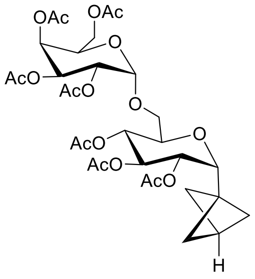

^{13}C NMR spectrum of **14** (101 MHz, $CDCl_3$)

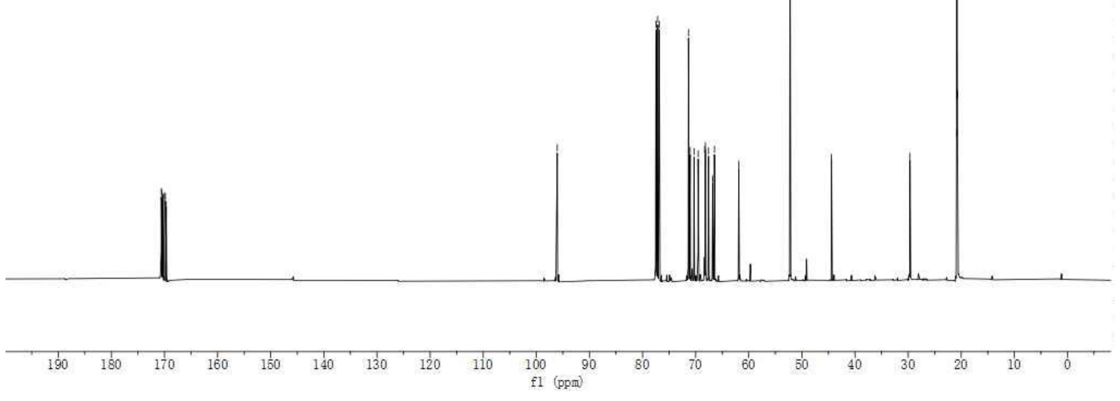

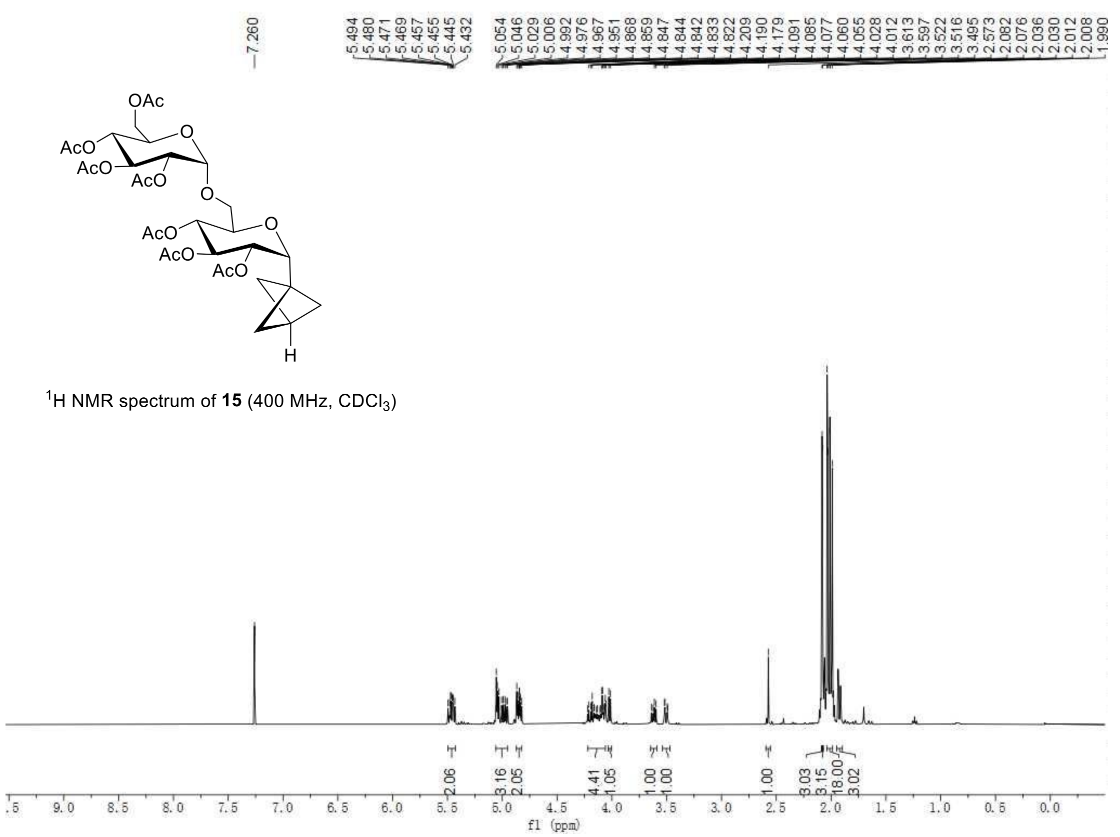

^{1}H NMR spectrum of **15** (400 MHz, $CDCl_3$)

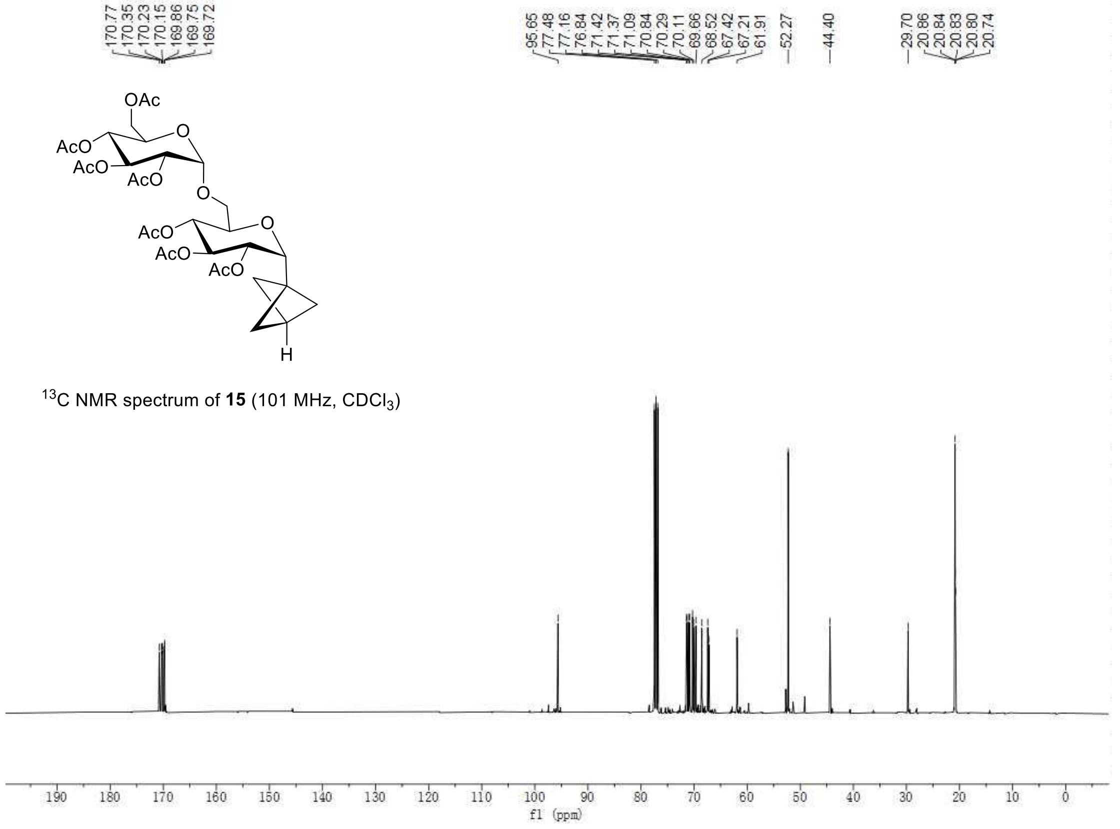

^{13}C NMR spectrum of **15** (101 MHz, $CDCl_3$)

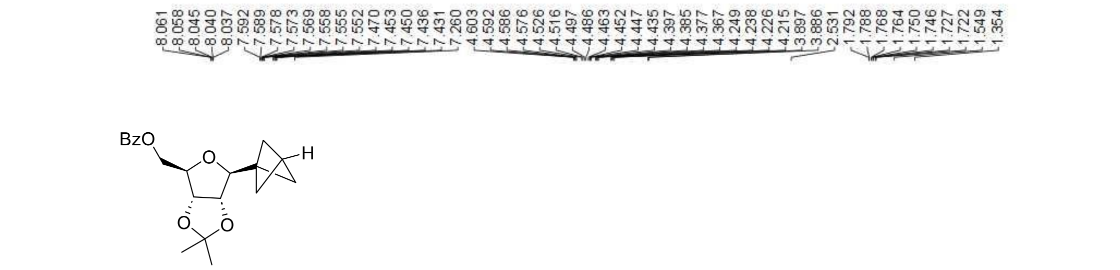

^{1}H NMR spectrum of **16** (400 MHz, $CDCl_3$)

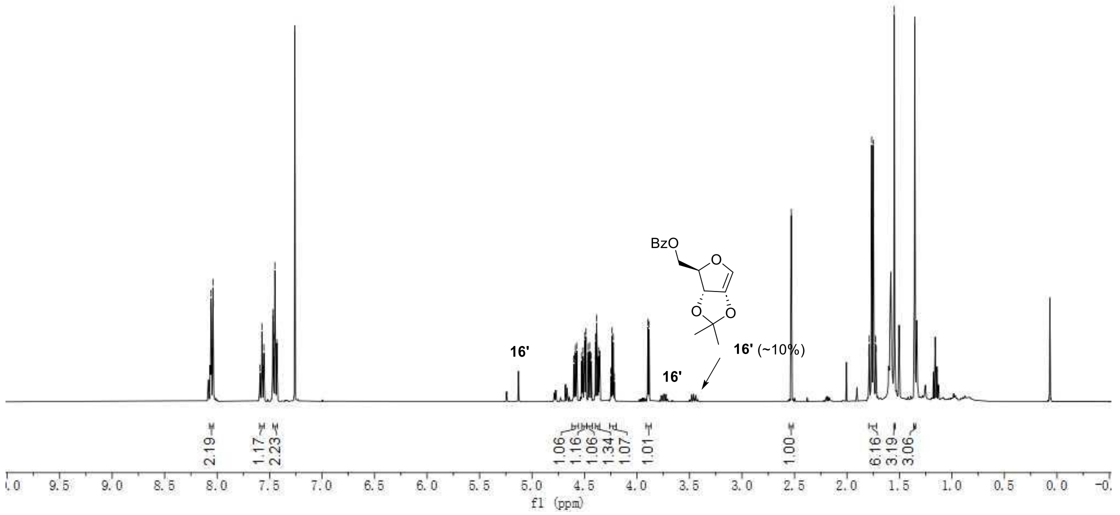

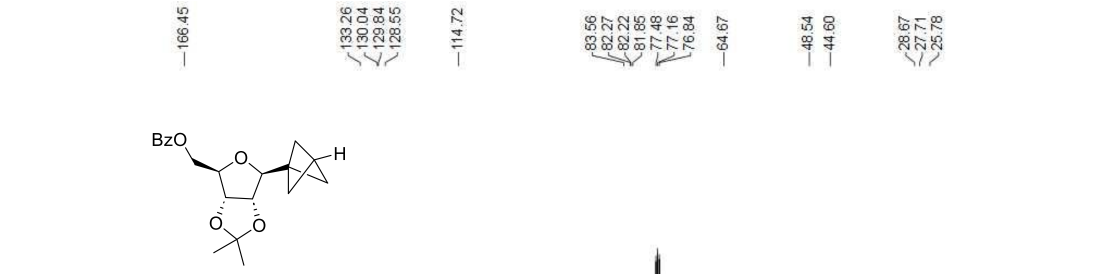

^{13}C NMR spectrum of **16** (101 MHz, $CDCl_3$)

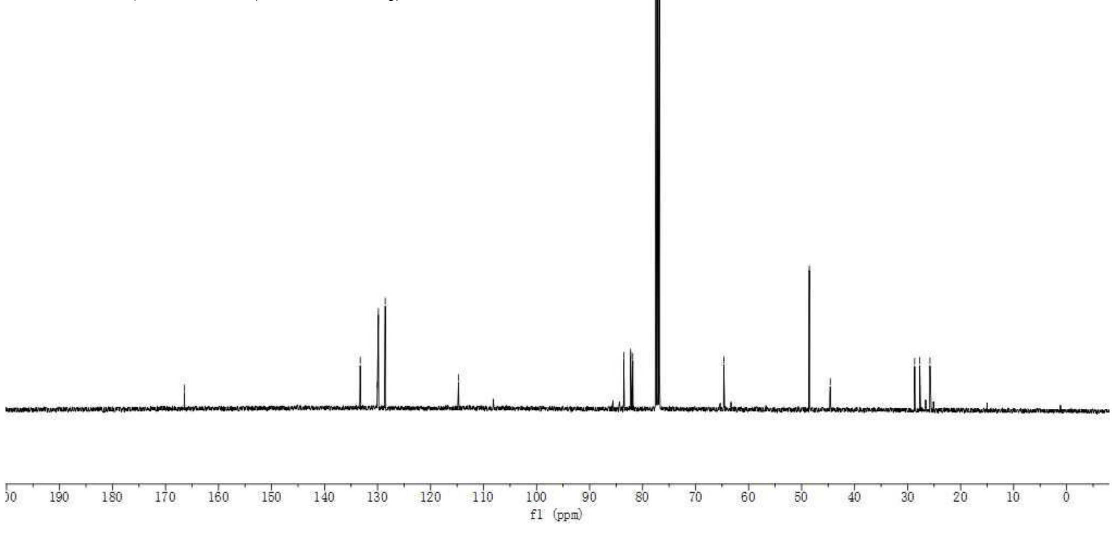

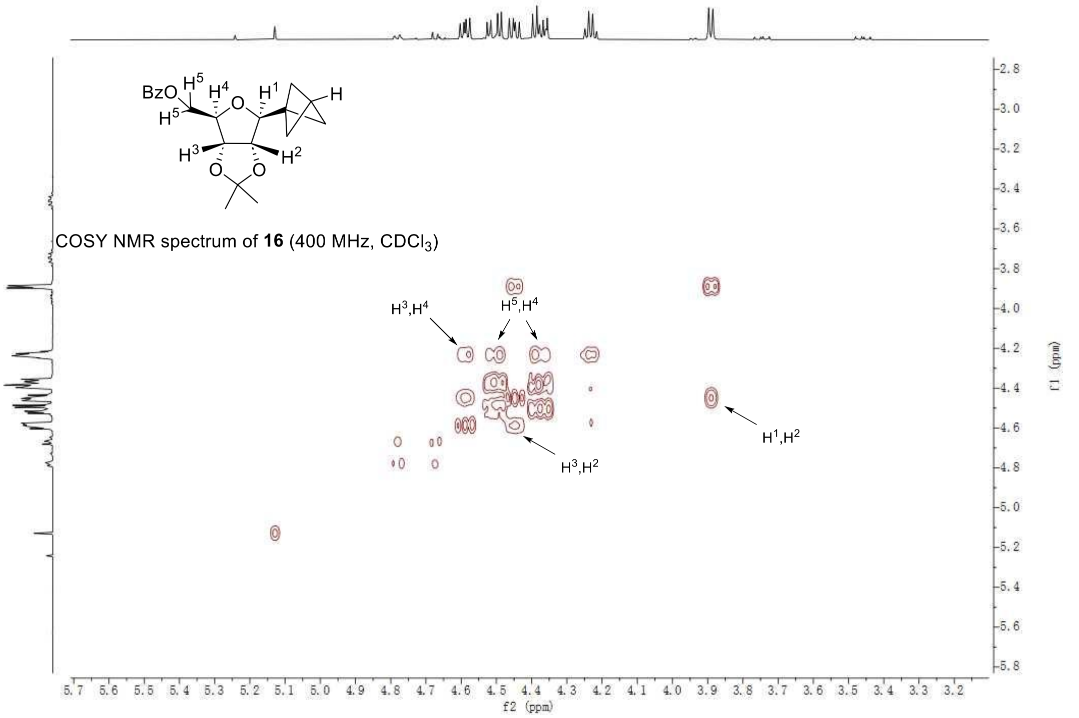

COSY NMR spectrum of **16** (400 MHz, $CDCl_3$)

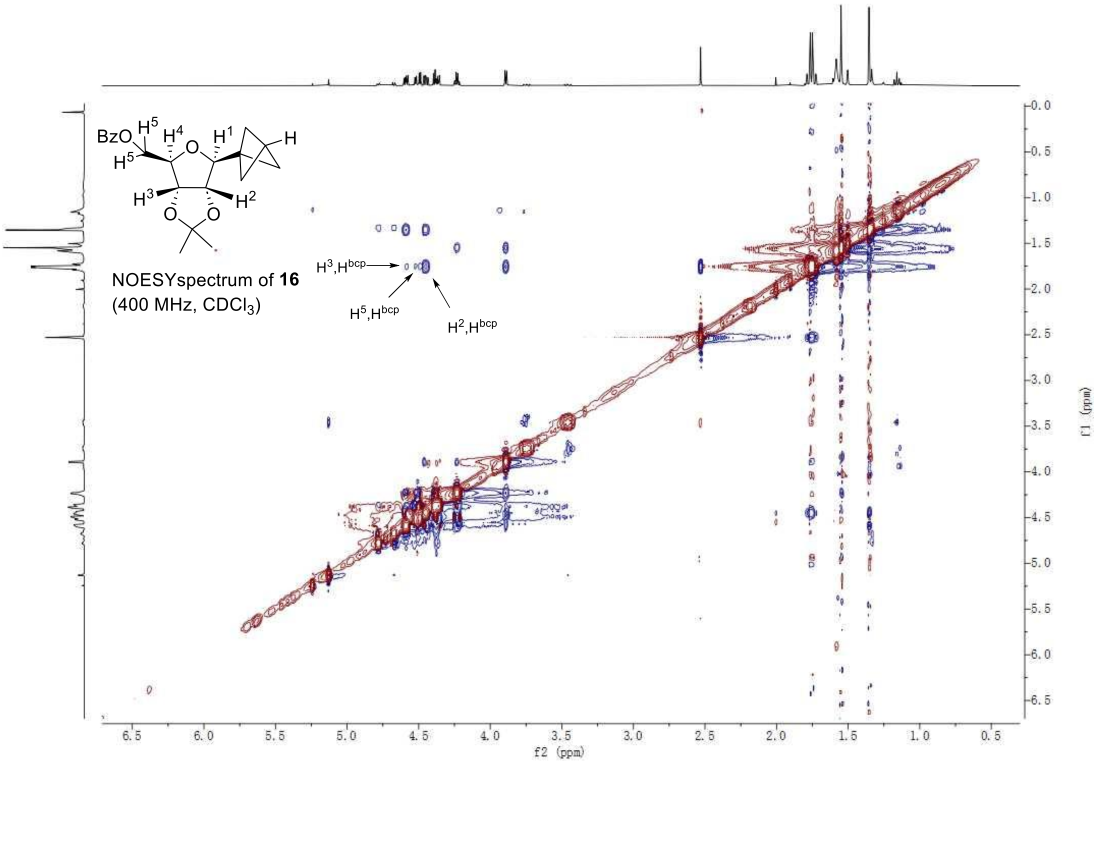

NOESYspectrum of **16** (400 MHz, $CDCl_3$)

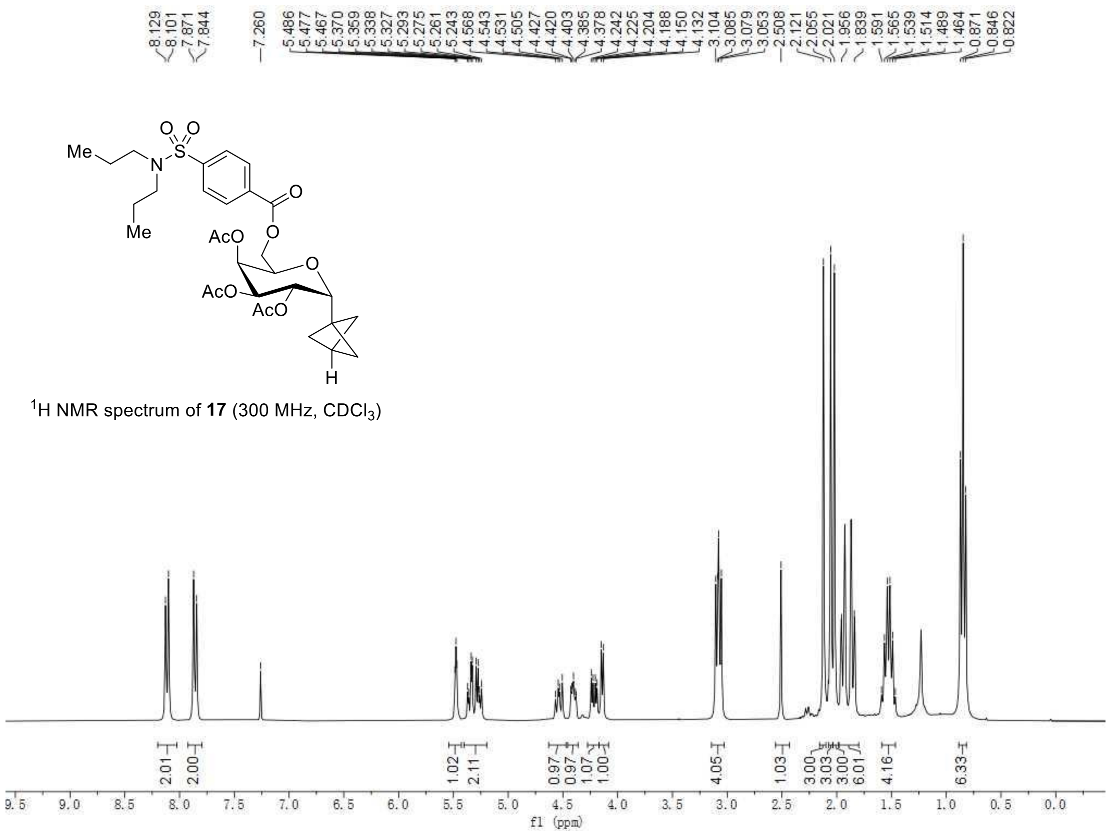

^{1}H NMR spectrum of **17** (300 MHz, $CDCl_3$)

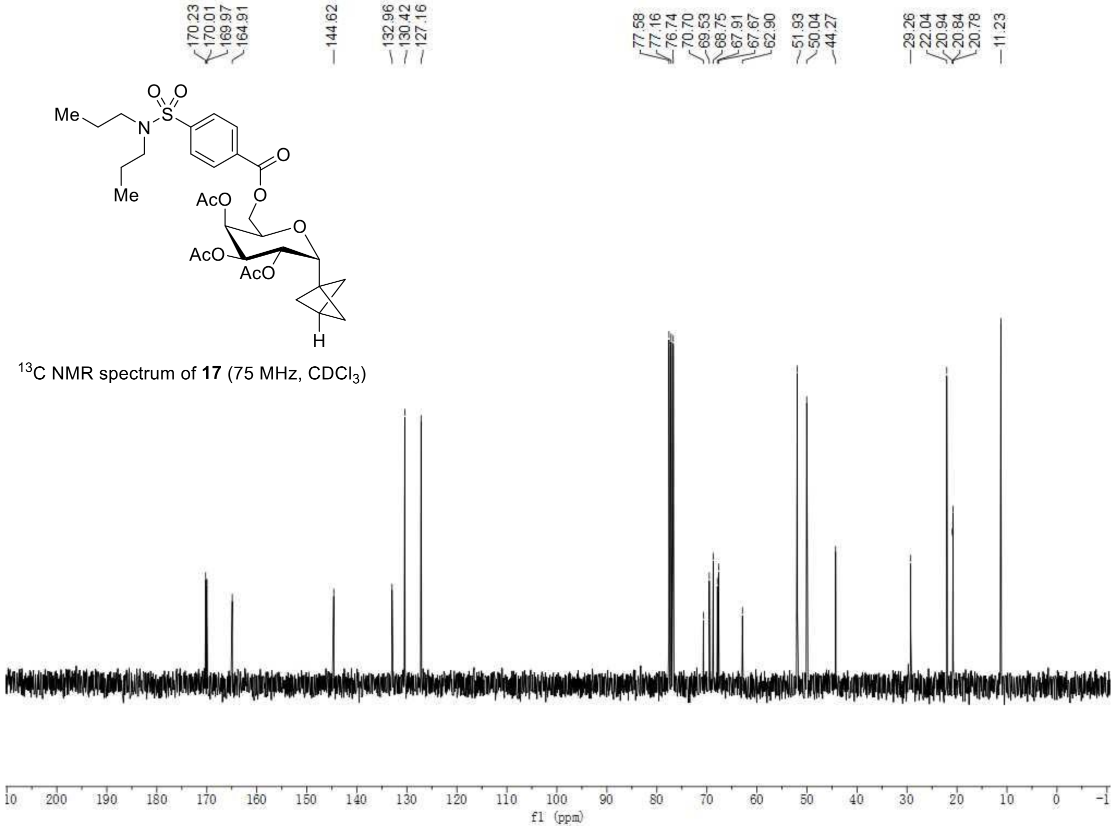

^{13}C NMR spectrum of **17** (75 MHz, $CDCl_3$)

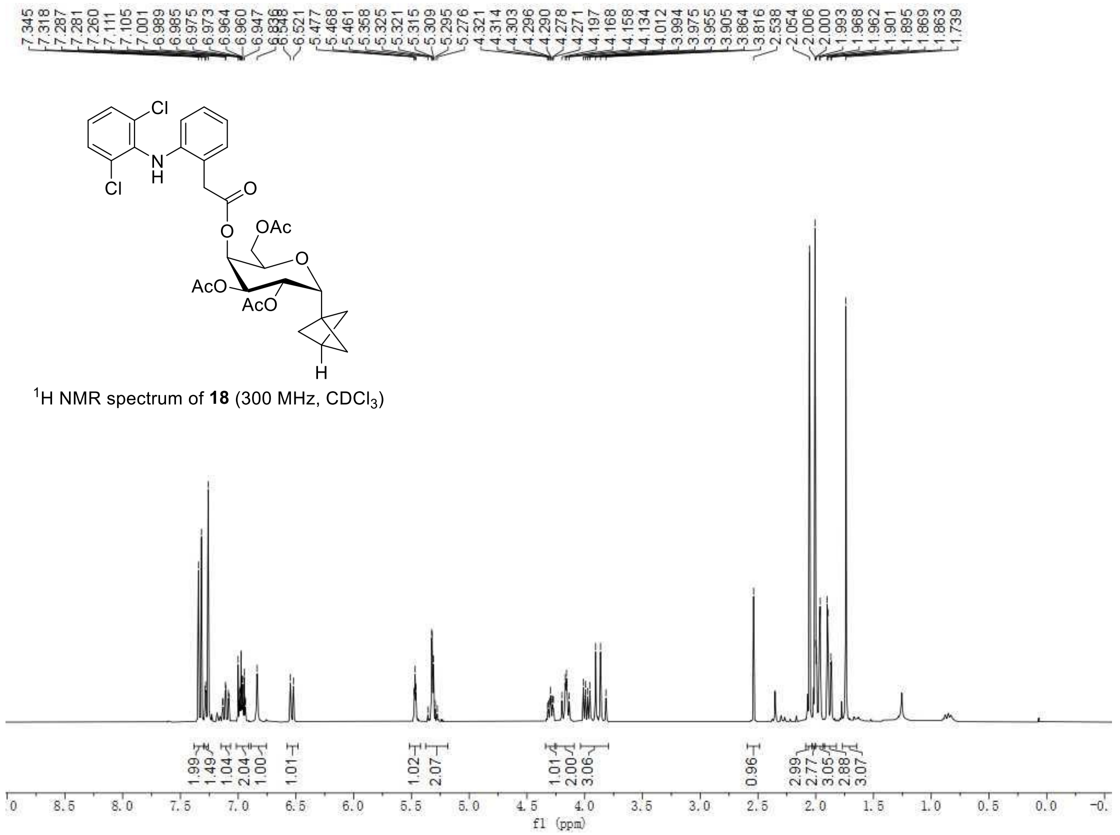

^{1}H NMR spectrum of **18** (300 MHz, $CDCl_3$)

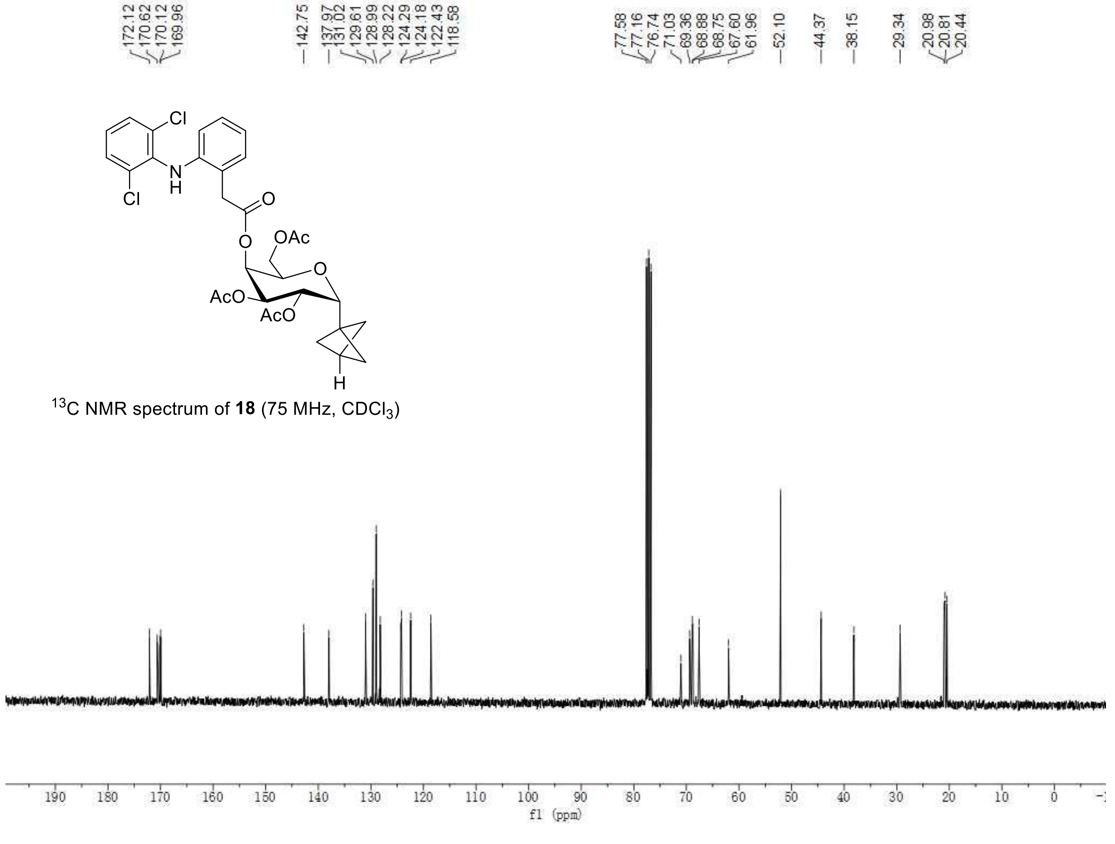

^{13}C NMR spectrum of **18** (75 MHz, $CDCl_3$)

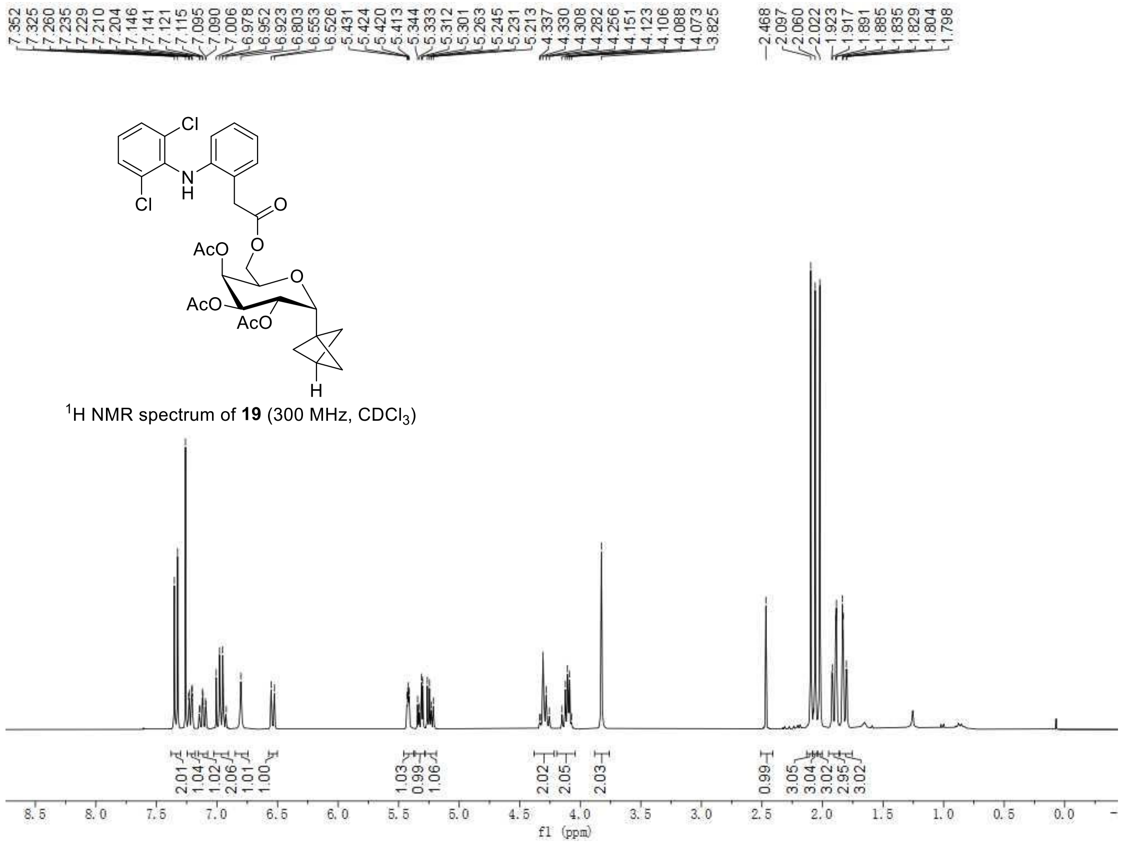

^{1}H NMR spectrum of **19** (300 MHz, $CDCl_3$)

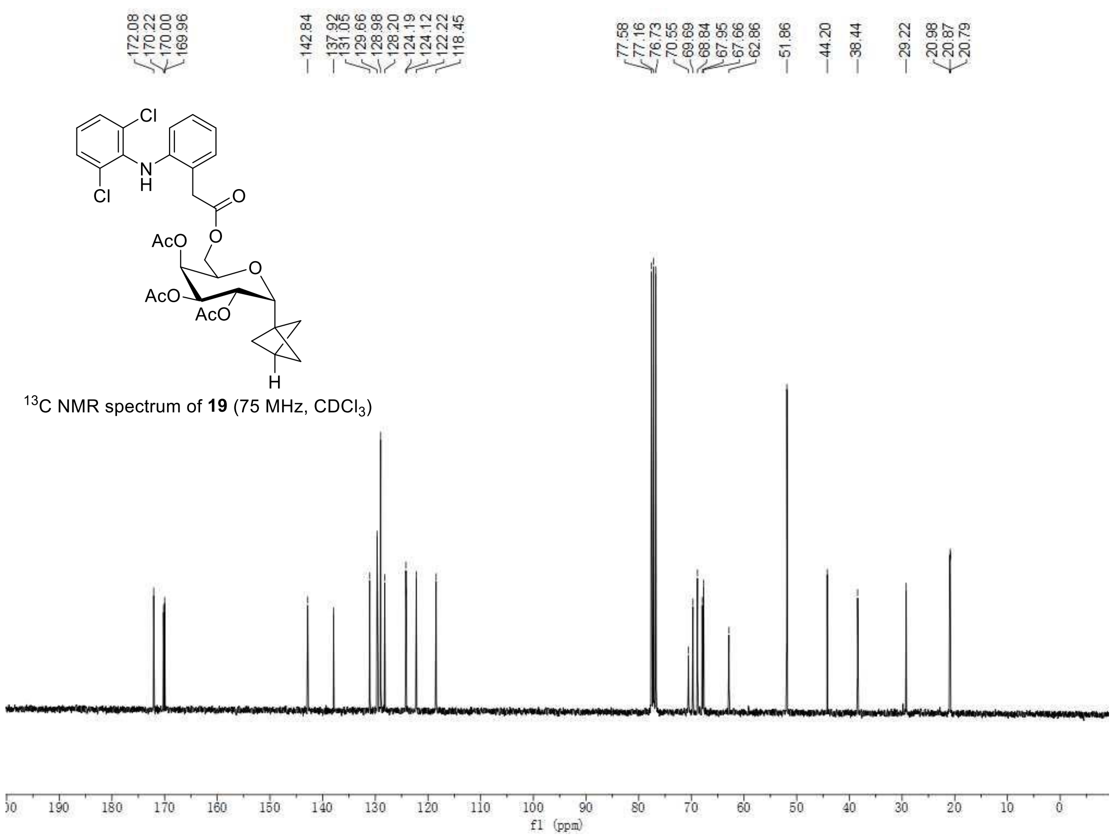

^{13}C NMR spectrum of **19** (75 MHz, $CDCl_3$)

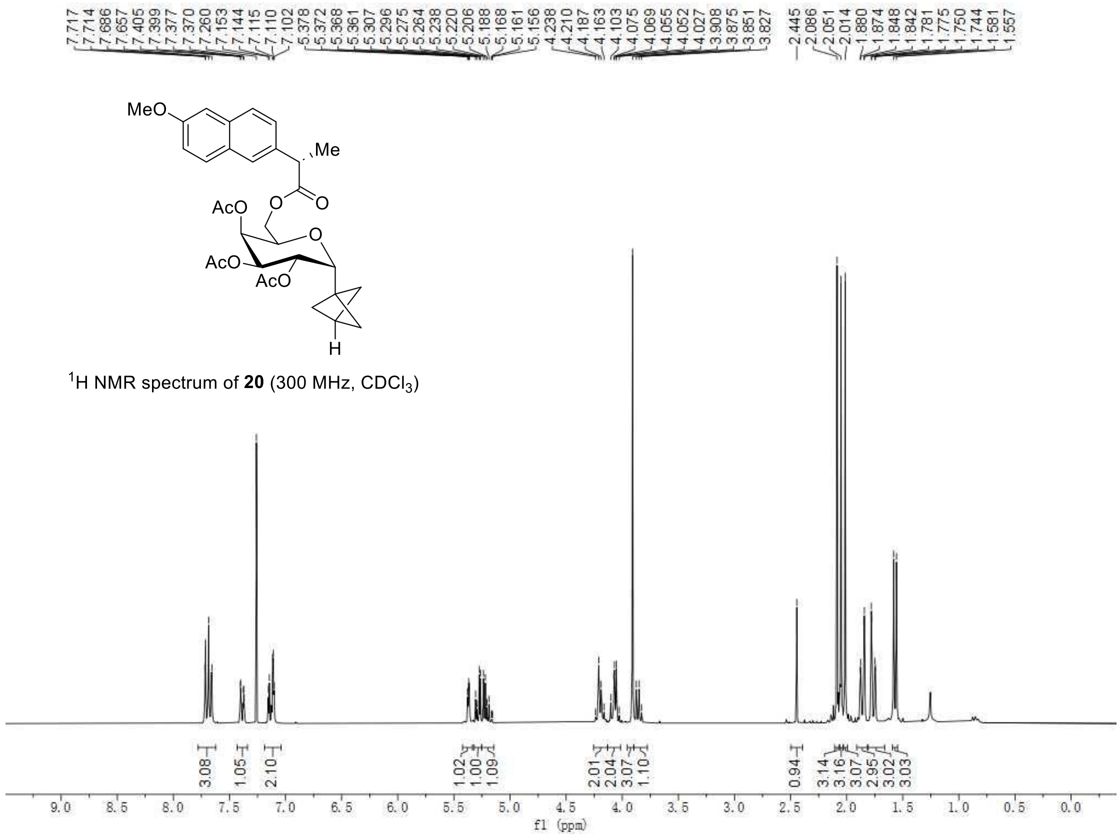

^{1}H NMR spectrum of **20** (300 MHz, $CDCl_3$)

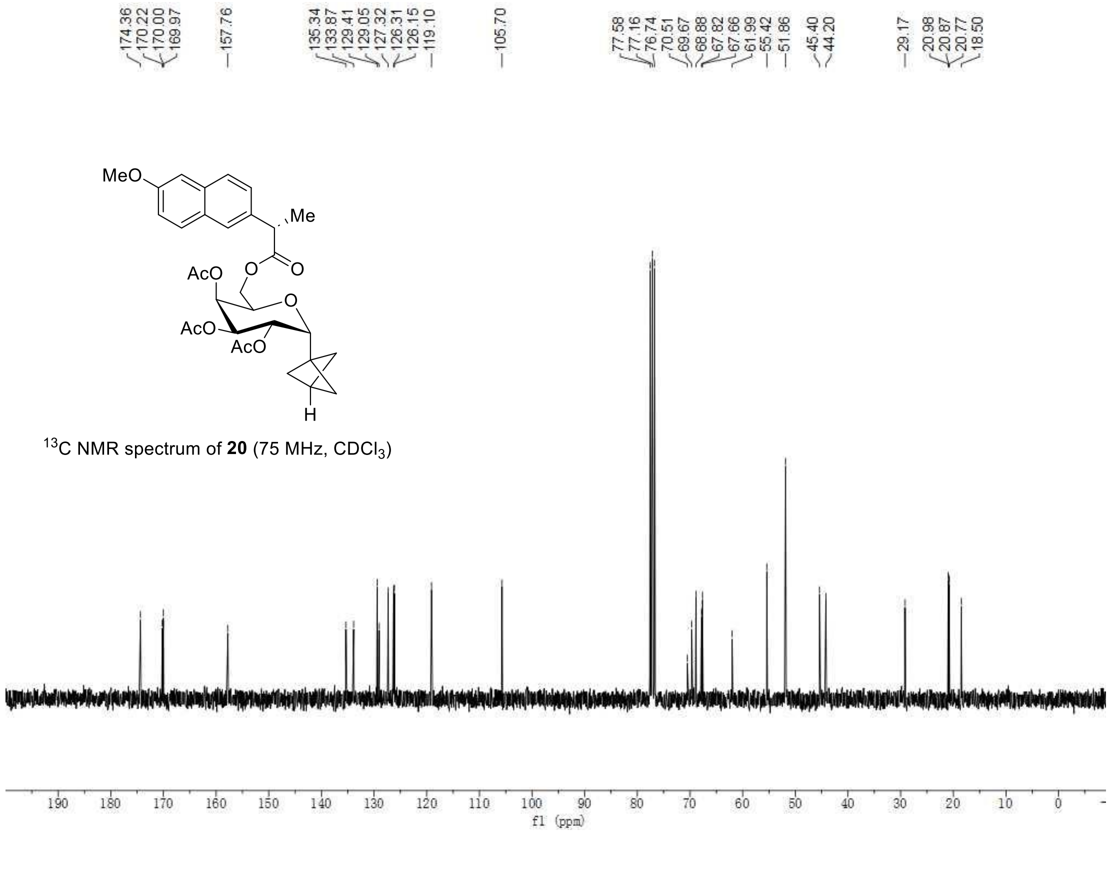

^{13}C NMR spectrum of **20** (75 MHz, $CDCl_3$)

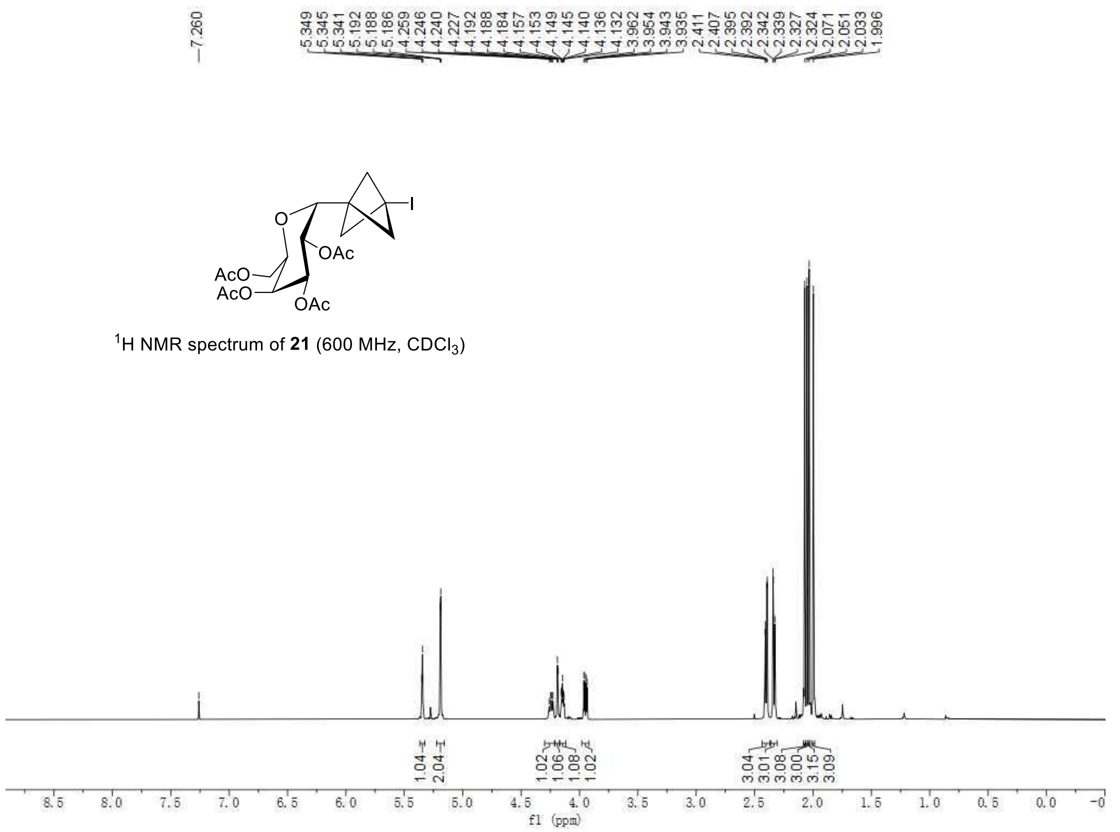

^{1}H NMR spectrum of **21** (600 MHz, $CDCl_3$)

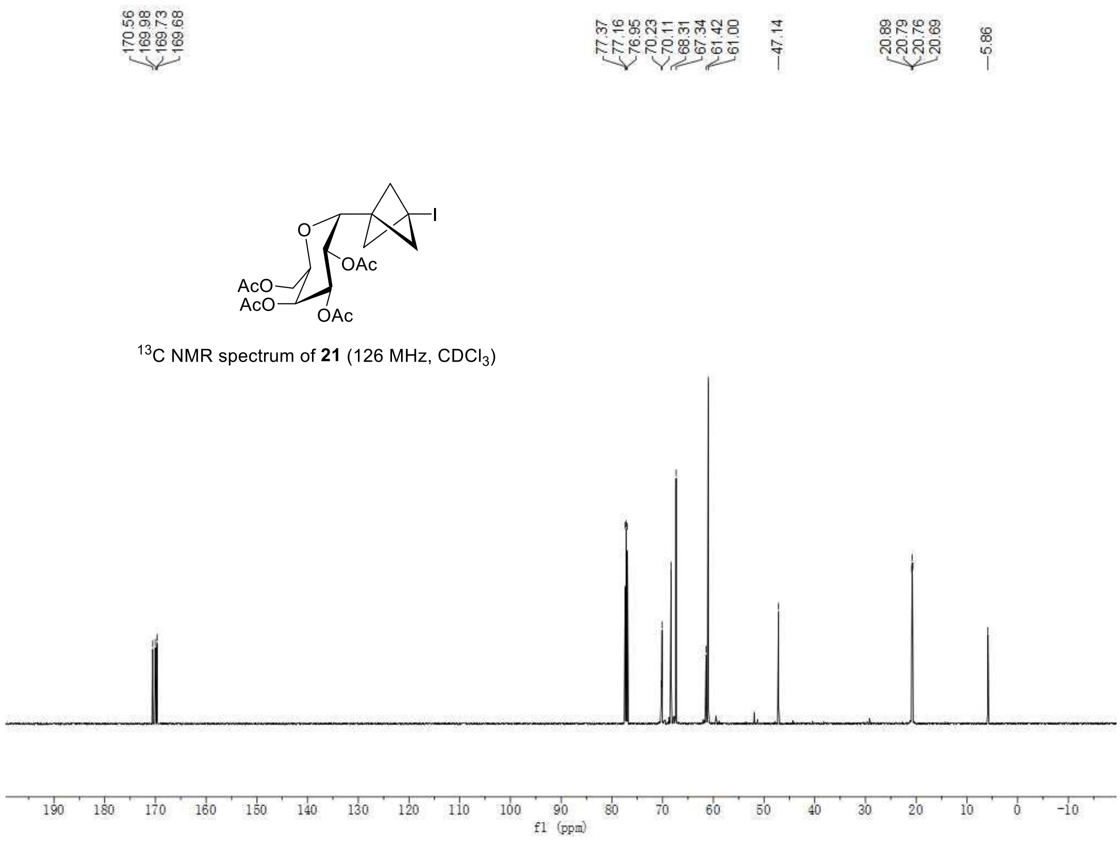

^{13}C NMR spectrum of **21** (126 MHz, $CDCl_3$)

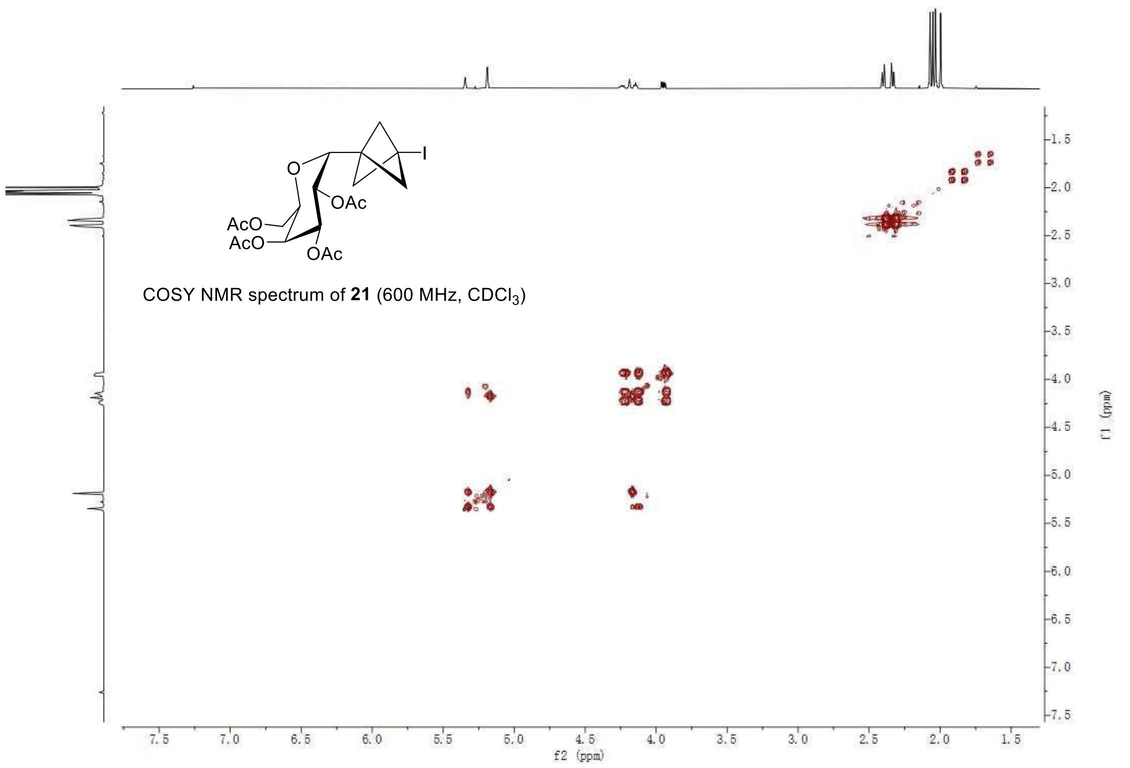
AcO
AcO
OAc
OAc
I
COSY NMR spectrum of 21 (600 MHz, CDCl3)
f2 (ppm)
f1 (ppm)

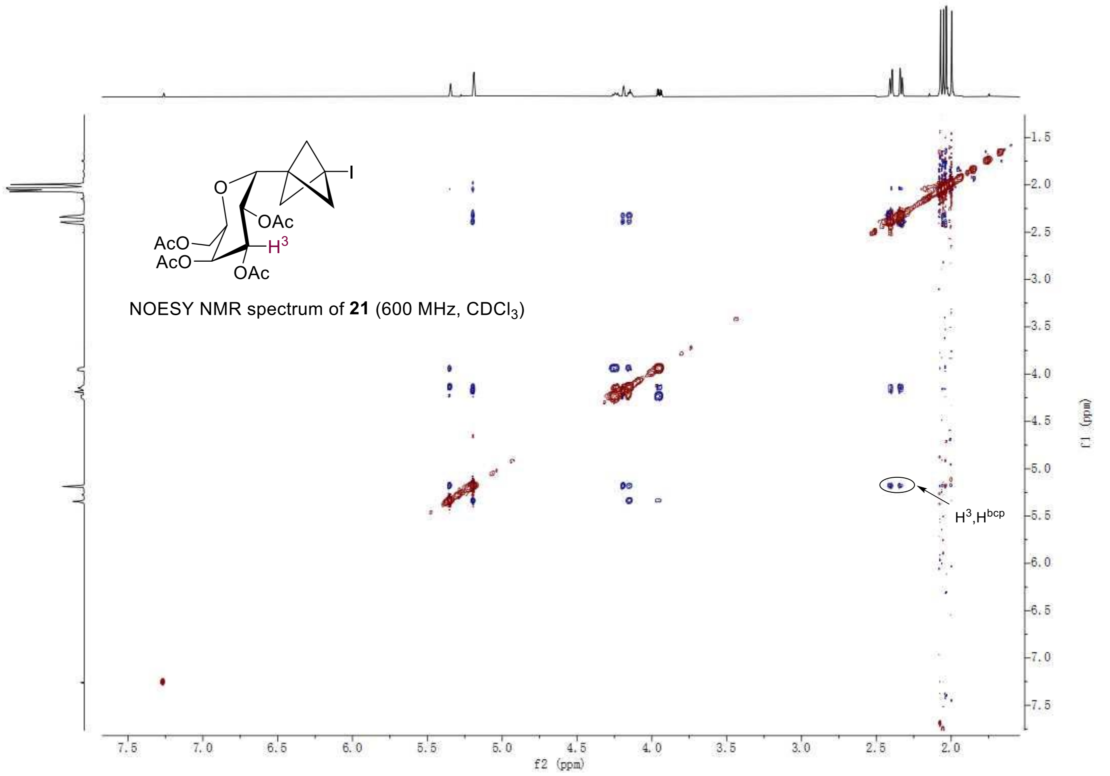
AcO
AcO
OAc
OAc
H3
I
NOESY NMR spectrum of 21 (600 MHz, CDCl3)
H3,Hbcp
f2 (ppm)
f1 (ppm)

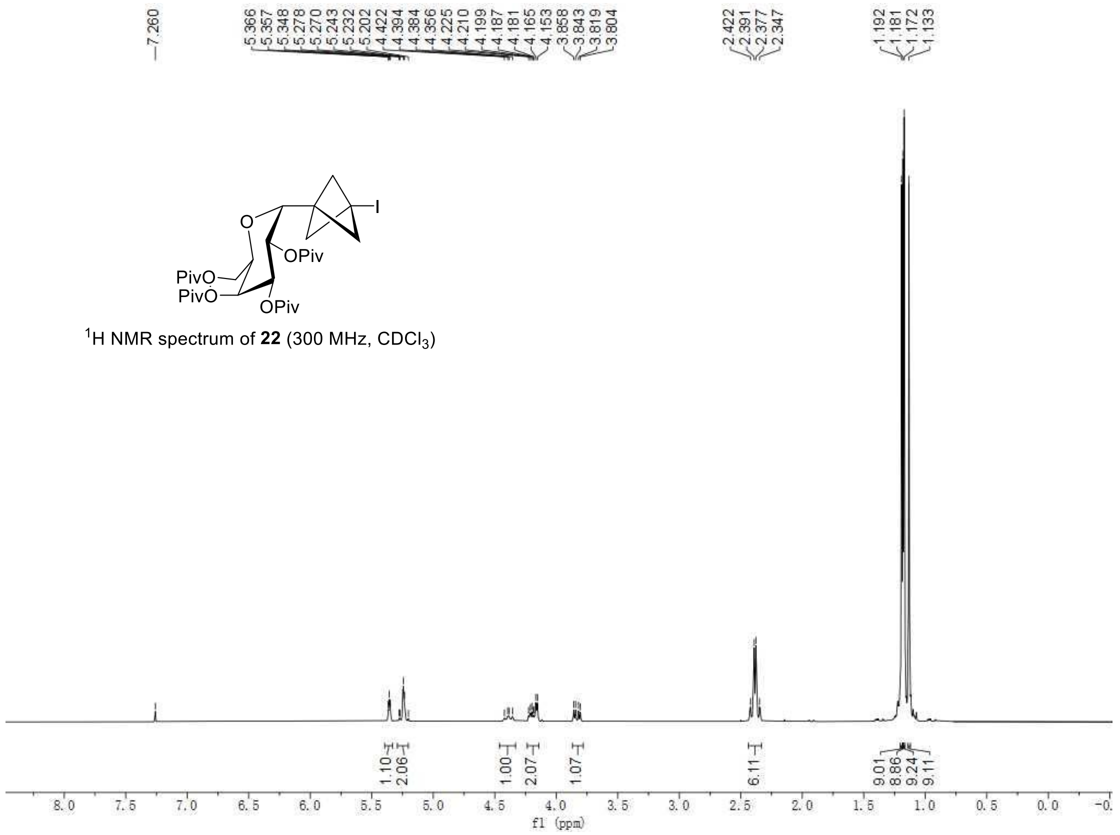

^{1}H NMR spectrum of **22** (300 MHz, $CDCl_3$)

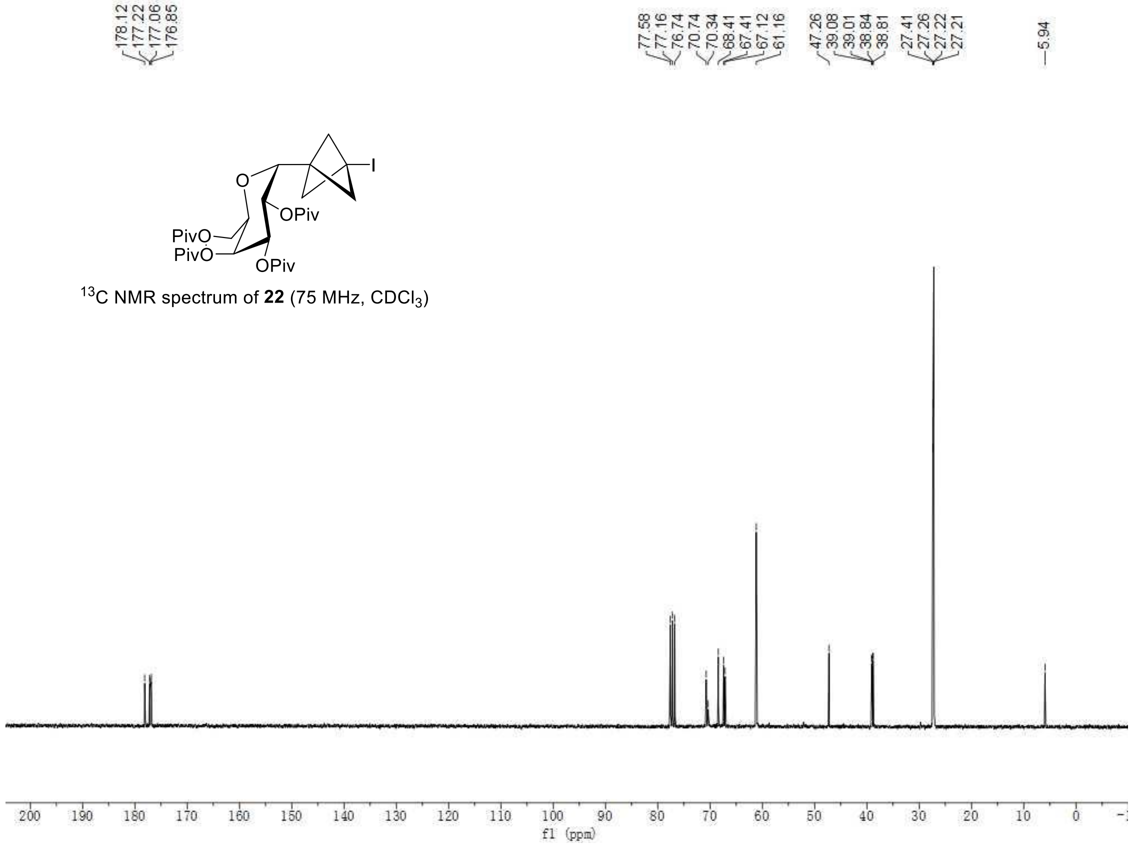

^{13}C NMR spectrum of **22** (75 MHz, $CDCl_3$)

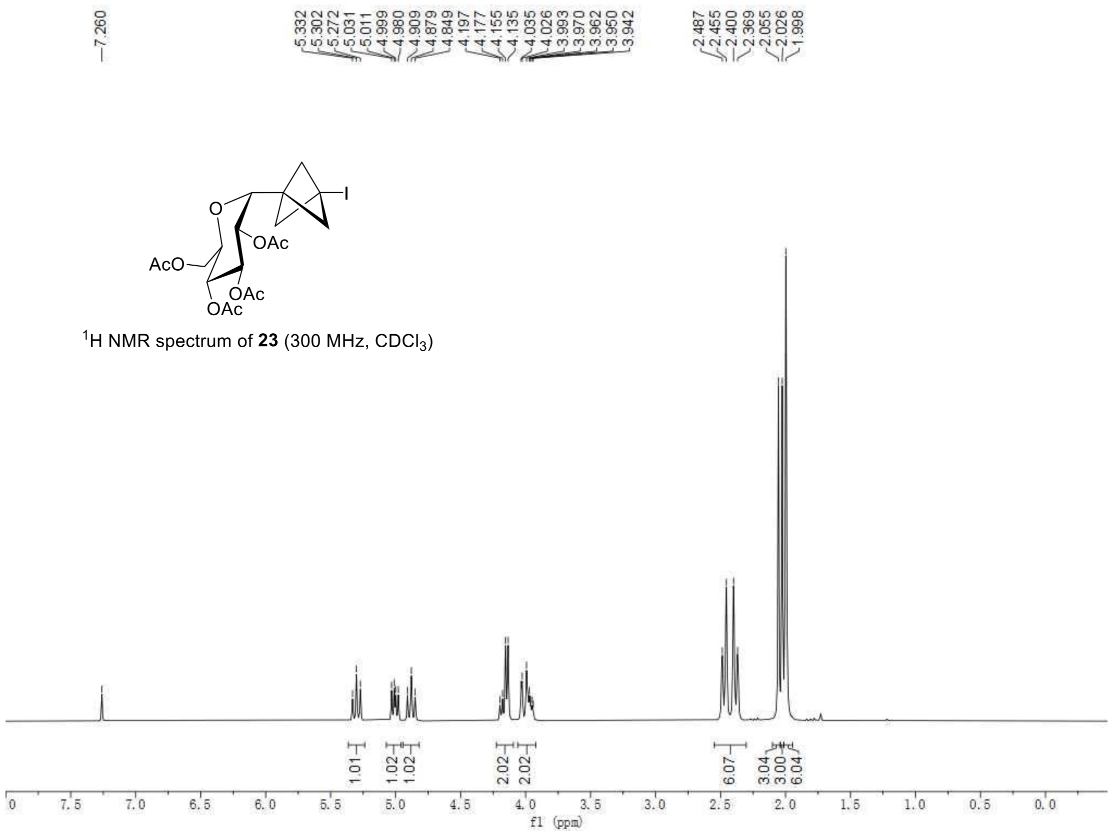

^{1}H NMR spectrum of **23** (300 MHz, $CDCl_3$)

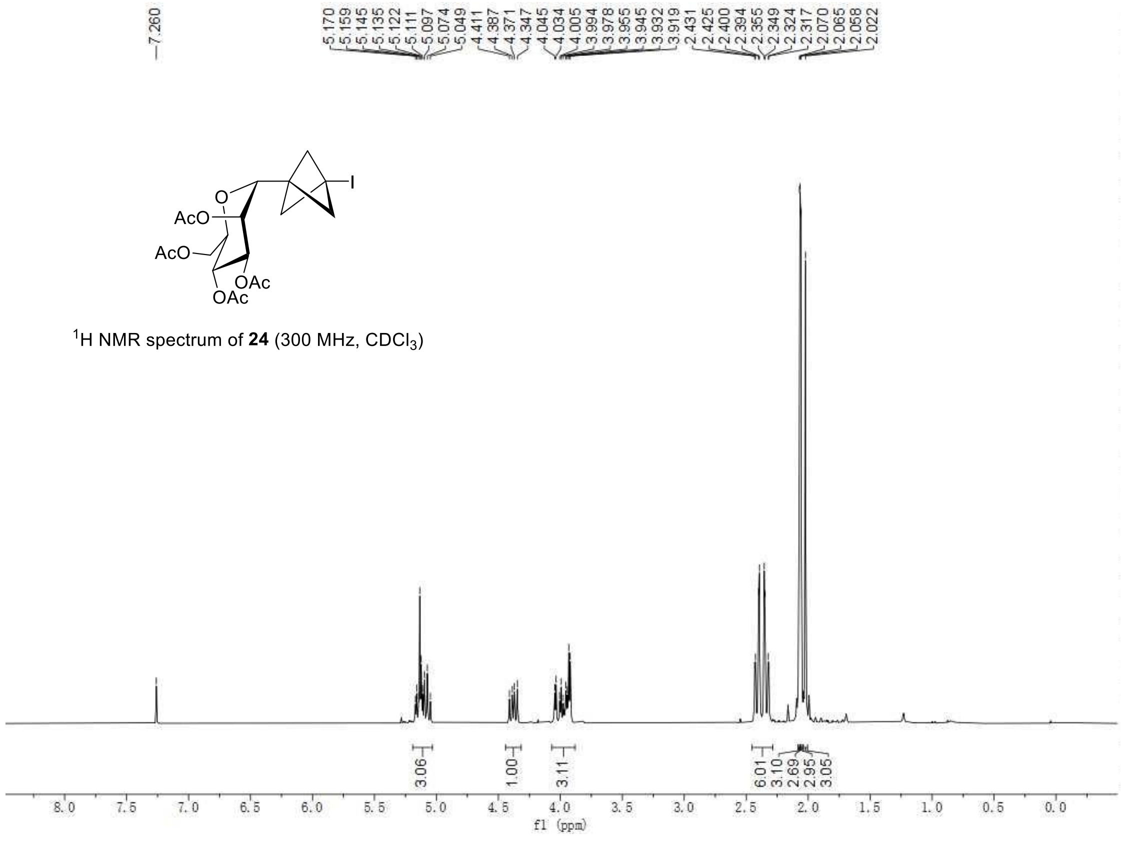

^{1}H NMR spectrum of **24** (300 MHz, $CDCl_3$)

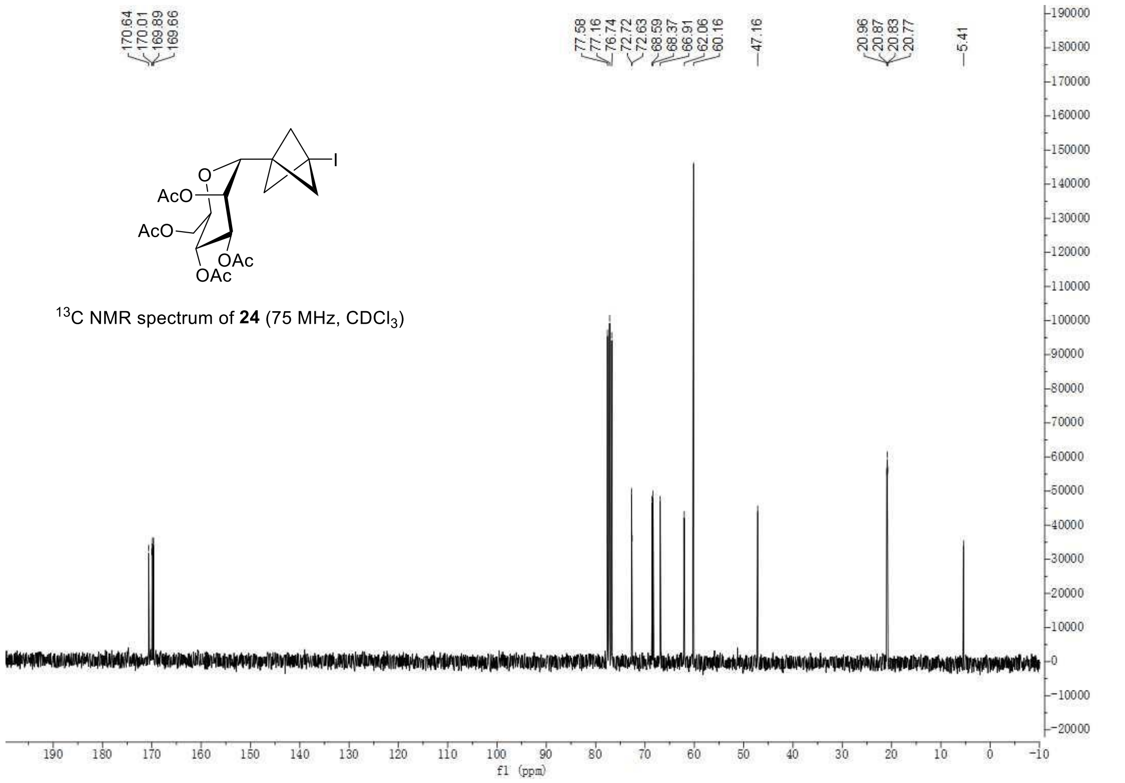

^{13}C NMR spectrum of **24** (75 MHz, $CDCl_3$)

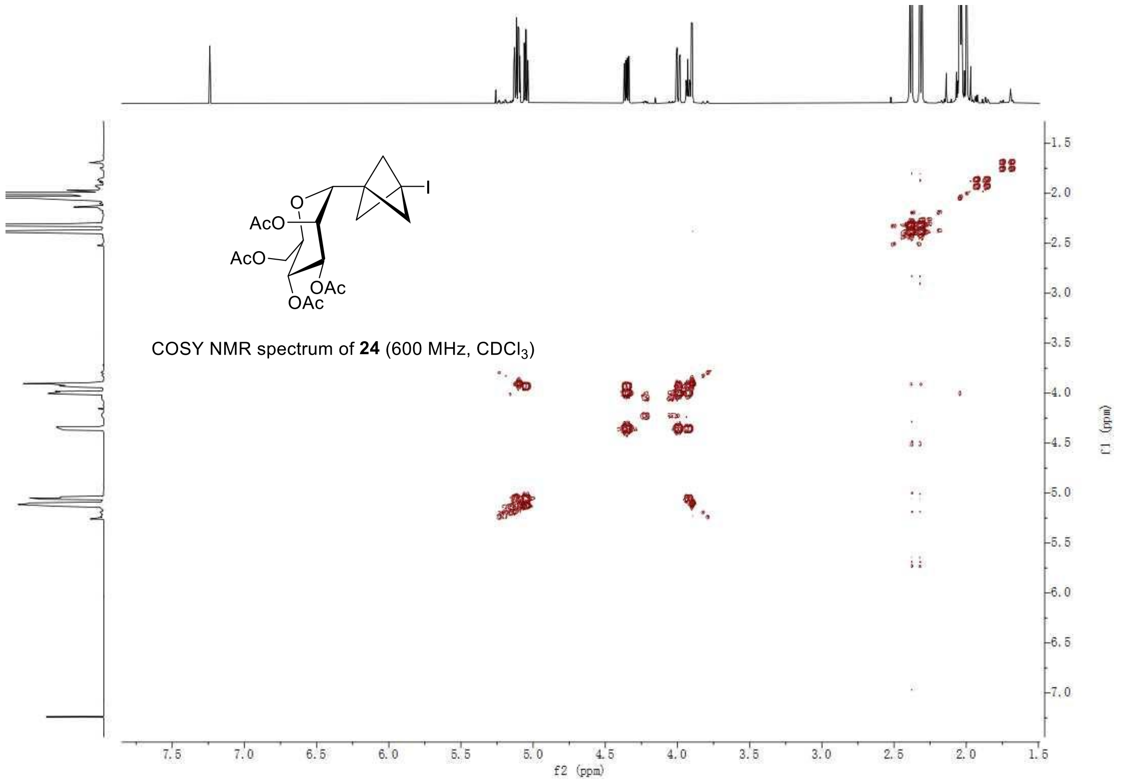

COSY NMR spectrum of **24** (600 MHz, $CDCl_3$)

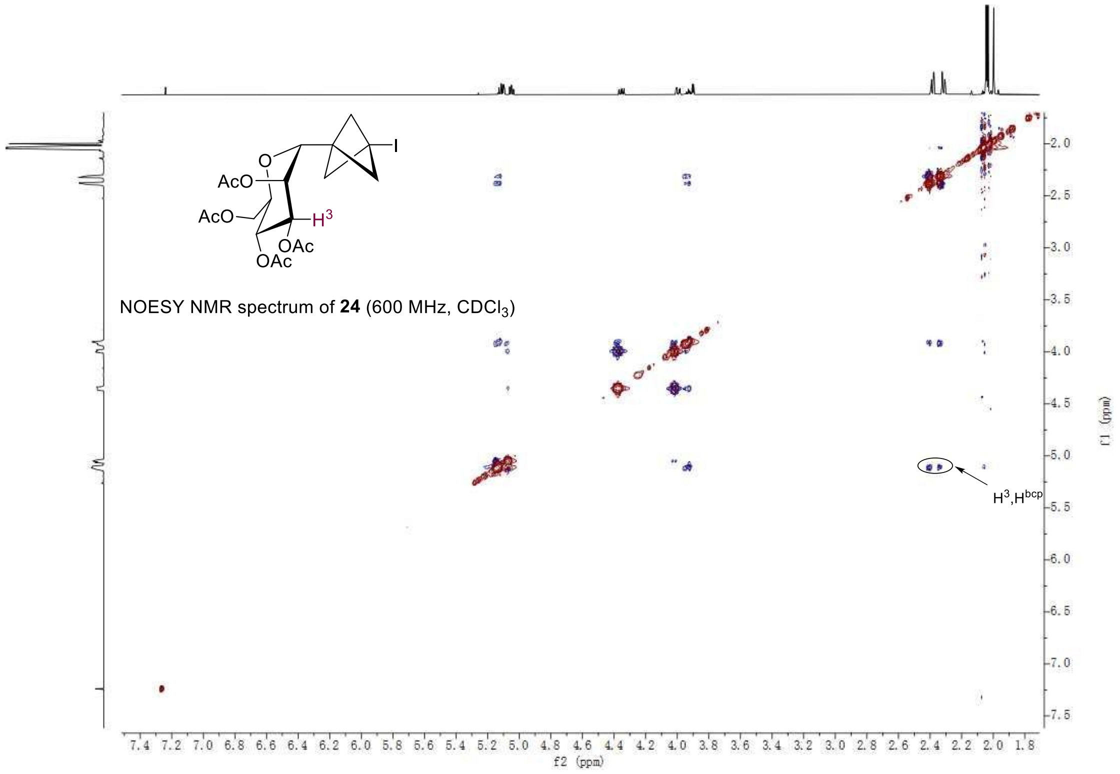

NOESY NMR spectrum of **24** (600 MHz, $CDCl_3$)

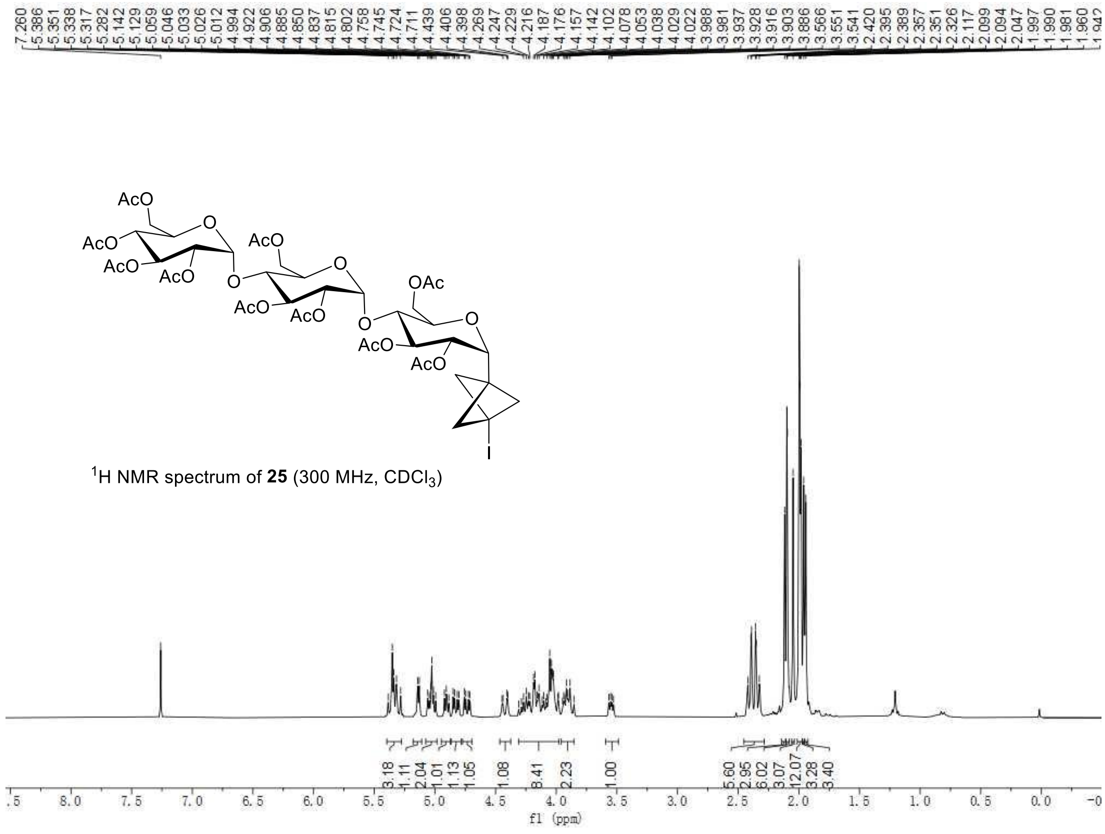

^{1}H NMR spectrum of **25** (300 MHz, $CDCl_3$)

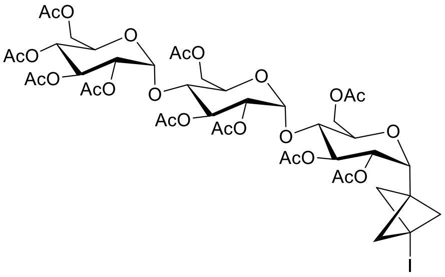

^{13}C NMR spectrum of **25** (75 MHz, $CDCl_3$)

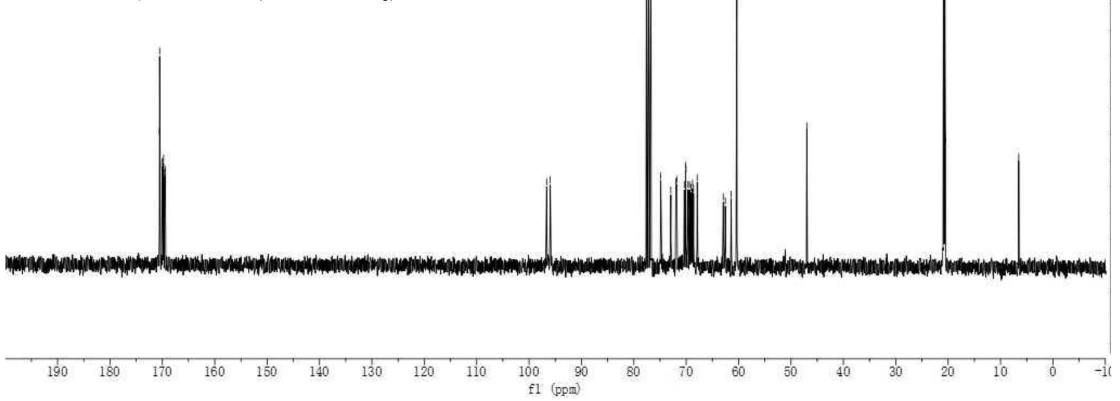

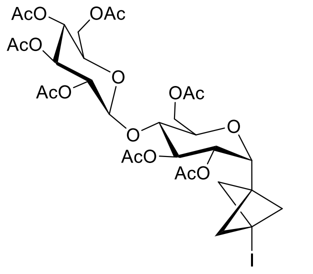

^{1}H NMR spectrum of **26** (300 MHz, $CDCl_3$)

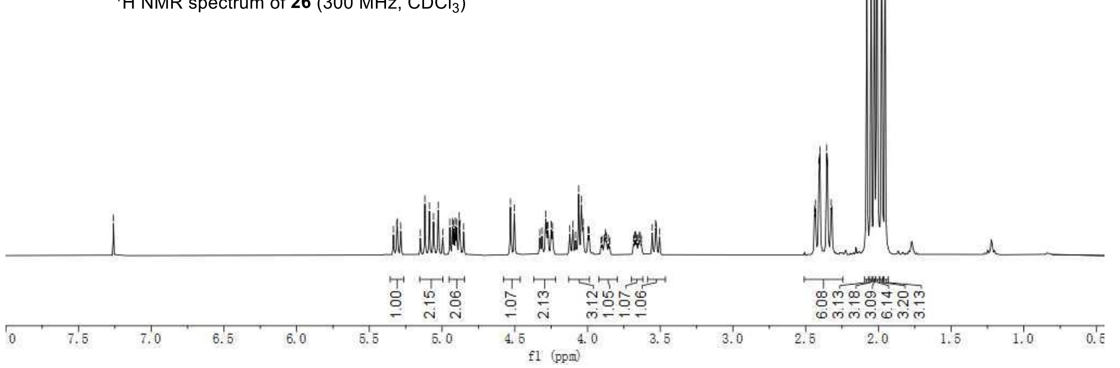

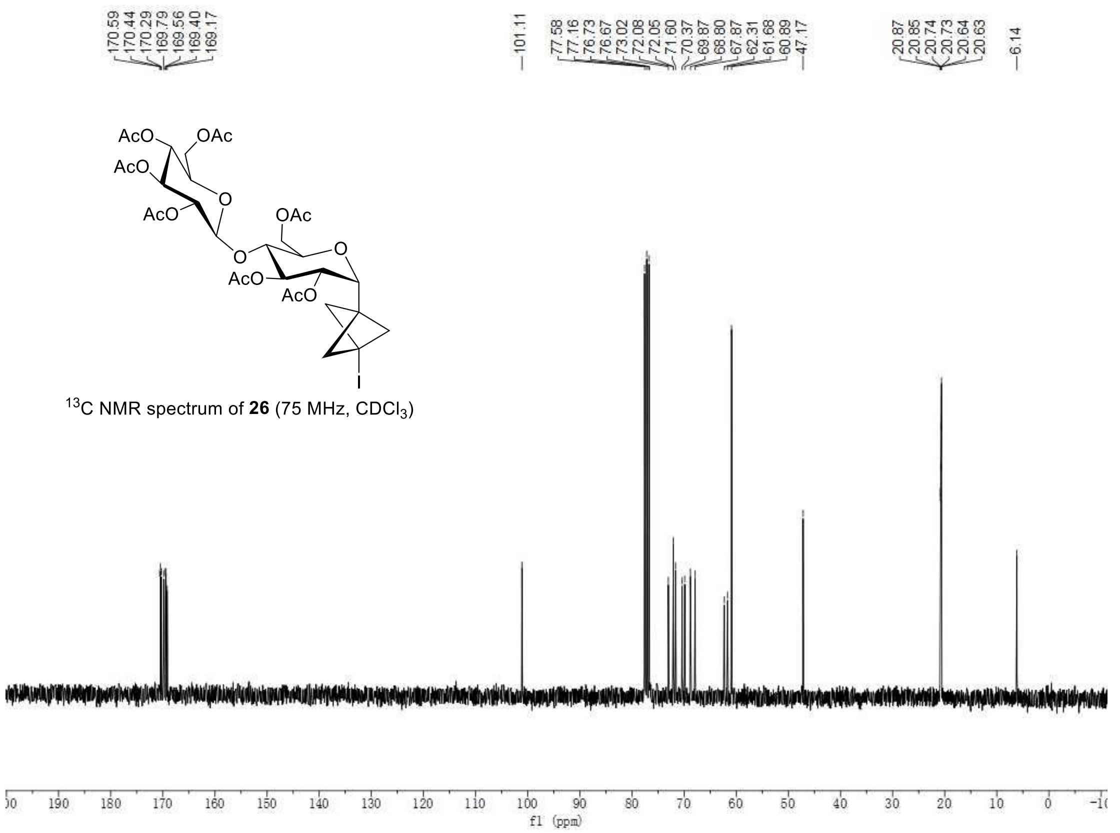

^{13}C NMR spectrum of **26** (75 MHz, $CDCl_3$)

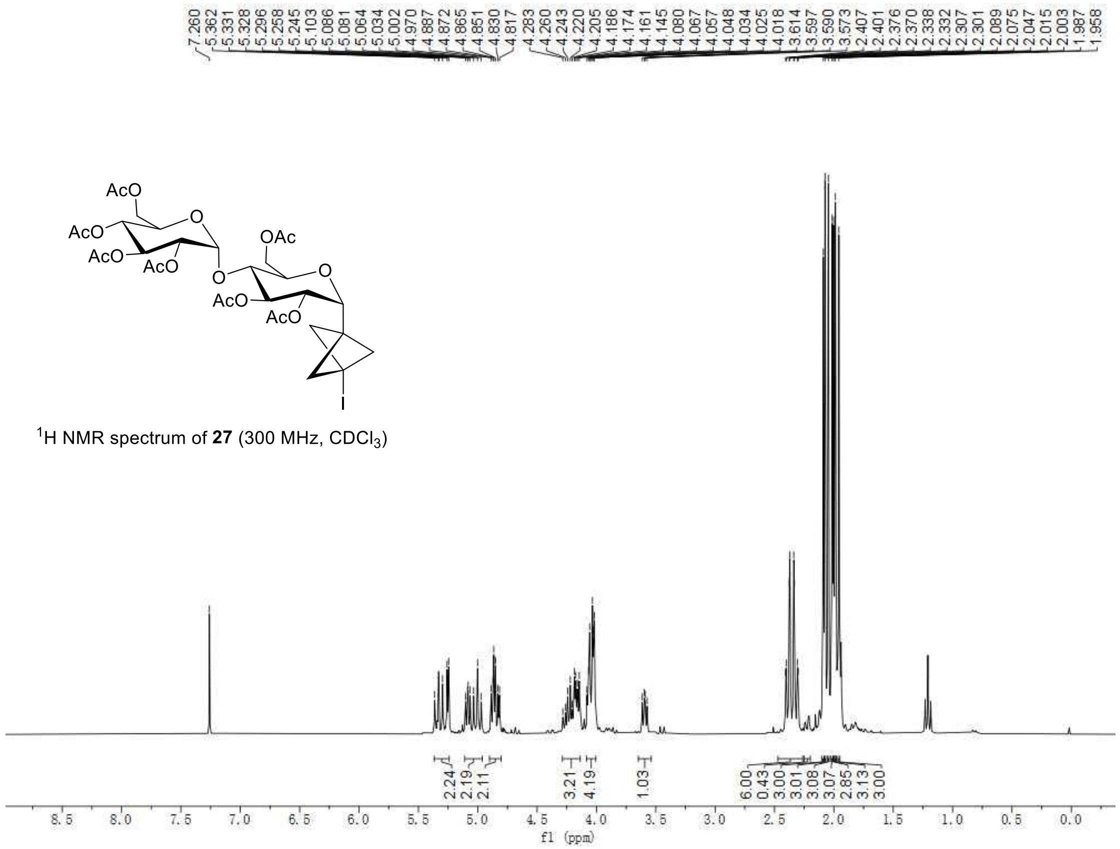

^{1}H NMR spectrum of **27** (300 MHz, $CDCl_3$)

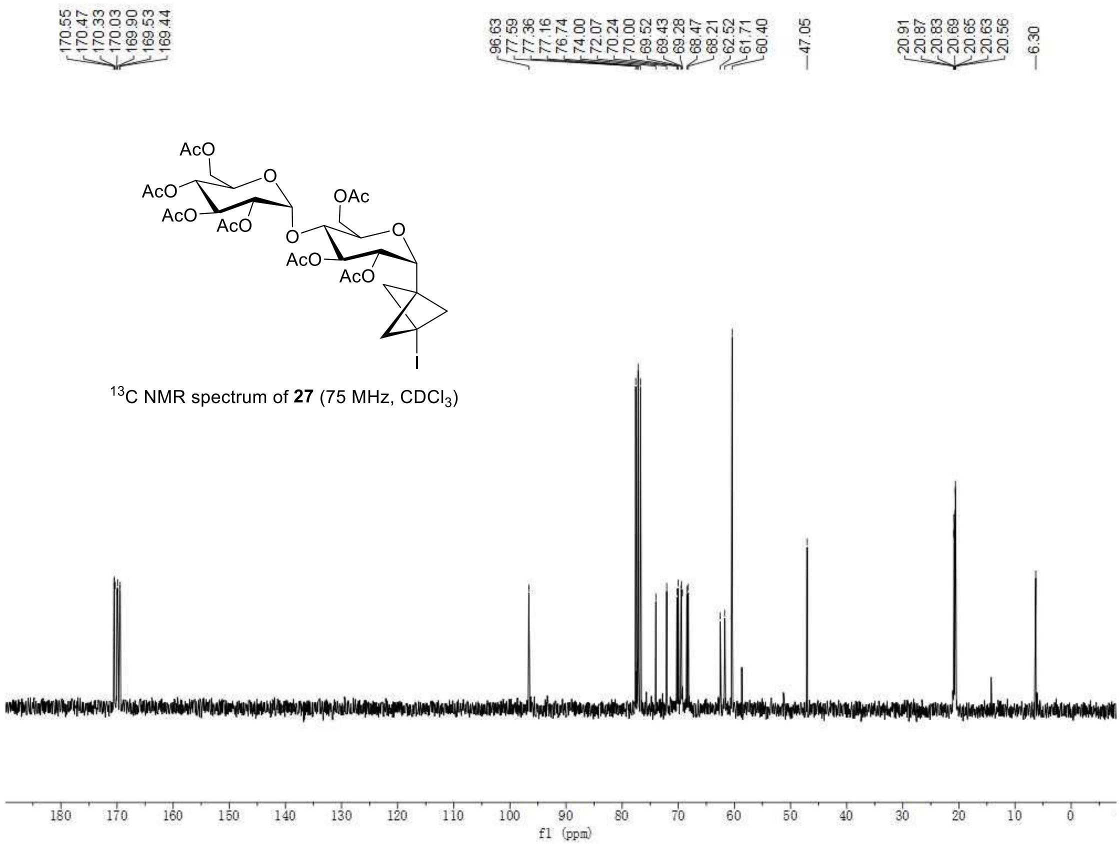

^{13}C NMR spectrum of **27** (75 MHz, $CDCl_3$)

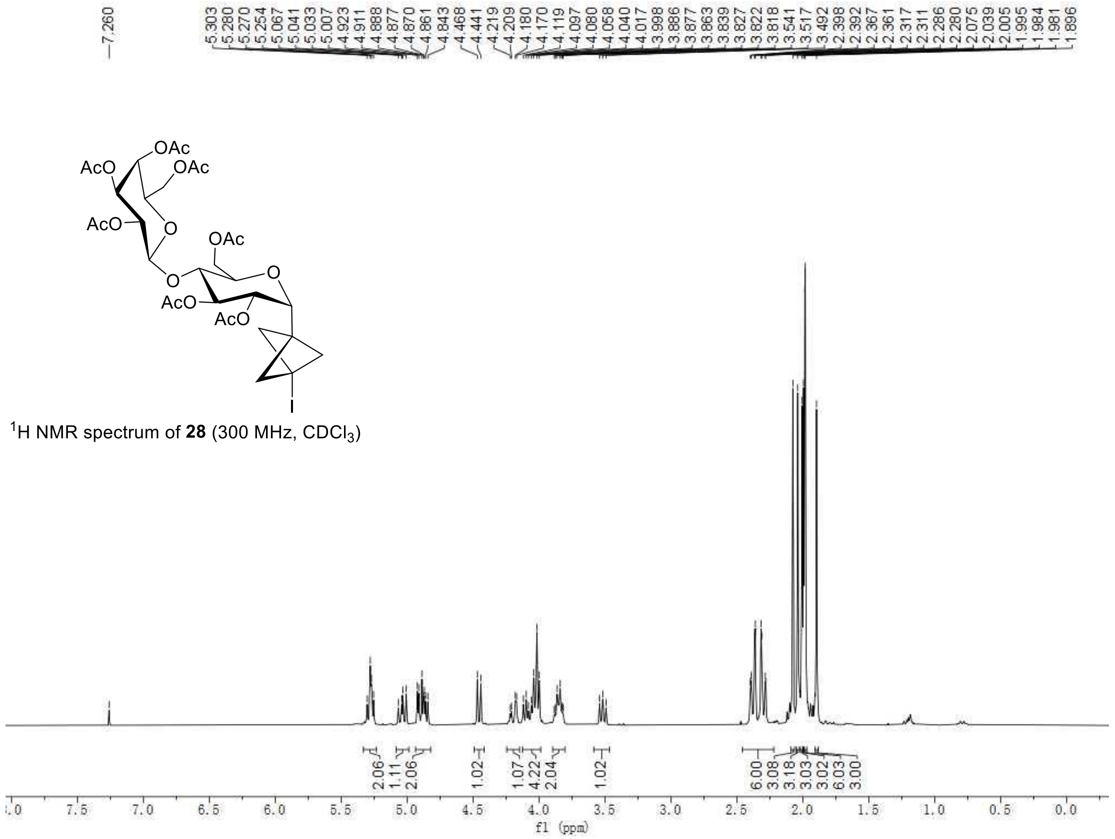

^{1}H NMR spectrum of **28** (300 MHz, $CDCl_3$)

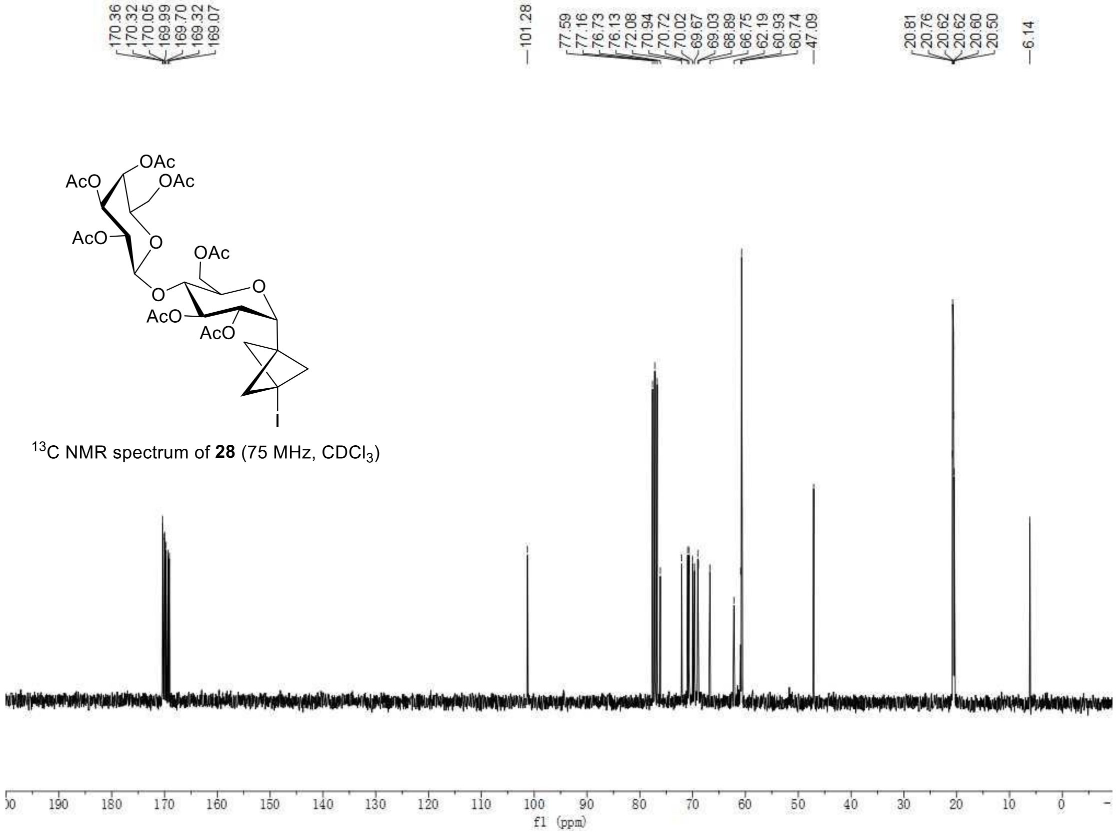

^{13}C NMR spectrum of **28** (75 MHz, $CDCl_3$)

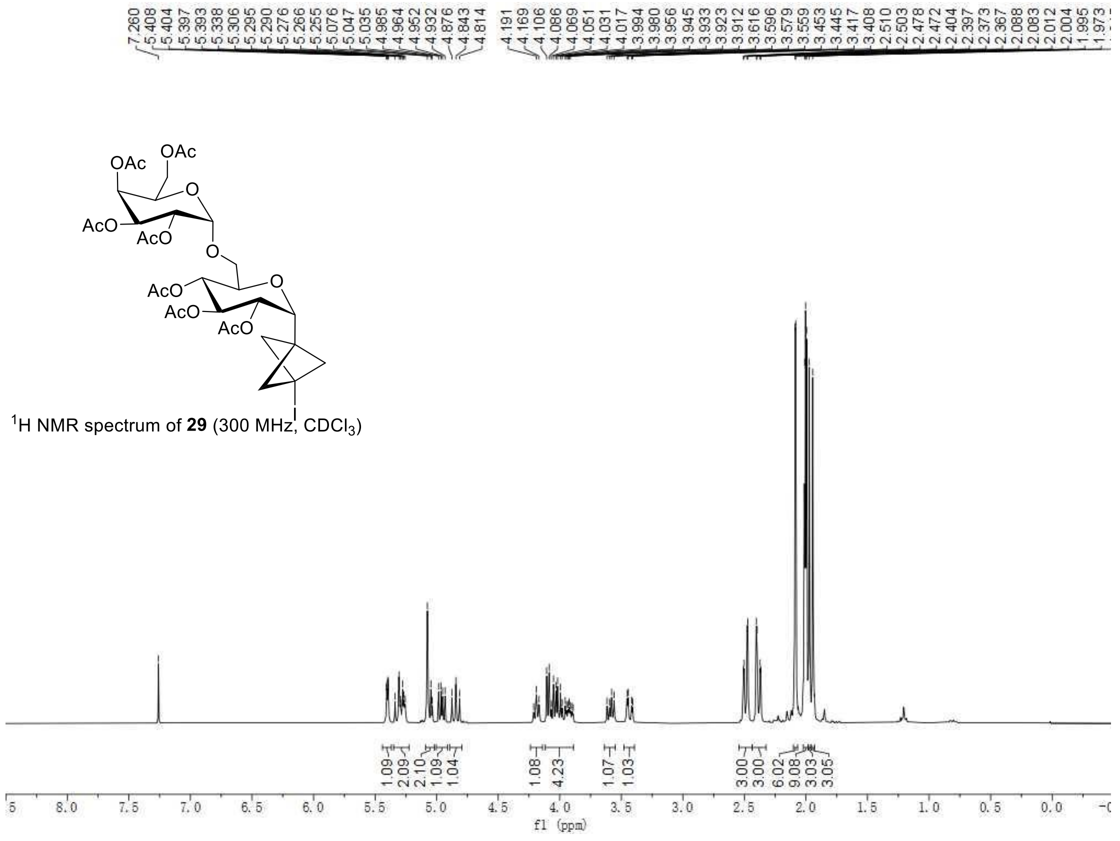

^{1}H NMR spectrum of **29** (300 MHz, $CDCl_3$)

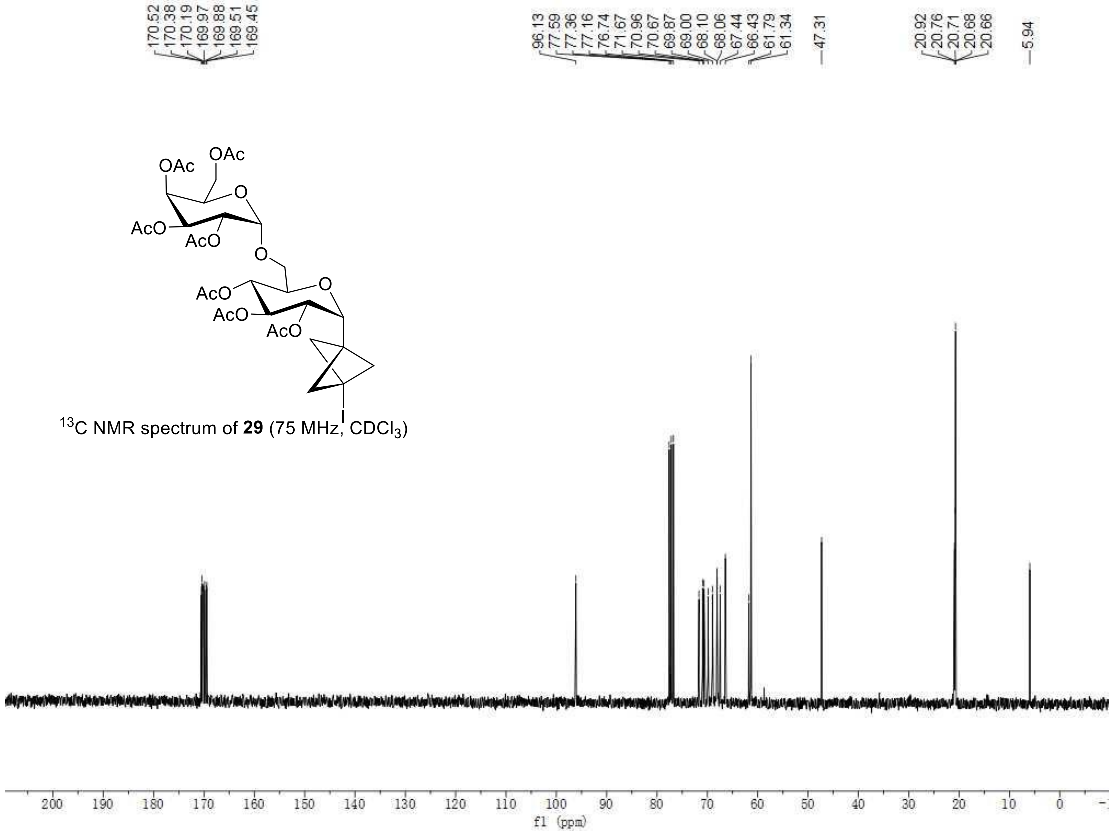

^{13}C NMR spectrum of **29** (75 MHz, $CDCl_3$)

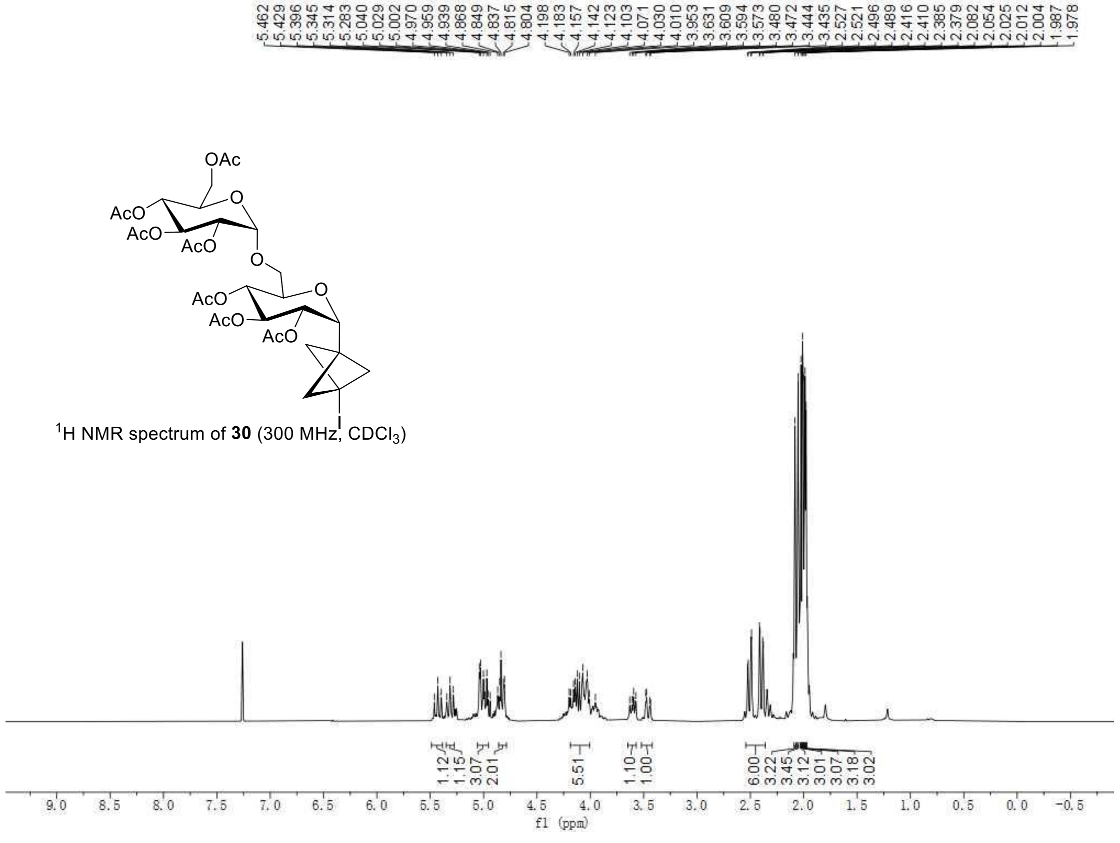

^{1}H NMR spectrum of **30** (300 MHz, $CDCl_3$)

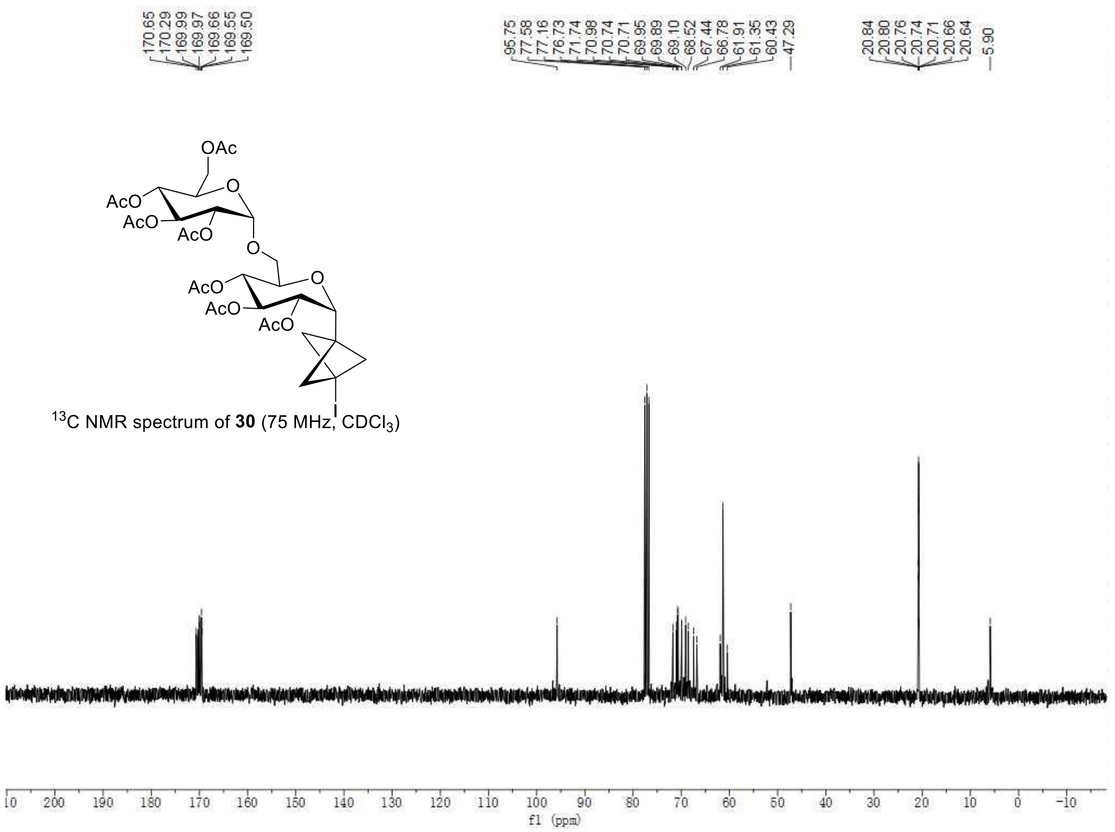

^{13}C NMR spectrum of **30** (75 MHz, $CDCl_3$)

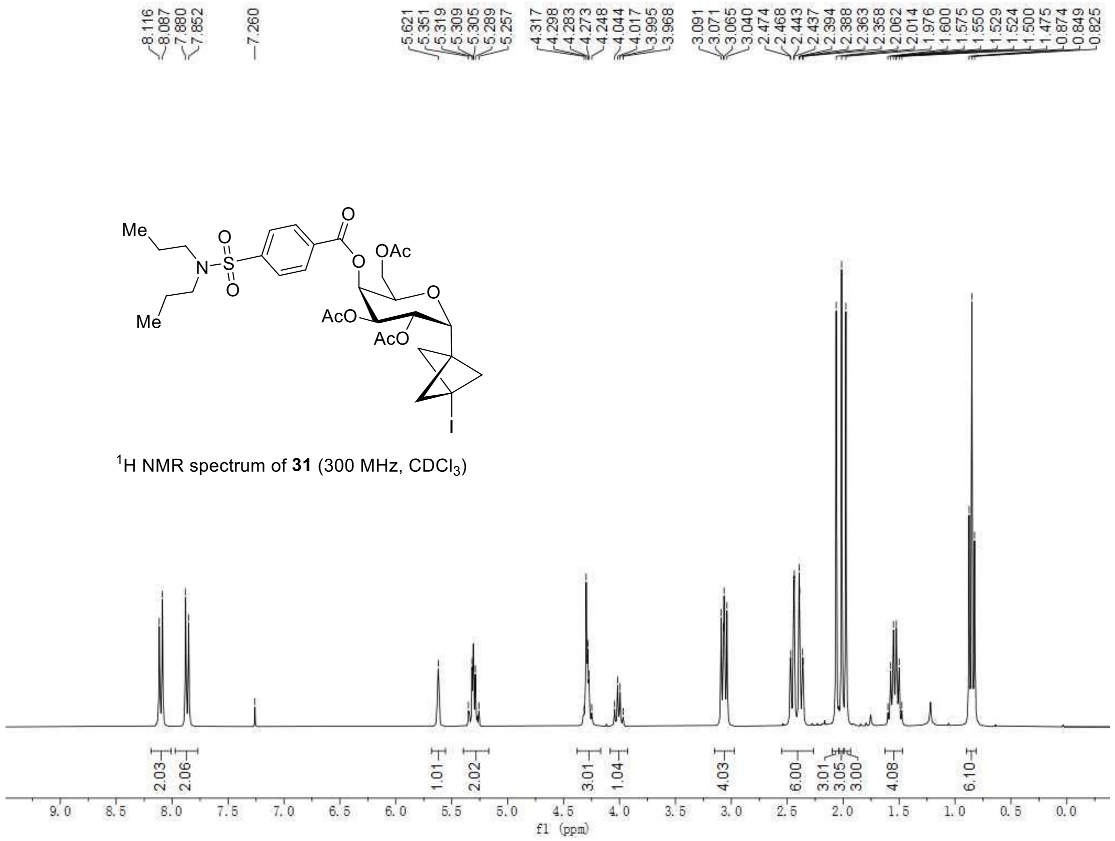

^{1}H NMR spectrum of **31** (300 MHz, $CDCl_3$)

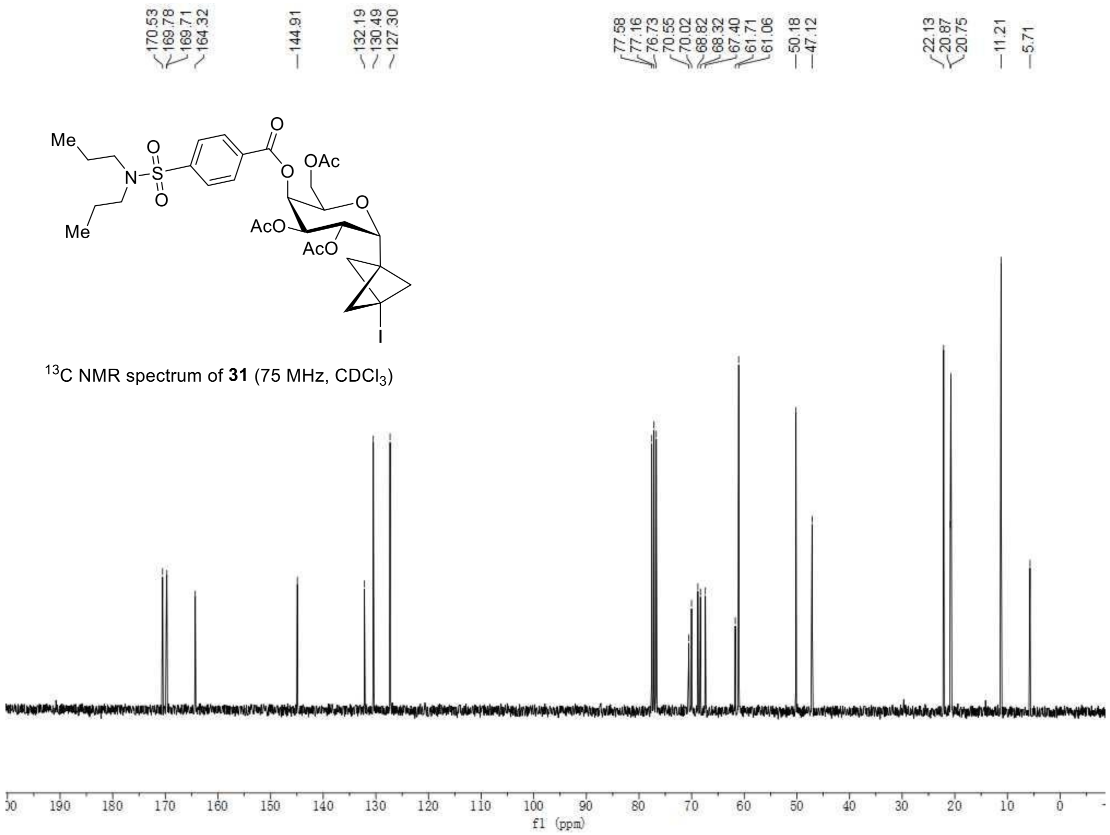

^{13}C NMR spectrum of **31** (75 MHz, $CDCl_3$)

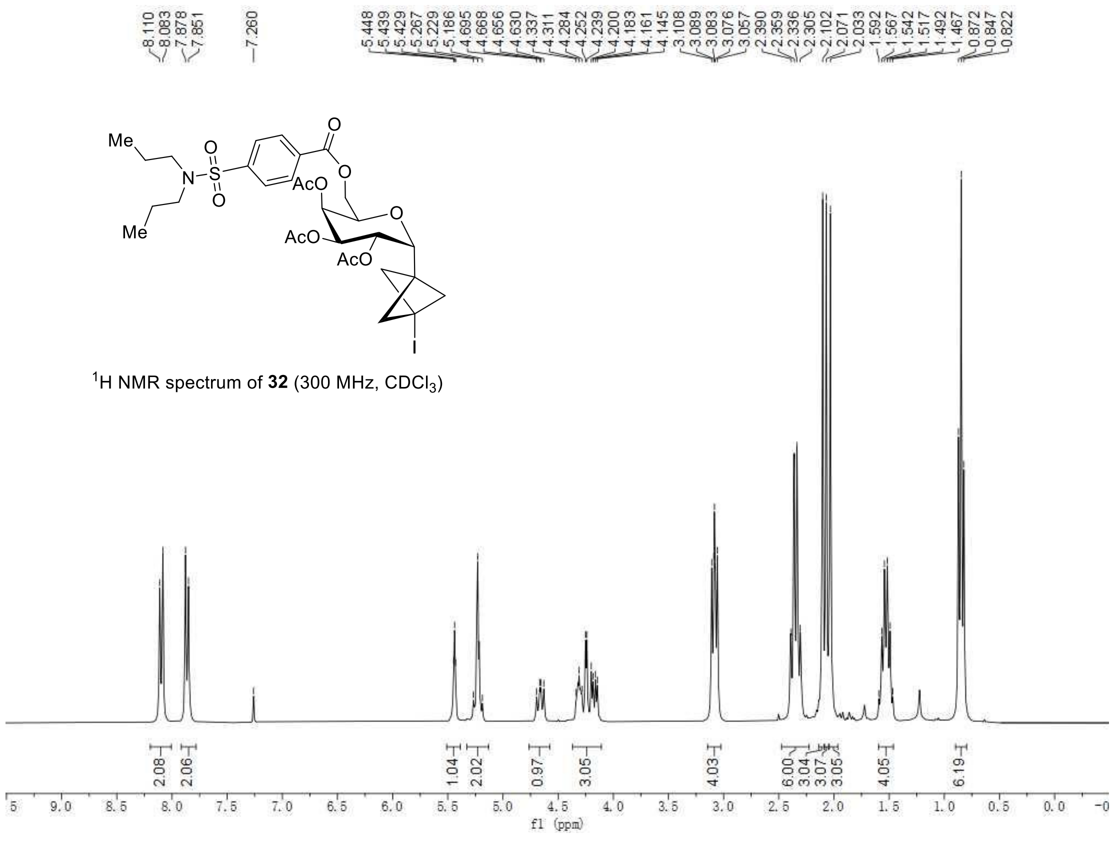

^{1}H NMR spectrum of **32** (300 MHz, $CDCl_3$)

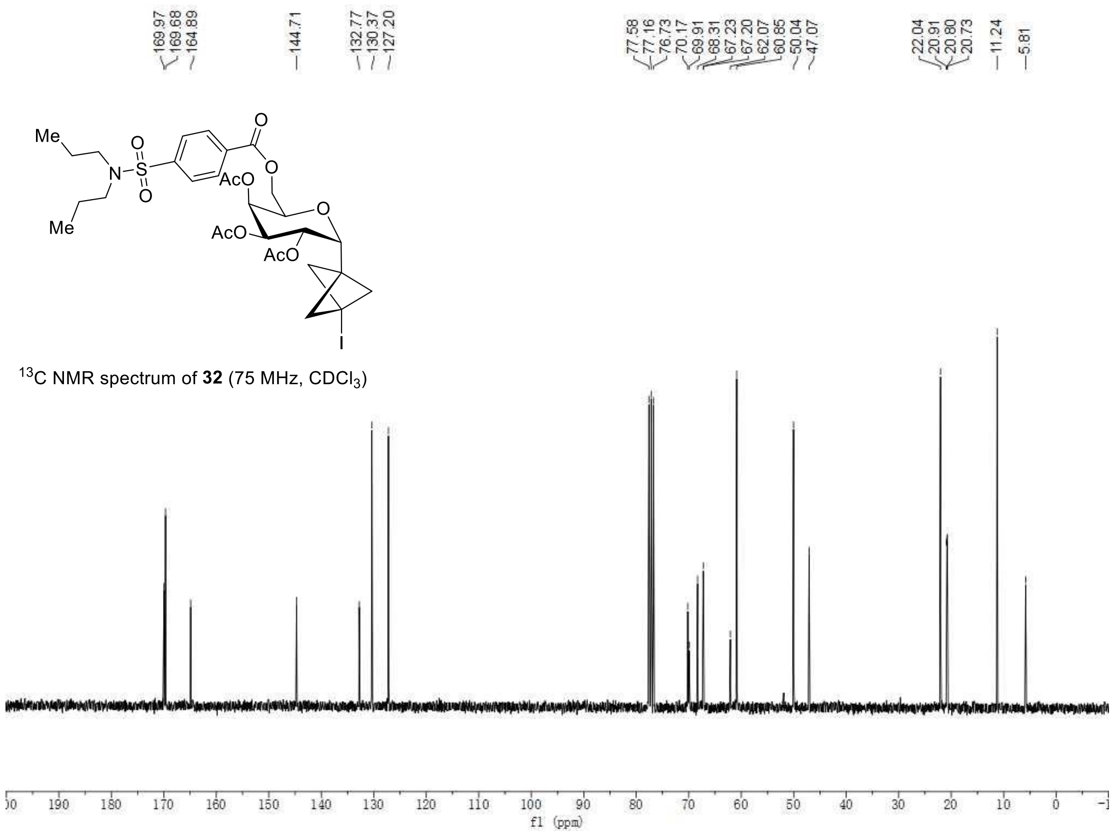

^{13}C NMR spectrum of **32** (75 MHz, $CDCl_3$)

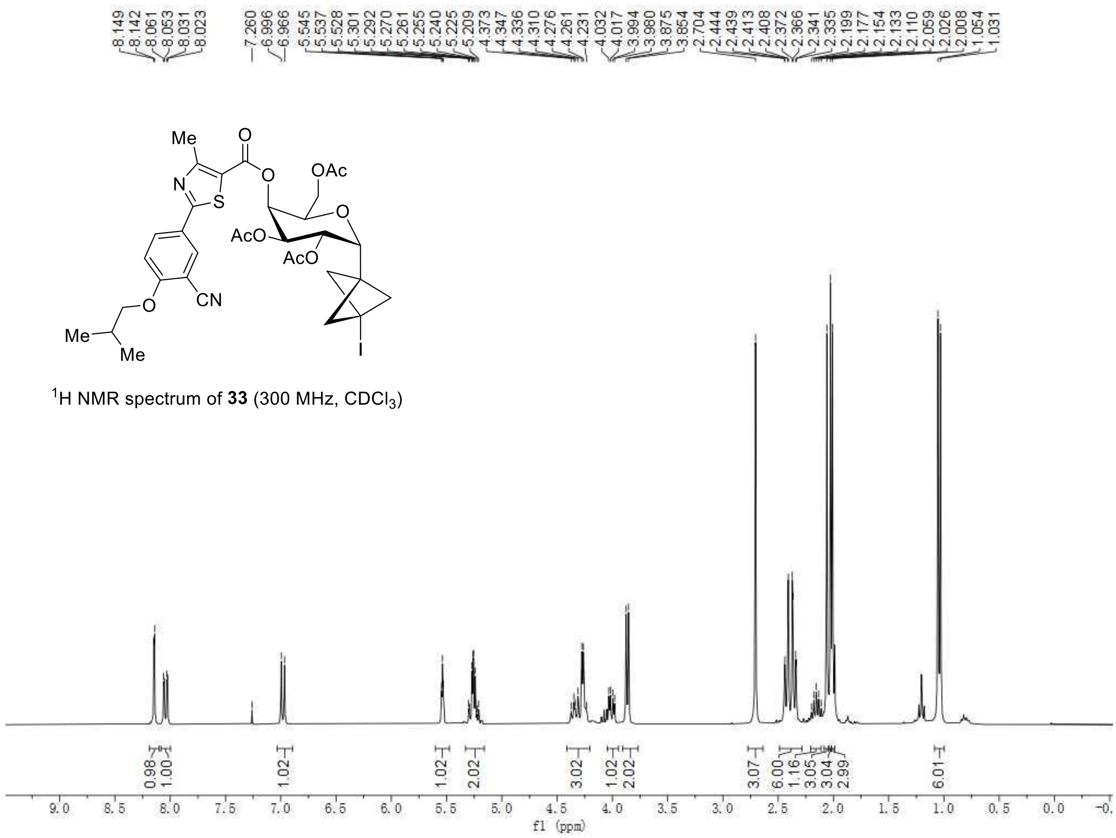

^{1}H NMR spectrum of **33** (300 MHz, $CDCl_3$)

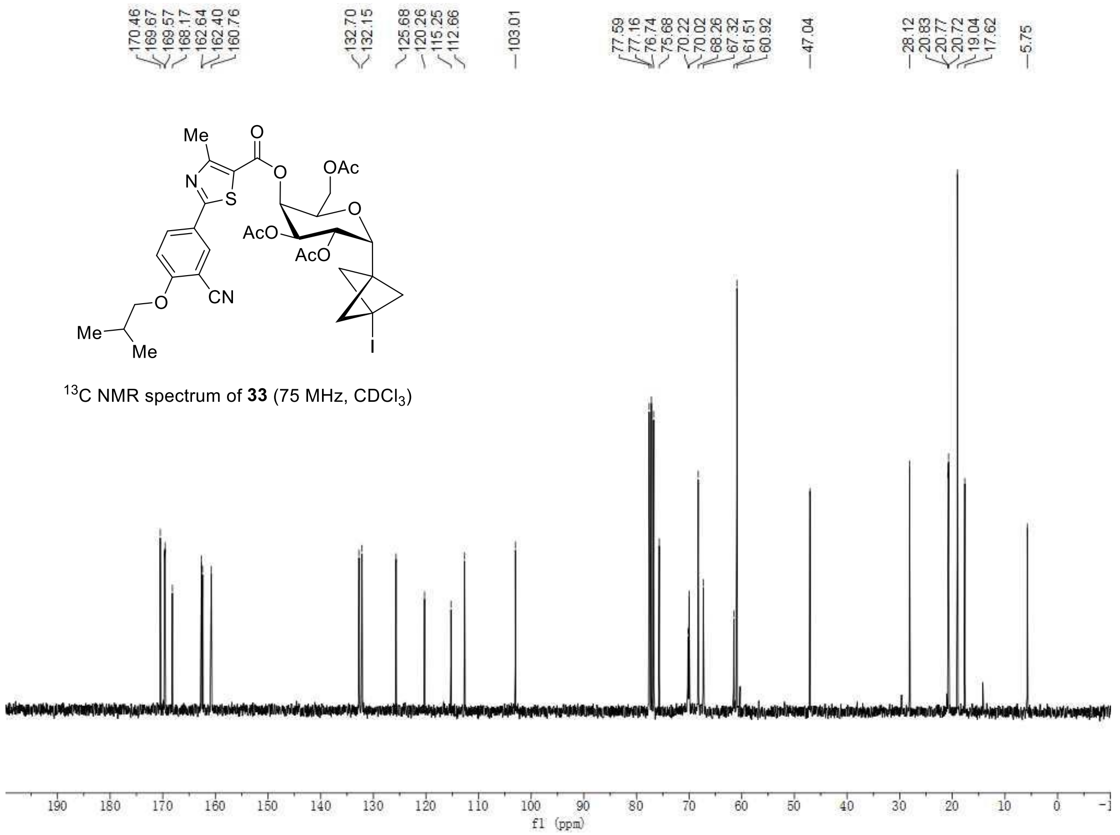

^{13}C NMR spectrum of **33** (75 MHz, $CDCl_3$)

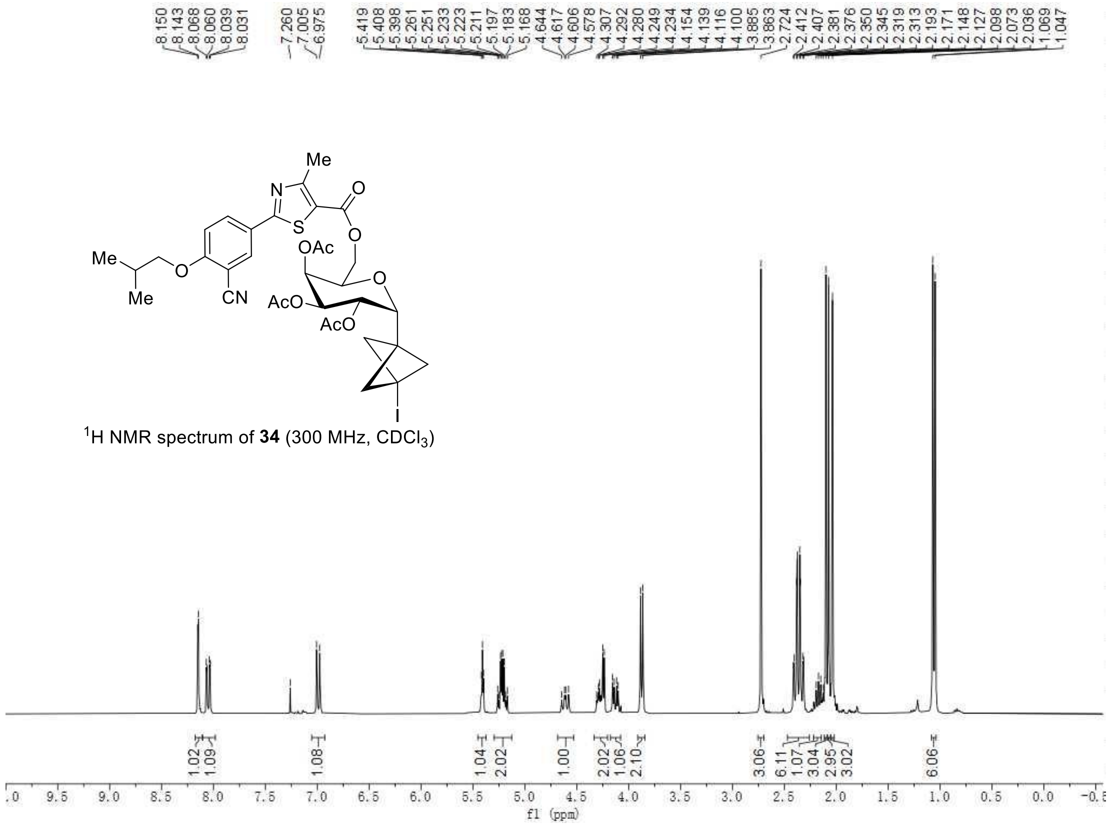

^{1}H NMR spectrum of **34** (300 MHz, $CDCl_3$)

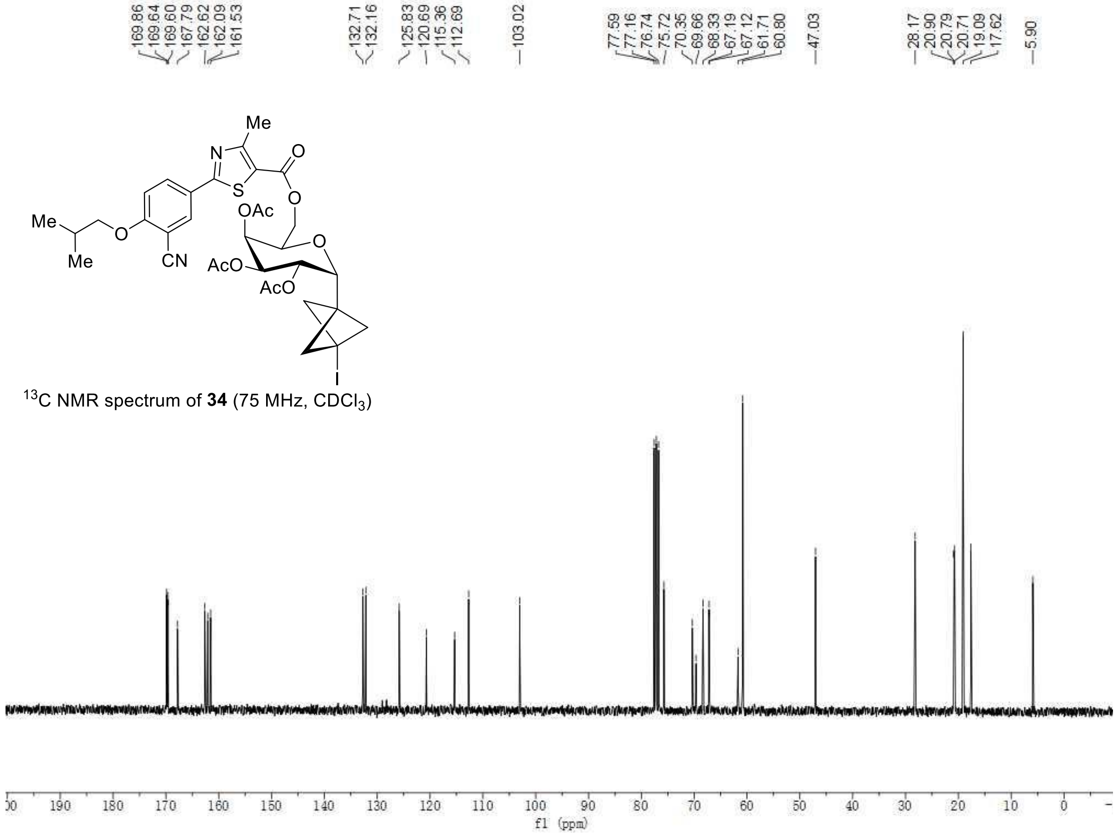

^{13}C NMR spectrum of **34** (75 MHz, $CDCl_3$)

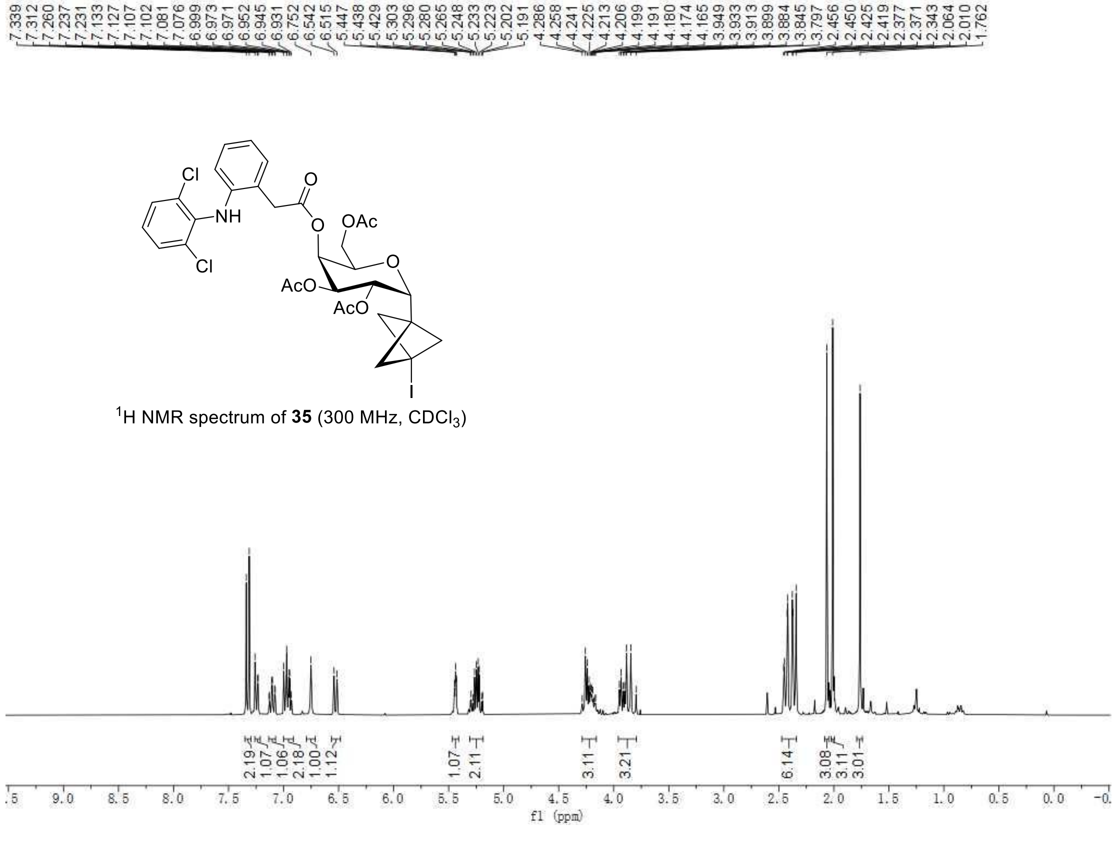

^{1}H NMR spectrum of **35** (300 MHz, $CDCl_3$)

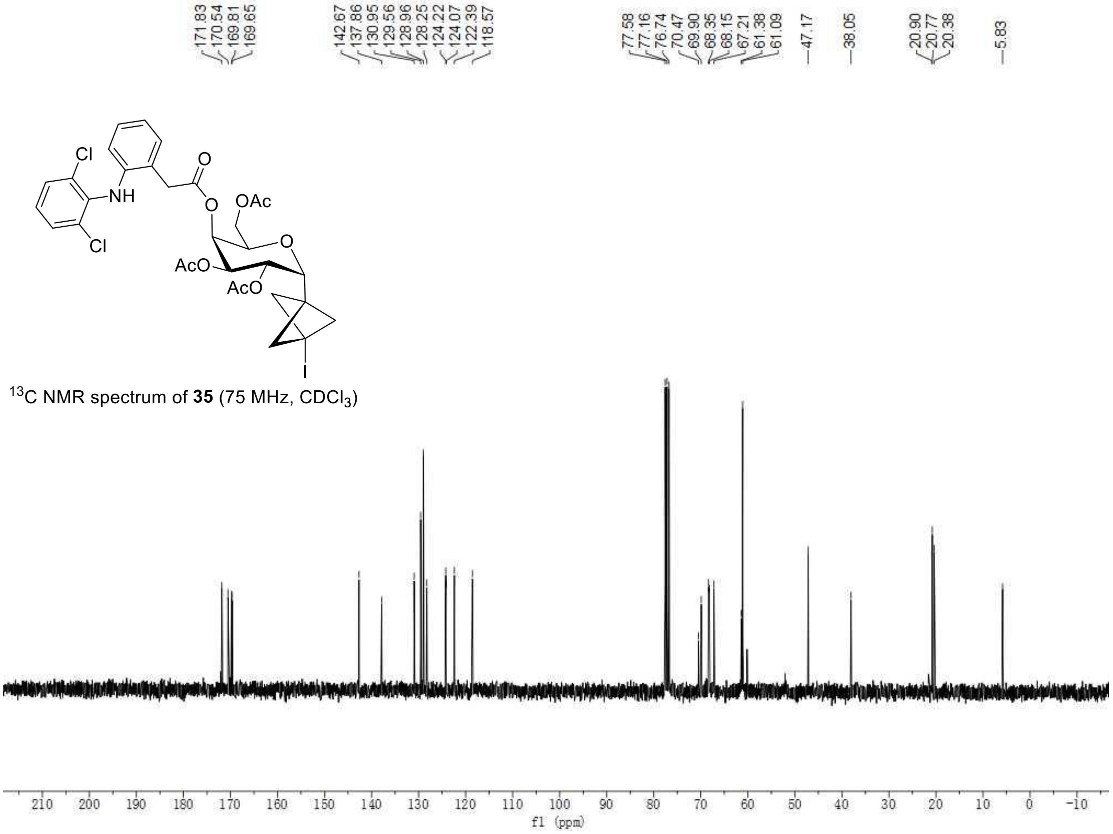

^{13}C NMR spectrum of **35** (75 MHz, $CDCl_3$)

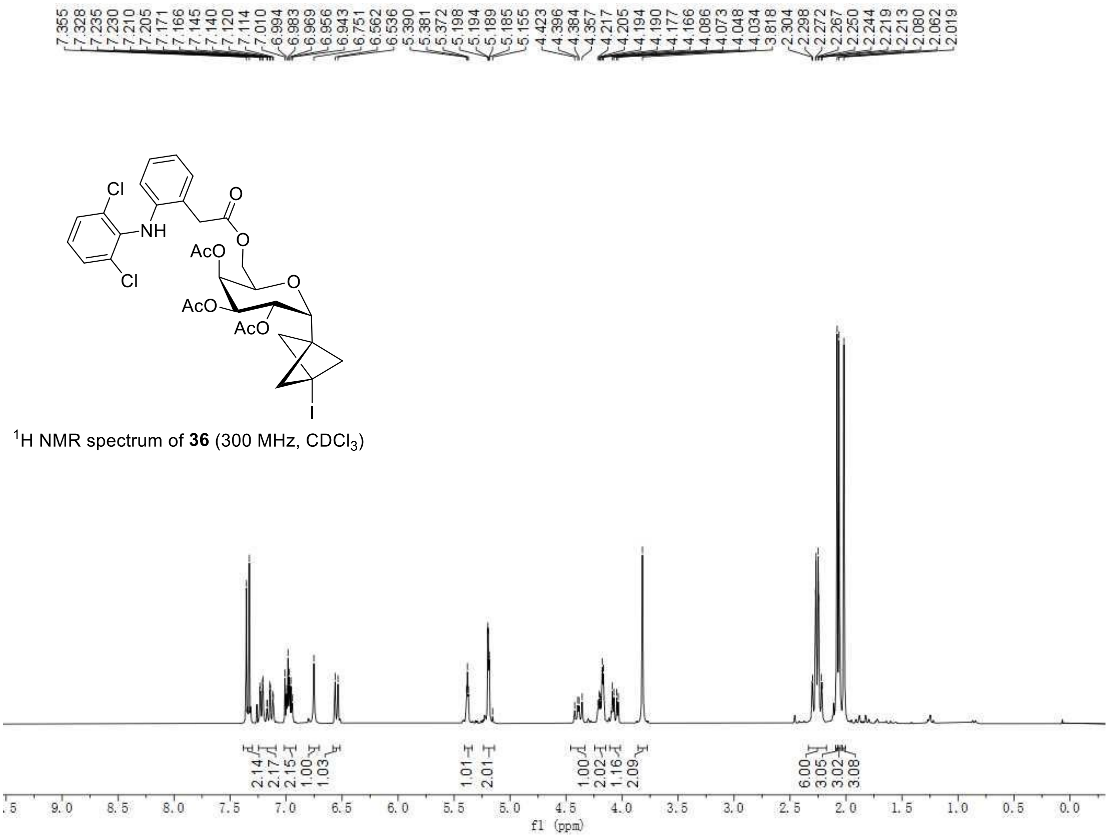

^{1}H NMR spectrum of **36** (300 MHz, $CDCl_3$)

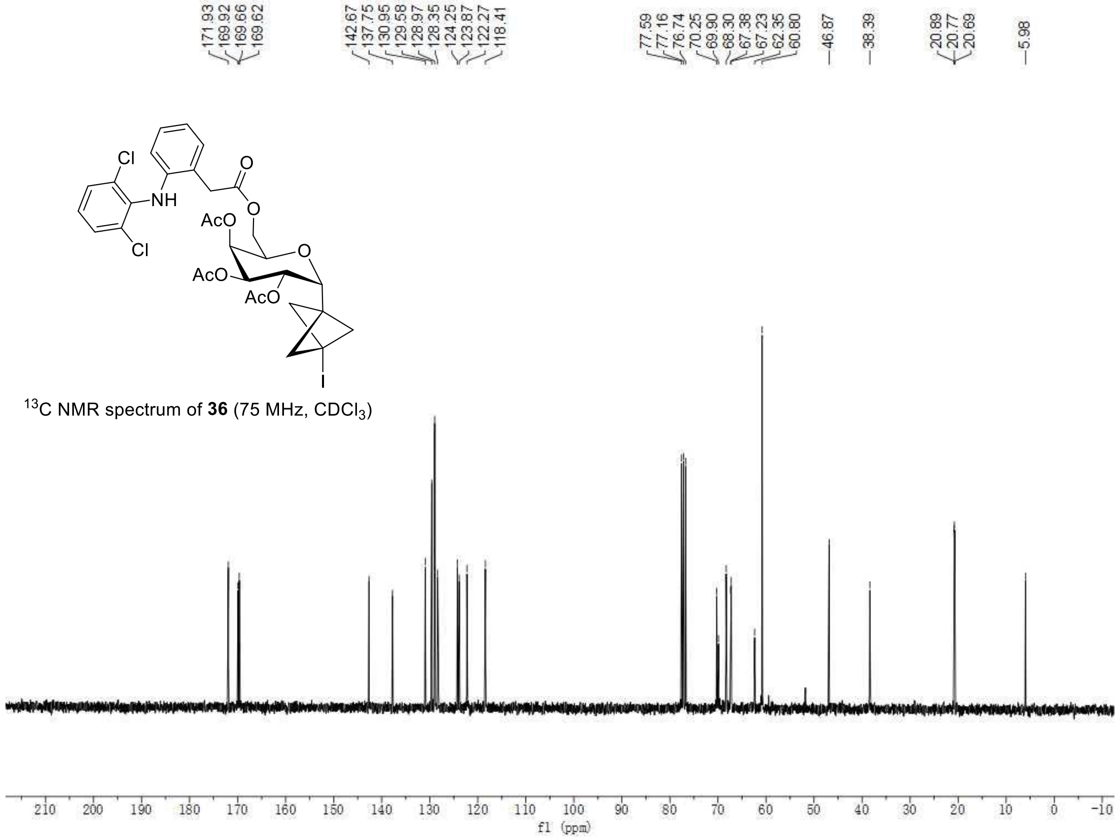

^{13}C NMR spectrum of **36** (75 MHz, $CDCl_3$)

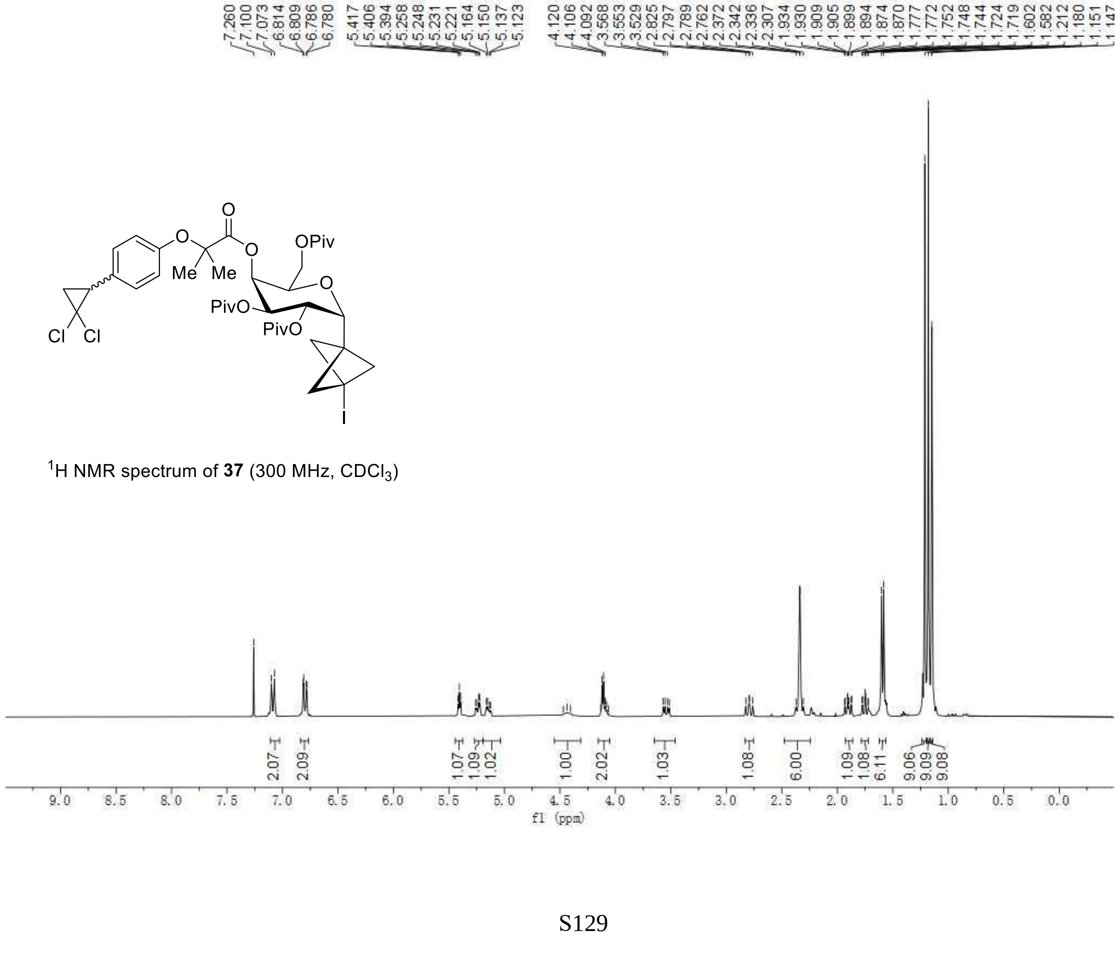

^{1}H NMR spectrum of **37** (300 MHz, $CDCl_3$)

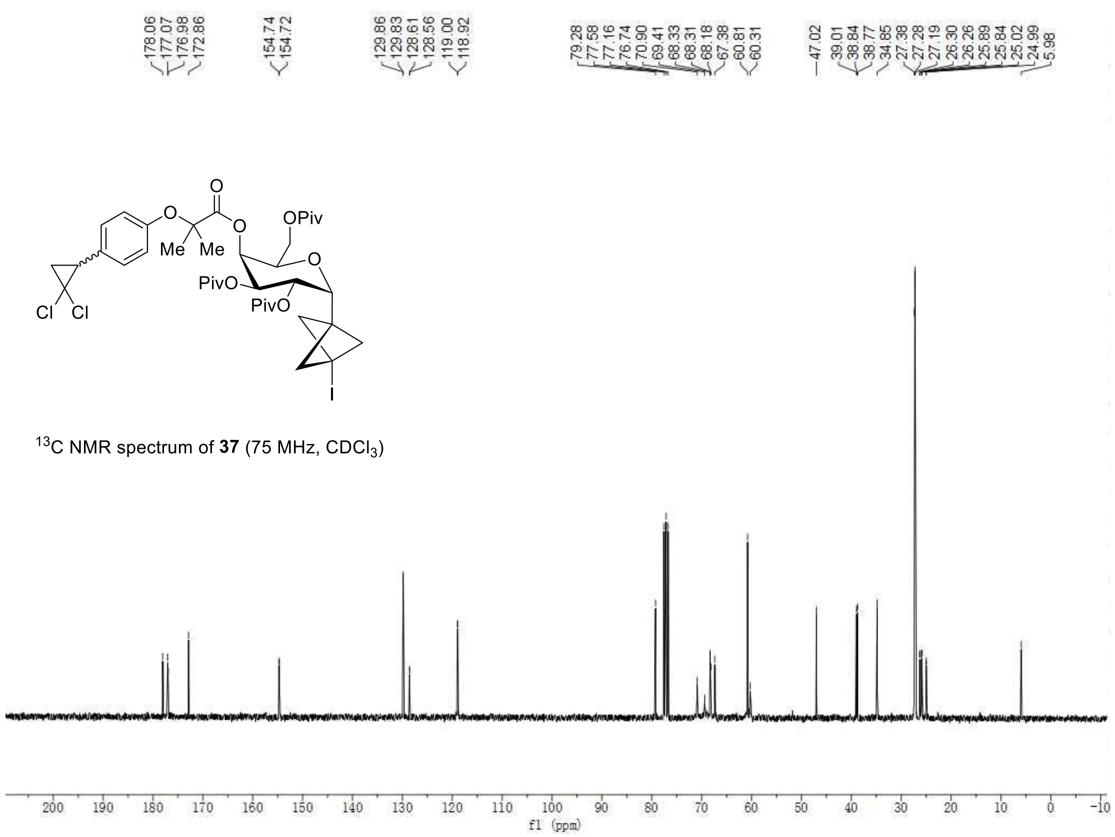

^{13}C NMR spectrum of **37** (75 MHz, $CDCl_3$)

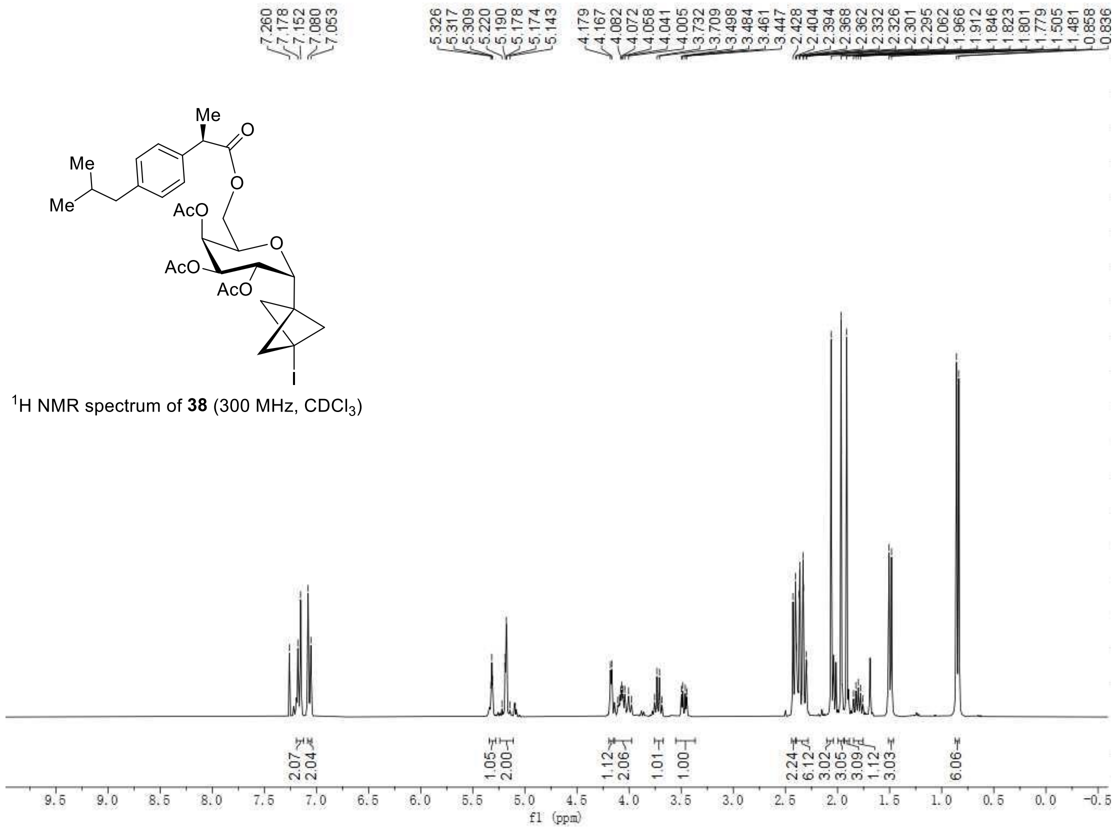

^{1}H NMR spectrum of **38** (300 MHz, $CDCl_3$)

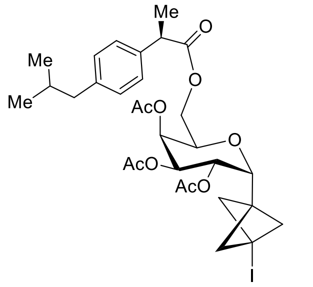

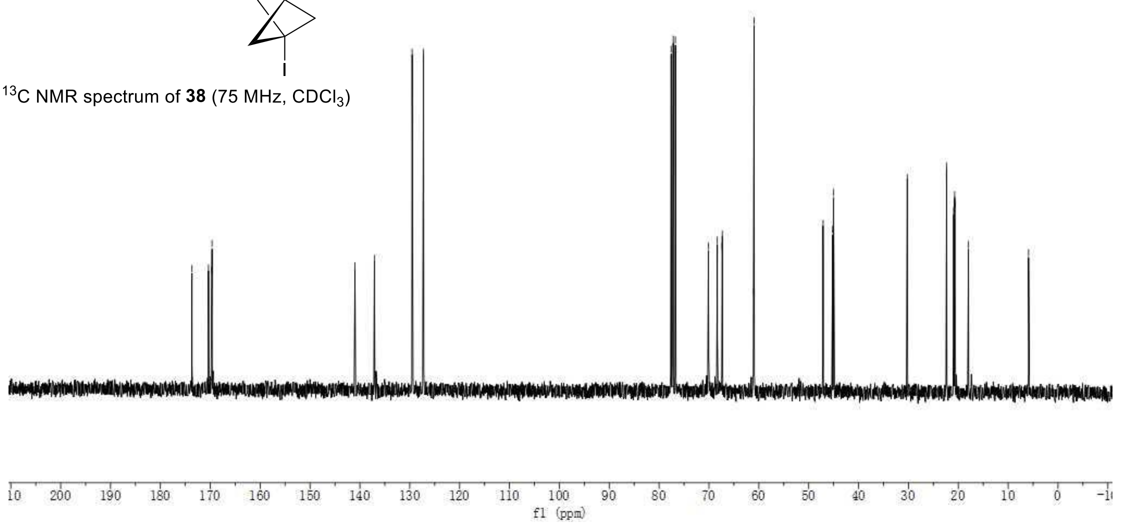

^{13}C NMR spectrum of **38** (75 MHz, $CDCl_3$)

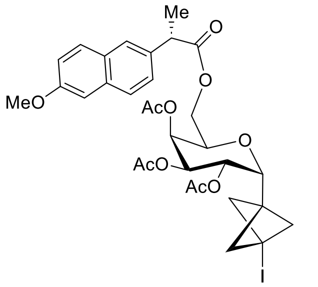

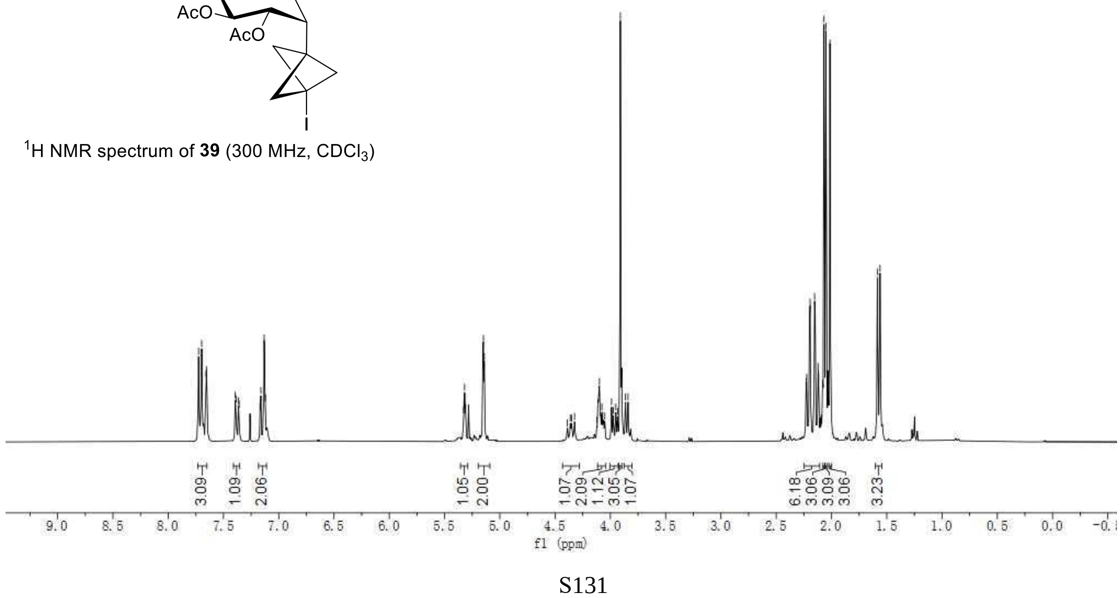

^{1}H NMR spectrum of **39** (300 MHz, $CDCl_3$)

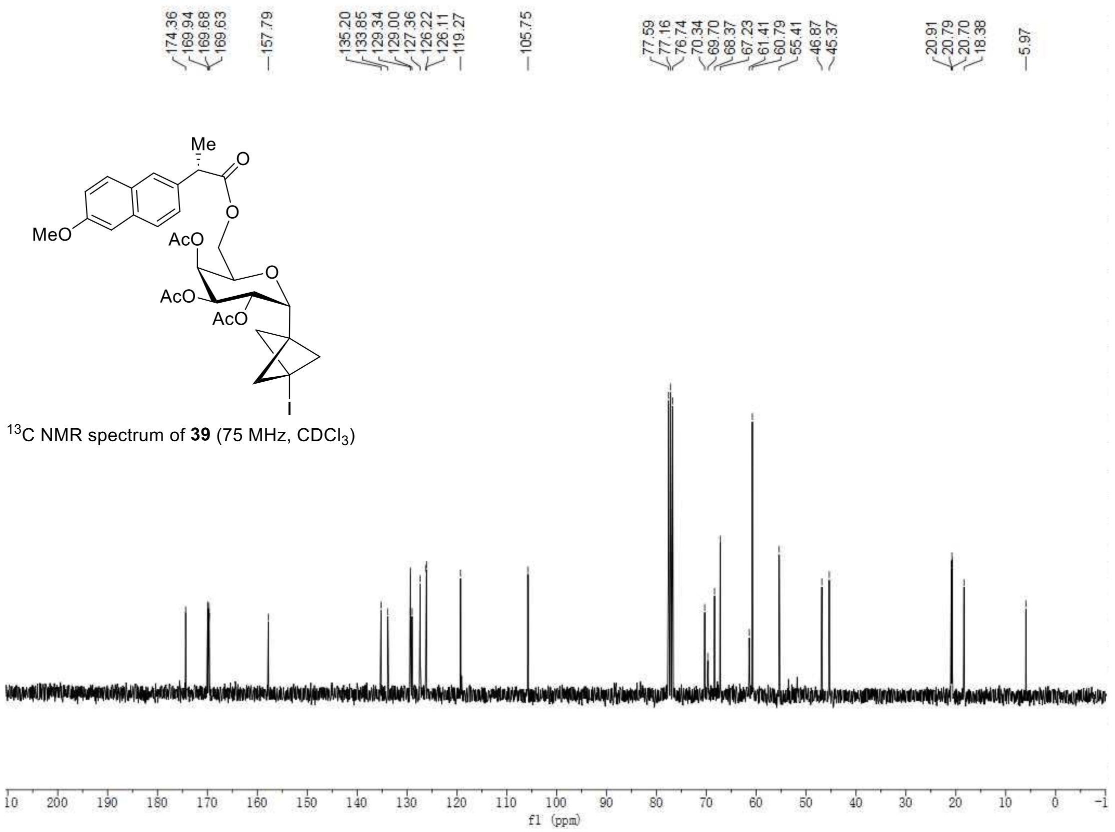

^{13}C NMR spectrum of **39** (75 MHz, $CDCl_3$)

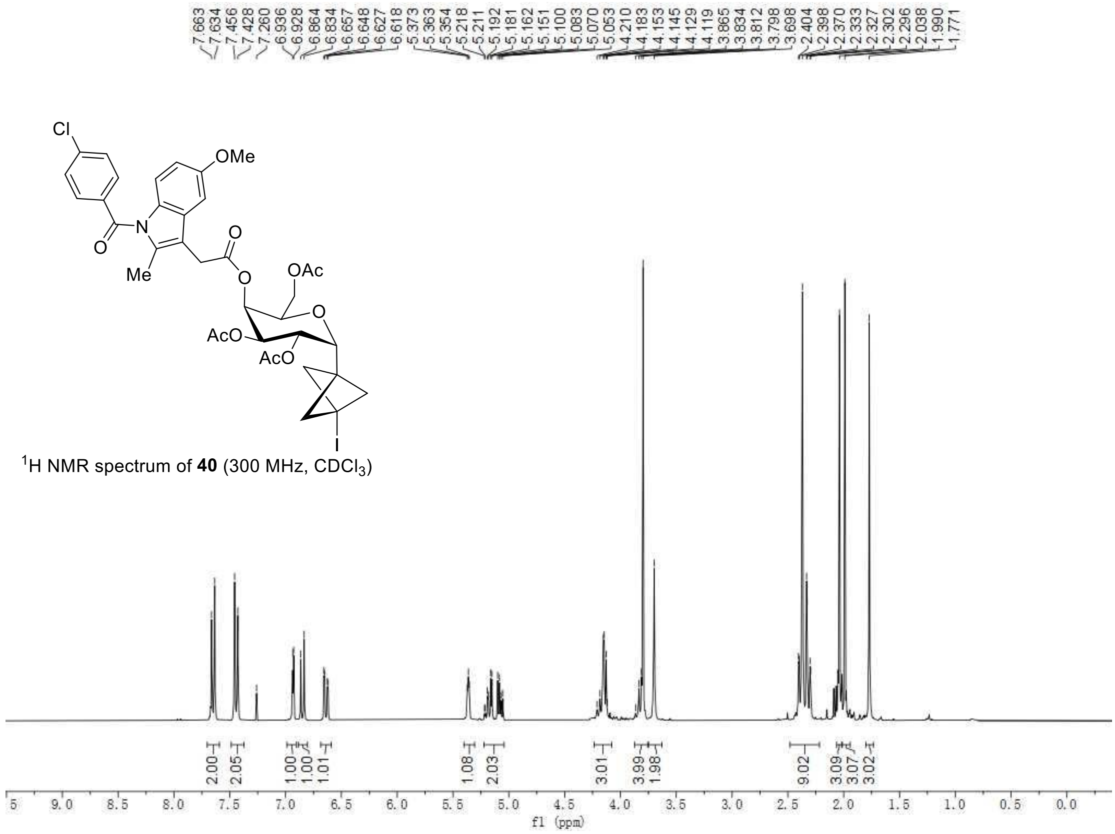

^{1}H NMR spectrum of **40** (300 MHz, $CDCl_3$)

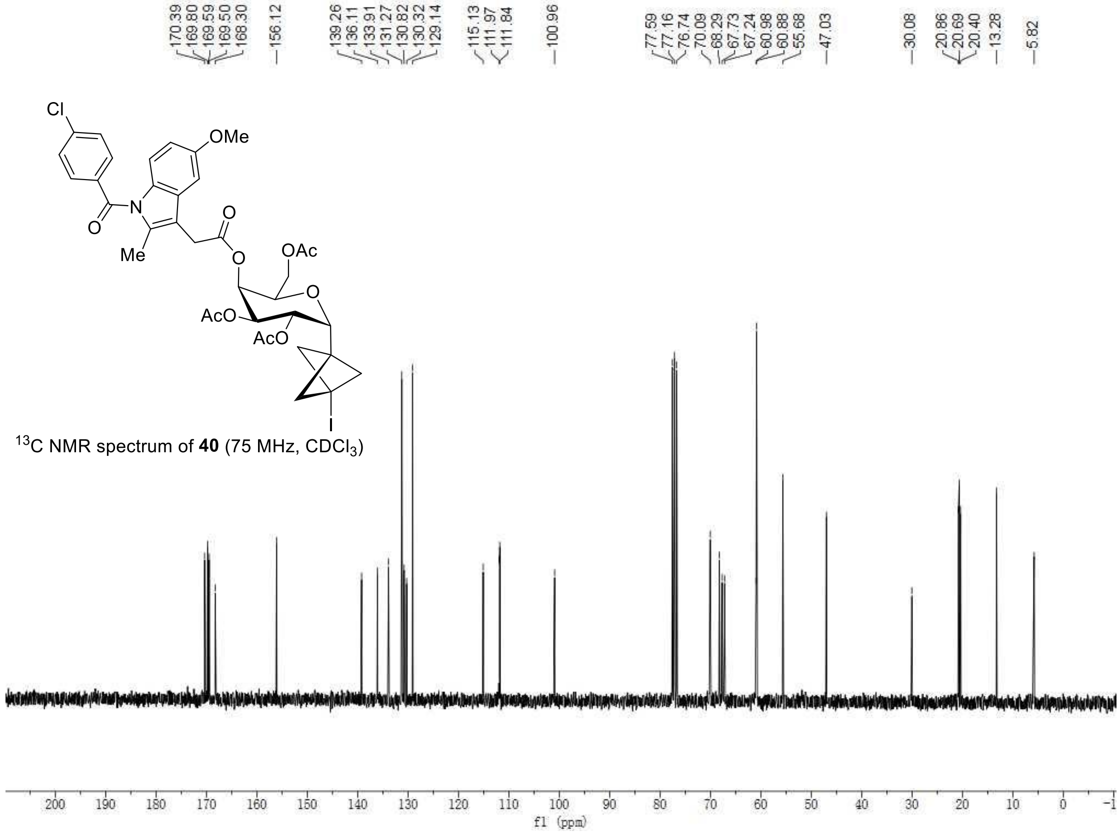

^{13}C NMR spectrum of **40** (75 MHz, $CDCl_3$)

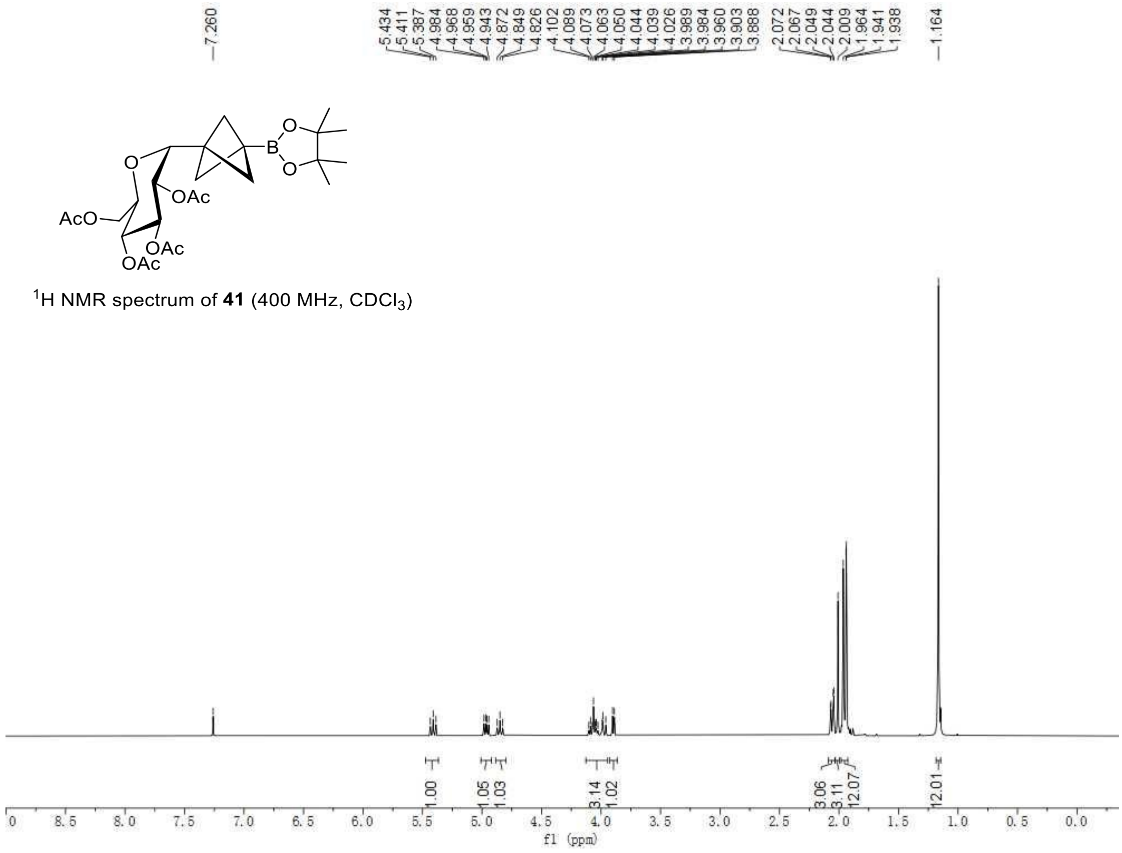

^{1}H NMR spectrum of **41** (400 MHz, $CDCl_3$)

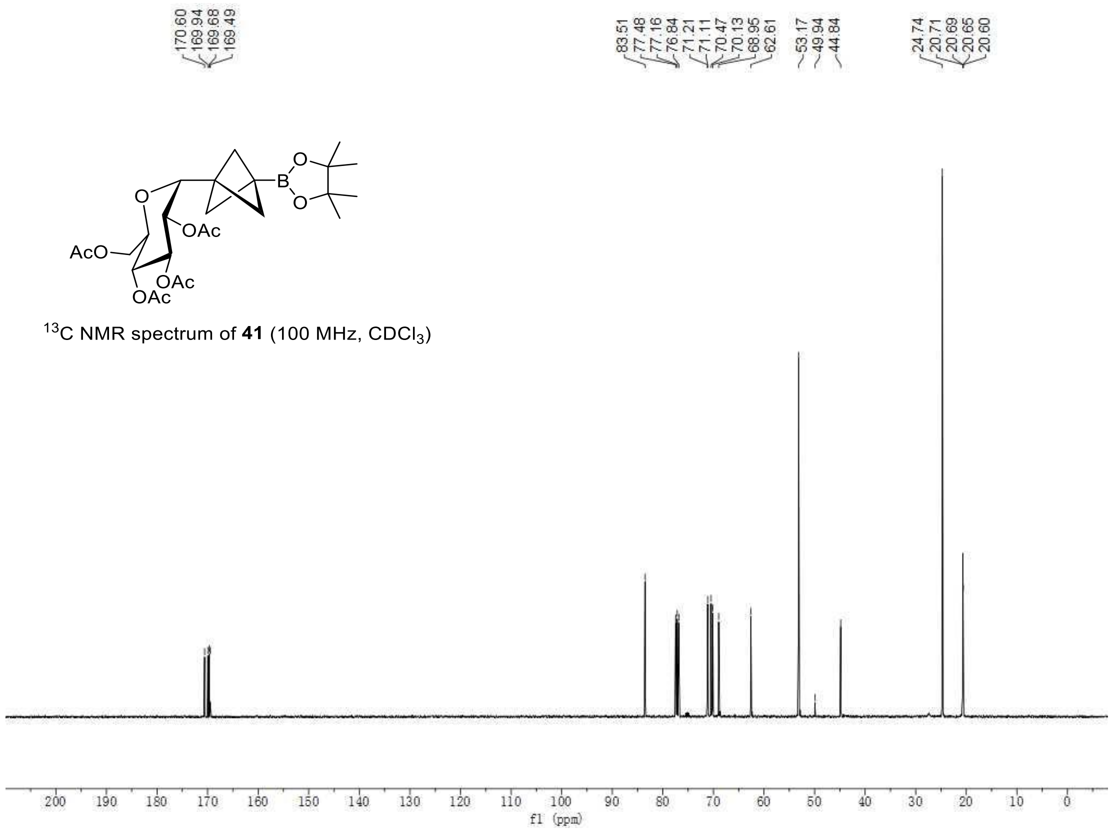

^{13}C NMR spectrum of **41** (100 MHz, $CDCl_3$)

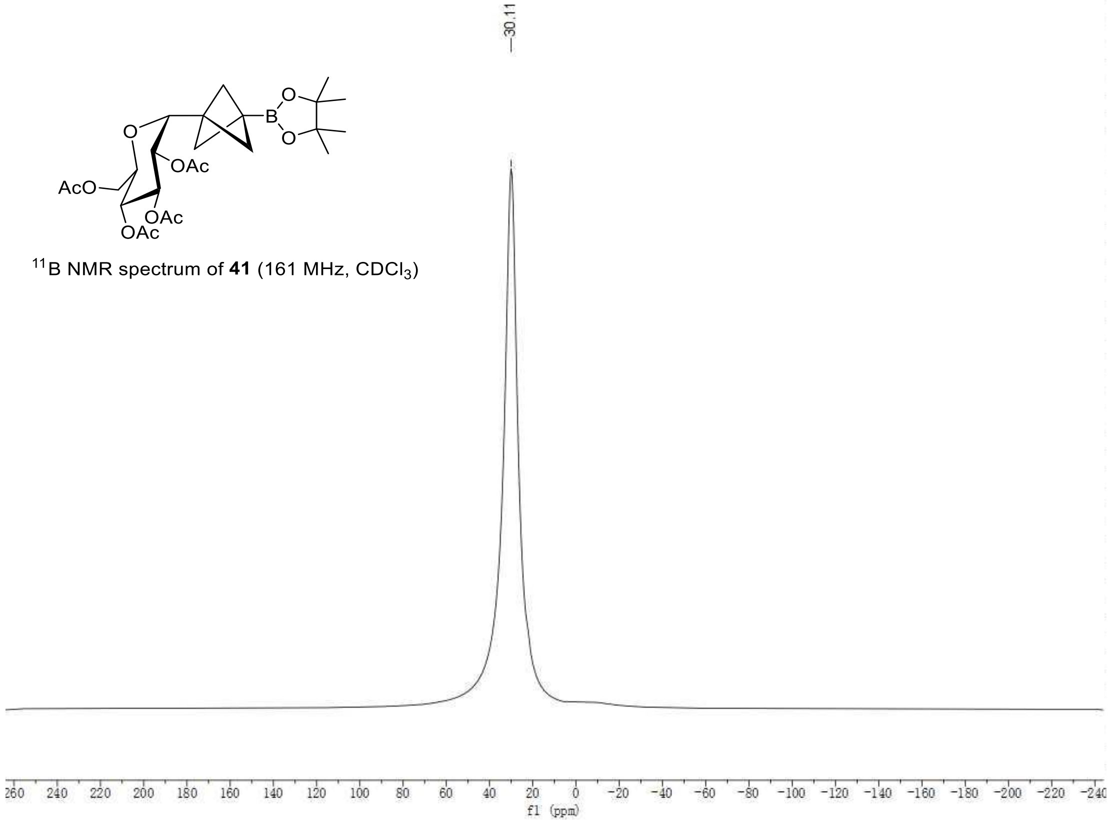

^{11}B NMR spectrum of **41** (161 MHz, $CDCl_3$)

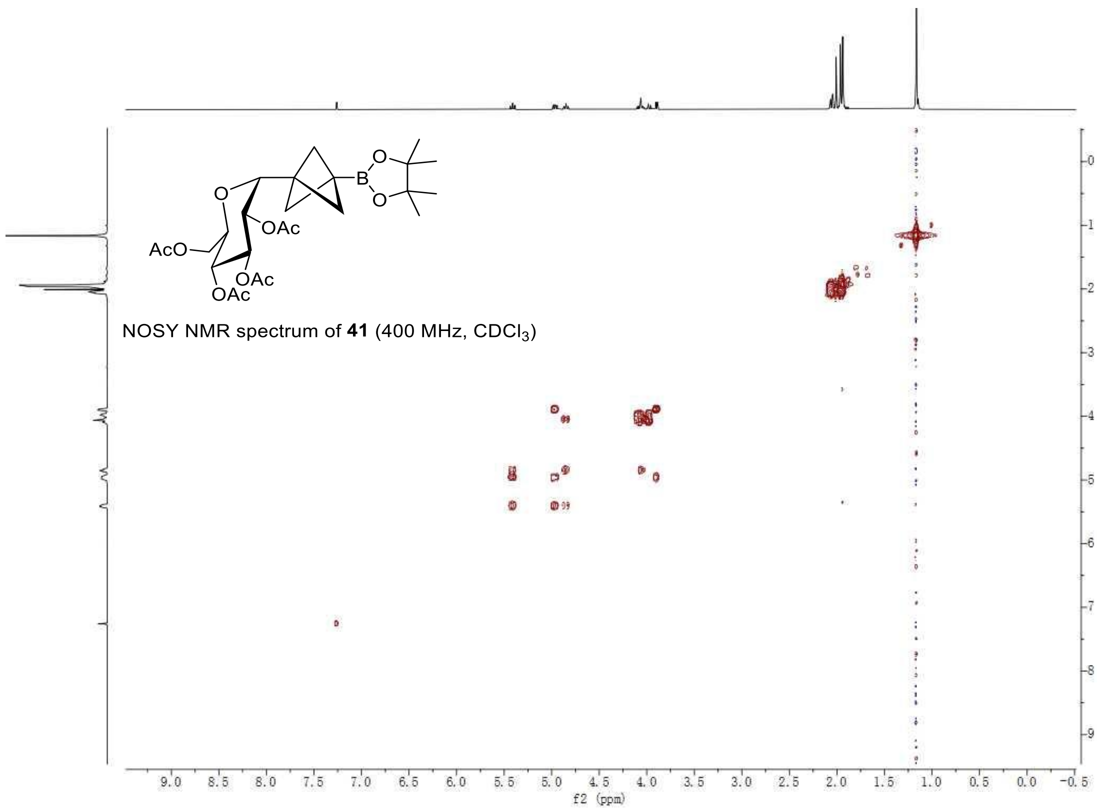
AcO
OAc
OAc
OAc
NOSY NMR spectrum of 41 (400 MHz, CDCl3)
f2 (ppm)

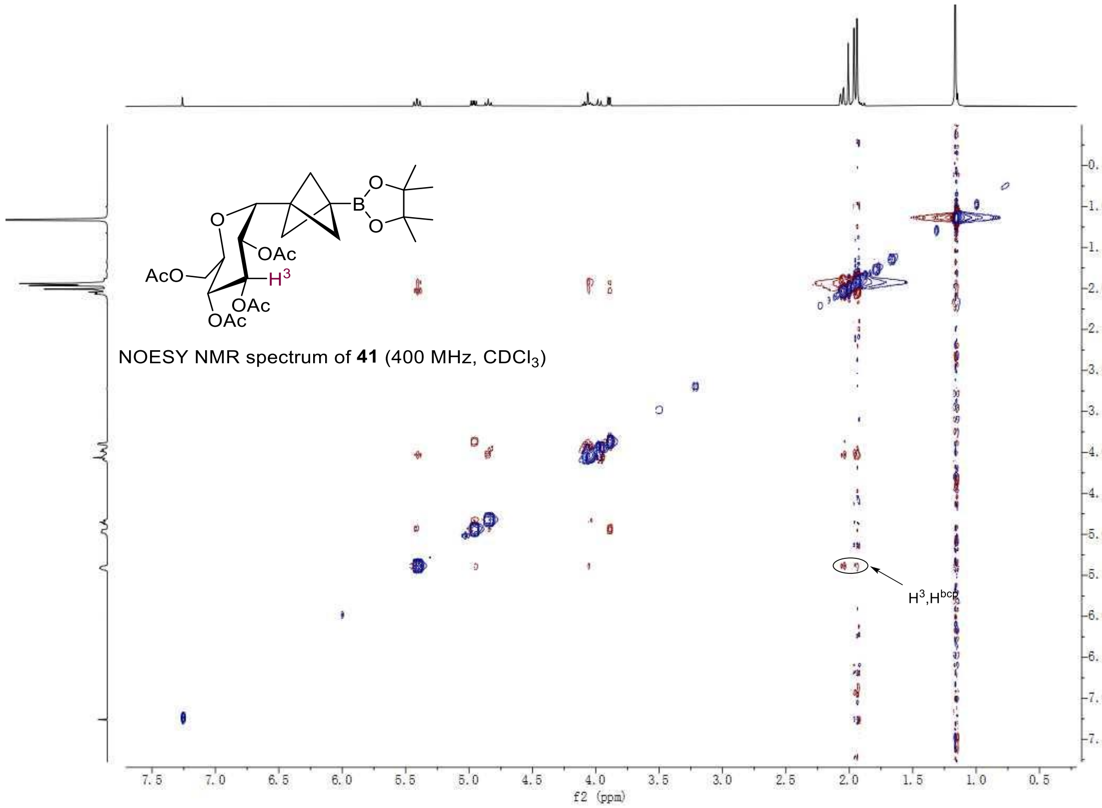
AcO
OAc
OAc
OAc
H3
NOESY NMR spectrum of 41 (400 MHz, CDCl3)
H3,Hbcp
f2 (ppm)

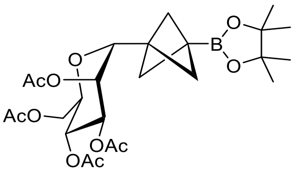

^{1}H NMR spectrum of **42** (400 MHz, $CDCl_3$)

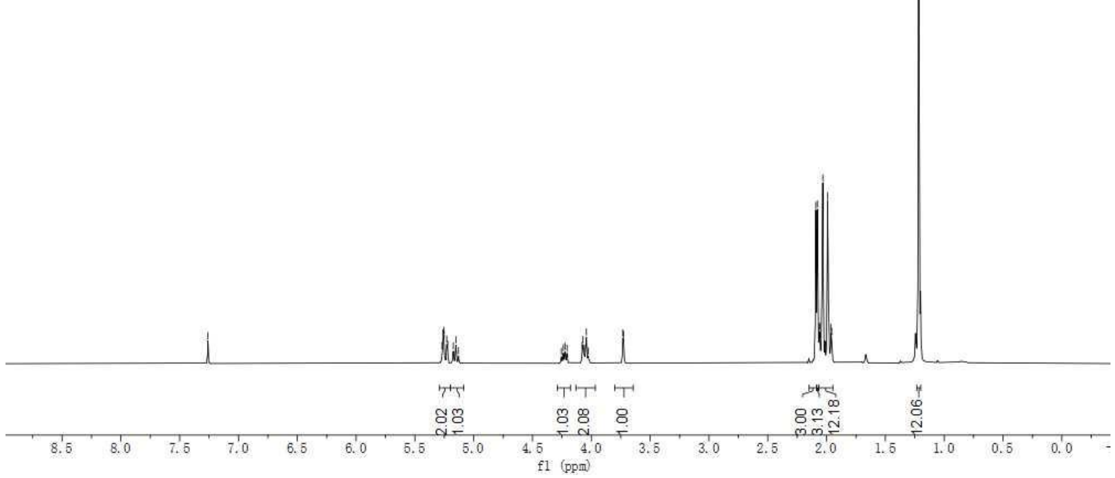

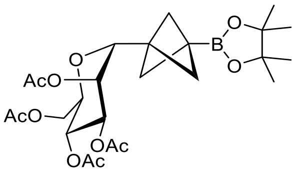

^{13}C NMR spectrum of **42** (100 MHz, $CDCl_3$)

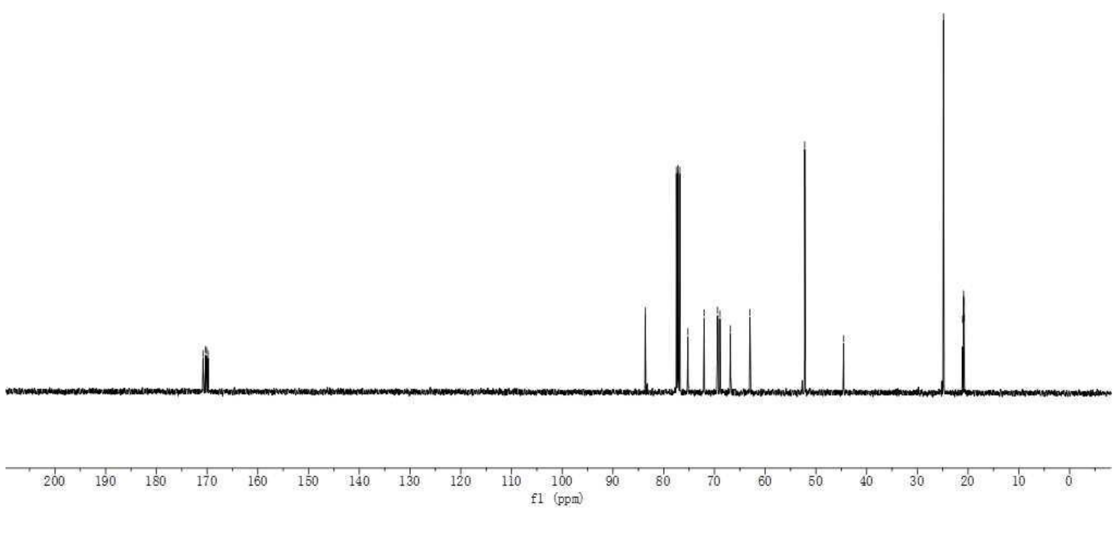

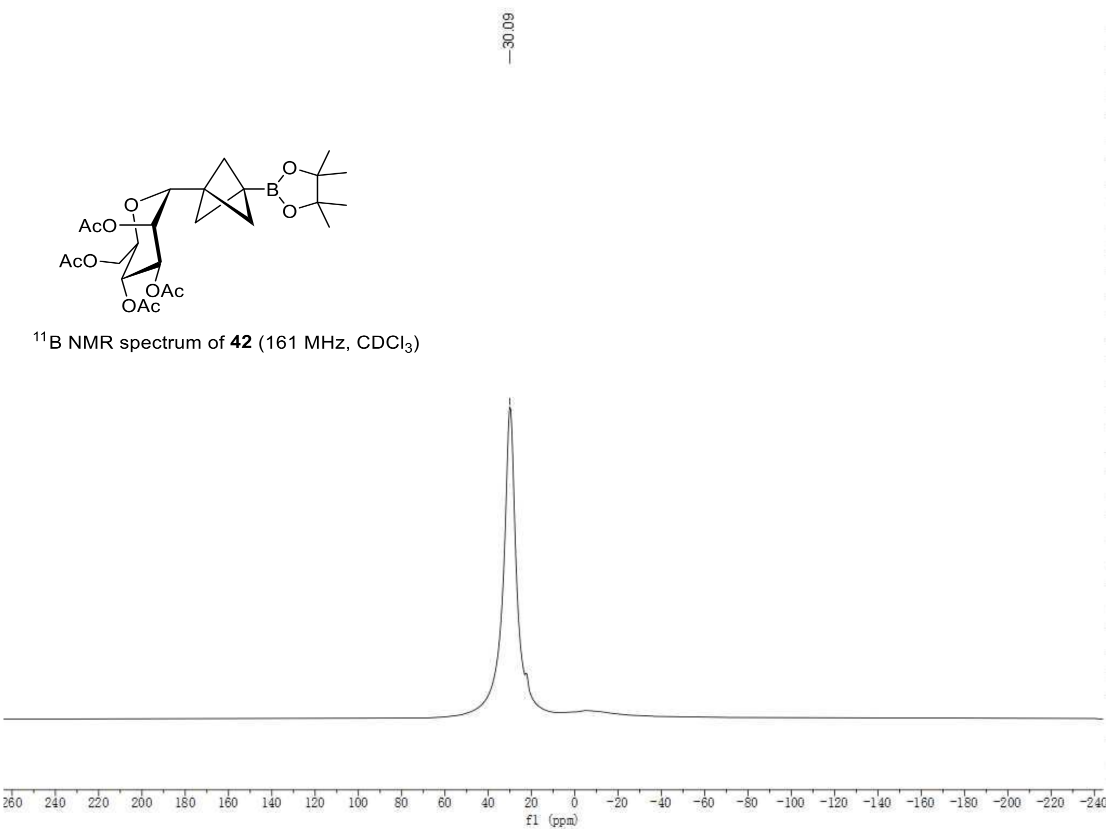

^{11}B NMR spectrum of **42** (161 MHz, $CDCl_3$)

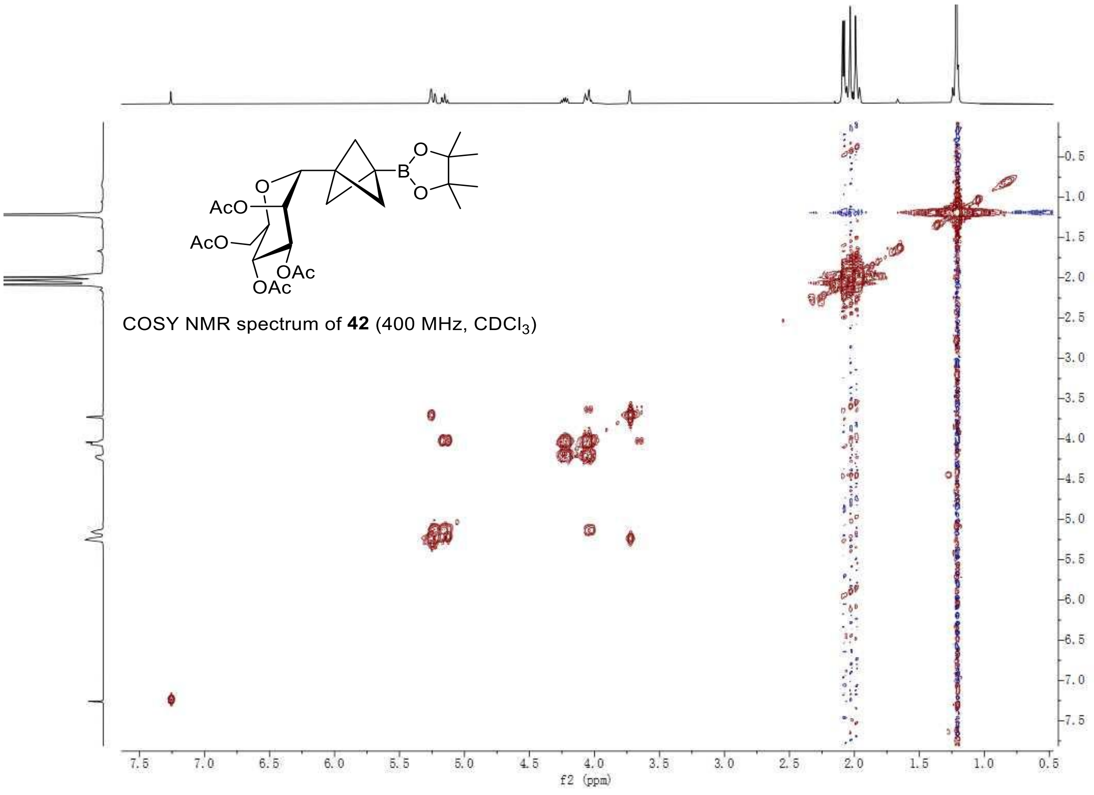

COSY NMR spectrum of **42** (400 MHz, $CDCl_3$)

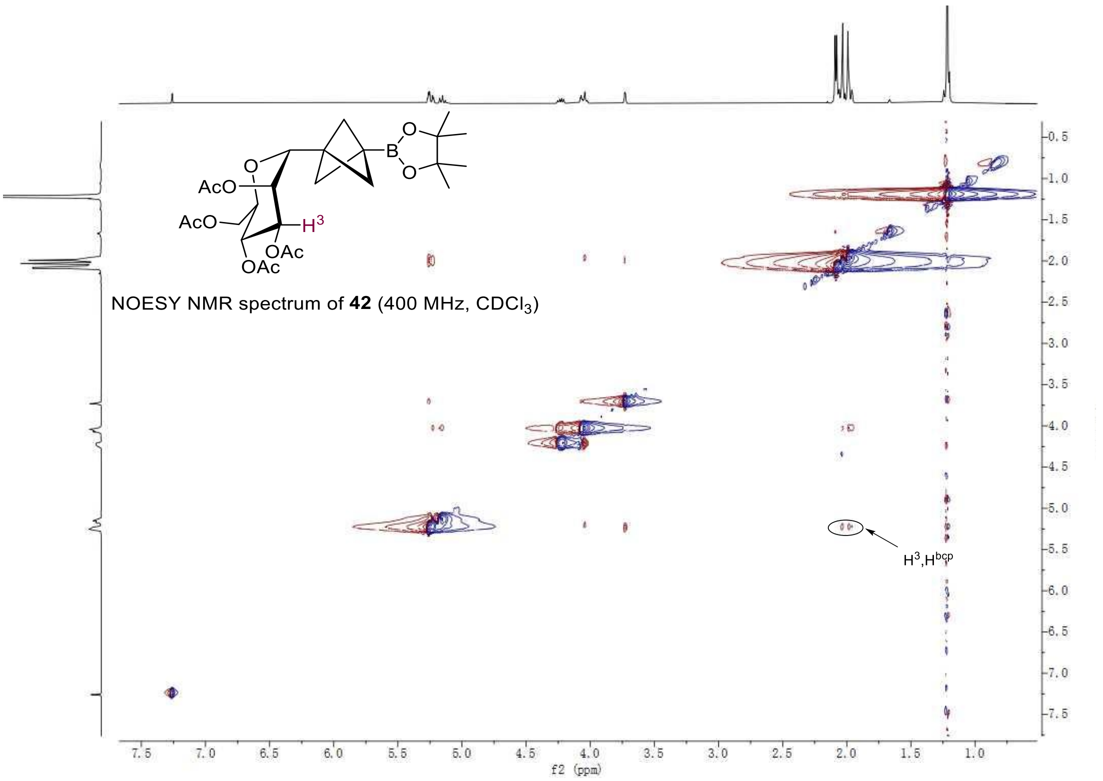

NOESY NMR spectrum of **42** (400 MHz, $CDCl_3$)

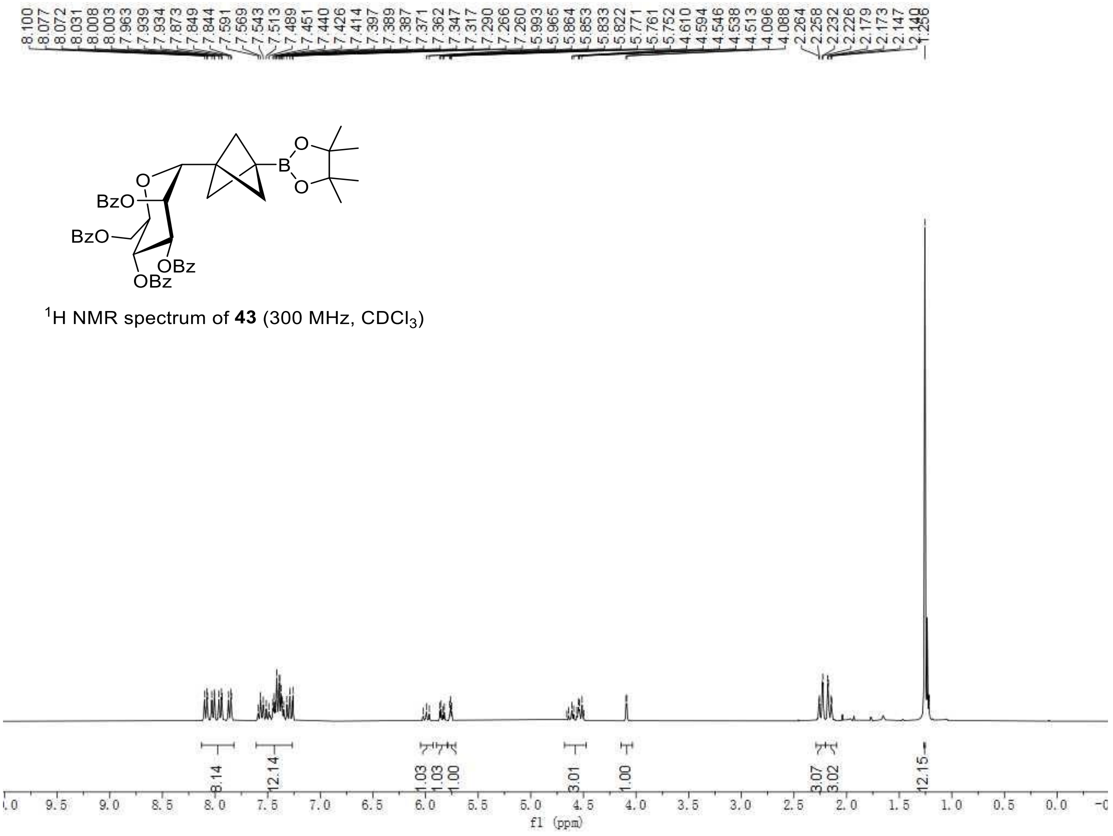

^{1}H NMR spectrum of **43** (300 MHz, $CDCl_3$)

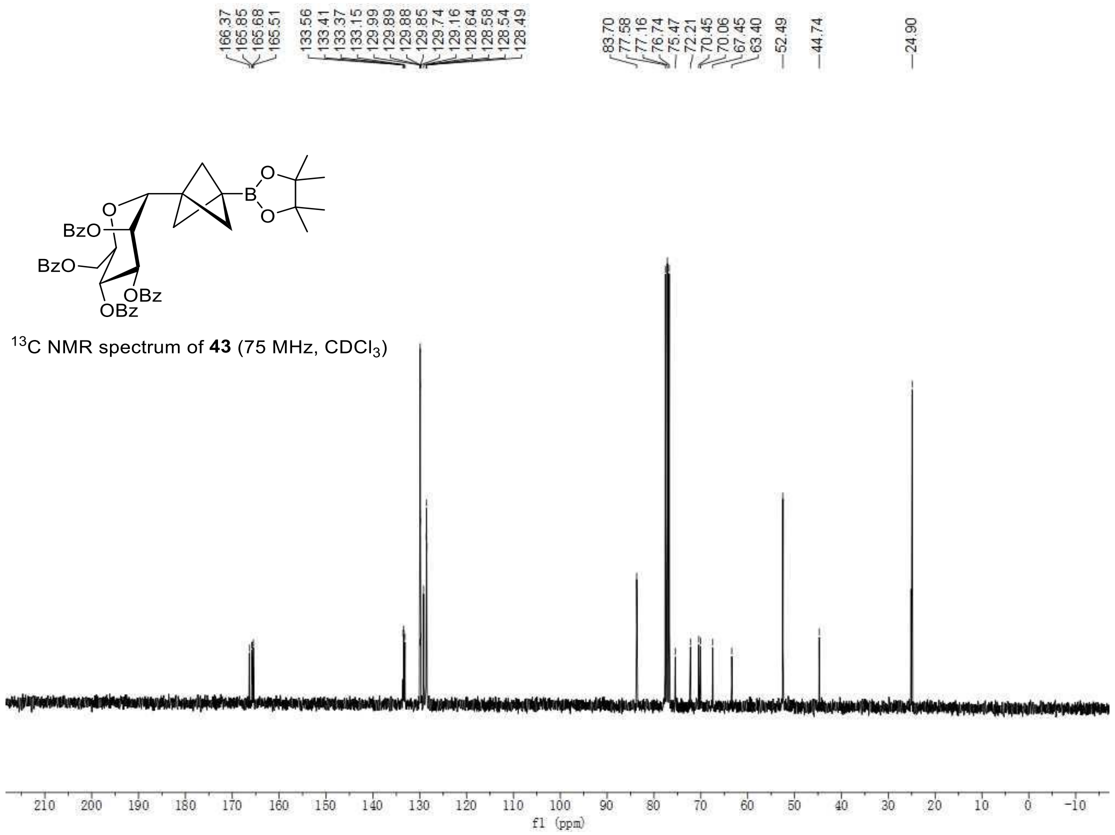

^{13}C NMR spectrum of **43** (75 MHz, $CDCl_3$)

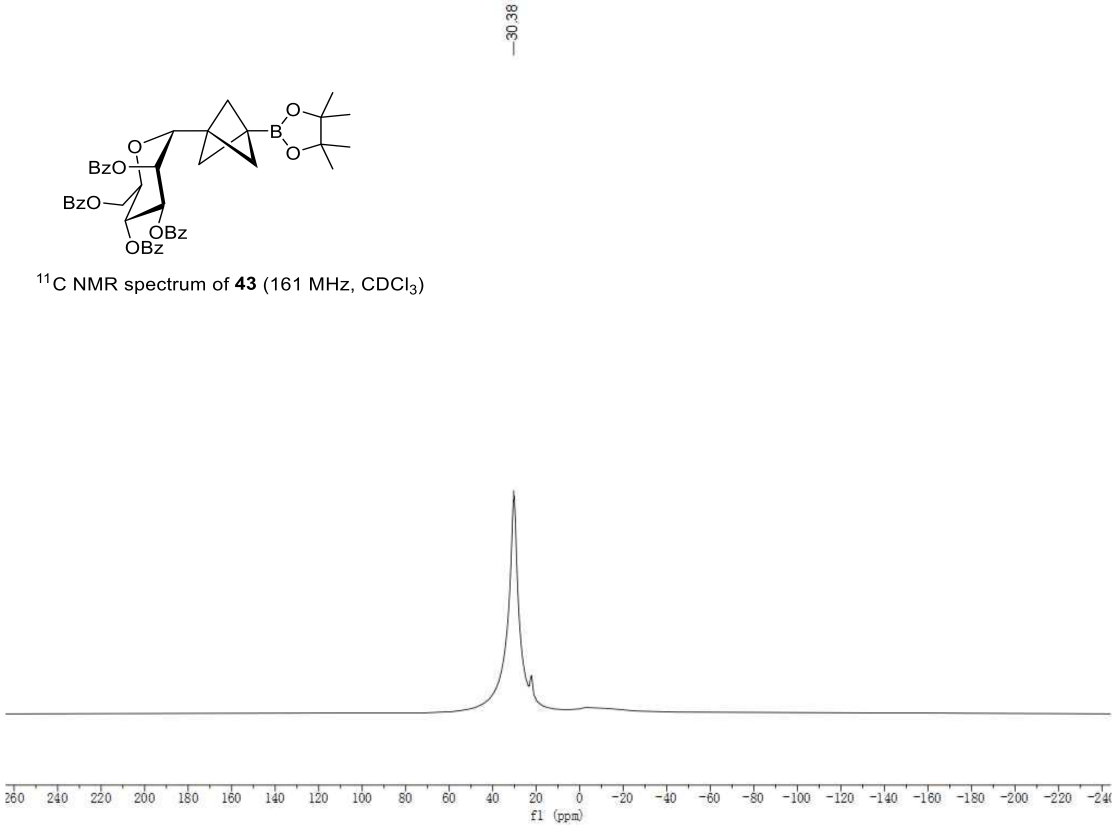

^{11}C NMR spectrum of **43** (161 MHz, $CDCl_3$)

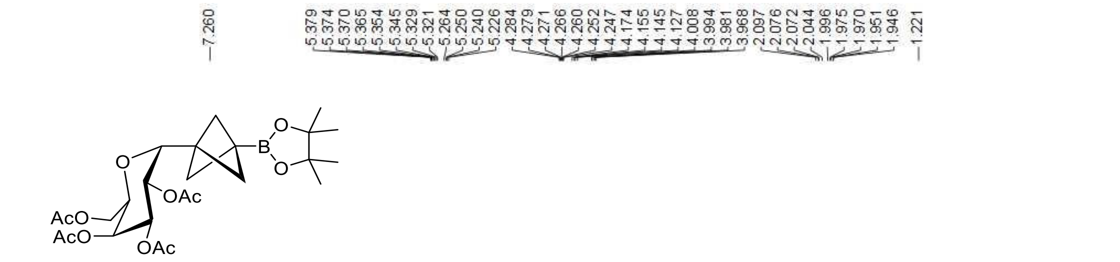

^{1}H NMR spectrum of **44** (400 MHz, $CDCl_3$)

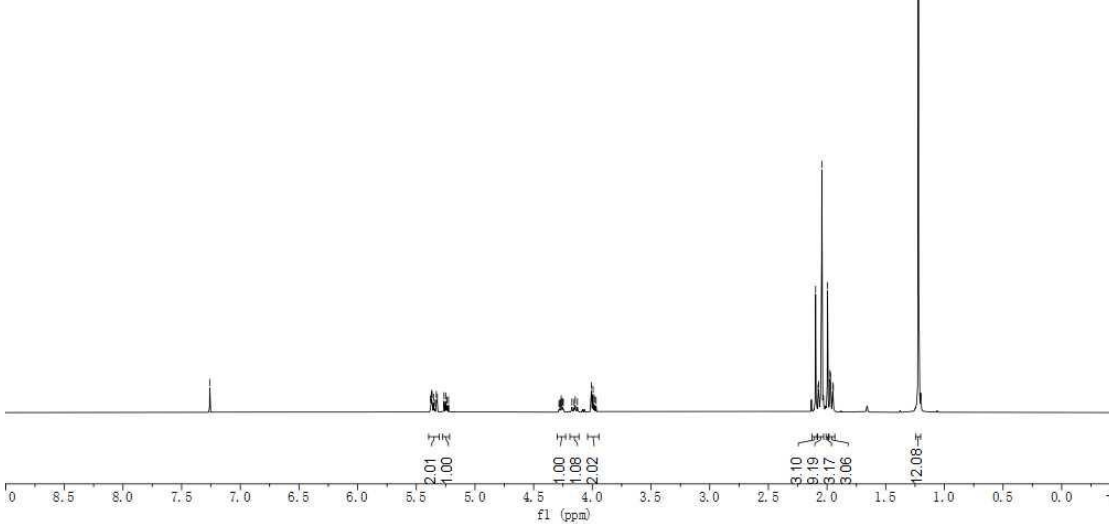

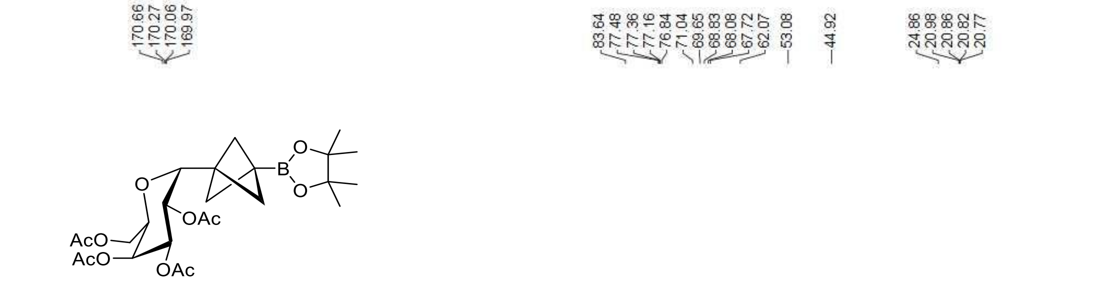

^{13}C NMR spectrum of **44** (100 MHz, $CDCl_3$)

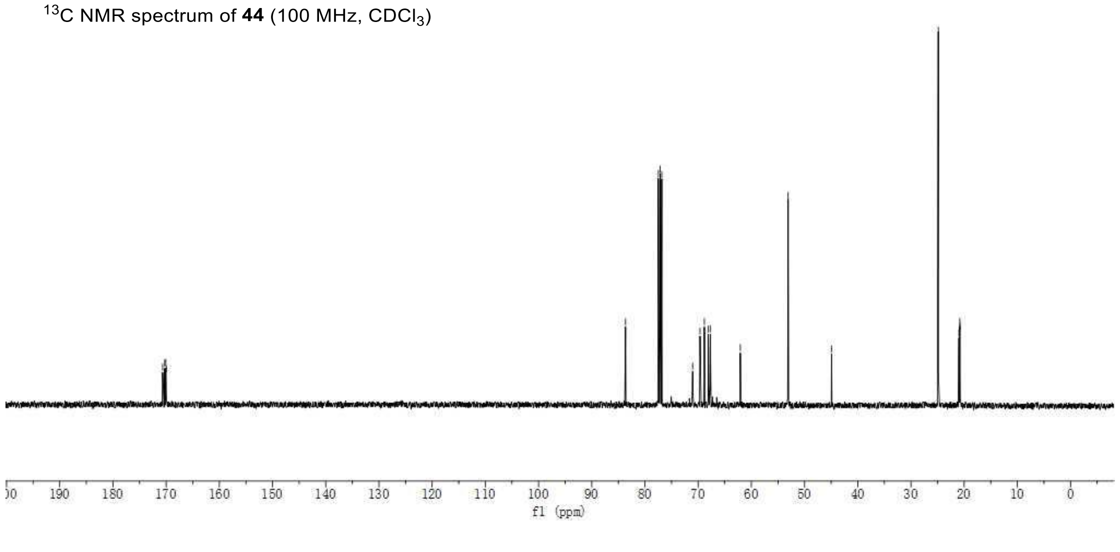

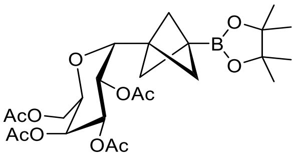

^{11}B NMR spectrum of **44** (161 MHz, $CDCl_3$)

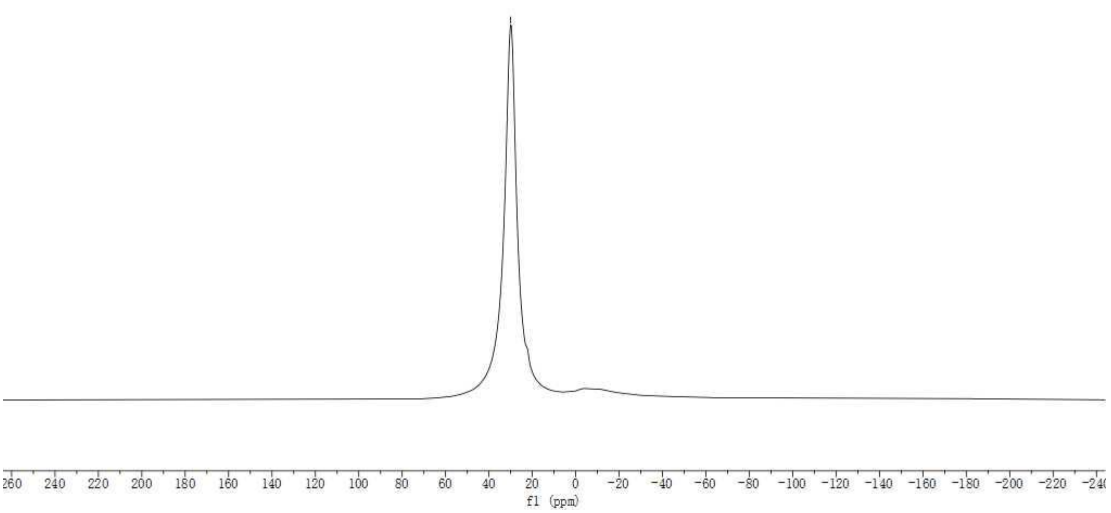

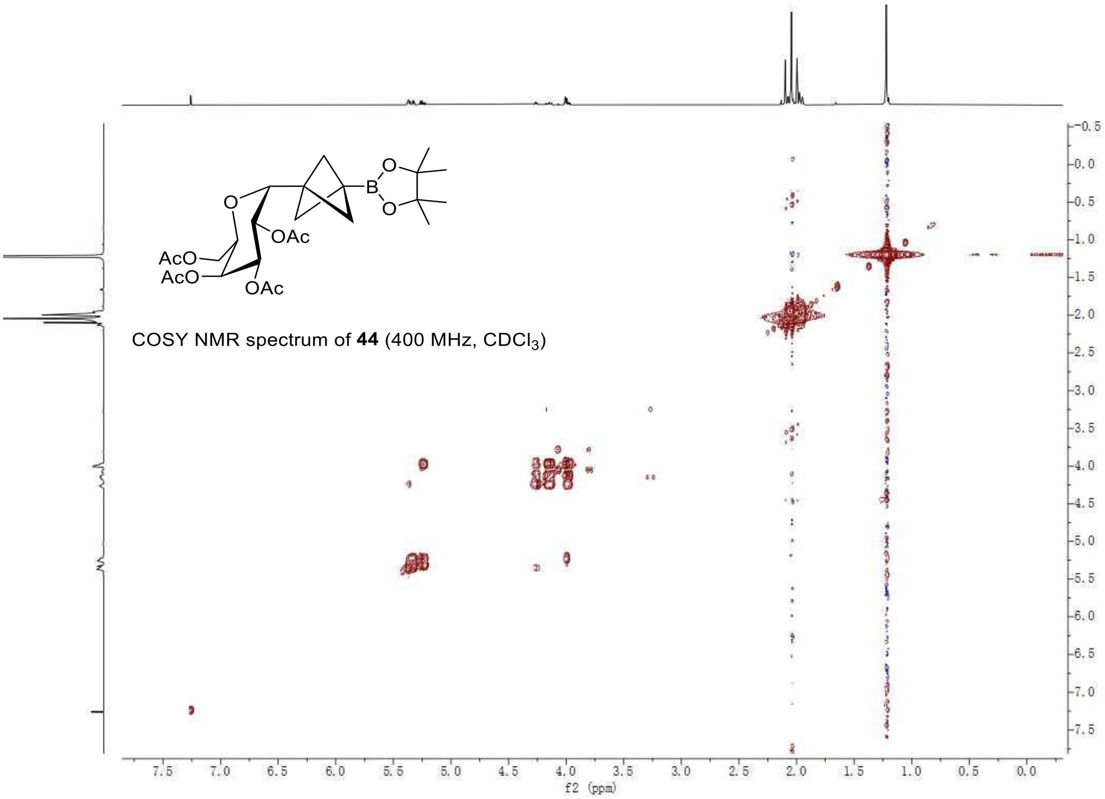

COSY NMR spectrum of **44** (400 MHz, $CDCl_3$)

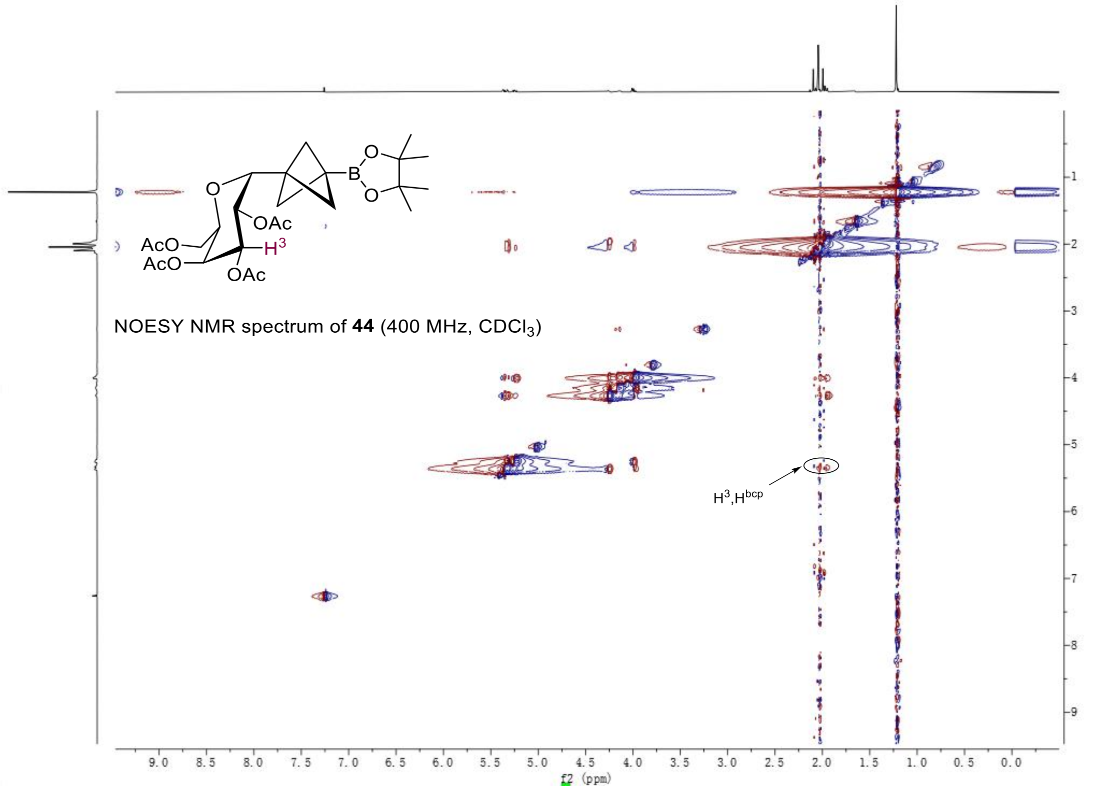

NOESY NMR spectrum of **44** (400 MHz, $CDCl_3$)

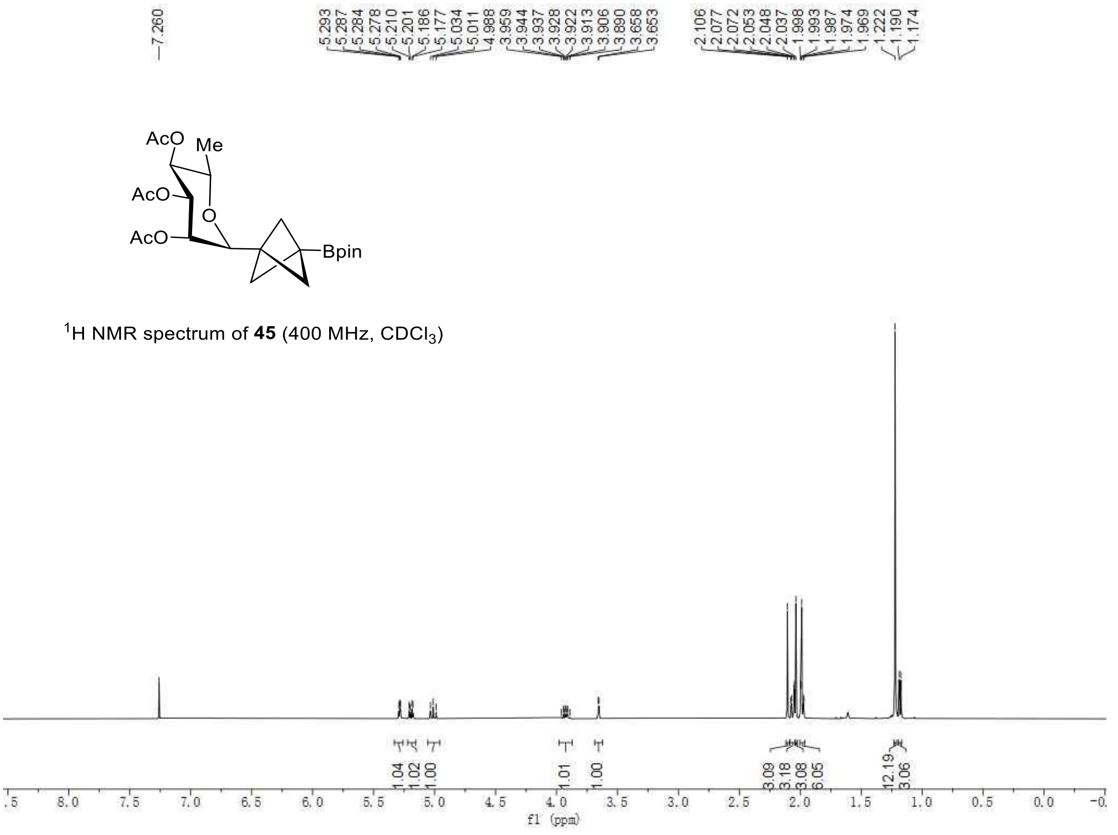

¹H NMR spectrum of **45** (400 MHz, $CDCl_3$)

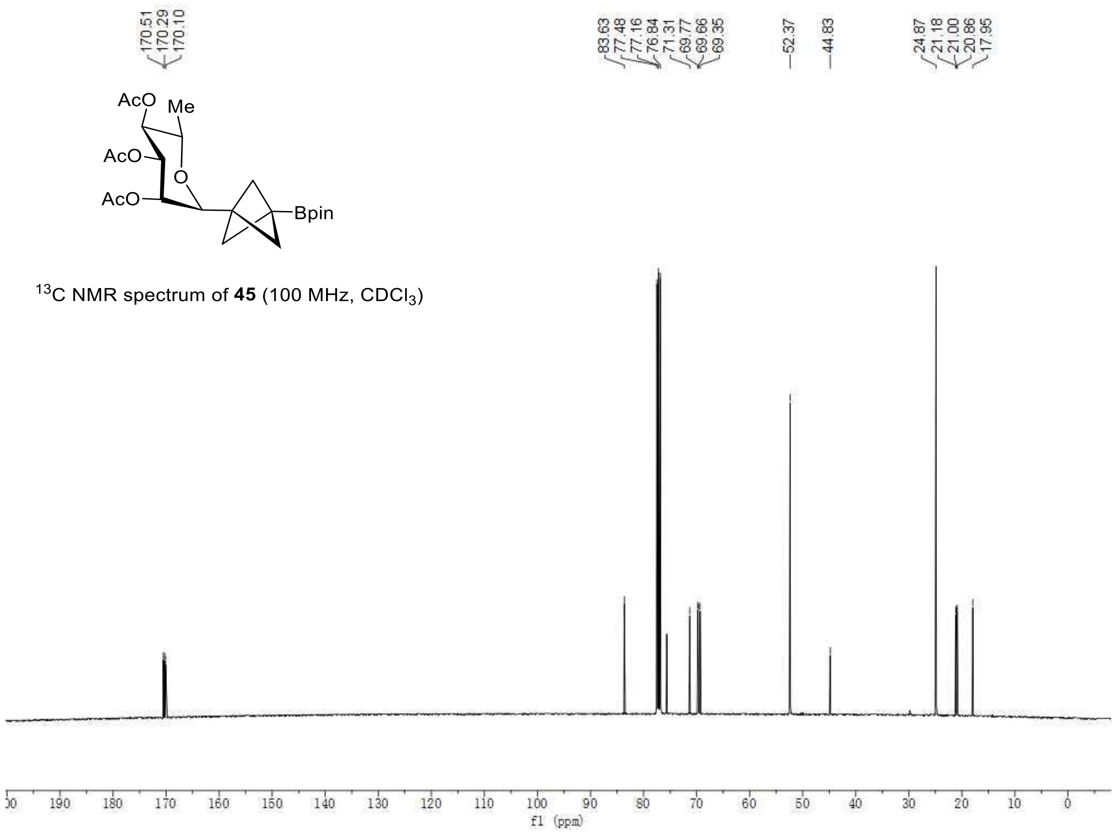

^{13}C NMR spectrum of **45** (100 MHz, $CDCl_3$)

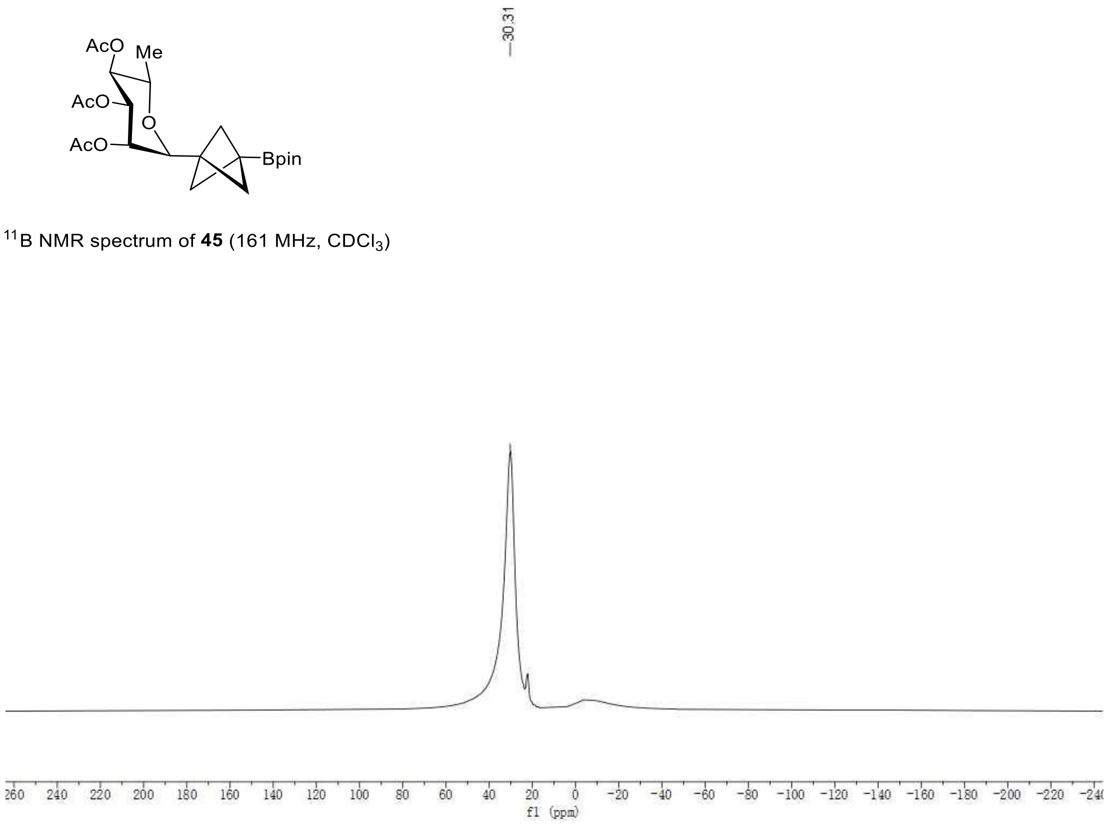

^{11}B NMR spectrum of **45** (161 MHz, $CDCl_3$)

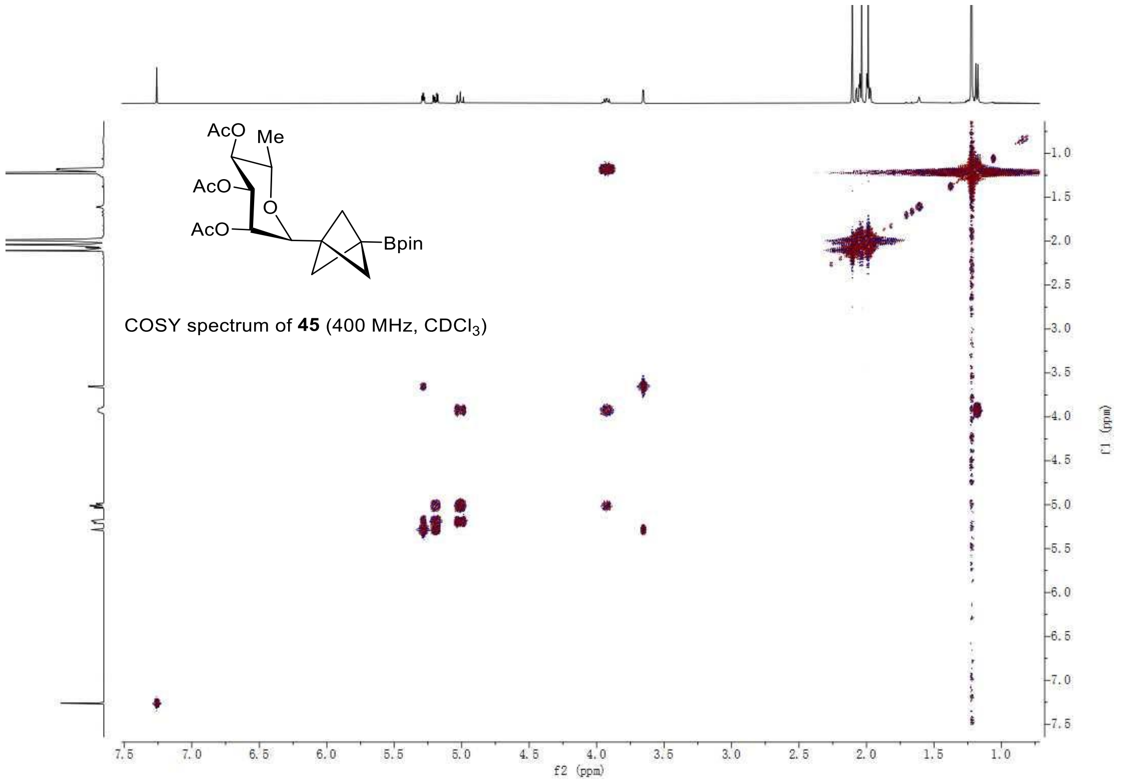

COSY spectrum of **45** (400 MHz, $CDCl_3$)

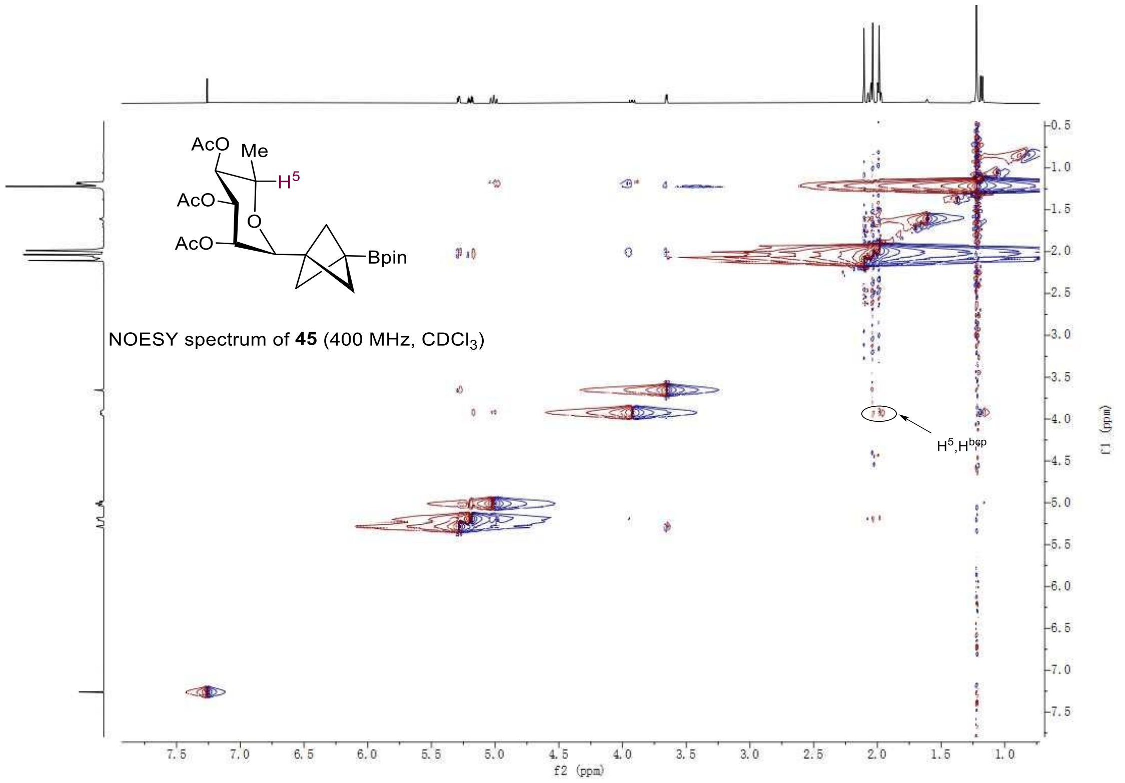

NOESY spectrum of **45** (400 MHz, $CDCl_3$)

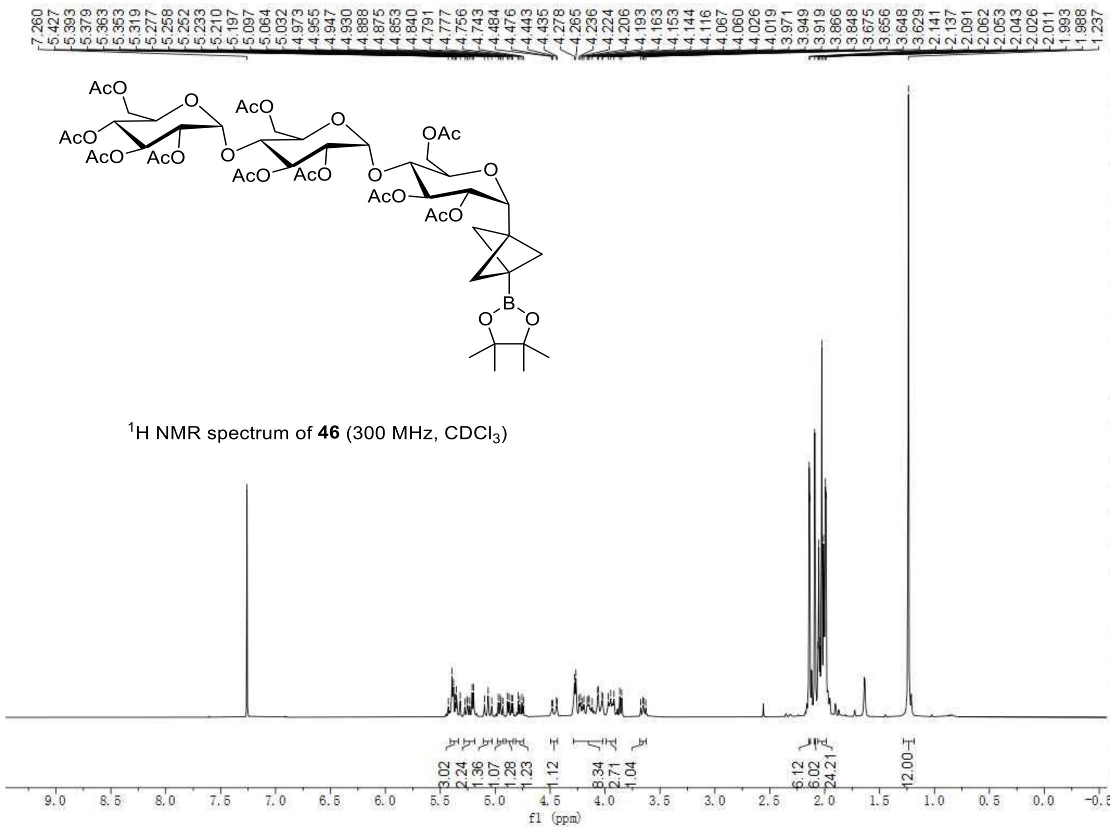

^{1}H NMR spectrum of **46** (300 MHz, $CDCl_3$)

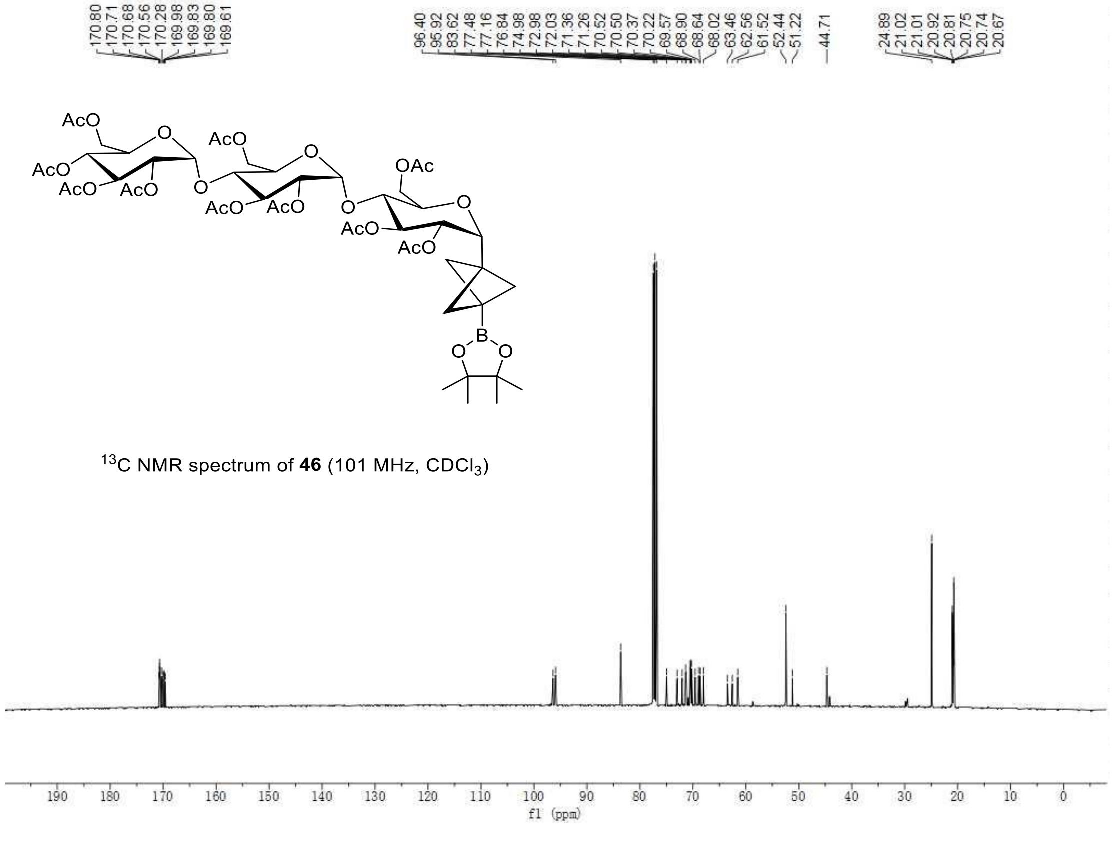

^{13}C NMR spectrum of **46** (101 MHz, $CDCl_3$)

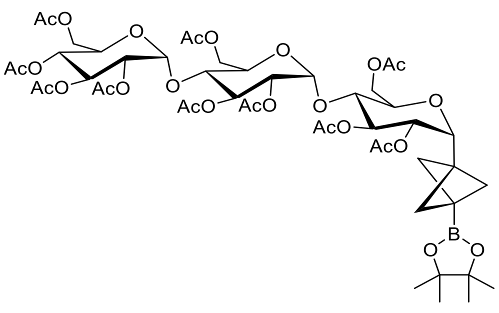

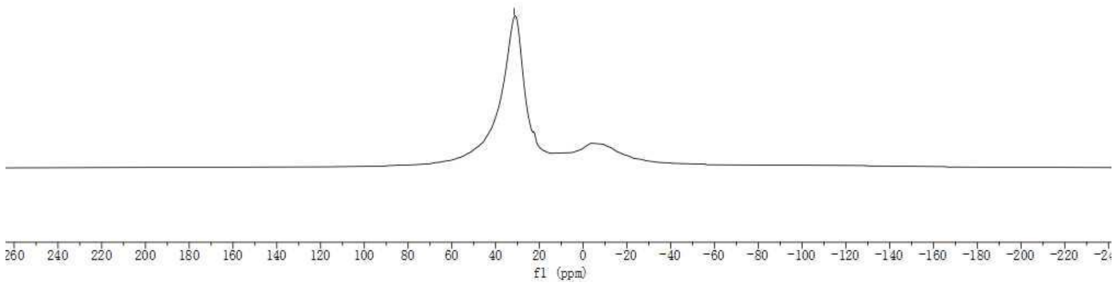

^{11}B NMR spectrum of **46** (161 MHz, $CDCl_3$)

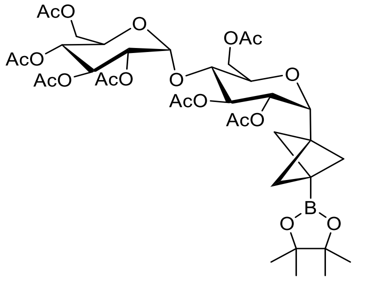

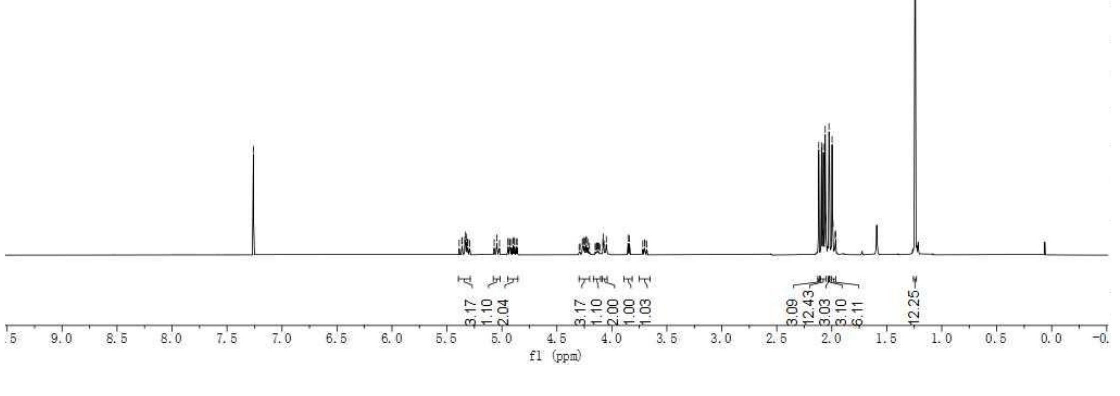

^{1}H NMR spectrum of **47** (400 MHz, $CDCl_3$)

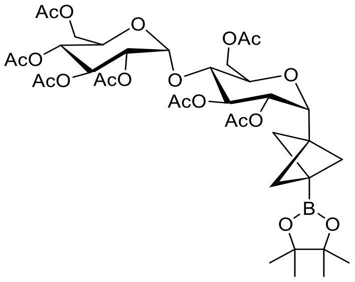

^{13}C NMR spectrum of **47** (100 MHz, $CDCl_3$)

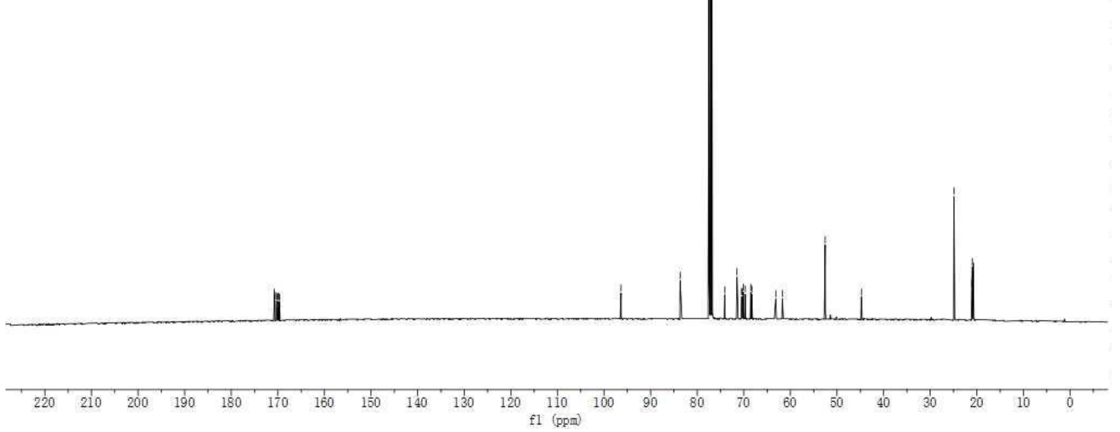

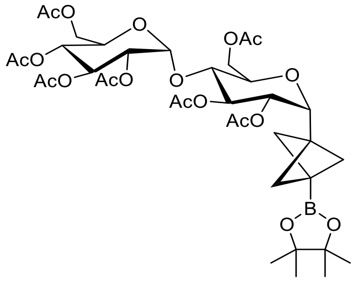

^{11}B NMR spectrum of **47** (161 MHz, $CDCl_3$)

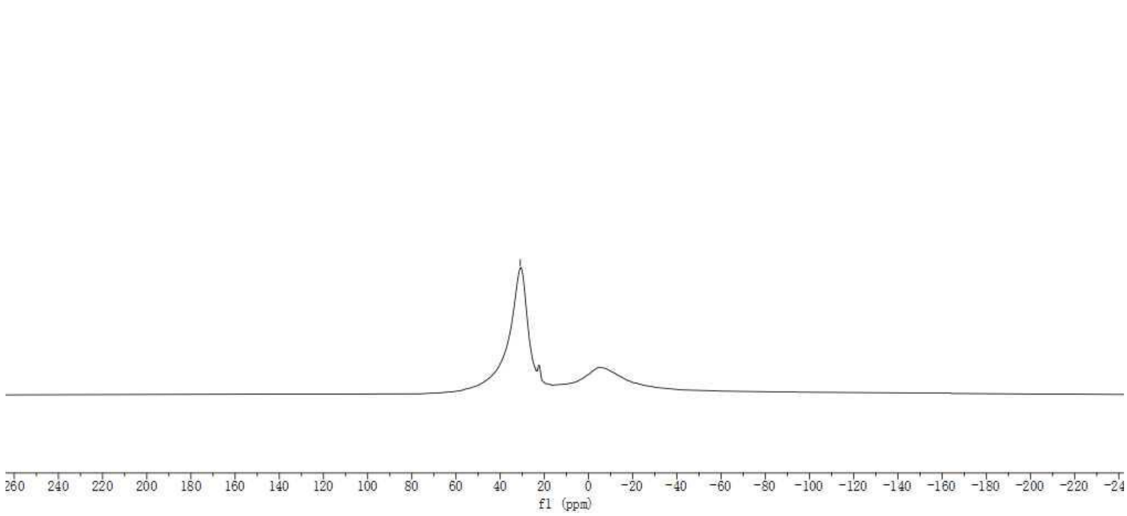

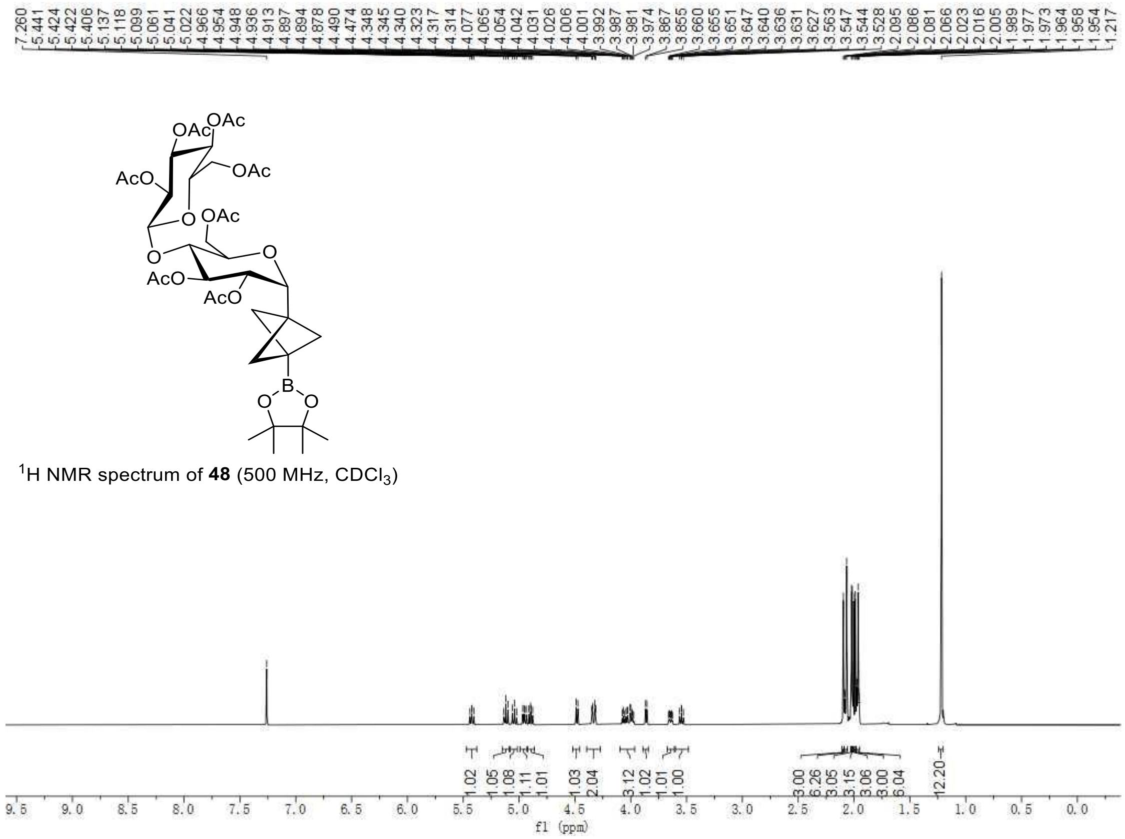

^{1}H NMR spectrum of **48** (500 MHz, $CDCl_3$)

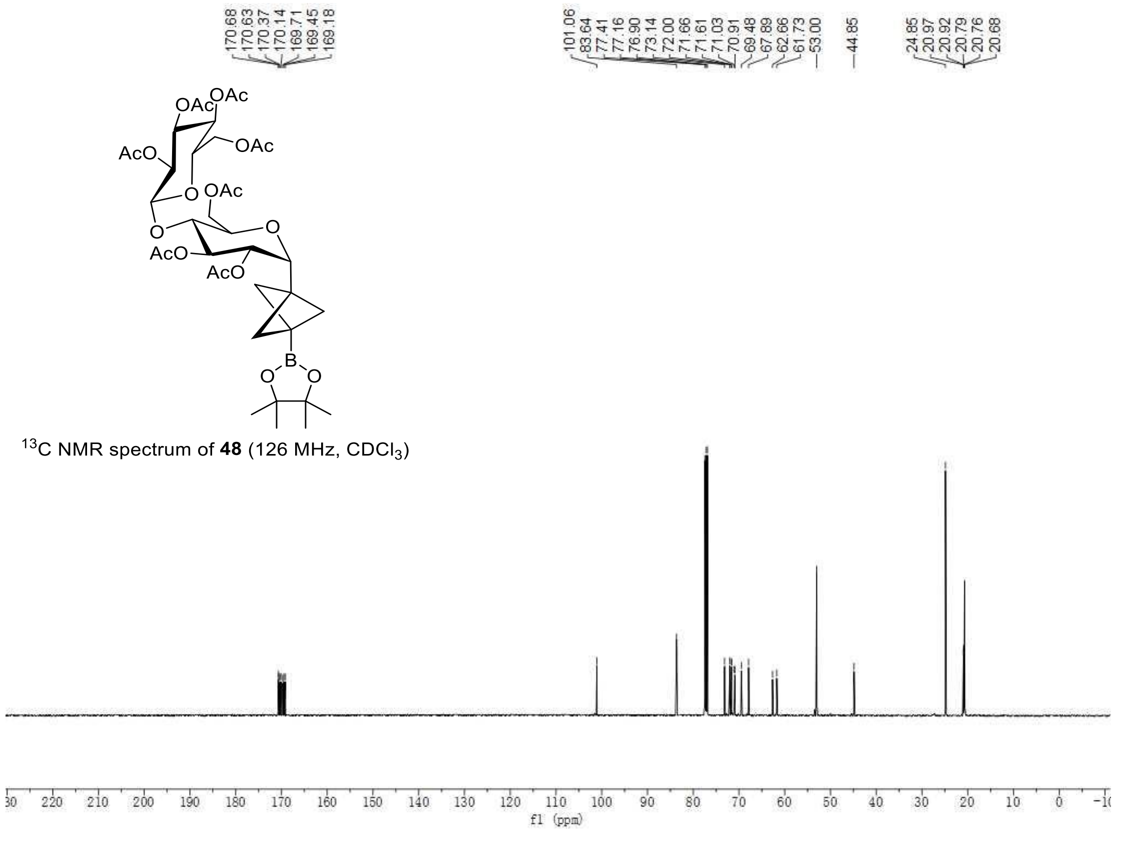

^{13}C NMR spectrum of **48** (126 MHz, $CDCl_3$)

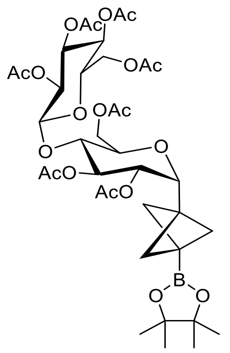

^{11}B NMR spectrum of **48** (161 MHz, $CDCl_3$)

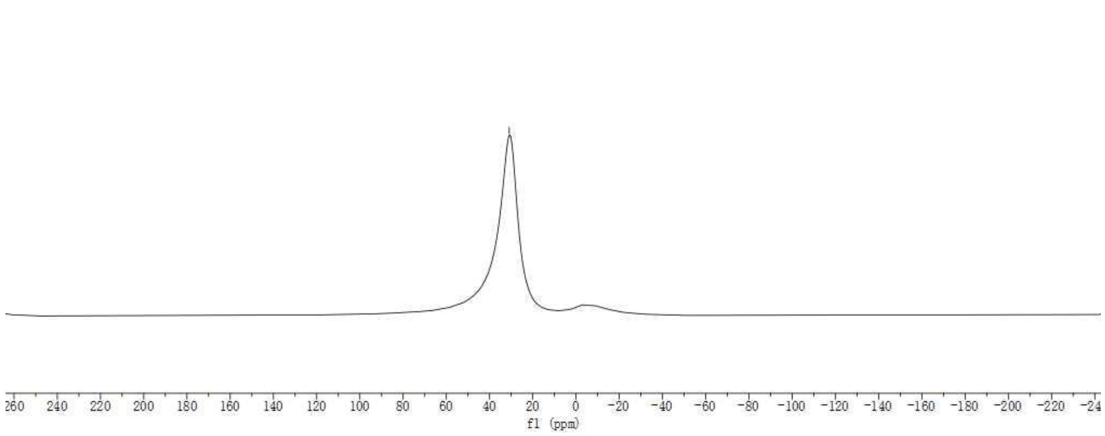

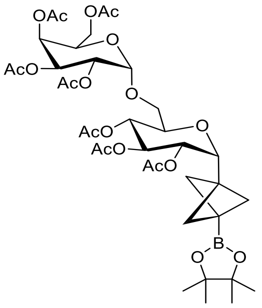

^{1}H NMR spectrum of **49** (400 MHz, $CDCl_3$)

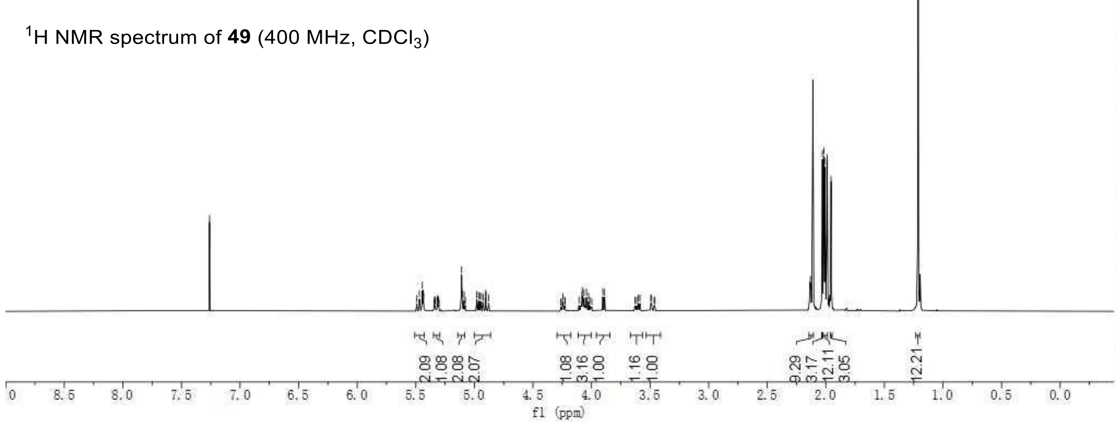

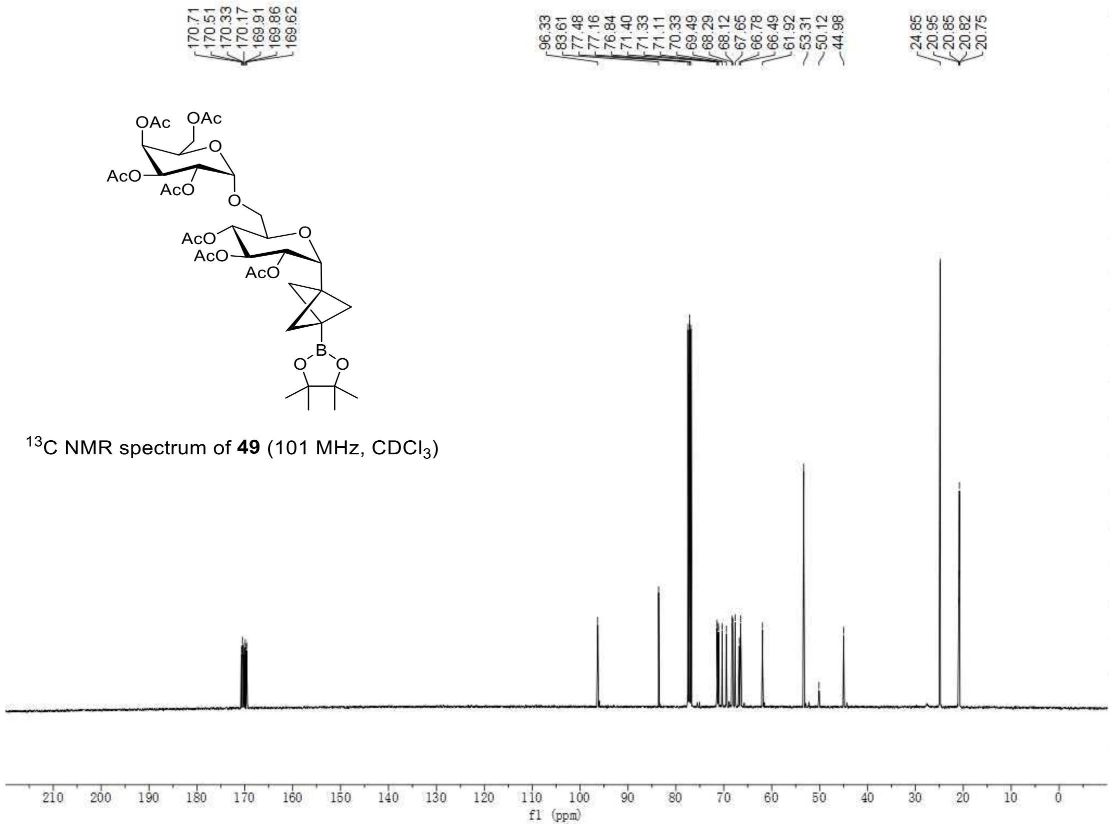

^{13}C NMR spectrum of **49** (101 MHz, $CDCl_3$)

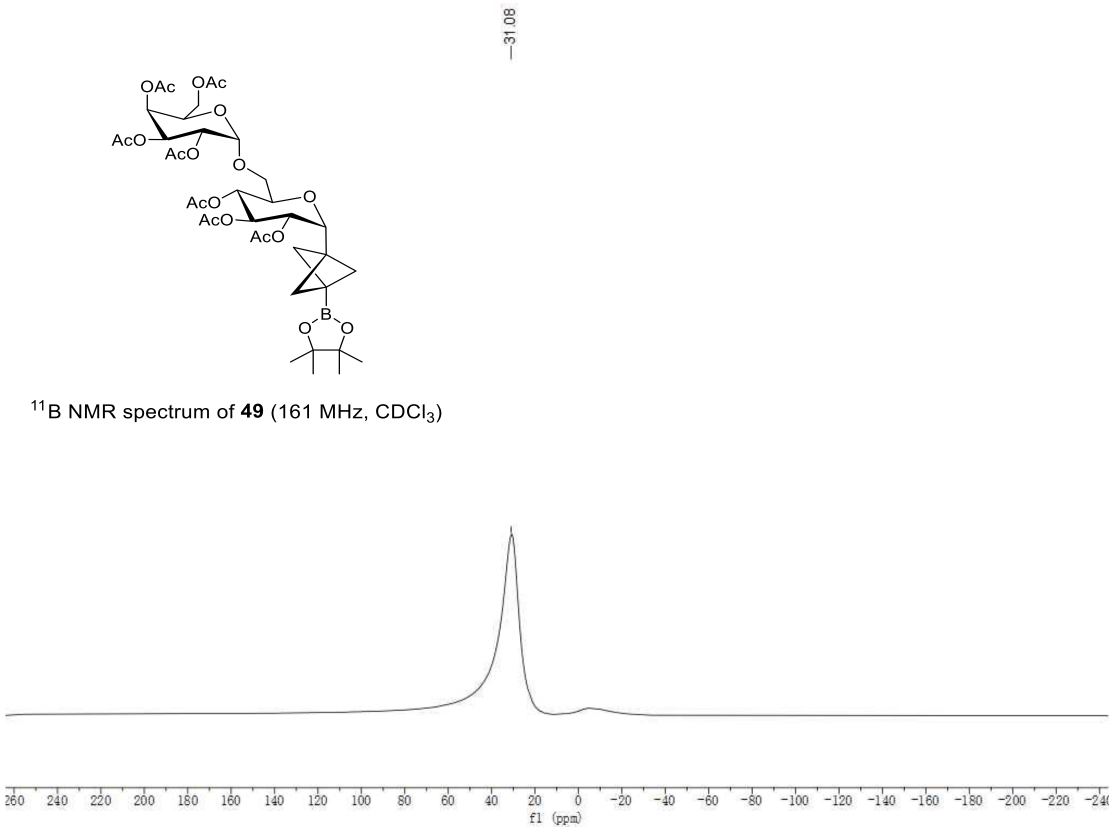

^{11}B NMR spectrum of **49** (161 MHz, $CDCl_3$)

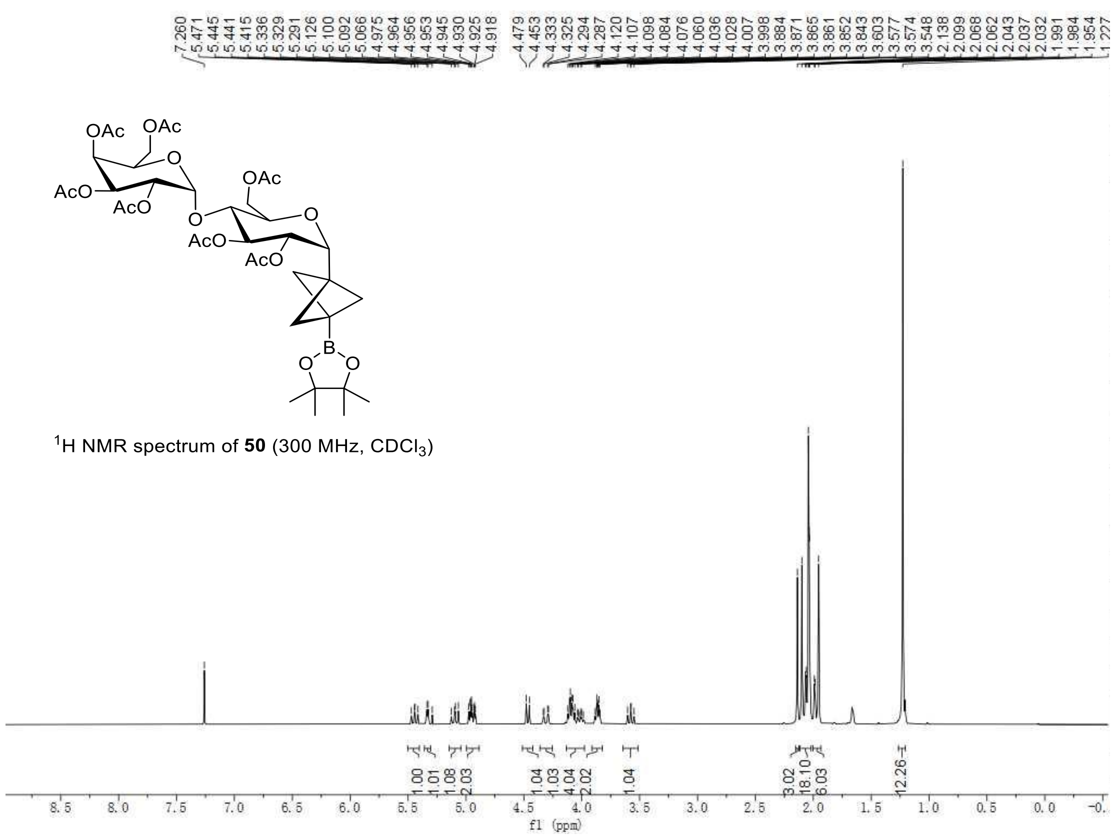

^{1}H NMR spectrum of **50** (300 MHz, $CDCl_3$)

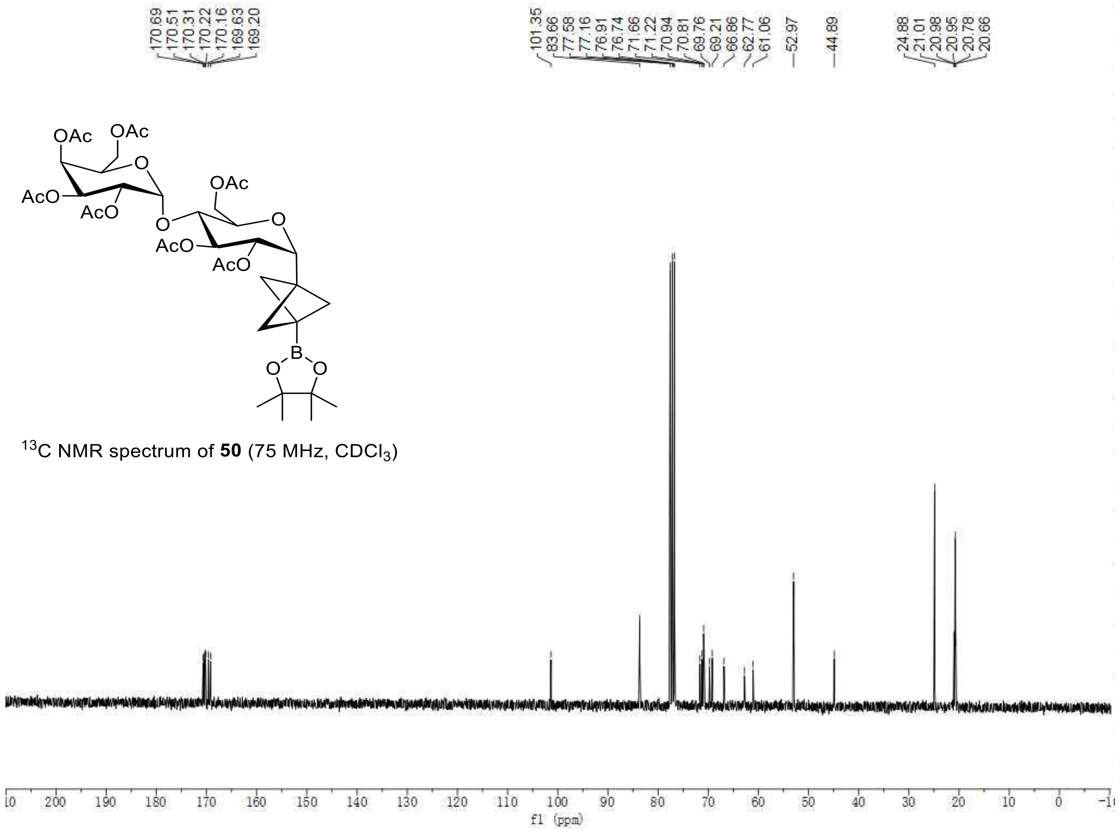

^{13}C NMR spectrum of **50** (75 MHz, $CDCl_3$)

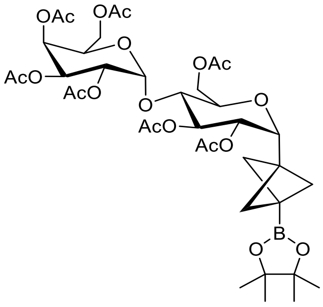

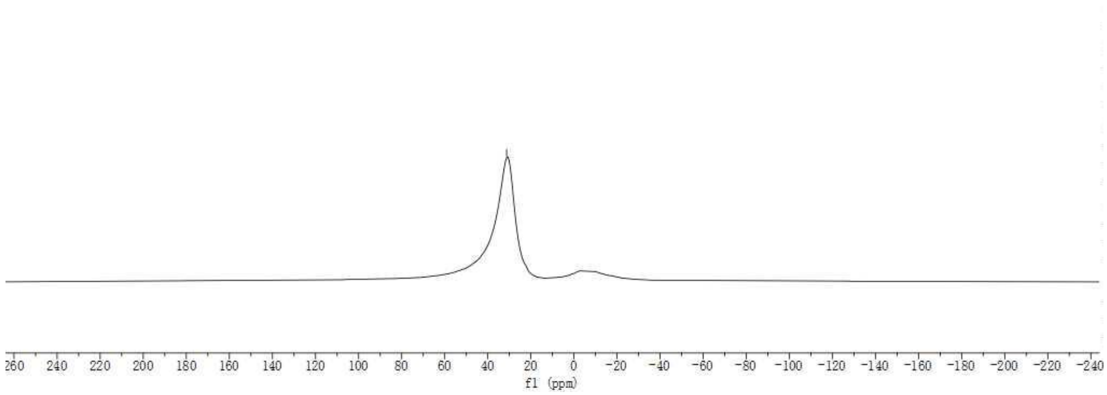

^{11}B NMR spectrum of **50** (161 MHz, $CDCl_3$)

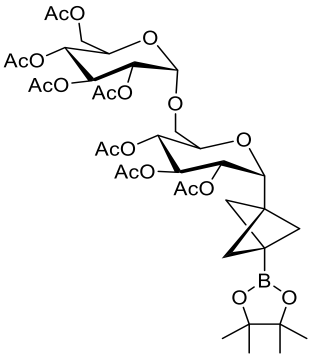

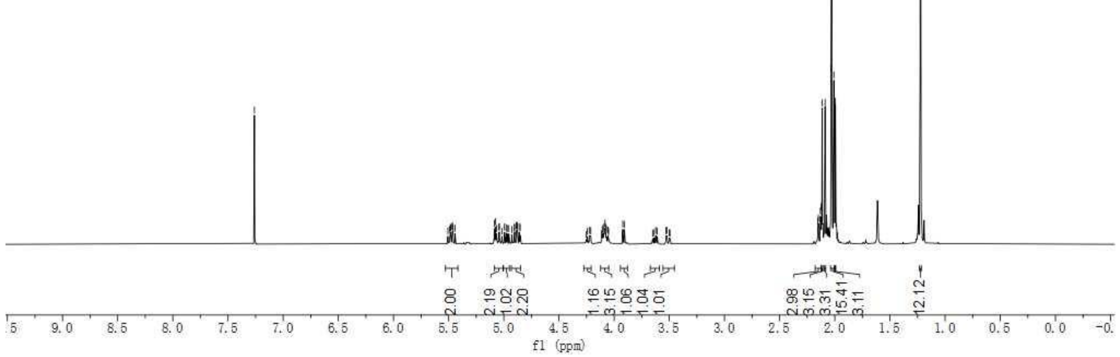

^{1}H NMR spectrum of **51** (400 MHz, $CDCl_3$)

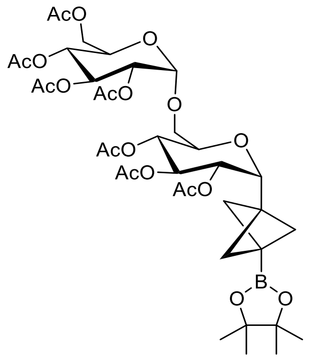

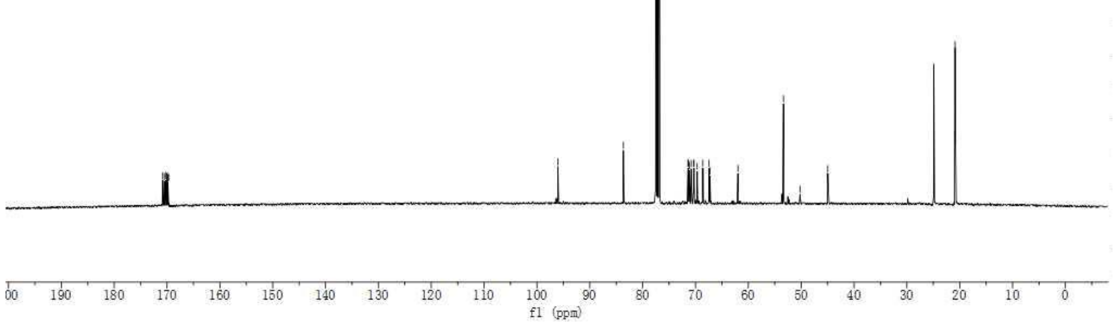

^{13}C NMR spectrum of **51** (101 MHz, $CDCl_3$)

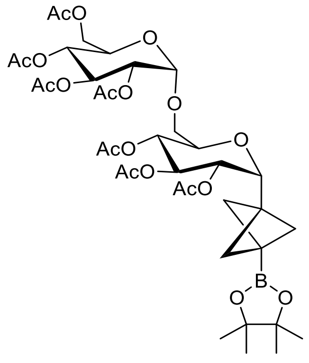

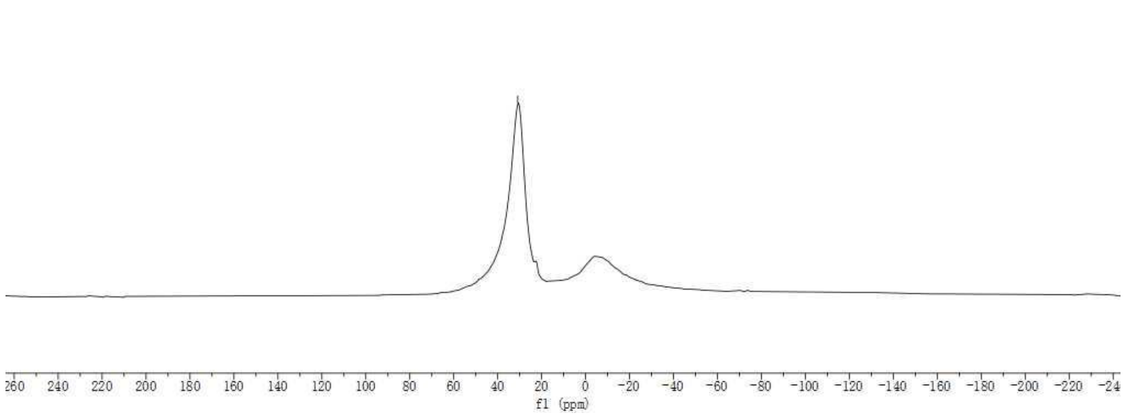

^{11}B NMR spectrum of **51** (161 MHz, $CDCl_3$)

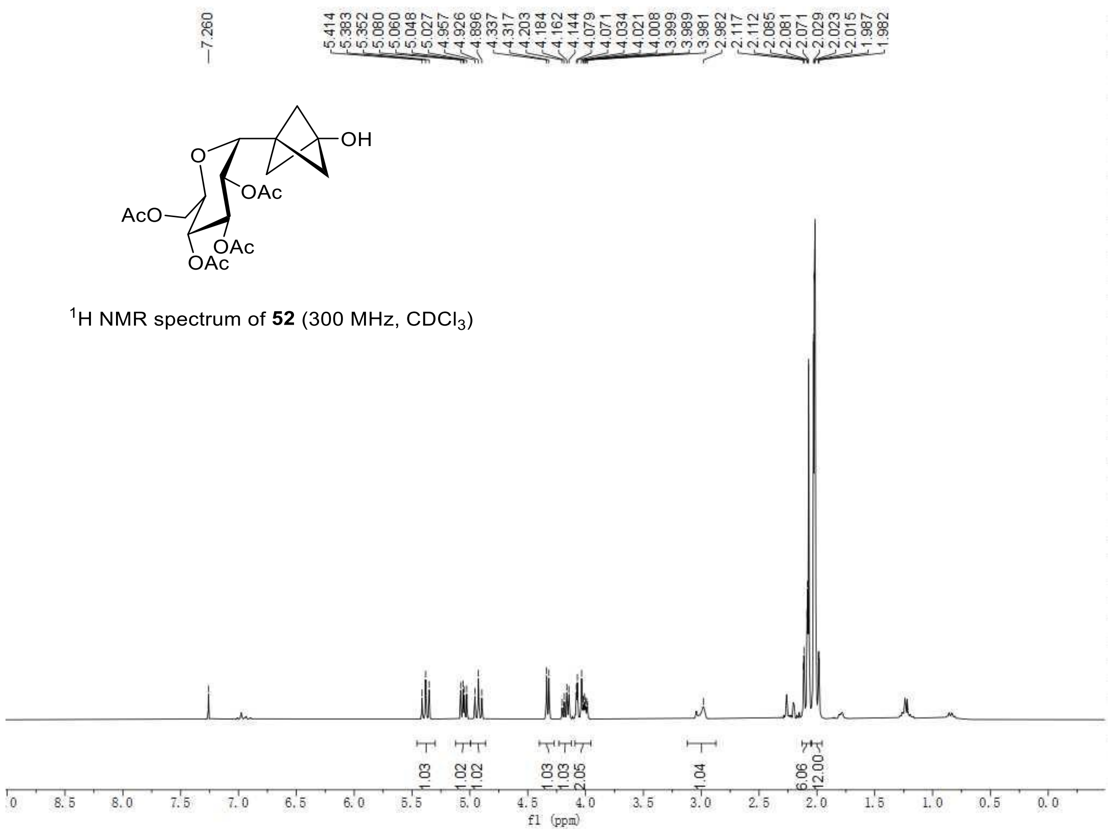

^{1}H NMR spectrum of **52** (300 MHz, $CDCl_3$)

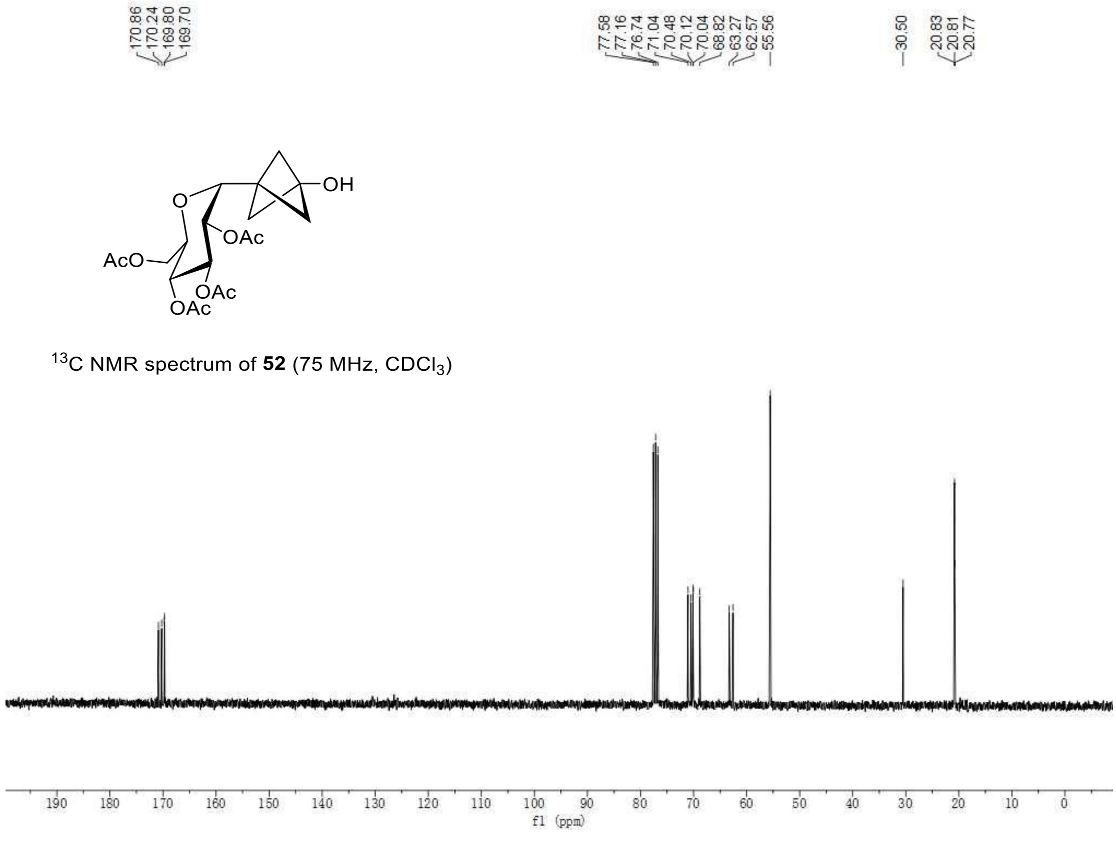

^{13}C NMR spectrum of **52** (75 MHz, $CDCl_3$)

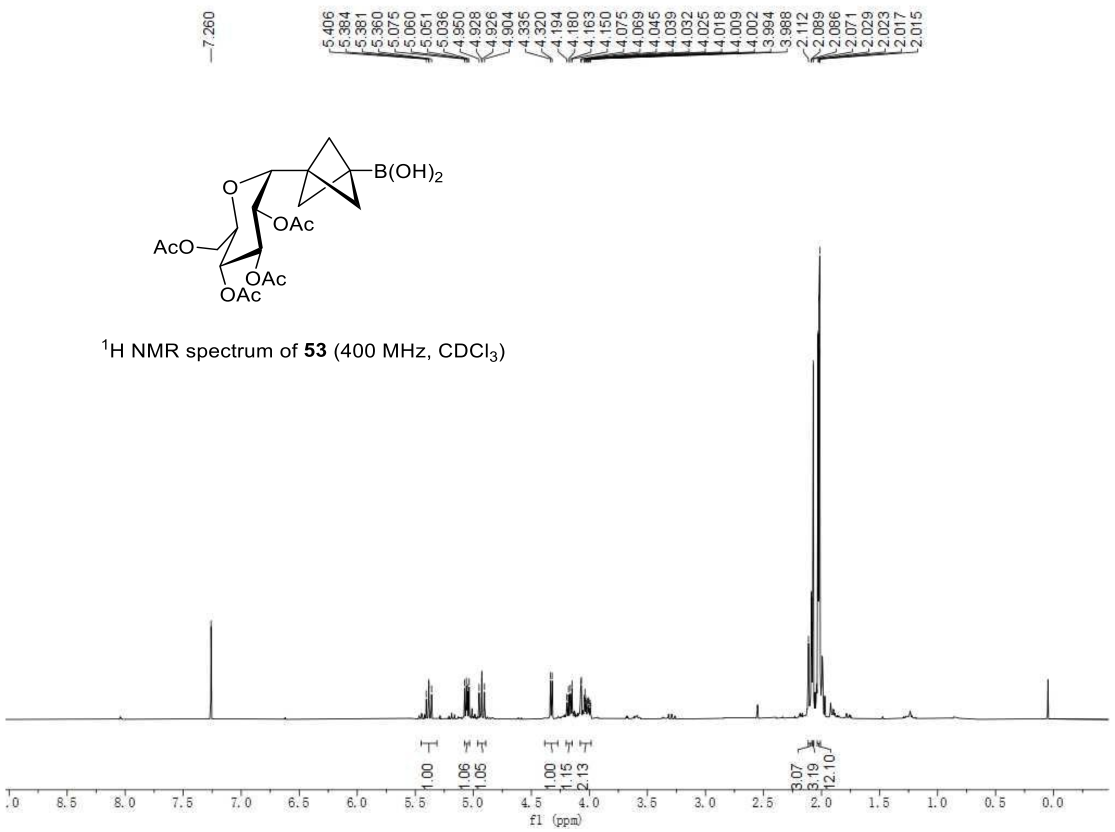

^{1}H NMR spectrum of **53** (400 MHz, $CDCl_3$)

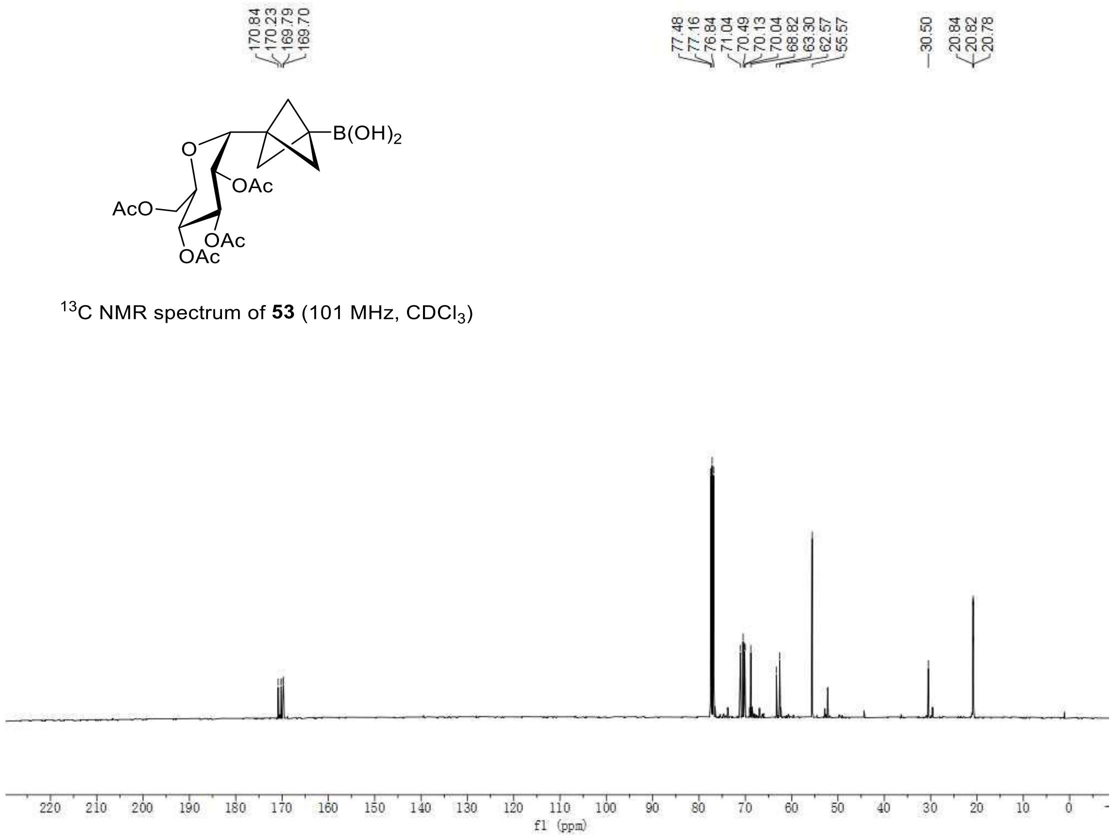

^{13}C NMR spectrum of **53** (101 MHz, $CDCl_3$)

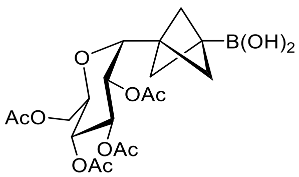

^{11}B NMR spectrum of **53** (161 MHz, $CDCl_3$)

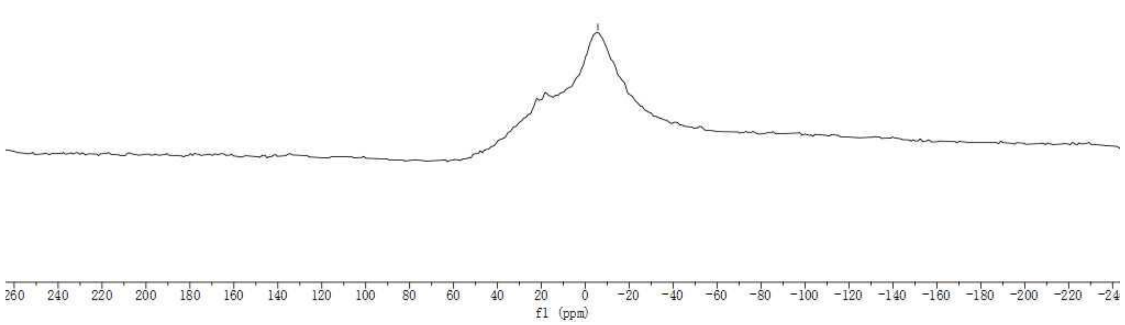

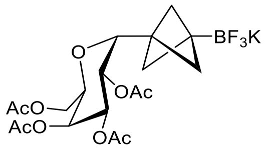

^{1}H NMR spectrum of **54** (500 MHz, DMSO-d_6)

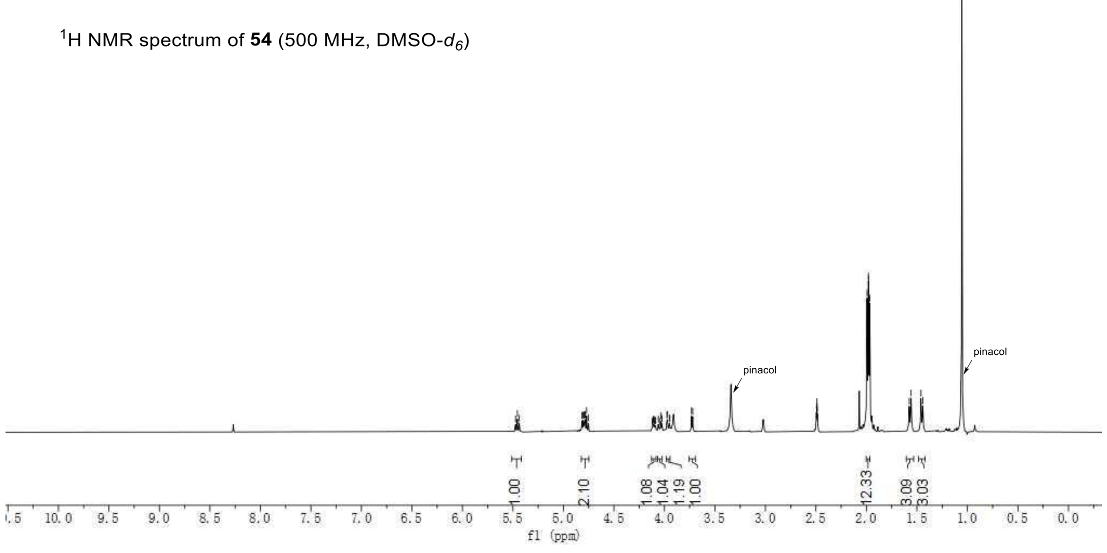

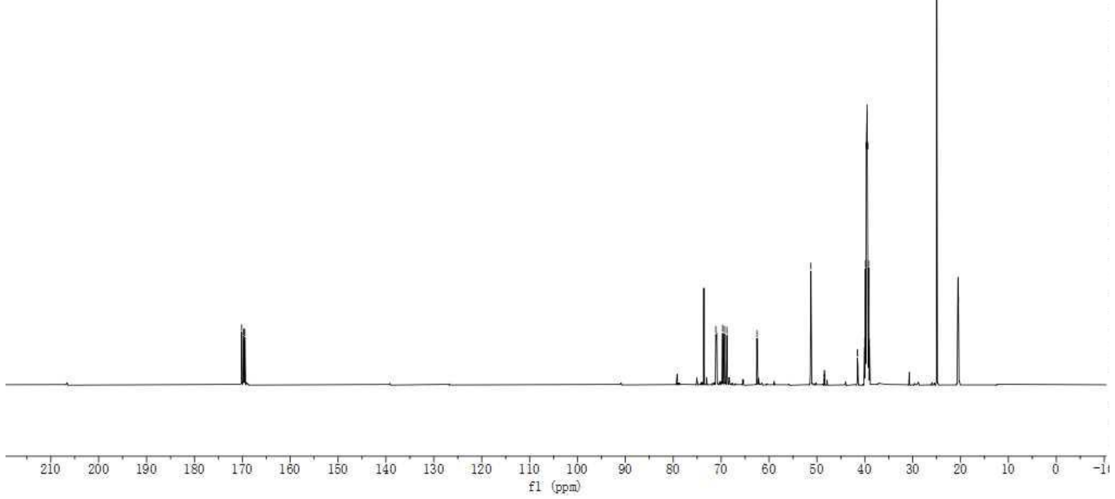

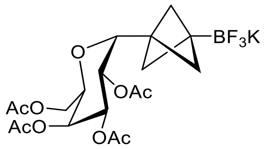

^{13}C NMR spectrum of **54** (126 MHz, DMSO-d_6)

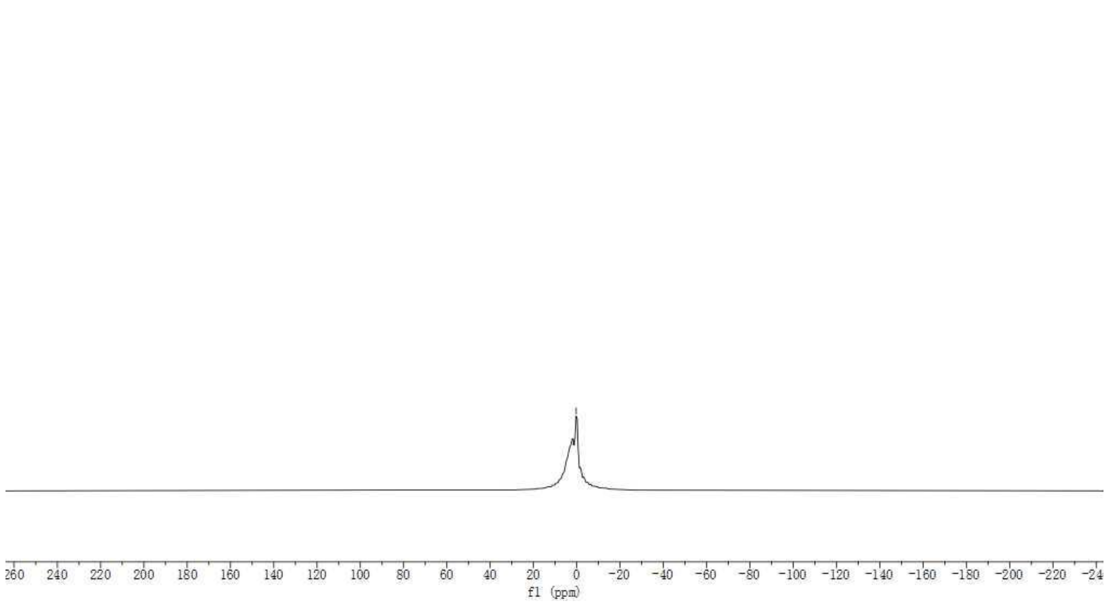

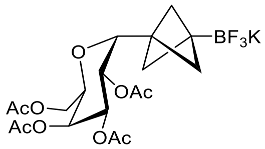

^{11}B NMR spectrum of **54** (161 MHz, DMSO-d_6)

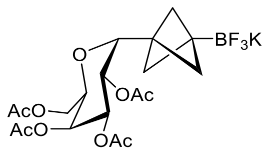

^{19}F NMR spectrum of **54** (282 MHz, DMSO-d_6)

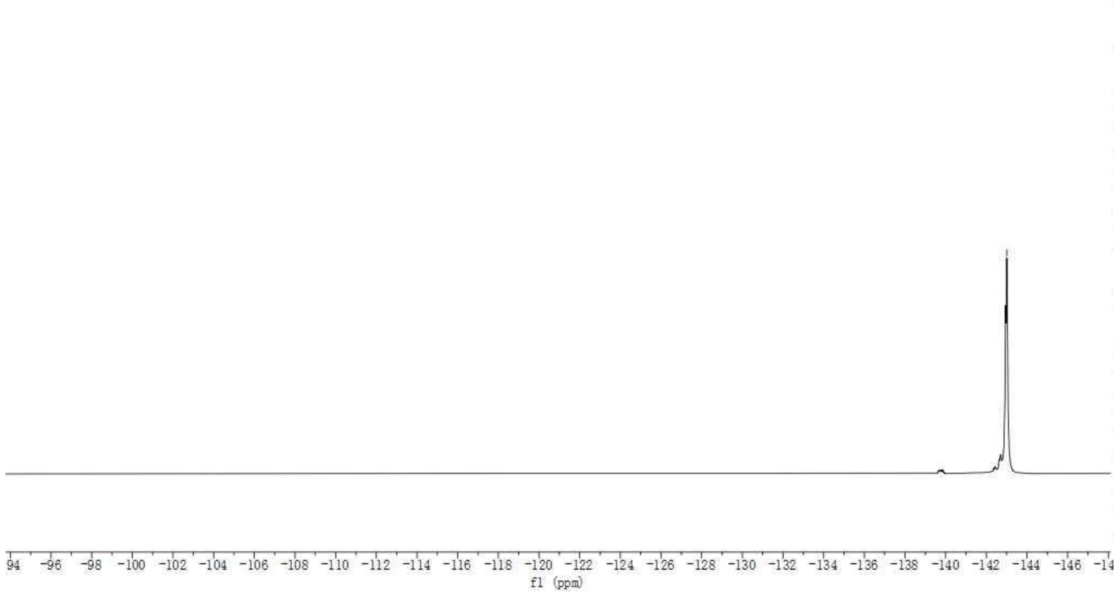

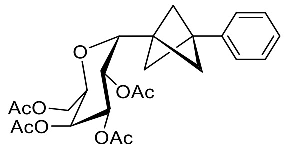

^{1}H NMR spectrum of **55** (300 MHz, $CDCl_3$)

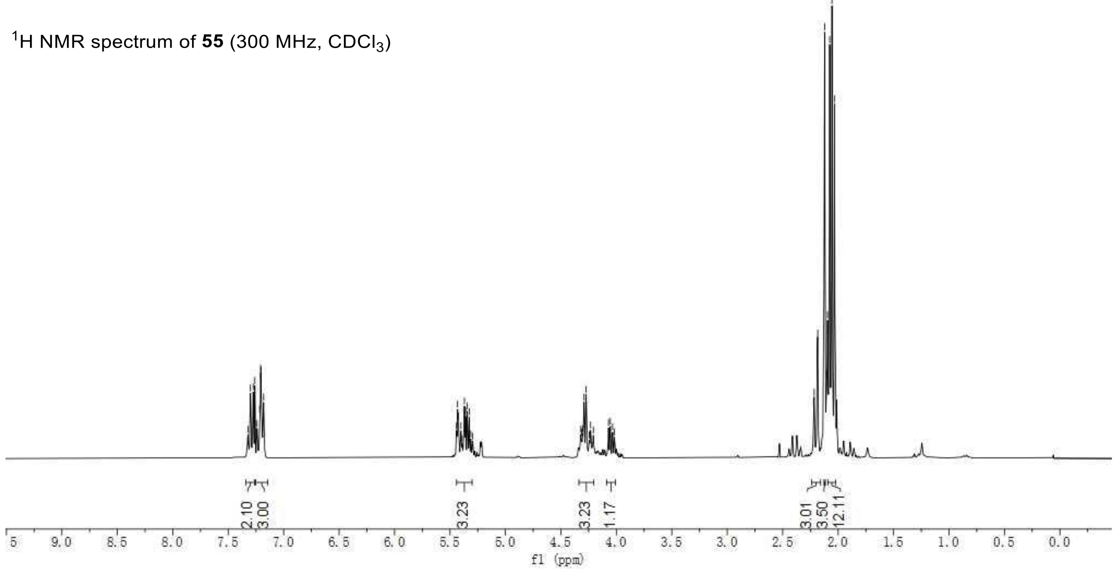

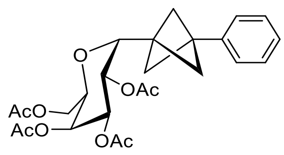

^{13}C NMR spectrum of **55** (75 MHz, $CDCl_3$)

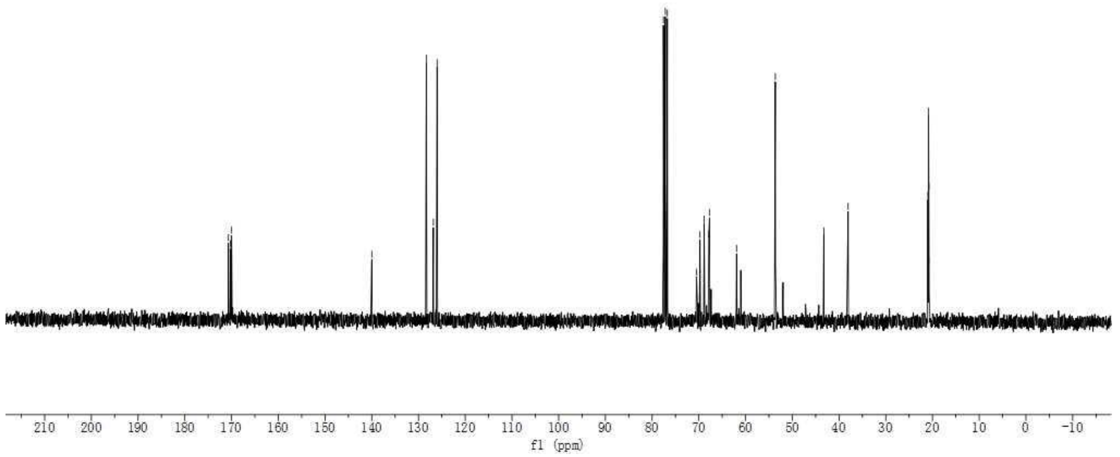

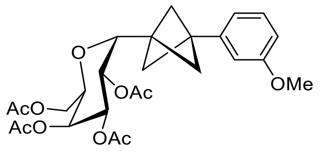

^{1}H NMR spectrum of **56** (300 MHz, $CDCl_3$)

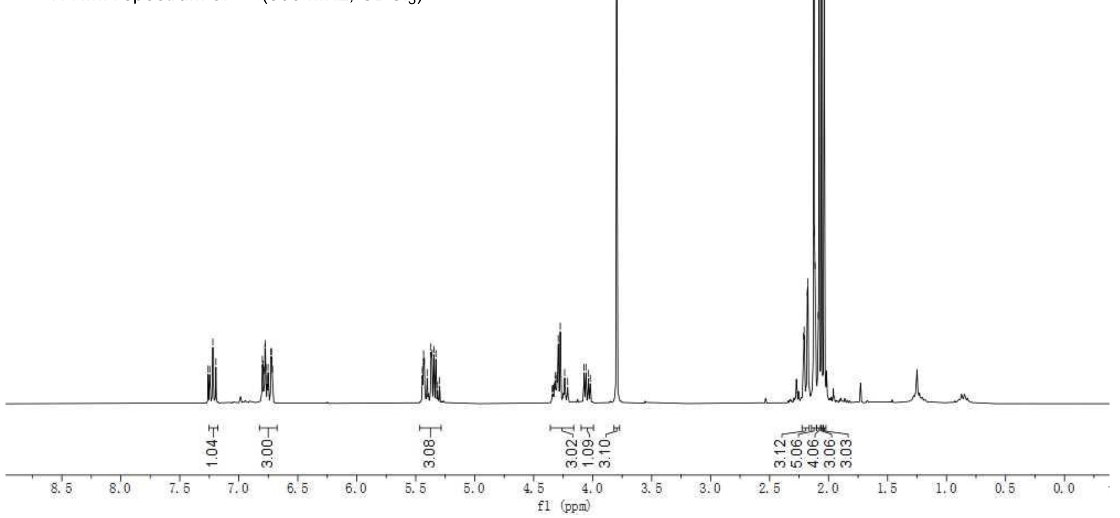

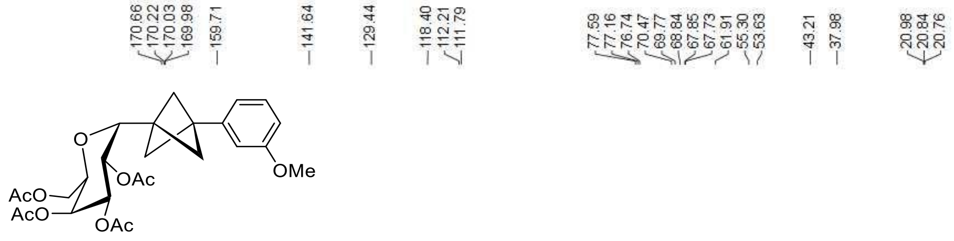

^{13}C NMR spectrum of **56** (75 MHz, $CDCl_3$)

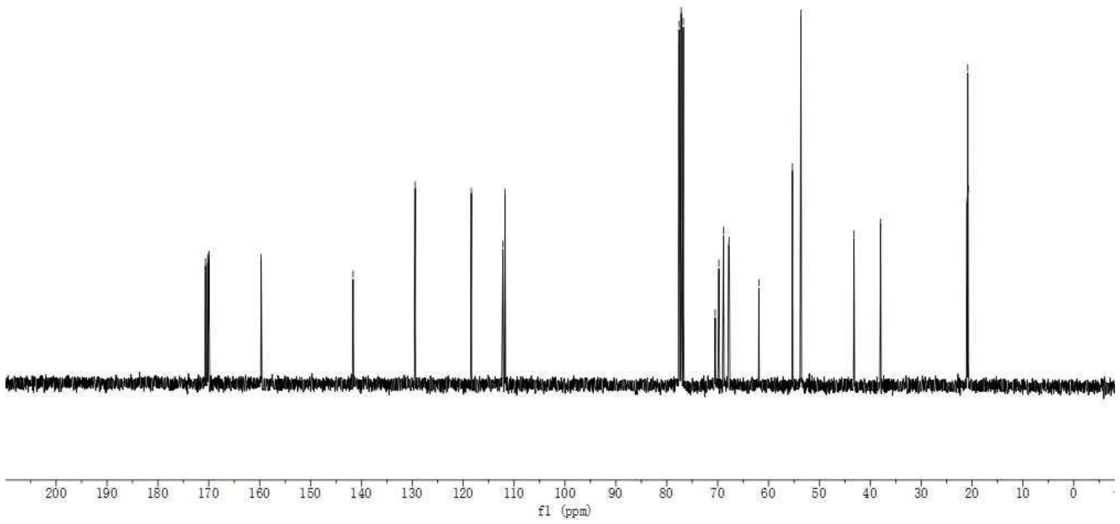

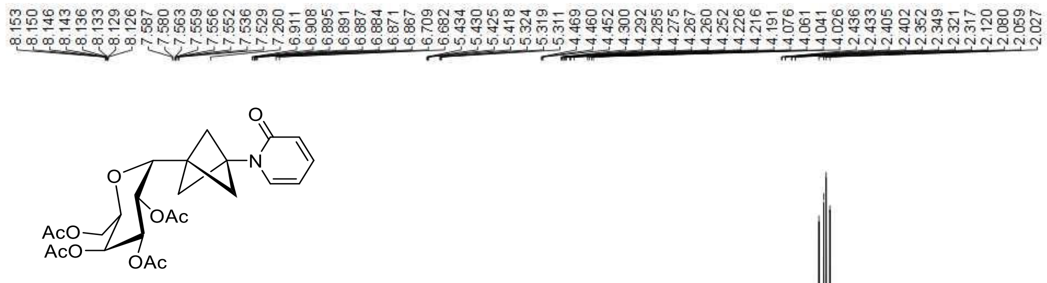

^{1}H NMR spectrum of **57** (300 MHz, $CDCl_3$)

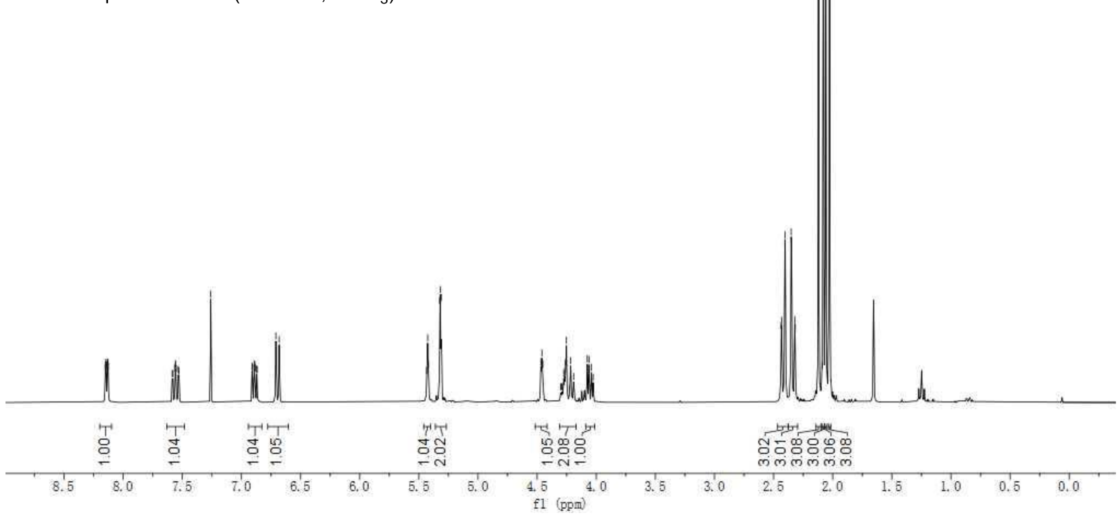

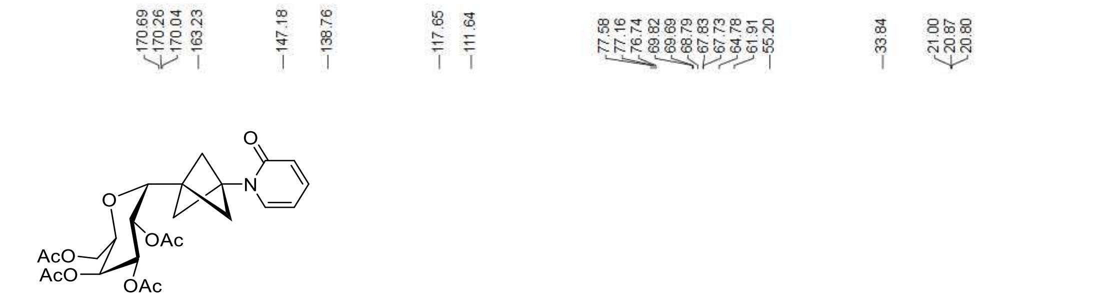

^{13}C NMR spectrum of **57** (75 MHz, $CDCl_3$)

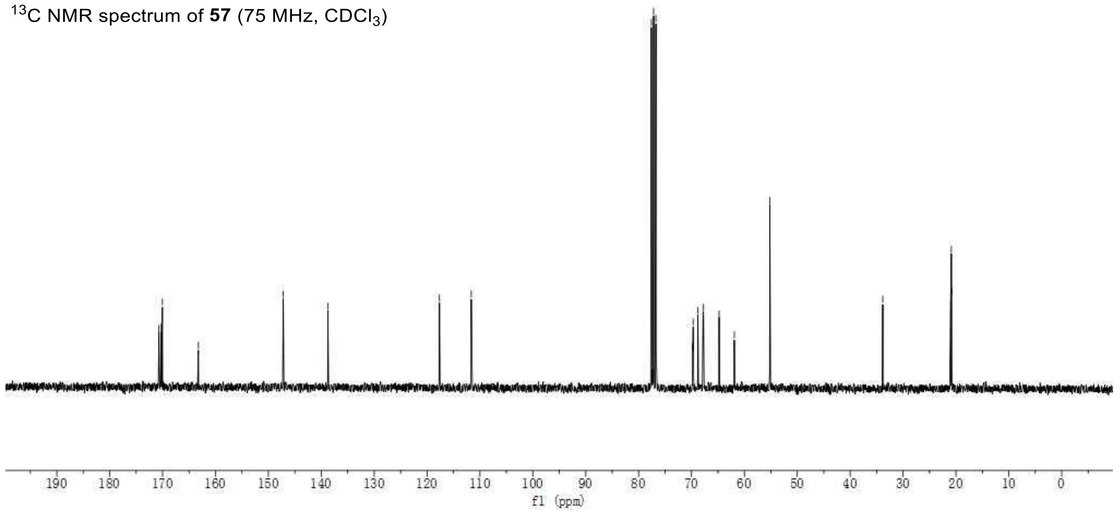

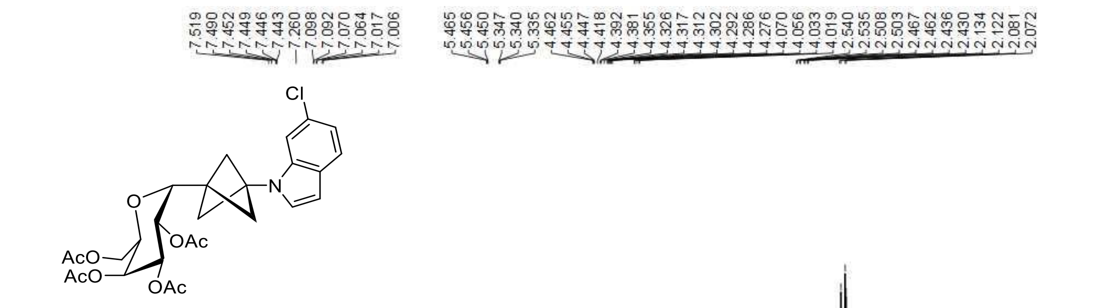

^{1}H NMR spectrum of **58** (300 MHz, $CDCl_3$)

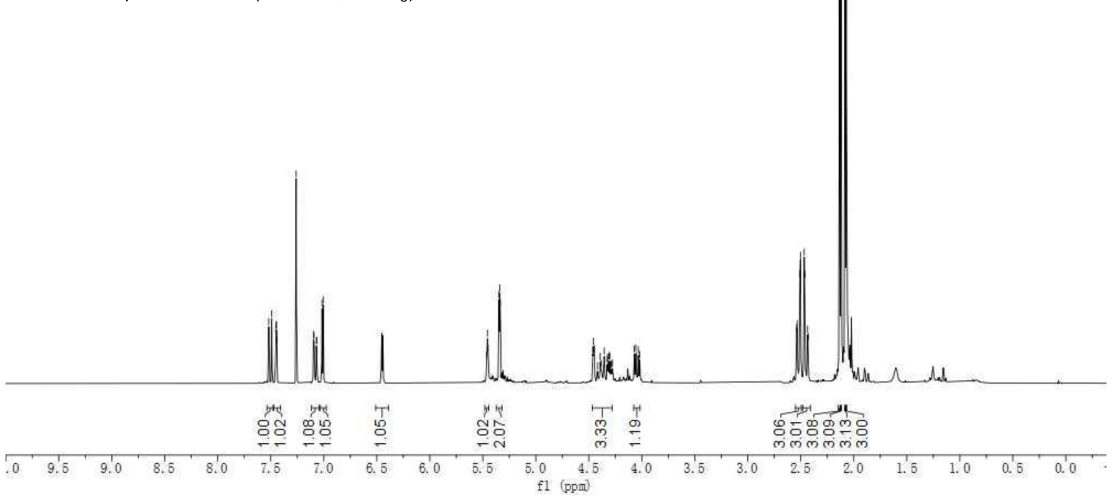

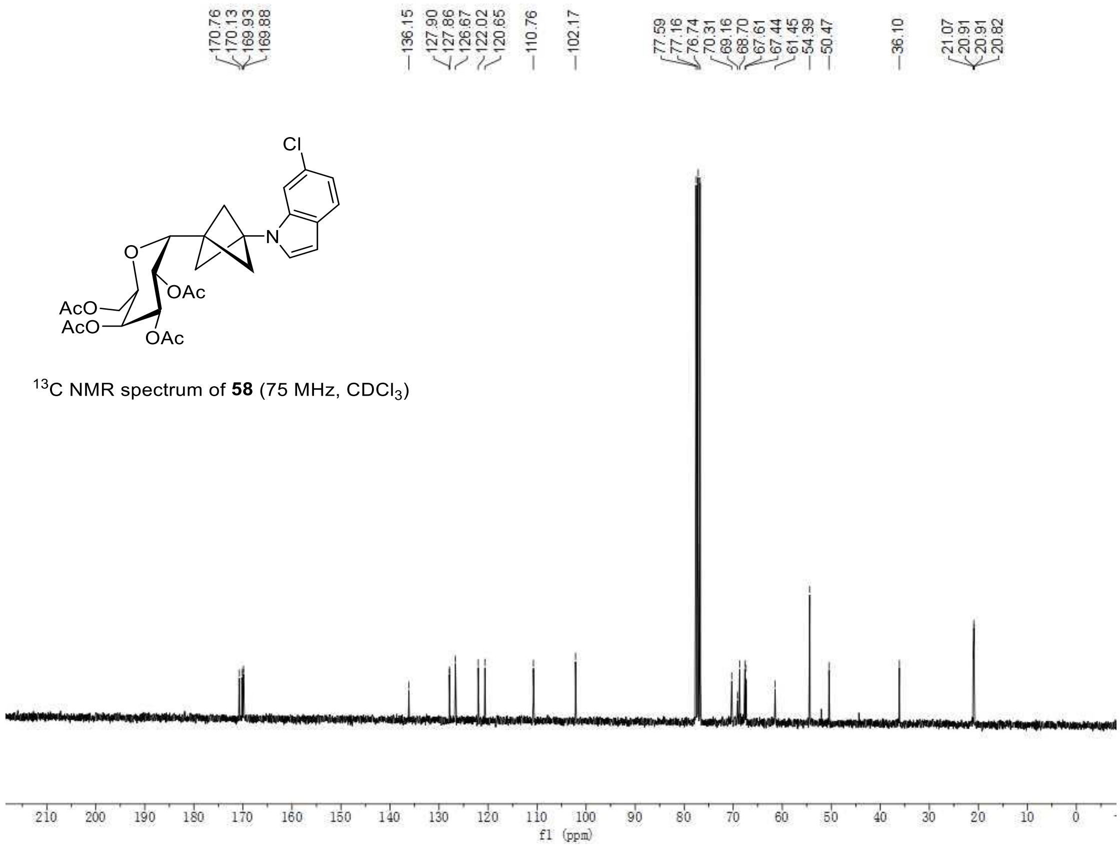

^{13}C NMR spectrum of **58** (75 MHz, $CDCl_3$)

References

1. Nugent, J.; Arroniz, C.; Shire, B. R.; Sterling, A. J.; Pickford, H. D.; Wong, M. L. J.; Mansfield, S. J.; Caputo, D. F. J.; Owen, B.; Mousseau, J. J.; Duarte, F.; and Anderson, E. A. A general route to bicyclo[1.1.1]pentanes through photoredox catalysis. *ACS Catal.* **2019**, *9*, 9568–9574.

2. Merchant, R. R.; Lopez, J. A. A general C(sp^3)−C(sp^3) cross-coupling of benzyl sulfonylhydrazones with alkyl boronic acids. *Org. Lett.* **2020**, *22*, 2271–2275.

3. Nugent, J.; Shire, B. R.; Caputo, D. F. J.; Pickford, H. D.; Nightingale, F.; Houlsby, I. T. T.; Mousseau, J. J.; and Anderson, E. A. Synthesis of all-carbon disubstituted bicyclo[1.1.1]pentanes by iron-catalyzed kumada cross-coupling. *Angew. Chem. Int. Ed.* **2020**, *59*, 11866–11870.

4. Alonso, M.; Cañellas, S.; Delgado, F.; Serrano, M.; Diéguez-Vázquez, A.; and Gómez, J. E. Accelerated synthesis of bicyclo[1.1.1]pentylamines: a high-throughput approach. *Org. Lett.* **2023**, *25*, 771−776.

5. Barton, L. M.; Chen, L.; Blackmond, D. G.; Baran, P. S. Electrochemical borylation of carboxylic acids. *Proc. Natl. Acad. Sci. U. S. A.* **2021**, *118*, e2109408118.

6. Dong, W.; Yen-Pon, E.; Li, L.; Bhattacharjee, A.; Jolit, A.; Molander, G. A. Exploiting the sp^2 Character of Bicyclo[1.1.1]pentyl Radicals in the Transition-Metal-Free Multicomponent Difunctionalization of [1.1.1]Propellane. *Nat. Chem.* **2022**, *14*, 1068–1077.
